# Supplementary material for: Widespread and increased drilling of wells into fossil aquifers in the USA
Source: Nat Commun. 2022 Apr 19;13:2129. doi: 10.1038/s41467-022-29678-7 (PMC9018791; doi:10.1038/s41467-022-29678-7)
Supplement: Supplementary file 1 — Supplementary Information [file 41467_2022_29678_MOESM1_ESM.pdf]

Supplementary Information for

Widespread and increased drilling of wells into fossil aquifers in the USA

GebreEgziabher, M.*, Jasechko, S., Perrone, D.
*corresponding author: gebremichael@ucsb.edu

Contents

Supplementary Note 1 – Evidence of fossil groundwater prevalence in the US 2
Supplementary Note 1.1 – Meta-analysis of fossil groundwater prevalence 2
Supplementary Note 1.2 – Radiocarbon evidence for fossil groundwater in thousands of US wells 7
Supplementary Note 2 – Supplementary Results 9
Supplementary Note 2.1 – Temporal variations in constructed well depths relative to a nearby well that contains fossil water 9
Supplementary Note 2.2 – Temporal variations in the proportion of wells deeper than 200±100m within the boundaries of aquifer systems 10
Supplementary Note 2.3 – Statistical relationships between the proportion of wells deeper than 200±100m within the boundaries of aquifer systems and groundwater level variations over time 15
Supplementary Note 3 – Delineation of aquifers across the United States 16
Supplementary Note 3.1 – Primary sources and approach to delineate hydrogeologic study areas 16
Supplementary Note 3.2 – Rationale for developing a new geodatabase of US aquifer systems 77
Supplementary Note 4 – Hydrostratigraphy in 12 aquifer systems with fossil water 81
Supplementary Note 4.1 – Categorizing hydrostratigraphy based on local-scale studies 81
Supplementary Note 4.2 – Alternate versions of main text Fig. 1 93
Supplementary Note 5 – Statistical relationships between groundwater level change over time and the prevalence of deep wells 97
Supplementary Note 6 – Groundwater quality in shallow versus deep wells 101
Supplementary Note 6.1 – Compilation of US groundwater quality data 101
Supplementary Note 6.2 – Groundwater quality in deep and shallow wells in aquifer systems 102
Supplementary Note 7 – Examples of marketing linked to the age of water 106
Supplementary Note 8 – Map of US Aquifer Database 107

Supplementary Note 1 – Evidence of fossil groundwater prevalence in the US

Supplementary Note 1.1 – Meta-analysis of fossil groundwater prevalence

Here we tabulate publications that suggest fossil groundwater may exist in an aquifer system. Most of the consulted references rely on radioisotope measurements in well waters (e.g., ^{14}C, ^{36}Cl).

Supplementary Table 1. Meta-analysis of fossil groundwater prevalence

**Aquifer**	**Broader Aquifer System**	**References* presenting evidence of fossil groundwater (quotes or reference to figures or tables that suggest fossil water is present)**
San Pedro Basin	-	"The Pearson-Hanshaw model described previously predicted that even the corrected ages for the two deep wells near the riparian zone with less than 13 pMC were more than 15,000 years (Wahi 2005)." Wahi et al. (2008)
Santa Maria Basin and Nipomo Valley	-	"The total mean ages ranged from 52.9 to 59.1 years (median value: 56.3 years) for modern groundwater, 2748 to 54,958 years (median value: 13,876 years) for pre-modern groundwater and 799 to 16,959 years (median value: 2746 years) for mixed waters." Hagedorn et al. (2018)
Michigan Basin	-	"These results are consistent with ^{14}C ages and oxygen isotope values of confined groundwaters in Devonian carbonates along the basin margin, which reflect past recharge beneath the Laurentide Ice Sheet (14–50 ka)" McIntosh et al. (2011)
Albuquerque Basin	Middle Rio Grande	See Figure 10 by Plummer et al. (2004)
Northern Cambrian-Ordovician Aquifers	Northern Midwest Aquifer System	See Figure 2 and Table A.3 of the Supplemental Information (Solder & Jurgens, (2020)
Tulare Basin	California Central Valley	See Figure 5 OR "Of the 111 groundwater samples assigned to an age category based on the 3H and 14C data, the majority (69%) are premodern, followed by mixed age (25%) and modern (6%)." McMahon et al. (2021)
Snake Valley	-	See Figure 9 Gardner and Heilweil (2014)
Southern San Juan Basin	-	"Ages range from young (^{14}C dates of less than 34,000 years [Mavor et al., 1991]) to very old (^{4}He residence times of up to 123 million years, Sanford and Sorek, [2003])" S.S. Papadopulos & Associates, Inc, (2006)
Estancia Basin	-	See Table 1 and Figure 1 McMahon et al. (2011)
Maryland Western Shores	North Atlantic Coastal Plain	See Table 2 and See Table 3 by Purdy et al. (2016)
North Carolina and Virginia Coastal Plain	North Atlantic Coastal Plain	See Table 2 OR "The 36Cl ages range from 23 to 328 ka on the northern flow path and 185 to 503 ka on the southern flow path (excluding sample S-10)." Plummer et al. (2012)
Eastern Flatwoods Southshores	Floridan Aquifer System	See Table 2 OR "The isotopic abundances of ^{81}Kr were between 89 pMKr (percent Modern Kr) and modern, suggesting relatively young (<40 kyr) apparent groundwater residence times (See Table 2)." Yokoch et al. (2021)
Espanola Basin	Middle Rio Grande	See Table 3 OR "Unadjusted ^{14}C ages range from 0.1 to 39.5 ka, and adjusted ages range from 0.0 to 35.4 ka (Table 3; Figs. 5 and 6)." Manning (2011)

Aquifer	Broader Aquifer System	References* presenting evidence of fossil groundwater (quotes or reference to figures or tables that suggest fossil water is present)
Ocala Uplift	Floridan Aquifer System	See Table 7 OR "The ^{14}C ages of these waters increase rapidly with distance of flow, with most ages of 15–30 ka (Fig. 11)" Plummer and Sprinkle (2001)
Williston Basin	Northern Great Plains	See Table S4 McMahon et al. (2021)
Santa Clara-Calleguas Basin	-	+23,400 years old groundwater| See Figure 6-7 by Izbicki (1996)
Darton's Dakota Aquifer	-	"New data for groundwater from the Dakota aquifer of the central Great Plains region indicates that apparent ^{14}C ages range from ~1800 to >40,000 years at depths from ~ 15 to 300 meters below the surface." Davisson et al. (1995)
Northern High Plains	High Plains	Up to 19,000-year-old water | See Table 8 McMahon et al. (2004)
Confined Claiborne Near Jackson	Mississippi Embayment	552,000-year-old water | See Table 4.1 Haile 2011 | See Table 1 Davis et al. (1998)
Paskapoo-Scollard	-	low carbon-14 on See Table 2 | C, H, O and Sr isotope compositions in groundwater (See Table 2) Huff et al. (2012).
Central High Plains	High Plains	up to 12,800-year-old water | See Table 8 McMahon et al. (2004).
Sacramento Basin	California Central Valley	up to 16,000-year-old water | See Table 1 Criss and Davisson (1996).
Milk River	-	up to 2-million-year-old water | METHOD: 36Cl/Cl measurements. See Pétré et al. (2016)
Southern High Plains	High Plains	up to 26,000-year-old water | See Table 10 Dutton (1993).
Palouse Basin	Columbia Plateau Regional Aquifer System	up to 31,000-year-old water | See Table 1 Douglas et al. (2007)
Mojave Basin	-	up to 43,000-years-old groundwater See Table 1 by Kulongoski et al. (2009)
Black Warrior River Aquifer System (Eutaw and McShan Formations and Tuscaloosa Group)	-	see Table 7 of Solder (2020)
Vidalia Upland	Floridan Aquifer System	see Table 7 of Solder (2020)
Upper Coastal Plain	Floridan Aquifer System	see Table 7 of Solder (2020)
Southern Hills	Gulf Coast Regional Aquifer System	see Table 7 of Solder (2020)
Lafayette Area	Gulf Coast Regional Aquifer System	see Table 7 of Solder (2020)
Catahoula Area	Gulf Coast Regional Aquifer System	see Table 7 of Solder (2020)
Rio Grande Delta	Gulf Coast Regional Aquifer System	see Table 7 of Solder (2020)

Aquifer	Broader Aquifer System	References* presenting evidence of fossil groundwater (quotes or reference to figures or tables that suggest fossil water is present)
Peedee and Black Creek and Cape Fear Aquifers	-	see Table 7 of Solder (2020)
Victoria Area	Gulf Coast Regional Aquifer System	see Table 7 of Solder (2020)
Dougherty Plain and Marianna Lowlands	Floridan Aquifer System	see Table 7 of Solder (2020)
Western Carrizo-Wilcox	Carrizo-Wilcox	see Table 7 of Solder (2020)
Central Carrizo-Wilcox	Carrizo-Wilcox	see Table 7 of Solder (2020)
Central Mississippi Embayment	Mississippi Embayment	see Table 7 of Solder (2020)
Pearl and Chattahoochee Aquifer System	-	see Table 7 of Solder (2020)
Eastern Mississippi Embayment	Mississippi Embayment	see Table 7 of Solder (2020)
Lower Coastal Plain	Floridan Aquifer System	see Table 7 of Solder (2020)
Houston-Galveston Area	Gulf Coast Regional Aquifer System	see Table 7 of Solder (2020)
Western Mississippi Embayment	Mississippi Embayment	see Table 7 of Solder (2020)
Eastern Carrizo-Wilcox	Carrizo-Wilcox	see Table 7 of Solder (2020)

* references within Supplementary Table 1 include:

Criss, R. E., Davisson, M. L. (1996). Isotopic imaging of surface water/groundwater interactions, Sacramento Valley, California. *Journal of Hydrology,* **178,** 205-222.

Davis, S. N., Cecil, D., Zreda, M., Sharma, P. (1998). Chlorine-36 and the initial value problem. *Hydrogeology Journal,* **6,** 104-114.

Davisson, M. L., Smith, D. K., Hudson, G. B., Niemeyer, S., Macfarlane, P. A., Whittemore, D. O. (1995). Compilation of the Dakota Aquifer Project isotope data and publications: The Isotope Hydrology Program of the Isotope Sciences Division. Accessed January 14, 2022 from https://digital.library.unt.edu/ark:/67531/metadc624985/

Douglas, A. A., Osiensky, J. L., Keller, C. K. (2007). Carbon-14 dating of ground water in the Palouse Basin of the Columbia River basalts. *Journal of Hydrology,* **334,** 502-512.

Dutton, A. R. (1993). Sources and Ages of Ground Water in Unconfined and Confined Aquifers Beneath the US High Plains: Final Technical Report. Bureau of Economic Geology, the University of Texas at Austin. Accessed January 14, 2022 from https://www.beg.utexas.edu/files/publications/contract-reports/CR1993-DuttonA-1.pdf

Fryar, A. E., Mullican, W. F., Macko, S. A. (2001). Groundwater recharge and chemical evolution in the southern High Plains of Texas, USA. *Hydrogeology Journal,* **9,** 522-542.

Gardner, P. M., Heilweil, V. M. (2014). A multiple-tracer approach to understanding regional groundwater flow in the Snake Valley area of the eastern Great Basin, USA. *Applied Geochemistry,* **45,** 33-49.

Hagedorn, B., Clarke, N., Ruane, M., Faulkner, K. (2018). Assessing aquifer vulnerability from lumped parameter modeling of modern water proportions in groundwater mixtures: Application to California's South Coast Range. *Science of the Total Environment,* **624,** 1550-1560.

Haile, E. (2011). Chemical evolution and residence time of groundwater in the Wilcox aquifer of the northern Gulf Coastal Plain. PhD Thesis, University of Kentucky.

Huff, G. F., Woods, L., Moktan, H., Jean, G. (2012). Geochemistry of groundwater and spring water in the Paskapoo formation and overlying glacial drift, south-central Alberta. ERCB/Alberta Geological Survey Open-File Report, 2005. Accessed January 14, 2022 from https://ags.aer.ca/publication/ofr-2012-05

Kulongoski, J. T., Hilton, D. R., Izbicki, J. A., Belitz, K. (2009). Evidence for prolonged El Nino-like conditions in the Pacific during the Late Pleistocene: a 43 ka noble gas record from California groundwaters. *Quaternary Science Reviews,* **28,** 2465-2473.

Manning, A. H. (2011). Mountain-block recharge, present and past, in the eastern Española Basin, New Mexico, USA. *Hydrogeology Journal,* **19,** 379-397.

McIntosh, J. C., Garven, G., Hanor, J. S. (2011). Impacts of Pleistocene glaciation on large-scale groundwater flow and salinity in the Michigan Basin. *Geofluids,* **11,** 18-33.

McMahon, P. B., Böhlke, J. K., Christenson, S. C. (2004). Geochemistry, radiocarbon ages, and paleorecharge conditions along a transect in the central High Plains aquifer, southwestern Kansas, USA. *Applied Geochemistry,* **19,** 1655-1686.

McMahon, P. B., Galloway, J. M., Hunt, A. G., Belitz, K., Jurgens, B. C., Johnson, T. D. (2021). Geochemistry and age of groundwater in the Williston Basin, USA: Assessing potential effects of shale-oil production on groundwater quality. *Applied Geochemistry,* **125,** 104833.

McMahon, P. B., Plummer, L. N., Böhlke, J. K., Shapiro, S. D., Hinkle, S. R. (2011). A comparison of recharge rates in aquifers of the United States based on groundwater-age data. *Hydrogeology Journal,* **19,** 779.

McMahon, P.B., Landon, M.K., Davis, T.A., Wright, M.T., Rosecrans, C.Z., Anders, R., Land, M.T., Kulongoski, J.T., Hunt, A.G. (2021). Relative risk of groundwater-quality degradation near California (usa) oil fields estimated from ^{3}H, ^{14}C, and ^{4}He. Applied Geochemistry, 105024.

Pétré, M. A., Rivera, A., Lefebvre, R., Hendry, M. J., Folnagy, A. J. (2016). A unified hydrogeological conceptual model of the Milk River transboundary aquifer, traversing Alberta (Canada) and Montana (USA). *Hydrogeology Journal,* **24,** 1847-1871.

Plummer, L. N., Bexfield, L. M., Anderholm, S. K., Sanford, W. E., Busenberg, E. (2004). Hydrochemical tracers in the middle Rio Grande Basin, USA: 1. Conceptualization of groundwater flow. *Hydrogeology Journal,* **12,** 359-388.

Plummer, L. N., Eggleston, J. R., Andreasen, D. C., Raffensperger, J. P., Hunt, A. G., Casile, G. C. (2012). Old groundwater in parts of the upper Patapsco aquifer, Atlantic Coastal Plain, Maryland, USA: evidence from radiocarbon, chlorine-36 and helium-4. *Hydrogeology Journal,* **20,** 1269-1294.

Plummer, N. L., Sprinkle, C. L. (2001). Radiocarbon dating of dissolved inorganic carbon in groundwater from confined parts of the Upper Floridan aquifer, Florida, USA. *Hydrogeology Journal,* **9,** 127-150.

Purdy, C. B., Burr, G. S., Rubin, M., Helz, G. R., Mignerey, A. C. (1992). Dissolved organic and inorganic ^{14}C concentrations and ages for coastal plain aquifers in southern Maryland. *Radiocarbon,* **34,** 654-663.

S.S. Papadopulos & Associates, Inc., (2006). Coalbed Methane Stream Depletion Assessment Study – Northern San Juan Basin, Colorado, prepared in conjunction with the CO Geological Survey, for the State of CO Dept. of Natural Resources and the CO Oil and Gas Conservation Commission. Accessed January 14, 2022 via https://cogcc.state.co.us/documents/library/AreaReports/SanJuanBasin/CMSDA_Study.pdf

Solder, J. E. (2020). Groundwater age and susceptibility of south Atlantic and Gulf Coast principal aquifers of the contiguous United States. US Geological Survey Report No. 2020-5050. Accessed January 14, 2022 from https://pubs.er.usgs.gov/publication/sir20205050

Solder, J. E., Jurgens, B. C. (2020). Evaluation of soil zone processes and a novel radiocarbon correction approach for groundwater with mixed sources. *Journal of Hydrology,* **588,** 124766.

Izbicki, J.A. (1996). Source, Movement, and Age of Ground Water in a Coastal California Aquifer. US Geological Survey Fact Sheet 126-96, 4 pp., Accessed January 14, 2022 from: https://pubs.usgs.gov/fs/1996/0126/report.pdf

Wahi, A. K., Hogan, J. F., Ekwurzel, B., Baillie, M. N., Eastoe, C. J. (2008). Geochemical quantification of semiarid mountain recharge. *Groundwater,* **46,** 414-425.

Yokochi, R., Zappala, J. C., Purtschert, R., Mueller, P. (2021). Origin of water masses in Floridan Aquifer System revealed by ^{81}Kr. *Earth and Planetary Science Letters,* **569,** 117060.

Supplementary Note 1.2 – Radiocarbon evidence for fossil groundwater in thousands of US wells

Supplementary Fig. 1 presents the prevalence of detectable fossil well waters from ref. [1], identifying the frequency with which wells draw >0% fossil water. The proportion of well water samples made up of fossil groundwater was determined on the basis of carbon isotope compositions (for full method, see ref. [1]). In brief, the fraction of fossil water in a well water sample (F_{Fossil}) was determined following:

$$\mathrm{F}_{Fossil} = \frac{{}^{14}\mathrm{C}_{Sample} - {}^{14}\mathrm{C}_{Fossil}}{{}^{14}\mathrm{C}_{Holocene} - {}^{14}\mathrm{C}_{Fossil}}$$

where ${}^{14}C_{Sample}$ is the measured dissolved inorganic radiocarbon of a well water sample, ${}^{14}C_{Fossil}$ is the calculated range of ^{14}C activities for precipitation that infiltrated more than 11,700 years ago (i.e., before the Holocene), and ${}^{14}C_{Holocene}$ is the calculated range of ^{14}C activities for precipitation that infiltrated within the past 11,700 years. Both ${}^{14}C_{Fossil}$ and ${}^{14}C_{Holocene}$ have large ranges; we calculated a value for the minimum F_{Fossil} determined from the range of ${}^{14}C_{Fossil}$ and ${}^{14}C_{Holocene}$ values.

Ref. 1 also considered the potential for dissolution of carbonate bearing zero radiocarbon (e.g., dissolution of Paleozoic limestone) using stable carbon isotope compositions (for details see equations 1-3 in ref. [1]). There are other potential sources of inorganic C that are not explicitly unaccounted for such as endogenic CO_2 (e.g., Noseck, U., Rozanski, K., Dulinski, M., Havlová, V., Sracek, O., Brasser, T., Hercik, M., Buckau, G (2009). Carbon chemistry and groundwater dynamics at natural analogue site Ruprechtov, Czech Republic: Insights from environmental isotopes. Applied Geochemistry, 24, 1765–1776); however, even where endogenic carbon contributions to DIC may be high, their presence has been suggested to have little influence on F_{fossil} estimations (Wang, T., Chen, J., Zhang, C. (2021). Estimation of fossil groundwater mass fraction accounting for endogenic carbon input across California. Journal of Hydrology, 595, 126034). For further details on the method applied to identify fossil well waters see ref. [1]. The fraction of all wells with ^{14}C data (reported in ref. [1]) where the minimal estimated F_{Fossil} exceeds zero are displayed in Supplementary Fig. 1 (as a function of total well depth on the y-axis).

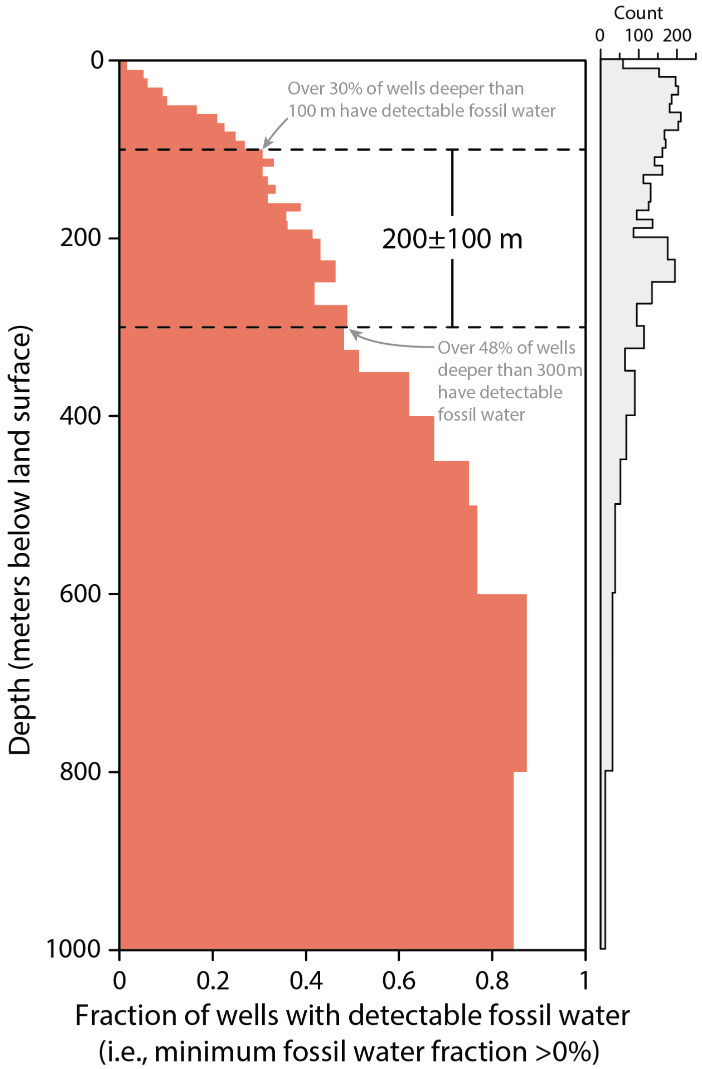

Supplementary Fig. 1. Documented occurrence of fossil groundwater in US wells. Fossil groundwater prevalence based on well depths. Red areas depict the fraction of well water samples that contain some (i.e., more than 0%) fossil groundwater within a given well depth band; these data are based on analyses of radiocarbon (results derive from Jasechko et al., 2017). Fossil groundwater tends to be more common in deeper wells, and occurs frequently in wells exceeding 200±100 m (white horizontal lines show the range: 200±100 m). Specifically, more than 30% of wells with depths at or exceeding 100 m contain detectable fossil water, and more than ~half (48%) of wells with depths at or exceeding 300 m contain detectable fossil water (defined as minimum 'fraction of well water sample comprised of fossil groundwater' values that exceed zero, determined on the basis of carbon isotope compositions; see ref. [1]). We stress that the lack of well screen interval data limits our interpretation of this figure; specifically, samples are categorized in this figure based on the total depth of the well from which they derive (rather than the screen interval of these wells, as such perforation data are unavailable for many of the wells in the dataset analyzed by ref. [1]).

Supplementary Note 2 – Supplementary Results

Here we present supplementary figures to support the methods and results described in the main text.

Supplementary Note 2.1 – Temporal variations in constructed well depths relative to a nearby well that contains fossil water

We completed tested the research question: "*is the proportion of newly drilled wells in the US that tap fossil aquifers increasing over time?*".

The following schematic figure (Supplementary Fig. 2) is provided to help convey the steps we describe in the main text (Fig. 3).

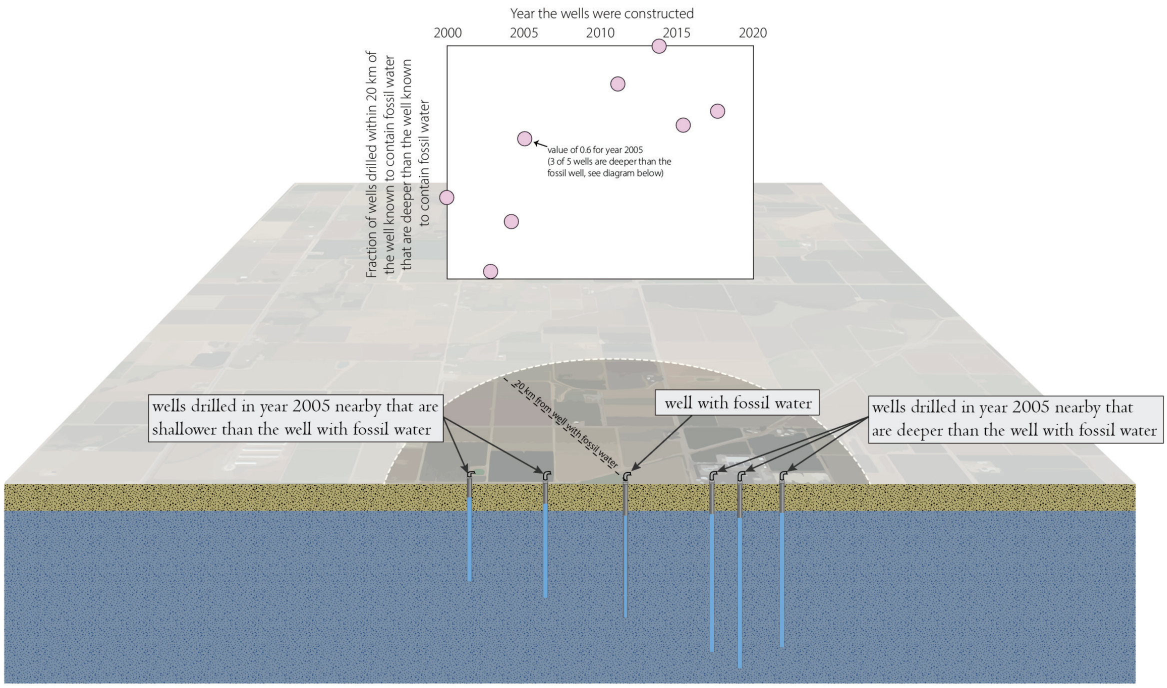

Supplementary Fig. 2. Schematic diagram showing the fraction of wells drilled within 20 km of the fossil water – shaded half circle – and the surrounding drilled borehole. The temporal variation of the fraction of well deeper than the fossil well is presented by considering the relative borehole drilling depth with respect to the fossil well.

Supplementary Note 2.2 – Temporal variations in the proportion of wells deeper than 200±100m within the boundaries of aquifer systems

To further explore fossil groundwater access over time, we analyzed changes in the fraction of newly constructed wells with depths exceeding 200±100 m over five different time intervals: 1950-1975, 1975-2000, 2000-2015, 1950-2015, and 1975-2015.

We evaluated temporal variations in the proportion of newly constructed wells that likely tap fossil aquifers following several steps. First, we identified all groundwater well completion records that present both a completion date (e.g., well completed on January 3, 1982) and a depth (e.g., 34 meters below ground). Next, we completed a spatial join to identify all wells within a single aquifer system (as defined in our US Aquifer Database). Then, for any calendar year within which at least 5 well completion records exist, we calculated the [proportion of all newly constructed wells that have a depth exceeding a 'threshold depth'] (where 'threshold depth' is 100 m, 200 m or 300 m, a set of threshold depths that encompass the broad range at which many aquifer systems transition from young water (shallow) to fossil water (deep)). Last, we completed non-parametric regressions of the rank transforms of [well completion year] versus the rank transforms of [the proportion of all newly constructed wells that have a depth exceeding a 'threshold depth'].

We only consider cases where all of the following criteria are met: (a) at least one calendar year met our criteria for analysis (i.e., at least five drilled wells) in the first five years of a studied time interval (e.g., for analyses of the time interval 1950-1975, we require at least one of the following five calendar years to meet our criteria for analysis: 1950, 1951, 1952, 1953 or 1954), (b) at least one calendar year met our criteria for analysis (i.e., at least five drilled wells) in the final five years of a studied time interval; and, (c) a minimum of at least five calendar years within the time interval met our criteria for analyses. The correlations were determined for five distinct time intervals: (i) 1950-1975, (ii) 1975-2000, (iii) 2000-2015, (iv) 1950-2015, and (v) 1975-2015. Positive Spearman rank correlation coefficients (ρ) imply that the proportion of drilled wells that are deeper than 100 m, 200 m, or 300 m (many of which are also likely pump fossil water) has increased over time (Supplementary Table 2 and Supplementary Fig. 3). Negative Spearman rank correlation coefficients imply that the proportion of drilled wells that are deeper than 100 m, 200 m, or 300 m has declined over time (Supplementary Fig. 3). Results for each time interval (i.e., the prevalence of blue versus red diamonds in Supplementary Fig. 3) are summarized in Supplementary Fig. 4.

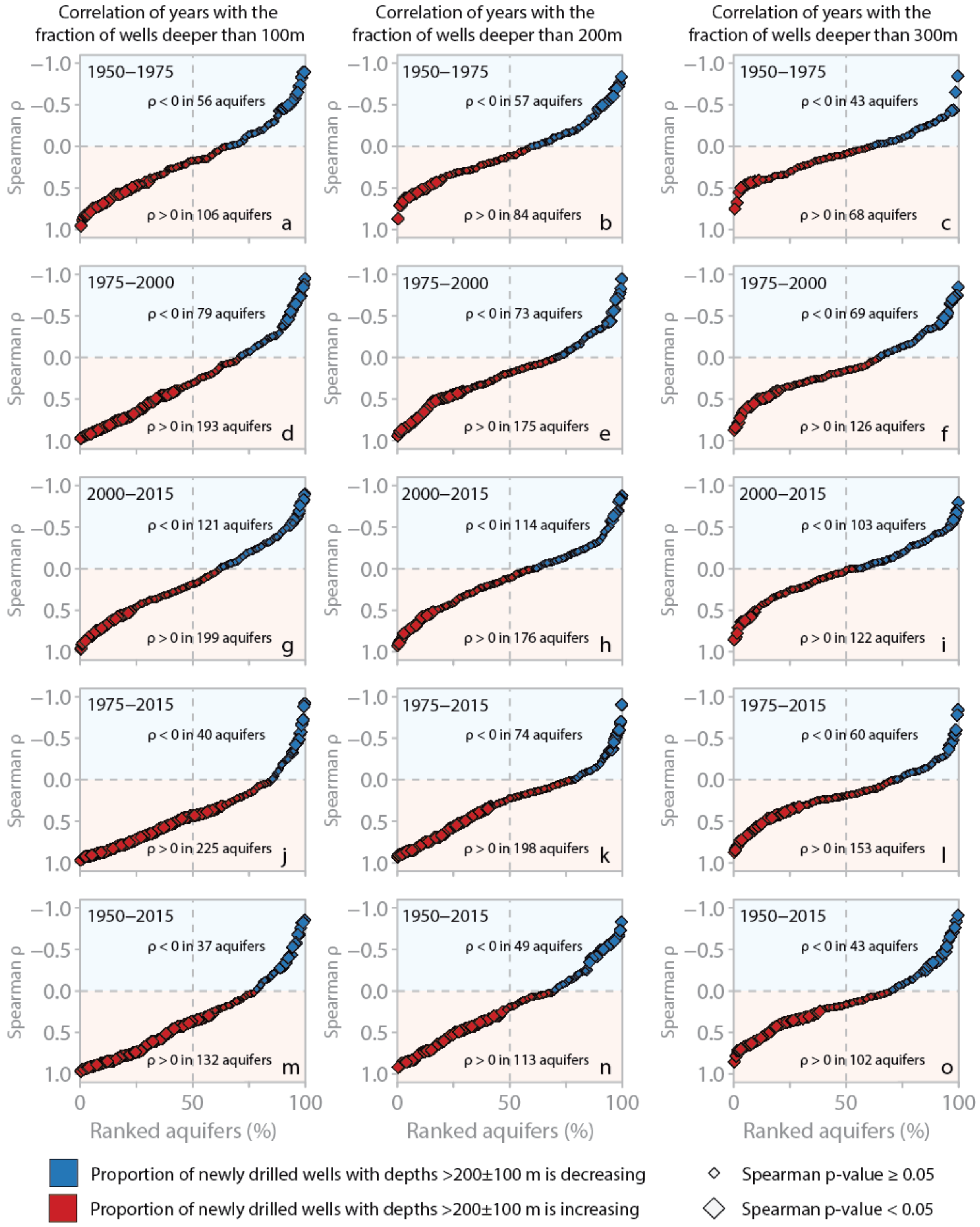

Correlation of years with the fraction of wells deeper than 100m
Correlation of years with the fraction of wells deeper than 200m
Correlation of years with the fraction of wells deeper than 300m
1950–1975
ρ < 0 in 56 aquifers
ρ > 0 in 106 aquifers
a
ρ < 0 in 57 aquifers
ρ > 0 in 84 aquifers
b
ρ < 0 in 43 aquifers
ρ > 0 in 68 aquifers
c
1975–2000
ρ < 0 in 79 aquifers
ρ > 0 in 193 aquifers
d
ρ < 0 in 73 aquifers
ρ > 0 in 175 aquifers
e
ρ < 0 in 69 aquifers
ρ > 0 in 126 aquifers
f
2000–2015
ρ < 0 in 121 aquifers
ρ > 0 in 199 aquifers
g
ρ < 0 in 114 aquifers
ρ > 0 in 176 aquifers
h
ρ < 0 in 103 aquifers
ρ > 0 in 122 aquifers
i
1975–2015
ρ < 0 in 40 aquifers
ρ > 0 in 225 aquifers
j
ρ < 0 in 74 aquifers
ρ > 0 in 198 aquifers
k
ρ < 0 in 60 aquifers
ρ > 0 in 153 aquifers
l
1950–2015
ρ < 0 in 37 aquifers
ρ > 0 in 132 aquifers
m
ρ < 0 in 49 aquifers
ρ > 0 in 113 aquifers
n
ρ < 0 in 43 aquifers
ρ > 0 in 102 aquifers
o
Spearman ρ
−1.0
−0.5
0.0
0.5
1.0
0
50
100
Ranked aquifers (%)
Proportion of newly drilled wells with depths >200±100 m is decreasing
Proportion of newly drilled wells with depths >200±100 m is increasing
Spearman p-value ≥ 0.05
Spearman p-value < 0.05

Supplementary Fig. 3 (previous page). Rank correlation of well completion year versus the fraction of all drilled wells that exceed 200±100 m. Each panel presents results for a single time interval (time interval stated in the upper left corner of each plot) using one threshold depth (100 m, 200 m, or 300 m; see labels at top of figure). Each red or blue diamond represents a single unique aquifer system. Each plot presents rank correlation coefficients expressing how the fraction of all wells constructed in a given year that are deeper than 100 m, 200 m), or 300 m has varied over time. Each row of plots corresponds to a distinct time interval (e.g., panels a-c in the top row present correlation coefficients for the time interval of 1950-1975); the studied time interval is specified in the top-left corner of each plot. Each column of plots corresponds to a distinct threshold well depth (e.g., the leftmost column expresses the proportion of wells drilled in a given year that are deeper than 100 m). Red diamonds suggest that the proportion of wells deeper than a threshold depth (e.g., deeper than 100 m (plots in leftmost column), 200 m (plots in middle column), or 300 m (plots in rightmost column)) increased over the studied time interval (i.e., Spearman ρ values exceed zero). Conversely, blue diamonds represent areas where the proportion of wells deeper than a threshold depth (e.g., deeper than 100 m (plots in leftmost column), 200 m (plots in middle column), or 300 m (plots in rightmost column)) decreased over the studied time interval (i.e., Spearman ρ values of less than zero). Larger-sized diamond symbols represent significant correlations (Spearman p-value < 0.05); smaller diamonds represent non-significant correlations (Spearman p-value ≥ 0.05).

Supplementary Table 2. Temporal variations in the frequency with which wells have been drilled deeper than 100 m, 200 m, and 300 m among

**Time interval**	**Prevalence of wells deeper than 100 meters**		**Prevalence of wells deeper than 200 meters**		**Prevalence of wells deeper than 300 meters**	
	n_{all}= total number of aquifers (any Spearman P-value)<br><br>n_{deep} = aquifers with increasing fraction of well deeper than 100 m (Spearman ρ > 0)<br><br>$n_{undefined}$ = aquifers with undefined Spearman ρ	n_{sig} = aquifers with significant correlation only (Spearman P-value < 0.05)<br><br>$n_{deep\text{-}sig}$ = aquifers with significant, increasing fraction of well deeper than 100 m (Spearman P-value < 0.05 and Spearman ρ > 0)	n_{all}= total number of aquifers (any Spearman P-value)<br><br>n_{deep} = aquifers with increasing fraction of well deeper than 200 m (Spearman ρ > 0)<br><br>$n_{undefined}$ = aquifers with undefined	n_{sig} = aquifers with significant correlation only (Spearman P-value < 0.05)<br><br>$n_{deep\text{-}sig}$ = aquifers with significant, increasing fraction of well deeper than 200 m (Spearman P-value < 0.05 and Spearman ρ > 0)	n_{all}= total number of aquifers (any Spearman P-value)<br><br>n_{deep} = aquifers with increasing fraction of well deeper than 300 m (Spearman ρ > 0)<br><br>$n_{undefined}$ = aquifers with undefined Spearman ρ	n_{sig} = aquifers with significant correlation only (Spearman P-value < 0.05)<br><br>$n_{deep\text{-}sig}$ = aquifers with significant, increasing fraction of well deeper than 300 m (Spearman P-value < 0.05 and Spearman ρ > 0)
1950-1975	n_{all}=166<br>n_{deep}=106 (64%)<br>$n_{shallow}$=56 (34%)<br>$n_{undefined}$=3 (2%)	$n_{deep\text{-}sig}$=45 of n_{sig}=64 (70%)	n_{all}=166<br>n_{deep}=84 (51%)<br>$n_{shallow}$=57 (34%)<br>$n_{undefined}$=25 (15%)	$n_{deep\text{-}sig}$=25 of n_{sig}=41 (61%)	n_{all}=166<br>n_{deep}=68 (41%)<br>$n_{shallow}$=43 (26%)<br>$n_{undefined}$=54 (33%)	$n_{deep\text{-}sig}$=12 of n_{sig}=16 (75%)
1975-2000	n_{all}=278<br>n_{deep}=193 (69%)<br>$n_{shallow}$=79 (28%)<br>$n_{undefined}$=6 (2%)	$n_{deep\text{-}sig}$=116 of n_{sig}=140 (83%)	n_{all}=278<br>n_{deep}=175 (63%)<br>$n_{shallow}$=73 (26%)<br>$n_{undefined}$=30 (11%)	$n_{deep\text{-}sig}$=69 of n_{sig}=86 (80%)	n_{all}=278<br>n_{deep}=126 (45%)<br>$n_{shallow}$=69 (25%)<br>$n_{undefined}$=82 (29%)	$n_{deep\text{-}sig}$=38 of n_{sig}=53 (72%)
2000-2015	n_{all}=326<br>n_{deep}=199 (61%)<br>$n_{shallow}$=121 (37%)<br>$n_{undefined}$=5 (2%)	$n_{deep\text{-}sig}$=70 of n_{sig}=91 (77%)	n_{all}=326<br>n_{deep}=176 (54%)<br>$n_{shallow}$=114 (35%)<br>$n_{undefined}$=34 (10%)	$n_{deep\text{-}sig}$=46 of n_{sig}=61 (75%)	n_{all}=326<br>n_{deep}=122 (37%)<br>$n_{shallow}$=103 (32%)<br>$n_{undefined}$=95 (29%)	$n_{deep\text{-}sig}$=22 of n_{sig}=30 (73%)
1950-2015	n_{all}=171<br>n_{deep}=132 (77%)<br>$n_{shallow}$=37 (22%)<br>$n_{undefined}$=2 (1%)	$n_{deep\text{-}sig}$=97 of n_{sig}=115 (84%)	n_{all}=171<br>n_{deep}=113 (66%)<br>$n_{shallow}$=49 (29%)<br>$n_{undefined}$=9 (5%)	$n_{deep\text{-}sig}$=74 of n_{sig}=99 (75%)	n_{all}=171<br>n_{deep}=102 (60%)<br>$n_{shallow}$=43 (25%)<br>$n_{undefined}$=26 (15%)	$n_{deep\text{-}sig}$=54 of n_{sig}=76 (71%)
1975-2015	n_{all}=269<br>n_{deep}=225 (84%)<br>$n_{shallow}$=40 (15%)<br>$n_{undefined}$=4 (1%)	$n_{deep\text{-}sig}$=163 of n_{sig}=175 (93%)	n_{all}=269<br>n_{deep}=198 (79%)<br>$n_{shallow}$=74 (20%)<br>$n_{undefined}$=17 (6%)	$n_{deep\text{-}sig}$=104 of n_{sig}=118 (88%)	n_{all}=269<br>n_{deep}=153 (57%)<br>$n_{shallow}$=60 (22%)<br>$n_{undefined}$=55 (20%)	$n_{deep\text{-}sig}$=60 of n_{sig}=69 (87%)

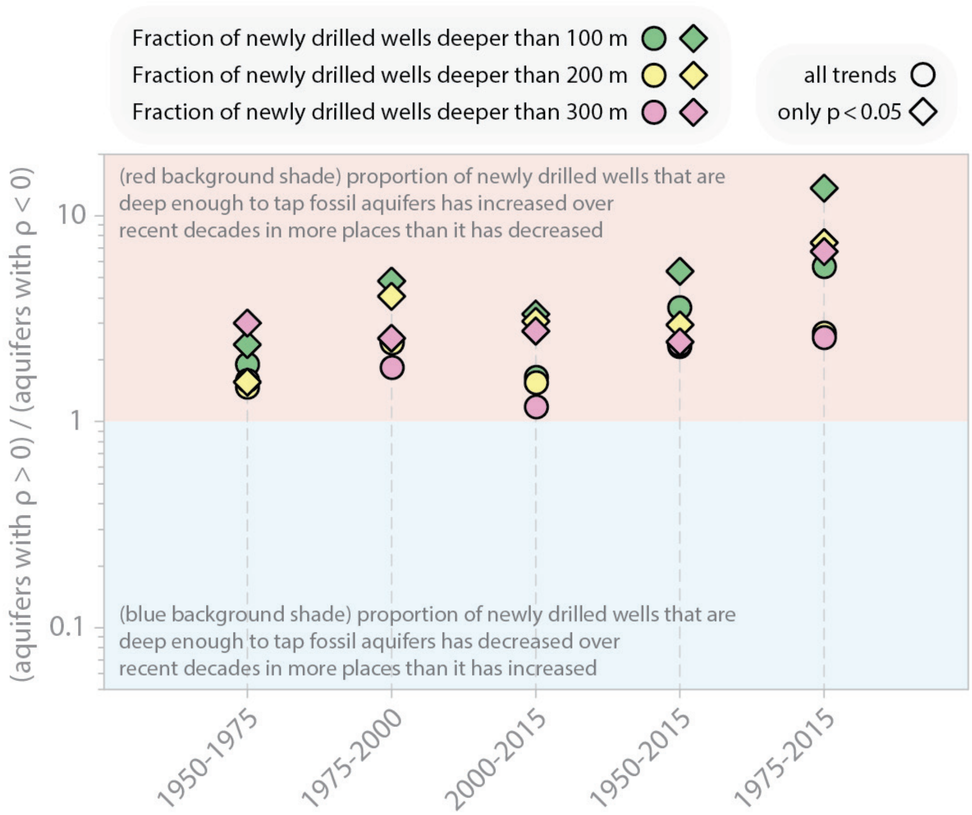

Supplementary Fig. 4. Variability in the proportion of wells deep enough to tap fossil water over time; points plotted in the red background indicate that the proportion of newly drilled wells that are deep enough to tap fossil aquifers has increased over recent decades in more places than it has decreased. The y-axis presents [the number of aquifers where the proportion of newly drilled wells that are deeper than 200±100 m is increasing over time (i.e., number of aquifers with $\rho > 0$)] divided by [the number of aquifers where the proportion of newly drilled wells that are deeper than 200±100 m is decreasing over time (i.e., number of aquifers with $\rho < 0$)]. The rank correlation coefficients for each aquifer were determined on the basis of statistical relationships between [the proportion of newly drilled wells in a given year that are deeper than a given threshold depth (Spearman rank correlation coefficients ($\rho > 0$)] and [well completion year]. Each point represents a unique combination of a studied time interval over which our correlation coefficients were determined and a given threshold depth (i.e., threshold depths are: 100 m, 200 m, and 300 m). Green points reflect temporal variability in the proportion of wells deeper than 100 m, yellow points reflect temporal variability in the proportion of wells deeper than 200 m, and pink points reflect temporal variability in the proportion of wells deeper than 300 m. Circles depict all results (i.e., any Spearman p-value); diamonds depict only significant correlations (i.e., only those correlations with a Spearman p-value of less than 0.05).

Supplementary Note 2.3 – Statistical relationships between the proportion of wells deeper than 200±100m within the boundaries of aquifer systems and groundwater level variations over time

We analyzed groundwater level time series from monitoring wells within each of our student aquifers. We calculated—for five different time intervals: 1950-1975, 1975-2000, 2000-2015, 1950-2015 and 1975-2015)—the Theil-Sen slope describing the rate of change in groundwater levels in each monitoring well. Next, we calculated the median groundwater level slope (in meters per year) for each aquifer system (see figure below).

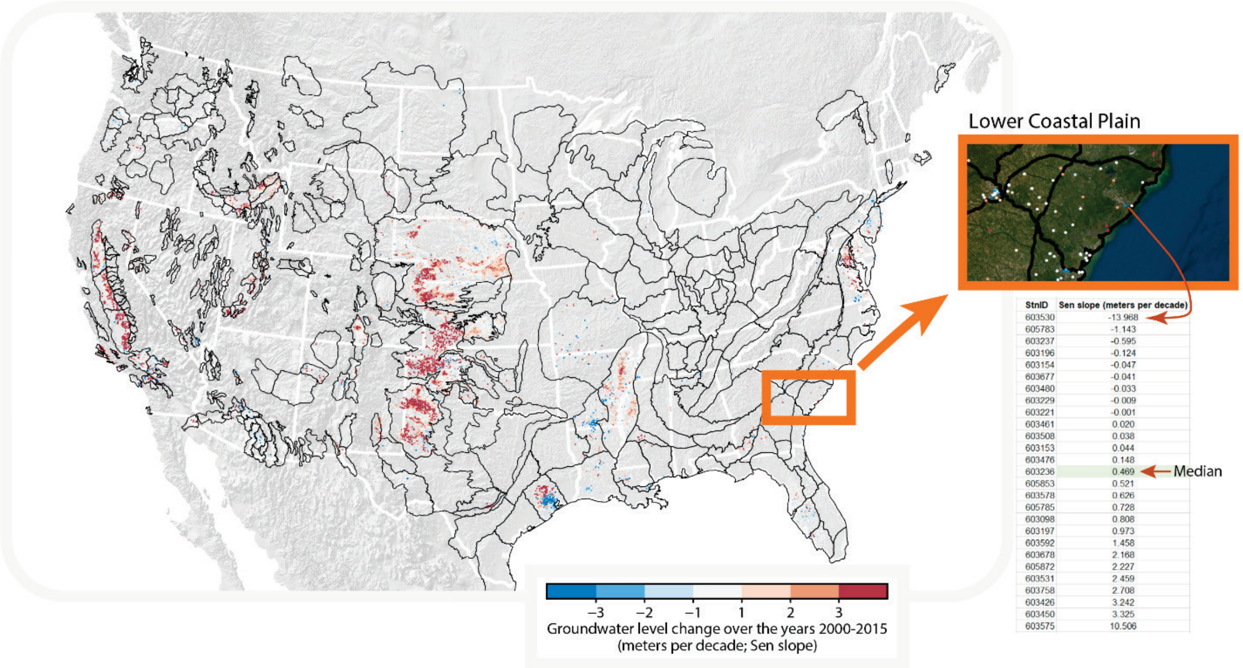

Supplementary Fig. 5. A schematic diagram showing the analysis of the rate of change of groundwater level over time for a given time period (e.g. 2000-2015). The diagram shows the calculated groundwater level change based on the Theil-Sen slope (m/decade). The red and blue point data set shows unique monitoring well locations that show groundwater level deepening over time (red dots) or shallowing over time (blue dots) respectively.

Supplementary Table 3. Prevalence of aquifers with declining groundwater levels

**Time span**	**Aquifers with a median** *(as determined from all monitoring wells with sufficient data within the aquifer system's boundaries)* **Theil-Sen slope > 0 (expressed in percent or the total number of study aquifers)**	**Aquifer with median Theil-Sen slope > 0**	**Total number of study aquifers**
1950-1975	67% *(181 divided by 269 in percent)*	181	269
1975-2015	67% *(245 divided by 364 in percent)*	245	364
2000-2015	71% *(204 divided by 288 in percent)*	204	288
1950-2015	70% *(258 divided by 371 in percent)*	258	371
1975-2000	57% *(194 divided by 339 in percent)*	194	339

Supplementary Note 3 – Delineation of aquifers across the United States

Here we present a new geospatial dataset of aquifer systems across the USA. The delineated aquifers derive from a review of local- and regional-scale hydrogeologic conditions (Supplementary Note 3.1). We were motivated to delineate US aquifer boundaries rather than analyze the USGS' Principal Aquifer geodatabase for numerous reasons (Supplementary Note 3.2).

Supplementary Note 3.1 – Primary sources and approach to delineate hydrogeologic study areas

Here we present a detailed overview of the approach we took to develop this new geodatabase of study areas (i.e., physiographic regions and aquifer systems; Supplementary Table 4).

Supplementary Table 4. Steps taken and references consulted to delineate aquifers across the United States

Aquifer	Reference 1	Reference 2	Reference 3	Steps taken to delineate
Central Allegheny Plateau, Appalachian Plateaus	Trapp Jr., H., Horn, M.A. (1997). Ground water atlas of the United States: Segment 11. U.S. Geological Survey Hydrologic Investigations Atlas 730-L, 26 pp. Accessed April 13, 2021 from https://pubs.usgs.gov/ha/730l/report.pdf	-	-	Broader Appalachian Plateau Aquifer System approximated from Fig. 3 of Trapp Jr. and Horn (1997)
Central Cumberland Plateau and Sequatchie Valley, Appalachian Plateaus	Brahana, J.V., Macy, J.A., Mulderink, D., Zemo, D. (1986). Preliminary delineation and description of the regional aquifers of Tennessee-Cumberland plateau aquifer system. U.S. Geological Survey, Water-Resources Investigations Report 82-338, 29 pp. Accessed April 13, 2021 from https://pubs.usgs.gov/wri/wrir82-338/pdf/wrir_82-338_a.pdf	-	-	Approximated from Fig. 2 by Brahana et al. (1986)
Northern Allegheny Plateau, Appalachian Plateaus	Trapp Jr., H., Horn, M.A. (1997). Ground water atlas of the United States: Segment 11. U.S. Geological Survey Hydrologic Investigations Atlas 730-L, 26 pp. Accessed April 13, 2021 from https://pubs.usgs.gov/ha/730l/report.pdf	-	-	Broader Appalachian Plateau Aquifer System approximated from Fig. 3 of Trapp Jr. and Horn (1997)
Northern Cumberland Plateau and Mountains, Appalachian Plateaus	Brahana, J.V., Macy, J.A., Mulderink, D., Zemo, D. (1986). Preliminary delineation and description of the regional aquifers of Tennessee-Cumberland plateau aquifer system. U.S. Geological Survey, Water-Resources Investigations Report 82-338, 29 pp. Accessed April 13, 2021 from https://pubs.usgs.gov/wri/wrir82-338/pdf/wrir_82-338_a.pdf	Trapp Jr., H., Horn, M.A. (1997). Ground water atlas of the United States: Segment 11. U.S. Geological Survey Hydrologic Investigations Atlas 730-L, 26 pp. Accessed April 13, 2021 from https://pubs.usgs.gov/ha/730l/report.pdf	-	Approximated from Fig. 2 by Brahana et al. (1986). Broader Appalachian Plateau Aquifer System approximated from Fig. 3 of Trapp Jr. and Horn (1997)
Southeastern Allegheny Plateau, Appalachian Plateaus	Trapp Jr., H., Horn, M.A. (1997). Ground water atlas of the United States: Segment 11. U.S. Geological Survey Hydrologic Investigations Atlas 730-L, 26 pp. Accessed April 13, 2021 from https://pubs.usgs.gov/ha/730l/report.pdf	-	-	Broader Appalachian Plateau Aquifer System approximated from Fig. 3 of Trapp Jr. and Horn (1997)
Southern Cumberland Plateau - Pottsville Aquifer, Appalachian Plateaus	Jennings, S.P. (2013). Hydrogeology and groundwater assessment of the water distribution area of the town of Hodges water department, Franklin and Marion Counties, Alabama. Geological Survey of Alabama Report, 15pp. Accessed April 8, 2021 from https://www.ogb.state.al.us/img/Groundwater/OFR/OFR1311.pdf	Geological Survey of Alabama (2018). Assessment of groundwater resources in Alabama, 2010-16. Geological Survey of Alabama Bulletin 186, 462 pp. Accessed April 8, 2021 from https://www.gsa.state.al.us/img/Groundwater/docs/assessment/00_B186_StatewideAssessment_Print_Document.pdf	-	Physiographic provinces from Fig. 1 of Jennings (2013) and Fig. 4 of Geological Survey of Alabama (2018). Aquifer recharge areas defined in Fig. 6 of Geological Survey of Alabama (2018). Northern limit of area was approximated at Guntersville Lake.
Southwestern Allegheny Plateau, Appalachian Plateaus	Brockman, S. (1998). Physiographic regions of Ohio. Ohio Department of Natural Resources, Division of Geological Survey map. Accessed April 12, 20221 from https://www.epa.state.oh.us/portals/27/SIP/Nonattain/F2-physiographic_regions_of_Ohio.pdf	-	-	Approximated from map by Brockman (1998). Northern boundary approximated along the Hackman River.
Western Allegheny Plateau, Appalachian Plateaus	Brockman, S. (1998). Physiographic regions of Ohio. Ohio Department of Natural Resources, Division of Geological Survey map. Accessed April 12, 20221 from https://www.epa.state.oh.us/portals/27/SIP/Nonattain/F2-physiographic_regions_of_Ohio.pdf	Trapp Jr., H., Horn, M.A. (1997). Ground water atlas of the United States: Segment 11. U.S. Geological Survey Hydrologic Investigations Atlas 730-L, 26 pp. Accessed April 13, 2021 from https://pubs.usgs.gov/ha/730l/report.pdf	-	Approximated from map by Brockman (1998). Southern boundary approximated along the Hackman River. Broader Appalachian Plateau Aquifer System approximated from Fig. 3 of Trapp Jr. and Horn (1997)

Aquifer	Reference 1	Reference 2	Reference 3	Steps taken to delineate
Sacramento Basin, California Central Valley	Bertoldi, G. L., Johnston, R. H., Evenson, K. D. (1991). Ground water in the Central Valley, California: a summary report. United States Geological Survey Professional Paper 1401-A.	Mendenhall, W. C. (1908). Ground waters of the San Joaquin Valley, California. US Geological Survey Water-Supply Paper 222, 54 pp. Accessed April 6, 2021 from https://pubs.usgs.gov/wsp/0222/report.pdf	-	Approximated from Fig. 1 of Bertoldi et al. (1991); hydrologic regions (https://atlas-dwr.opendata.arcgis.com/datasets/2a572a181e094020bdaeb5203162de15_0) used to distinguish the three parts of the Central Valley. Delineation of conditions also available via plate 1 by Mendenhall (1908).
San Joaquin Basin, California Central Valley	Bertoldi, G. L., Johnston, R. H., Evenson, K. D. (1991). Ground water in the Central Valley, California: a summary report. United States Geological Survey Professional Paper 1401-A.	Mendenhall, W. C. (1908). Ground waters of the San Joaquin Valley, California. US Geological Survey Water-Supply Paper 222, 54 pp. Accessed April 6, 2021 from https://pubs.usgs.gov/wsp/0222/report.pdf	-	Approximated from Fig. 1 of Bertoldi et al. (1991); hydrologic regions (https://atlas-dwr.opendata.arcgis.com/datasets/2a572a181e094020bdaeb5203162de15_0) used to distinguish the three parts of the Central Valley. Delineation of conditions also available via plate 1 by Mendenhall (1908).
Tulare Basin, California Central Valley	Bertoldi, G. L., Johnston, R. H., Evenson, K. D. (1991). Ground water in the Central Valley, California: a summary report. United States Geological Survey Professional Paper 1401-A.	-	-	Approximated from Fig. 1 of Bertoldi et al. (1991); hydrologic regions (https://atlas-dwr.opendata.arcgis.com/datasets/2a572a181e094020bdaeb5203162de15_0) used to distinguish the three parts of the Central Valley
Central Carrizo-Wilcox, Carrizo-Wilcox	Huang, Y., Scanlon, B. R., Nicot, J. P., Reedy, R. C., Dutton, A. R., Kelley, V. A., Deeds, N. E. (2012). Sources of groundwater pumpage in a layered aquifer system in the Upper Gulf Coastal Plain, USA. Hydrogeology Journal, 20(4), 783-796.	Kelley, V.A., Deeds, N.E., Fryar, D.G., Nicot, J.P. (2004). Groundwater availability models for the Queen City and Sparta aquifers. Contract report to the Texas Water Development Board, Austin, TX, 867 pp. Accessed February 24, 2022 from https://www.twdb.texas.gov/groundwater/models/gam/qcsp/QCSP_Model_Report.pdf?d=29484	-	Approximated from Fig. 2.2 by Kelley et al. (2004). Texas portion approximated from Fig. 1 of Huang et al. (2012). Eastern and western boundaries approximated by Texas' 12th Groundwater Management Area (GMA) (accessed February 11, 2021 from https://www.twdb.texas.gov/groundwater/management_areas/gma12.asp)
Eastern Carrizo-Wilcox, Carrizo-Wilcox	Huang, Y., Scanlon, B. R., Nicot, J. P., Reedy, R. C., Dutton, A. R., Kelley, V. A., Deeds, N. E. (2012). Sources of groundwater pumpage in a layered aquifer system in the Upper Gulf Coastal Plain, USA. Hydrogeology Journal, 20(4), 783-796.	Kelley, V.A., Deeds, N.E., Fryar, D.G., Nicot, J.P. (2004). Groundwater availability models for the Queen City and Sparta aquifers. Contract report to the Texas Water Development Board, Austin, TX, 867 pp. Accessed February 24, 2022 from https://www.twdb.texas.gov/groundwater/models/gam/qcsp/QCSP_Model_Report.pdf?d=29484	Louisiana Department of Environmental Quality (2007). Carrizo-Wilcox Aquifer Summary Report 2007 Aquifer Sampling and Assessment Program (ASSET) Program. Accessed February 11, 2021 from https://deq.louisiana.gov/assets/docs/Water/Triennial_reports/AquiferSummaries_2007-2009/02Carrizo-WilcoxAquiferSummary09.pdf	Approximated from Fig. 2.2 by Kelley et al. (2004). Texas portion approximated from Fig. 1 of Huang et al. (2012). Louisiana portion approximated from Fig. 2-1 of Louisiana Department of Environmental Quality (2007). Western boundary is the western margin of the Texas Groundwater Management Area (GMA) #11 (accessed February 11, 2021 from https://www.twdb.texas.gov/groundwater/management_areas/gma12.asp)
Western Carrizo-Wilcox, Carrizo-Wilcox	Huang, Y., Scanlon, B. R., Nicot, J. P., Reedy, R. C., Dutton, A. R., Kelley, V. A., Deeds, N. E. (2012). Sources of groundwater pumpage in a layered aquifer	Kelley, V.A., Deeds, N.E., Fryar, D.G., Nicot, J.P. (2004). Groundwater availability models	-	Approximated from Fig. 2.2 by Kelley et al. (2004). Texas portion approximated from Fig. 1 of Huang et al. (2012), with the eastern

Aquifer	Reference 1	Reference 2	Reference 3	Steps taken to delineate
	system in the Upper Gulf Coastal Plain, USA. Hydrogeology Journal, 20(4), 783-796.	for the Queen City and Sparta aquifers. Contract report to the Texas Water Development Board, Austin, TX, 867 pp. Accessed February 24, 2022 from https://www.twdb.texas.gov/groundwater/models/gam/qcsp/QCSP_Model_Report.pdf?d=29484		boundary approximated by the division between Texas' 12th and 13th Groundwater Management Area (GMA; accessed February 11, 2021 from https://www.twdb.texas.gov/groundwater/management_areas/gma12.asp). The Mexico portion of the aquifer was approximated based on well depth data.
Carson Valley, Carson River Basin	Maurer, D. K. (2011). Geologic framework and hydrogeology of the middle Carson River Basin, Eagle, Dayton, and Churchill Valleys, West-Central Nevada. U. S. Geological Survey Scientific Investigations Report 2011–5055. 74 pp. Accessed March 10, 2021 from https://pubs.usgs.gov/sir/2011/5055/pdf/sir20115055.pdf	-	-	Approximated from Fig. 1 of Maurer et al. (2011).
Churchill Valley, Carson River Basin	Maurer, D. K. (2011). Geologic framework and hydrogeology of the middle Carson River Basin, Eagle, Dayton, and Churchill Valleys, West-Central Nevada. U. S. Geological Survey Scientific Investigations Report 2011–5055. 74 pp. Accessed March 10, 2021 from https://pubs.usgs.gov/sir/2011/5055/pdf/sir20115055.pdf	-	-	Approximated from Fig. 1 of Maurer et al. (2011).
Dayton Valley, Carson River Basin	Maurer, D. K. (2011). Geologic framework and hydrogeology of the middle Carson River Basin, Eagle, Dayton, and Churchill Valleys, West-Central Nevada. U. S. Geological Survey Scientific Investigations Report 2011–5055. 74 pp. Accessed March 10, 2021 from https://pubs.usgs.gov/sir/2011/5055/pdf/sir20115055.pdf	-	-	Approximated from Fig. 1 of Maurer et al. (2011).
Eagle Valley, Carson River Basin	Maurer, D. K. (2011). Geologic framework and hydrogeology of the middle Carson River Basin, Eagle, Dayton, and Churchill Valleys, West-Central Nevada. U. S. Geological Survey Scientific Investigations Report 2011–5055. 74 pp. Accessed March 10, 2021 from https://pubs.usgs.gov/sir/2011/5055/pdf/sir20115055.pdf	Maurer, D.K., Thodal, C.E. (2000). Quantity and chemical quality of recharge, and updated water budgets, for the basin-fill aquifer in Eagle Valley, western Nevada: U.S. Geological Survey Water-Resources Investigations Report 99-4289, 52 pp. Accessed November 29, 2021 from https://pubs.usgs.gov/wri/1999/4289/report.pdf	-	Approximated from Fig. 1 of Maurer et al. (2011) and Fig. 1 by Maurer and Thodal (2000)
Bellefontaine Upland and Mad River Interlobate Plain, Central Lowland Till Plain	Brockman, S. (1998). Physiographic regions of Ohio. Ohio Department of Natural Resources, Division of Geological Survey map. Accessed April 12, 20221 from https://www.epa.state.oh.us/portals/27/SIP/Nonattain/F2-physiographic_regions_of_Ohio.pdf	Fenneman, N.M., Johnson, D.W. (1946). Physiographic divisions of the conterminous United States. U.S. Geological Survey map, 1:7,000,000 Scale.	-	Broader Till Plain physiographic region approximated from Fenneman and Johnson (1946). Bellefontaine Upland and Mad River Interlobate Plain areas approximated from the map by Brockman (1998).
Bluffton Till Plain, Central Lowland Till Plain	Gray, H.H. (2001). Map of Indiana showing physiographic divisions. Indiana Geological Survey Miscellaneous Map 69, 15 pp. Accessed April 12, 2021 from https://igws.indiana.edu/ReferenceDocs/Maps/PhysiographicRegions.pdf	Brockman, S. (1998). Physiographic regions of Ohio. Ohio Department of Natural Resources, Division of Geological Survey map. Accessed April 12, 20221 from https://www.epa.state.oh.us/portals/27/SIP/Nonattain/F2-physiographic_regions_of_Ohio.pdf	Fenneman, N.M., Johnson, D.W. (1946). Physiographic divisions of the conterminous United States. U.S. Geological Survey map, 1:7,000,000 Scale.	Broader Till Plain physiographic region approximated from Fenneman and Johnson (1946). Bluffton Till Plain approximated for Indiana from map by Gray (2001) and for Ohio following the approximate border of the "Central Ohio Clayey Till Plain" defined in map by Brockman (1998).

Aquifer	Reference 1	Reference 2	Reference 3	Steps taken to delineate
Central Wabash and Bloomington Ridged Plain, Central Lowland Till Plain	Stephenson, D.A. (1967). Hydrogeology of glacial deposits of the Mahomet Bedrock Valley in east-central Illinois. Illinois State Geological Survey Circular 409, 56 pp. Accessed November 29, 2021 from https://www.ideals.illinois.edu/bitstream/handle/2142/35125/hydrogeologyofgl409step.pdf?sequence=2	Leighton, M. M., Ekblaw, G. E., Horberg, L. (1948). Physiographic divisions of Illinois. The Journal of Geology, 56, 16-33.	Gray, H.H. (2001). Map of Indiana showing physiographic divisions. Indiana Geological Survey Miscellaneous Map 69, 15 pp. Accessed April 12, 2021 from https://igws.indiana.edu/ReferenceDocs/Maps/PhysiographicRegions.pdf	Bloomington Ridged Plain approximated from Figs. 1 and 2 of Leighton et al. (1948) and Fig. 1 by Stephenson (1967); "Central Wabash Valley" delineated by Gray (2001) was included for the Indiana portion of the subarea
Columbus Lowland and Darby Plain, Central Lowland Till Plain	Brockman, S. (1998). Physiographic regions of Ohio. Ohio Department of Natural Resources, Division of Geological Survey map. Accessed April 12, 20221 from https://www.epa.state.oh.us/portals/27/SIP/Nonattain/F2-physiographic_regions_of_Ohio.pdf	Fenneman, N.M., Johnson, D.W. (1946). Physiographic divisions of the conterminous United States. U.S. Geological Survey map, 1:7,000,000 Scale.	-	Broader Till Plain physiographic region approximated from Fenneman and Johnson (1946). Columbus Lowland and Darby Plain areas approximated from the map by Brockman (1998).
Galion Glaciated Low Plateau, Central Lowland Till Plain	Brockman, S. (1998). Physiographic regions of Ohio. Ohio Department of Natural Resources, Division of Geological Survey map. Accessed April 12, 20221 from https://www.epa.state.oh.us/portals/27/SIP/Nonattain/F2-physiographic_regions_of_Ohio.pdf	Fenneman, N.M., Johnson, D.W. (1946). Physiographic divisions of the conterminous United States. U.S. Geological Survey map, 1:7,000,000 Scale.	-	Broader Till Plain physiographic region approximated from Fenneman and Johnson (1946). Galion Glaciated Low Plateau approximated from the map by Brockman (1998).
Illinoian Till Plain, Central Lowland Till Plain	Brockman, S. (1998). Physiographic regions of Ohio. Ohio Department of Natural Resources, Division of Geological Survey map. Accessed April 12, 20221 from https://www.epa.state.oh.us/portals/27/SIP/Nonattain/F2-physiographic_regions_of_Ohio.pdf	Fenneman, N.M., Johnson, D.W. (1946). Physiographic divisions of the conterminous United States. U.S. Geological Survey map, 1:7,000,000 Scale.	-	Broader Till Plain physiographic region approximated from Fenneman and Johnson (1946). Illinoian Till Plain approximated from the map by Brockman (1998).
Iroquois Till Plains, Central Lowland Till Plain	Fenneman, N.M., Johnson, D.W. (1946). Physiographic divisions of the conterminous United States. U.S. Geological Survey map, 1:7,000,000 Scale.	Gray, H.H. (2001). Map of Indiana showing physiographic divisions. Indiana Geological Survey Miscellaneous Map 69, 15 pp. Accessed April 12, 2021 from https://igws.indiana.edu/ReferenceDocs/Maps/PhysiographicRegions.pdf	-	Broader Till Plain physiographic region approximated from Fenneman and Johnson (1946). "Iroquois Till Plain" delineated from Gray (2001).
Mount Vernon Hill County, Central Lowland Till Plain	Leighton, M. M., Ekblaw, G. E., Horberg, L. (1948). Physiographic divisions of Illinois. The Journal of Geology, 56, 16-33.	Fenneman, N.M., Johnson, D.W. (1946). Physiographic divisions of the conterminous United States. U.S. Geological Survey map, 1:7,000,000 Scale.	-	Broader Till Plain physiographic region approximated from Fenneman and Johnson (1946). Mount Vernon Hill County approximated from Figs. 1 and 2 of Leighton et al. (1948)
Newcastle Till Plain, Central Lowland Till Plain	Gray, H.H. (2001). Map of Indiana showing physiographic divisions. Indiana Geological Survey Miscellaneous Map 69, 15 pp. Accessed April 12, 2021 from https://igws.indiana.edu/ReferenceDocs/Maps/PhysiographicRegions.pdf	Brockman, S. (1998). Physiographic regions of Ohio. Ohio Department of Natural Resources, Division of Geological Survey map. Accessed April 12, 20221 from https://www.epa.state.oh.us/portals/27/SIP/Nonattain/F2-physiographic_regions_of_Ohio.pdf	Fenneman, N.M., Johnson, D.W. (1946). Physiographic divisions of the conterminous United States. U.S. Geological Survey map, 1:7,000,000 Scale.	Broader Till Plain physiographic region approximated from Fenneman and Johnson (1946). Newcastle Till Plain approximated for Indiana from map by Gray (2001) and for Ohio following the approximate border of the "Southern Ohio Loamy Till Plain" defined in map by Brockman (1998).
Springfield Plain, Central Lowland Till Plain	Leighton, M. M., Ekblaw, G. E., Horberg, L. (1948). Physiographic divisions of Illinois. The Journal of Geology, 56, 16-33.	Fenneman, N.M., Johnson, D.W. (1946). Physiographic divisions of the conterminous United	Gray, H.H. (2001). Map of Indiana showing physiographic divisions.	Broader Till Plain physiographic region approximated from Fenneman and Johnson (1946). Springfield Plain approximated from

Aquifer	Reference 1	Reference 2	Reference 3	Steps taken to delineate
		States. U.S. Geological Survey map, 1:7,000,000 Scale.	Indiana Geological Survey Miscellaneous Map 69, 15 pp. Accessed April 12, 2021 from https://igws.indiana.edu/ReferenceDocs/Maps/PhysiographicRegions.pdf	Figs. 1 and 2 of Leighton et al. (1948); northern portion of "Wabash Lowland" delineated by Gray (2001) was included for the Indiana portion of the subarea
Tipton Till Plain, Central Lowland Till Plain	Gray, H.H. (2001). Map of Indiana showing physiographic divisions. Indiana Geological Survey Miscellaneous Map 69, 15 pp. Accessed April 12, 2021 from https://igws.indiana.edu/ReferenceDocs/Maps/PhysiographicRegions.pdf	Fenneman, N.M., Johnson, D.W. (1946). Physiographic divisions of the conterminous United States. U.S. Geological Survey map, 1:7,000,000 Scale.	-	Broader Till Plain physiographic region approximated from Fenneman and Johnson (1946). Tipton Till Plain approximated from the map by Gray (2001).
Central Plain, Central Snake River Plain	Graham, W. G., Campbell, L. J. (1981). Groundwater resources of Idaho. Idaho Department of Water Resources Report, 61 pp. Accessed March 23, 2021 from https://idwr.idaho.gov/files/publications/198108-MISC-GW-Resources-ID.pdf	Lindholm, G. F. (1996). Summary of the Snake River Plain regional aquifer-system analysis in Idaho and eastern Oregon. U.S Geological Survey Professional Paper 1408-A. 59 pp. Accessed March 1, 2021 via https://pubs.usgs.gov/pp/1408a/report.pdf	-	Approximated from Plate 1 of Graham and Campbell (1981). Broader portions of the Snake River Plain (i.e., Central, Western and Eastern) were approximated from Fig. 1 of Lindholm (1996); "Eastern" portion as defined by Lindholm (1996) was split into two portions (entitled "Central" and "Eastern" Snake River Plain, respectively) at Craters of the Moon National Monument.
Goose Creek and Golden Valley, Central Snake River Plain	Graham, W. G., Campbell, L. J. (1981). Groundwater resources of Idaho. Idaho Department of Water Resources Report, 61 pp. Accessed March 23, 2021 from https://idwr.idaho.gov/files/publications/198108-MISC-GW-Resources-ID.pdf	-	-	Approximated from Plate 1 of Graham and Campbell (1981).
Marsh Valley, Central Snake River Plain	Graham, W. G., Campbell, L. J. (1981). Groundwater resources of Idaho. Idaho Department of Water Resources Report, 61 pp. Accessed March 23, 2021 from https://idwr.idaho.gov/files/publications/198108-MISC-GW-Resources-ID.pdf	-	-	Approximated from Plate 1 of Graham and Campbell (1981).
Raft River Valley, Central Snake River Plain	Graham, W. G., Campbell, L. J. (1981). Groundwater resources of Idaho. Idaho Department of Water Resources Report, 61 pp. Accessed March 23, 2021 from https://idwr.idaho.gov/files/publications/198108-MISC-GW-Resources-ID.pdf	-	-	Approximated from Plate 1 of Graham and Campbell (1981).
Twin Falls, Central Snake River Plain	Graham, W. G., Campbell, L. J. (1981). Groundwater resources of Idaho. Idaho Department of Water Resources Report, 61 pp. Accessed March 23, 2021 from https://idwr.idaho.gov/files/publications/198108-MISC-GW-Resources-ID.pdf	-	-	Approximated from Plate 1 of Graham and Campbell (1981).
Blue Mountains and Clearwater Embayment, Columbia Plateau Regional Aquifer System	Kahle, S.C., Morgan, D.S., Welch, W.B., Ely, S.R., Vaccaro, J.J., Orzol, L.L. (2011). Hydrogeologic Framework and Hydrologic Budget Components of the Columbia Plateau Regional Aquifer System, Washington, Oregon, and Idaho. US Geological Survey Scientific Investigations Report 2011–5124, 80 pp. Accessed February 16, 2021 from https://pubs.usgs.gov/sir/2011/5124/pdf/sir20115124.pdf	Graham, W. G., Campbell, L. J. (1981). Groundwater resources of Idaho. Idaho Department of Water Resources Report, 61 pp. Accessed March 23, 2021 from https://idwr.idaho.gov/files/publications/198108-MISC-GW-Resources-ID.pdf	-	Physiographic Provinces (Blue Mountains, Clearwater Embayment) approximated from Fig. 1 of Kahle et al. (2011). Idaho portion approximated following Plate 1 of Graham and Campbell (1981; see "Clearwater Uplands (Mussel Shell Basalt Subsection"). Columbia River Aquifer System approximated from Figs. 1 and 10 of Kahle et al. (2011).
Klickitat Valley, Columbia Plateau Regional Aquifer System	Kahle, S.C., Morgan, D.S., Welch, W.B., Ely, S.R., Vaccaro, J.J., Orzol, L.L. (2011). Hydrogeologic Framework and Hydrologic Budget Components of the Columbia Plateau Regional Aquifer System, Washington, Oregon, and Idaho. US Geological Survey Scientific Investigations Report 2011–5124, 80 pp. Accessed February 16, 2021 from https://pubs.usgs.gov/sir/2011/5124/pdf/sir20115124.pdf	Luzier, J.E. (1969). Ground-water occurrence in the Goldendale area, Klickitat County, Washington. US Geological Survey Numbered Series, Hydrologic Atlas. Accessed February 23, 2021	-	Approximate Klickitat Valley boundary from Plate 1 of Luzier (1969). Columbia River Aquifer System approximated from Figs. 1 and 10 of Kahle et al. (2011).

Aquifer	Reference 1	Reference 2	Reference 3	Steps taken to delineate
		from https://pubs.er.usgs.gov/publication/ha313		
Little Spokane Basin, Columbia Plateau Regional Aquifer System	Kahle, S.C., Morgan, D.S., Welch, W.B., Ely, S.R., Vaccaro, J.J., Orzol, L.L. (2011). Hydrogeologic Framework and Hydrologic Budget Components of the Columbia Plateau Regional Aquifer System, Washington, Oregon, and Idaho. US Geological Survey Scientific Investigations Report 2011–5124, 80 pp. Accessed February 16, 2021 from https://pubs.usgs.gov/sir/2011/5124/pdf/sir20115124.pdf	Kahle, S. C., Olsen, T. D., Fasser, E. T. (2013). Hydrogeology of the Little Spokane River Basin, Spokane, Stevens, and Pend Oreille Counties, Washington. US Geological Survey Scientific Investigations Report 2013-5124, 64 pp. Accessed February 18, 2021 from https://pubs.usgs.gov/sir/2013/5124/pdf/sir20135124.pdf	-	Little Spokane Basin approximated from Fig. 5 of Kahle et al. (2012). Columbia River Aquifer System approximated from Figs. 1 and 10 of Kahle et al. (2011).
Lower Deschutes Area, Columbia Plateau Regional Aquifer System	Deschutes River Conservancy (2021). Webpage entitled "The Deschutes River Basin" accessed October 9, 2021 from https://www.deschutesriver.org/where-we-work/	-	-	Approximated from Deschutes River Conservancy (2021).
Northern Columbia River Basin, Columbia Plateau Regional Aquifer System	Kahle, S.C., Morgan, D.S., Welch, W.B., Ely, S.R., Vaccaro, J.J., Orzol, L.L. (2011). Hydrogeologic Framework and Hydrologic Budget Components of the Columbia Plateau Regional Aquifer System, Washington, Oregon, and Idaho. US Geological Survey Scientific Investigations Report 2011–5124, 80 pp. Accessed February 16, 2021 from https://pubs.usgs.gov/sir/2011/5124/pdf/sir20115124.pdf	-	-	Columbia River Aquifer System approximated from Figs. 1 and 10 of Kahle et al. (2011).
Odessa Subregion, Columbia Plateau Regional Aquifer System	United States Bureau of Reclamation (2012). Final Feasibility-Level Special Study Report Odessa Subarea Special Study. 244 pp. Accessed February 18, 2021 from https://www.usbr.gov/pn/programs/eis/odessa/finaleis/final.pdf	Kahle, S.C., Morgan, D.S., Welch, W.B., Ely, S.R., Vaccaro, J.J., Orzol, L.L. (2011). Hydrogeologic Framework and Hydrologic Budget Components of the Columbia Plateau Regional Aquifer System, Washington, Oregon, and Idaho. US Geological Survey Scientific Investigations Report 2011–5124, 80 pp. Accessed February 16, 2021 from https://pubs.usgs.gov/sir/2011/5124/pdf/sir20115124.pdf	Vaccaro, J.J., Kahle, S.C., Ely, D.M., Burns, E.R., Snyder, D.T., Haynes, J.V., Olsen, T.D., Welch, W.B., Morgan, D.S. (2015). Groundwater availability of the Columbia Plateau Regional Aquifer System, Washington, Oregon, and Idaho: U.S. Geological Survey Professional Paper 1817, 87 pp. Accessed February 18, 2021 from http://dx.doi.org/10.3133/pp1817.	Odessa subarea approximated from location map on page 75 (Fig. 4-10) of United States Bureau of Reclamation (2012) and Fig. 38 of Vaccaro et al. (2015). Broader Columbia River Aquifer System approximated from Figs. 1 and 10 of Kahle et al. (2011).
Palouse Basin, Columbia Plateau Regional Aquifer System	Douglas, A. A., Osiensky, J. L., Keller, C. K. (2007). Carbon-14 dating of ground water in the Palouse Basin of the Columbia River basalts. Journal of Hydrology, 334(3-4), 502-512.	Kahle, S.C., Morgan, D.S., Welch, W.B., Ely, S.R., Vaccaro, J.J., Orzol, L.L. (2011). Hydrogeologic Framework and Hydrologic Budget Components of the Columbia Plateau Regional Aquifer System, Washington, Oregon, and Idaho. US Geological Survey Scientific Investigations Report 2011–5124, 80 pp. Accessed February 16, 2021 from	Reidel, S. P., Spane, F. A., Johnson, V. G. (2002). Natural gas storage in basalt aquifers of the Columbia basin, Pacific Northwest USA: A guide to site characterization. Pacific Northwest National Lab (PNNL) Report n No. PNNL-13962. Richland, Washington, USA, 277 pp. Accessed February 16, 2021 from	Palouse Basin approximated based on Fig. 1 of Douglas et al. (2007). Broader Columbia River Aquifer System approximated from Figs. 1 and 10 of Kahle et al. (2011) and sub-province map shown in Fig. 2.1 of Reidel et al. (2002).

Aquifer	Reference 1	Reference 2	Reference 3	Steps taken to delineate
		https://pubs.usgs.gov/sir/2011/5124/pdf/sir20115124.pdf	https://www.pnnl.gov/main/publications/external/technical_reports/PNNL-13962.pdf	
Palouse Slope, Columbia Plateau Regional Aquifer System	Kahle, S.C., Morgan, D.S., Welch, W.B., Ely, S.R., Vaccaro, J.J., Orzol, L.L. (2011). Hydrogeologic Framework and Hydrologic Budget Components of the Columbia Plateau Regional Aquifer System, Washington, Oregon, and Idaho. US Geological Survey Scientific Investigations Report 2011–5124, 80 pp. Accessed February 16, 2021 from https://pubs.usgs.gov/sir/2011/5124/pdf/sir20115124.pdf	-	-	Physiographic Provinces (Blue Mountains, Clearwater Embayment) approximated from Fig. 1 of Kahle et al. (2011). Columbia River Aquifer System approximated from Figs. 1 and 10 of Kahle et al. (2011).
Quincy Subregion, Columbia Plateau Regional Aquifer System	United States Bureau of Reclamation (2012). Final Feasibility-Level Special Study Report Odessa Subarea Special Study. 244 pp. Accessed February 18, 2021 from https://www.usbr.gov/pn/programs/eis/odessa/finaleis/final.pdf	Kahle, S.C., Morgan, D.S., Welch, W.B., Ely, S.R., Vaccaro, J.J., Orzol, L.L. (2011). Hydrogeologic Framework and Hydrologic Budget Components of the Columbia Plateau Regional Aquifer System, Washington, Oregon, and Idaho. US Geological Survey Scientific Investigations Report 2011–5124, 80 pp. Accessed February 16, 2021 from https://pubs.usgs.gov/sir/2011/5124/pdf/sir20115124.pdf	Vaccaro, J.J., Kahle, S.C., Ely, D.M., Burns, E.R., Snyder, D.T., Haynes, J.V., Olsen, T.D., Welch, W.B., Morgan, D.S. (2015). Groundwater availability of the Columbia Plateau Regional Aquifer System, Washington, Oregon, and Idaho: U.S. Geological Survey Professional Paper 1817, 87 pp. Accessed February 18, 2021 from http://dx.doi.org/10.3133/pp1817.	Quincy subarea approximated from location map on page 75 (Fig. 4-10) of US Bureau of Reclamation (2012) and Fig. 38 of Vaccaro et al. (2015). Broader Columbia River Aquifer System approximated from Figs. 1 and 10 of Kahle et al. (2011).
Spokane Valley-Rathdrum Prairie Aquifer, Columbia Plateau Regional Aquifer System	Kahle, S.C., Morgan, D.S., Welch, W.B., Ely, S.R., Vaccaro, J.J., Orzol, L.L. (2011). Hydrogeologic Framework and Hydrologic Budget Components of the Columbia Plateau Regional Aquifer System, Washington, Oregon, and Idaho. US Geological Survey Scientific Investigations Report 2011–5124, 80 pp. Accessed February 16, 2021 from https://pubs.usgs.gov/sir/2011/5124/pdf/sir20115124.pdf	Hsieh, P. A., Barber, M. E., Contor, B. A., Hossain, M., Johnson, G. S., Jones, J. L., Wylie, A. H. (2007). Ground-water flow model for the Spokane valley-Rathdrum prairie aquifer, Spokane County, Washington, and Bonner and Kootenai Counties, Idaho. U. S. Geological Survey Scientific Investigations Report 2007-5044, 90 pp. Accessed February 18, 2021 from https://pubs.usgs.gov/sir/2007/5044/pdf/sir20075044.pdf	Graham, W. G., Campbell, L. J. (1981). Groundwater resources of Idaho. Idaho Department of Water Resources Report, 61 pp. Accessed March 23, 2021 from https://idwr.idaho.gov/files/publications/198108-MISC-GW-Resources-ID.pdf	Spokane Valley-Rathdrum Prairie Aquifer approximated from Fig. 1 of Hseih et al. (2007). Columbia River Aquifer System approximated from Figs. 1 and 10 of Kahle et al. (2011). Idaho portion delineated from Graham and Campbell (1981).
Umatilla Basin and Horse Heaven Hills, Columbia Plateau Regional Aquifer System	Kahle, S.C., Morgan, D.S., Welch, W.B., Ely, S.R., Vaccaro, J.J., Orzol, L.L. (2011). Hydrogeologic Framework and Hydrologic Budget Components of the Columbia Plateau Regional Aquifer System, Washington, Oregon, and Idaho. US Geological Survey Scientific Investigations Report 2011–5124, 80 pp. Accessed February 16, 2021 from https://pubs.usgs.gov/sir/2011/5124/pdf/sir20115124.pdf	Herrera, N. B., Ely, K., Mehta, S., Stonewall, A. J., Risley, J. C., Hinkle, S. R., Conlon, T. D. (2017). Hydrogeologic framework and selected components of the groundwater budget for the upper Umatilla River Basin, Oregon (No. 2017-5020). US Geological Survey Scientific Investigations Report 2017-5020, 68 pp. Accessed February 18, 2021 from	Davies-Smith, A., Bolke, E. L., Collins, C. A. (1988). Geohydrology and digital simulation of the ground-water flow system in the Umatilla Plateau and Horse Heaven Hills area, Oregon and Washington. Water-Resources Investigations Report 87-4268, 77 pp. Accessed February 23, 2021 from	Approximate Umatilla Basin boundaries from Fig. 9 of Kahle et al. (2011); see also Fig. 3 of Herrera et al. (2017). Horse Heaven Hills extent (and co-study with Umatilla Basin) from Fig. 1 of Davies-Smith et al. (1983). Columbia River Aquifer System approximated from Figs. 1 and 10 of Kahle et al. (2011).

Aquifer	Reference 1	Reference 2	Reference 3	Steps taken to delineate
		https://pubs.usgs.gov/sir/2017/5020/sir20175020.pdf	https://pubs.usgs.gov/wri/1987/4268/report.pdf	
Walla Walla Basin, Columbia Plateau Regional Aquifer System	Kahle, S.C., Morgan, D.S., Welch, W.B., Ely, S.R., Vaccaro, J.J., Orzol, L.L. (2011). Hydrogeologic Framework and Hydrologic Budget Components of the Columbia Plateau Regional Aquifer System, Washington, Oregon, and Idaho. US Geological Survey Scientific Investigations Report 2011–5124, 80 pp. Accessed February 16, 2021 from https://pubs.usgs.gov/sir/2011/5124/pdf/sir20115124.pdf	Henry, R., Lindsay, K., Wolcott, B., Patten, S., Baker, T. (2013). Walla Walla Basin Aquifer Recharge Strategic Plan. Walla Walla Basin Watershed Council Report, 106 pp. Accessed February 23, 2021 from http://wwbwc.org/images/Projects/AR/Reports/RechargeStrategy_FINAL_1-29-13_sp.pdf	Newcomb, R.C. (1965). Geology and ground-water resources of the Walla Walla River Basin, Washington-Oregon: Washington Division of Water Resources Water-Supply Bulletin 21, 162 pp. Accessed November 29, 2021 from https://apps.ecology.wa.gov/publications/documents/wsb21.pdfComment created	Walla Walla Basin outline approximated from Fig. 1 of Walla Walla Basin Watershed Council Report by Henry et al. (2013) and Fig. 1 by Newcomb (1965). Columbia River Aquifer System approximated from Figs. 1 and 10 of Kahle et al. (2011).
Yakima Basin, Columbia Plateau Regional Aquifer System	Burns, E. R., Snyder, D. T., Haynes, J. V., Waibel, M. S. (2012). Groundwater status and trends for the Columbia Plateau Regional Aquifer System, Washington, Oregon, and Idaho. U.S. Geological Survey Scientific Investigations Report 2012–5261, 52 pp. Accessed February 18, 2021 from http://pubs.er.usgs.gov/publication/sir20125261.	Kahle, S.C., Morgan, D.S., Welch, W.B., Ely, S.R., Vaccaro, J.J., Orzol, L.L. (2011). Hydrogeologic Framework and Hydrologic Budget Components of the Columbia Plateau Regional Aquifer System, Washington, Oregon, and Idaho. US Geological Survey Scientific Investigations Report 2011–5124, 80 pp. Accessed February 16, 2021 from https://pubs.usgs.gov/sir/2011/5124/pdf/sir20115124.pdf	Reidel, S. P., Spane, F. A., Johnson, V. G. (2002). Natural gas storage in basalt aquifers of the Columbia basin, Pacific Northwest USA: A guide to site characterization. Pacific Northwest National Lab (PNNL) Report n No. PNNL-13962. Richland, Washington, USA, 277 pp. Accessed February 16, 2021 from https://www.pnnl.gov/main/publications/external/technical_reports/PNNL-13962.pdf	Yakima Basin approximated based on Fig. 7 of Burns et al. (2012). Broader Columbia River Aquifer System approximated from Figs. 1 and 10 of Kahle et al. (2011) and sub-province map shown in Fig. 2.1 of Reidel et al. (2002).
Lower Colville Basin, Colville Basin	Kahle, S.C., Longpré, C.I., Smith, R.R., Sumioka, S.S., Watkins, A.M., Kresch, D.L. (2003). Water Resources of the Ground-Water System in the Unconsolidated Deposits of the Colville River Watershed, Stevens County, Washington. U.S. Geological Survey Water-Resources Investigations Report 03-4128, 84 pp. Accessed March 15, 2021 from https://pubs.usgs.gov/wri/wri034128/pdf/wri034128.pdf	-	-	Approximated from Fig. 6 of Kahle et al. (2003) with guidance from well completion report data. Upper and Lower basins defined near the town of Addy (Washington).
Upper Colville Basin, Colville Basin	Kahle, S.C., Longpré, C.I., Smith, R.R., Sumioka, S.S., Watkins, A.M., Kresch, D.L. (2003). Water Resources of the Ground-Water System in the Unconsolidated Deposits of the Colville River Watershed, Stevens County, Washington. U.S. Geological Survey Water-Resources Investigations Report 03-4128, 84 pp. Accessed March 15, 2021 from https://pubs.usgs.gov/wri/wri034128/pdf/wri034128.pdf	-	-	Approximated from Fig. 6 of Kahle et al. (2003) with guidance from well completion report data. Upper and Lower basins defined near the town of Addy (Washington).
Arbon Valley, Eastern Snake River Plain	Graham, W. G., Campbell, L. J. (1981). Groundwater resources of Idaho. Idaho Department of Water Resources Report, 61 pp. Accessed March 23, 2021 from https://idwr.idaho.gov/files/publications/198108-MISC-GW-Resources-ID.pdf	-	-	Approximated from Plate 1 of Graham and Campbell (1981).
Big Lost River Valley, Eastern Snake River Plain	Graham, W. G., Campbell, L. J. (1981). Groundwater resources of Idaho. Idaho Department of Water Resources Report, 61 pp. Accessed March 23, 2021 from https://idwr.idaho.gov/files/publications/198108-MISC-GW-Resources-ID.pdf	-	-	Approximated from Plate 1 of Graham and Campbell (1981).

Aquifer	Reference 1	Reference 2	Reference 3	Steps taken to delineate
Eastern Plain, Eastern Snake River Plain	Graham, W. G., Campbell, L. J. (1981). Groundwater resources of Idaho. Idaho Department of Water Resources Report, 61 pp. Accessed March 23, 2021 from https://idwr.idaho.gov/files/publications/198108-MISC-GW-Resources-ID.pdf	-	-	Approximated from Plate 1 of Graham and Campbell (1981) (see #39 "Snake Plain"; Volcanic Rift Zone" area (see preceding aquifer in this list) excluded from delineation)
Little Lost River Valley, Eastern Snake River Plain	Graham, W. G., Campbell, L. J. (1981). Groundwater resources of Idaho. Idaho Department of Water Resources Report, 61 pp. Accessed March 23, 2021 from https://idwr.idaho.gov/files/publications/198108-MISC-GW-Resources-ID.pdf	-	-	Approximated from Plate 1 of Graham and Campbell (1981).
Marsh Creek, Eastern Snake River Plain	Graham, W. G., Campbell, L. J. (1981). Groundwater resources of Idaho. Idaho Department of Water Resources Report, 61 pp. Accessed March 23, 2021 from https://idwr.idaho.gov/files/publications/198108-MISC-GW-Resources-ID.pdf	-	-	Approximated from Plate 1 of Graham and Campbell (1981).
Mud Lake, Eastern Snake River Plain	Rattray, G. (2015). Geochemical evolution of groundwater in the Mud Lake area, Eastern Idaho, USA. Environmental Earth Sciences, 73(12), 8251-8269.	-	-	Approximated from Fig. 2 of Rattray (2015)
Rockland Valley, Eastern Snake River Plain	Graham, W. G., Campbell, L. J. (1981). Groundwater resources of Idaho. Idaho Department of Water Resources Report, 61 pp. Accessed March 23, 2021 from https://idwr.idaho.gov/files/publications/198108-MISC-GW-Resources-ID.pdf	-	-	Approximated from Plate 1 of Graham and Campbell (1981).
Volcanic Rift Zone, Eastern Snake River Plain	Whitehead, R. L. (1992). Geohydrologic framework of the Snake River Plain regional aquifer system, Idaho and eastern Oregon. US Geological Survey Professional Paper 1408-B, 39 pp. Accessed March 1, 2021 via https://pubs.usgs.gov/pp/1408b/report.pdf	-	-	Approximated from Plate 1 of Whitehead (1992).
Balcones Fault Zone, Edwards-Trinity Aquifer System	Bruun, B., Jackson, K., Lake, P., Walker, J. (2016). Texas Aquifers Study. Texas Water Development Board Report. 336 pp. Accessed April 1, 2021 from https://www.twdb.texas.gov/groundwater/docs/studies/TexasAquifersStudy_2016.pdf#page=89	Barker, R. A., Ardis, A. F. (1996). Hydrogeological framework of the Edwards-Trinity aquifer system, west-central Texas. U.S. Geological Survey Professional Paper 1421-B, 76 pp. Accessed April 1, 2021 from https://pubs.usgs.gov/pp/1421b/report.pdf	-	Approximated from Fig. 6-6 by Bruun et al. (2016) and Fig. 3 by Barker and Ardis (1996).
Edwards Plateau, Edwards-Trinity Aquifer System	Bruun, B., Jackson, K., Lake, P., Walker, J. (2016). Texas Aquifers Study. Texas Water Development Board Report. 336 pp. Accessed April 1, 2021 from https://www.twdb.texas.gov/groundwater/docs/studies/TexasAquifersStudy_2016.pdf#page=89	Barker, R. A., Ardis, A. F. (1996). Hydrogeological framework of the Edwards-Trinity aquifer system, west-central Texas. U.S. Geological Survey Professional Paper 1421-B, 76 pp. Accessed April 1, 2021 from https://pubs.usgs.gov/pp/1421b/report.pdf	-	Approximated from Fig. 6-11 by Bruun et al. (2016) and Fig. 3 by Barker and Ardis (1996).
Hill Country, Edwards-Trinity Aquifer System	Bruun, B., Jackson, K., Lake, P., Walker, J. (2016). Texas Aquifers Study. Texas Water Development Board Report. 336 pp. Accessed April 1, 2021 from https://www.twdb.texas.gov/groundwater/docs/studies/TexasAquifersStudy_2016.pdf#page=89	Barker, R. A., Ardis, A. F. (1996). Hydrogeological framework of the Edwards-Trinity aquifer system, west-central Texas. U.S. Geological Survey Professional Paper 1421-B, 76 pp. Accessed April 1, 2021 from https://pubs.usgs.gov/pp/1421b/report.pdf	-	Approximated from Fig. 6-11 by Bruun et al. (2016) and Fig. 3 by Barker and Ardis (1996).
Stockton Plateau, Edwards-Trinity Aquifer System	Bruun, B., Jackson, K., Lake, P., Walker, J. (2016). Texas Aquifers Study. Texas Water Development Board Report. 336 pp. Accessed April 1, 2021 from	Barker, R. A., Ardis, A. F. (1996). Hydrogeological framework of the Edwards-Trinity aquifer	Ryder, P. (1996). Ground Water Atlas of the United States Segment 4 Oklahoma,	Approximated from Fig. 6-11 by Bruun et al. (2016), Fig. 84 by Ryder (1996), and Fig. 3 by Barker and Ardis (1996).

Aquifer	Reference 1	Reference 2	Reference 3	Steps taken to delineate
	https://www.twdb.texas.gov/groundwater/docs/studies/TexasAquifersStudy_2016.pdf#page=89	system, west-central Texas. U.S. Geological Survey Professional Paper 1421-B, 76 pp. Accessed April 1, 2021 from https://pubs.usgs.gov/pp/1421b/report.pdf	Texas. Hydrologic Investigations Atlas 730-E, 32 pp. Accessed November 29, 2021 from https://pubs.usgs.gov/ha/730e/report.pdf	
Trinity Aquifer System, Edwards-Trinity Aquifer System	Bruun, B., Jackson, K., Lake, P., Walker, J. (2016). Texas Aquifers Study. Texas Water Development Board Report. 336 pp. Accessed April 1, 2021 from https://www.twdb.texas.gov/groundwater/docs/studies/TexasAquifersStudy_2016.pdf#page=89	Gordon, C.H. (1913). Geology and underground waters of the Wichita region, north-central Texas. U.S. Geological Survey Water Supply Paper 317, 90 pp. Accessed April 7, 2021 from https://pubs.usgs.gov/wsp/0317/report.pdf	Ryder, P. (1996). Ground Water Atlas of the United States Segment 4 Oklahoma, Texas. Hydrologic Investigations Atlas 730-E, 32 pp. Accessed November 29, 2021 from https://pubs.usgs.gov/ha/730e/report.pdf	Approximated from Fig. 6-37 by Bruun et al. (2016) and Fig. 114 by Ryder (1996). Delineated area includes the northeast portion of the Balcones Fault Zone following the major aquifer delineation of in map on page vii of Bruun et al. (2016). Local hydrogeology in northern area of aquifer are detailed in Plate 1 of Gordon (1913).
Beryl-Enterprise Subarea, Escalante Valley	Sandberg, G. W. (1964). Ground-water resources of selected basins in southwestern Utah. US Geological Survey Open File Report. 66 pp. Accessed March 7, 2021 from https://cicwcd.org/wp-content/uploads/2018/09/1966-tech-pub-13-ground-water-resources-of-selected-basins-in-southwestern-utah-by-g-w-sandburg-usgs.pdf	Fix, P. F., Nelson, W. B., Lofgren, B. E., Butler, R. G. (1950). Ground water in the Escalante Valley, Beaver, Iron, and Washington Counties, Utah Technical Publication 6. 102 pp. Accessed March 7, 2021 from https://waterrights.utah.gov/docSys/v920/w920/w9200085.pdf	-	Approximated from Fig. 1 of Fix et al. (1950) with subareas ("Districts") approximated from the map on page 49 of Sandberg (1966).
Lund Subarea, Escalante Valley	Sandberg, G. W. (1964). Ground-water resources of selected basins in southwestern Utah. US Geological Survey Open File Report. 66 pp. Accessed March 7, 2021 from https://cicwcd.org/wp-content/uploads/2018/09/1966-tech-pub-13-ground-water-resources-of-selected-basins-in-southwestern-utah-by-g-w-sandburg-usgs.pdf	Fix, P. F., Nelson, W. B., Lofgren, B. E., Butler, R. G. (1950). Ground water in the Escalante Valley, Beaver, Iron, and Washington Counties, Utah Technical Publication 6. 102 pp. Accessed March 7, 2021 from https://waterrights.utah.gov/docSys/v920/w920/w9200085.pdf	-	Approximated from Fig. 1 of Fix et al. (1950) with subareas ("Districts") approximated from the map on page 49 of Sandberg (1966).
Milford-Blackrock Subarea, Escalante Valley	Sandberg, G. W. (1966). Ground-water resources of selected basins in southwestern Utah. US Geological Survey Open File Report. 66 pp. Accessed March 7, 2021 from https://cicwcd.org/wp-content/uploads/2018/09/1966-tech-pub-13-ground-water-resources-of-selected-basins-in-southwestern-utah-by-g-w-sandburg-usgs.pdf	Fix, P. F., Nelson, W. B., Lofgren, B. E., Butler, R. G. (1950). Ground water in the Escalante Valley, Beaver, Iron, and Washington Counties, Utah Technical Publication 6. 102 pp. Accessed March 7, 2021 from https://waterrights.utah.gov/docSys/v920/w920/w9200085.pdf	-	Approximated from Fig. 1 of Fix et al. (1950) with subareas ("Districts") approximated from the map on page 49 of Sandberg (1966).
Camas Prairie Basin, Flathead Valley	LaFave, J. I., Smith, L. N., Patton, T. W. (2004). Ground-Water Resources of the Flathead Lake Area: Flathead, Lake, Missoula, and Sanders Counties, Montana. Part A – Descriptive Overview and Water-Quality Data.Montana Bureau of Mines and Geology, Butte, MT. Montana Ground-Water Assessment Atlas 2. Accessed March 2, 2021 from http://mbmg.mtech.edu/pdf/GWA_2.pdf	-	-	Approximated from Fig. 2 of LaFave et al. (2004)
Flathead Lake Perimeter, Flathead Valley	LaFave, J. I., Smith, L. N., Patton, T. W. (2004). Ground-Water Resources of the Flathead Lake Area: Flathead, Lake, Missoula, and Sanders Counties, Montana. Part A – Descriptive Overview and Water-Quality Data.Montana Bureau of Mines and Geology, Butte, MT. Montana Ground-Water	-	-	Approximated from Fig. 2 of LaFave et al. (2004)

Aquifer	Reference 1	Reference 2	Reference 3	Steps taken to delineate
	Assessment Atlas 2. Accessed March 2, 2021 from http://mbmg.mtech.edu/pdf/GWA_2.pdf			
Kalispell Valley, Flathead Valley	Wheaton, J., Rose, J., Bobst, A., Gebril, A. (2015). Flathead Valley Deep Aquifer: Geologic Setting and Hydrogeologic Implications. Montana Bureau of Mines and Geology presentation at the 2015 Clark Fork Symposium. Accessed March 2, 2021 from https://www.mbmg.mtech.edu/gwip/gwip_pdf/2015/2015_ClarkForkSymposium-Flathead.pdf	LaFave, J.I. (2000). Potentiometric surface map of the Deep Aquifer, Kalispell valley, Flathead County, Montana. Montana Ground-Water Assessment Atlas GWAA-02B-2, Accessed March 29, 2021 from https://ngmdb.usgs.gov/Prodesc/proddesc_51253.htm	-	Approximated from Figure shown in the upper right corner of the poster by Wheaton et al. (2015) and map by LaFave (2000).
Little Bitterroot Valley, Flathead Valley	LaFave, J. I., Smith, L. N., Patton, T. W. (2004). Ground-Water Resources of the Flathead Lake Area: Flathead, Lake, Missoula, and Sanders Counties, Montana. Part A – Descriptive Overview and Water-Quality Data.Montana Bureau of Mines and Geology, Butte, MT. Montana Ground-Water Assessment Atlas 2. Accessed March 2, 2021 from http://mbmg.mtech.edu/pdf/GWA_2.pdf	Meinzer, O.E. (1916a). Artesian water for irrigation in Little Bitterroot Valley, Montana. Water Supply Paper 400, 34 pp. Accessed April 7, 2021 from https://pubs.usgs.gov/wsp/0400b/report.pdf	-	Approximated from Fig. 2 of LaFave et al. (2004) and Fig. 1 of Meinzer (1916a).
Mission Valley and Irvine Flats, Flathead Valley	LaFave, J. I. (2004). Potentiometric Surface Map of the Southern Part of the Flathead Lake Area, Lake, Missoula, Sanders Counties, Montana. Montana Ground-Water Assessment Atlas No. 2, Part B, Map 4. Montana Bureau of Mines and Geology, A Department of Montana Tech of The University of Montana, Butte, MT.	Smith, L.N. (2004). Hydrogeologic framework of the southern part of the Flathead Lake Area, Flathead, Lake, Missoula, and Sanders counties, Montana : Montana Bureau of Mines and Geology Montana Ground-Water Assessment Atlas 2-B-10, 1 sheet, scale 1:300,000. Accessed March 2, 2021 from http://mbmggwic.mtech.edu/gwcpmaps/gwaa02map10untiled.pdf	-	Approximated from map by Smith (2004) and LaFave (2004).
Smith Subarea, Flathead Valley	LaFave, J. I., Smith, L. N., Patton, T. W. (2004). Ground-Water Resources of the Flathead Lake Area: Flathead, Lake, Missoula, and Sanders Counties, Montana. Part A – Descriptive Overview and Water-Quality Data.Montana Bureau of Mines and Geology, Butte, MT. Montana Ground-Water Assessment Atlas 2. Accessed March 2, 2021 from http://mbmg.mtech.edu/pdf/GWA_2.pdf	-	-	Approximated from Fig. 2 of LaFave et al. (2004)
Apalachicola Delta, Floridan Aquifer System	Davis, H. (1996). Hydrogeologic Investigation and Simulation of Ground-Water Flow in the Upper Floridan Aquifer of North-Central Florida and Southwestern Georgia and Delineation of Contributing Areas for Selected City of Tallahassee, Florida, Water-Supply Wells. U.S. Geological Survey Water-Resources investigations Report 95-4296, 61 pp. Accessed March 31, 2021 from https://fl.water.usgs.gov/PDF_files/wri95_4296_davis.pdf	Clark, W. Z., Zisa, A. C. (1976) Physiographic map of Georgia: Georgia Department of Natural Resources, 1 sheet. Accessed March 31, 2021 from https://epd.georgia.gov/document/publication/sm-4-physiographic-map-georgia-12000000-1988/download	Brooks, H. K. (1981). Physiographic divisions of Florida. Report for the Florida Cooperative Extension Service Institute of Food and Agricultural Sciences, University of Florida, 12 pp.	Approximated from Fig. 2 of Davis (1996) and the physiographic provinces for Georgia and Florida by Clark and Zisa (1976) and Brooks (1981)
Bacon Terrace, Floridan Aquifer System	Williams, L.J., Kuniansky, E.L. (2016). Revised hydrogeologic framework of the Floridan aquifer system in Florida and parts of Georgia, Alabama, and South Carolina. U.S. Geological Survey Professional Paper 1807, 140 pp. Accessed March 31, 2021 from https://pubs.usgs.gov/pp/1807/pdf/pp1807.pdf	Clark, W. Z., Zisa, A. C. (1976) Physiographic map of Georgia: Georgia Department of Natural Resources, 1 sheet. Accessed March 31, 2021 from	-	Approximated from physiographic provinces of Georgia outlined by Clark and Zisa (1976). Broader Florida Aquifer System from Williams and Kuniansky (2016).

Aquifer	Reference 1	Reference 2	Reference 3	Steps taken to delineate
		https://epd.georgia.gov/document/publication/sm-4-physiographic-map-georgia-12000000-1988/download		
Central Lake Area, Floridan Aquifer System	Williams, L.J., Kuniansky, E.L. (2016). Revised hydrogeologic framework of the Floridan aquifer system in Florida and parts of Georgia, Alabama, and South Carolina. U.S. Geological Survey Professional Paper 1807, 140 pp. Accessed March 31, 2021 from https://pubs.usgs.gov/pp/1807/pdf/pp1807.pdf	Brooks, H. K. (1981). Physiographic divisions of Florida. Report for the Florida Cooperative Extension Service Institute of Food and Agricultural Sciences, University of Florida, 12 pp.	-	Approximated from physiographic provinces of Florida outlined by Brooks (1981). Broader Florida Aquifer System from Williams and Kuniansky (2016).
Dougherty Plain and Marianna Lowlands, Floridan Aquifer System	Williams, L.J., Kuniansky, E.L. (2016). Revised hydrogeologic framework of the Floridan aquifer system in Florida and parts of Georgia, Alabama, and South Carolina. U.S. Geological Survey Professional Paper 1807, 140 pp. Accessed March 31, 2021 from https://pubs.usgs.gov/pp/1807/pdf/pp1807.pdf	Clark, W. Z., Zisa, A. C. (1976) Physiographic map of Georgia: Georgia Department of Natural Resources, 1 sheet. Accessed March 31, 2021 from https://epd.georgia.gov/document/publication/sm-4-physiographic-map-georgia-12000000-1988/download	-	Approximated from Fig. 21 of Williams and Kuniansky (2016). Georgia portion of area also provided by Clark and Zisa (1976).
Eastern Flatwoods Northshores, Floridan Aquifer System	Williams, L.J., Kuniansky, E.L. (2016). Revised hydrogeologic framework of the Floridan aquifer system in Florida and parts of Georgia, Alabama, and South Carolina. U.S. Geological Survey Professional Paper 1807, 140 pp. Accessed March 31, 2021 from https://pubs.usgs.gov/pp/1807/pdf/pp1807.pdf	Brooks, H. K. (1981). Physiographic divisions of Florida. Report for the Florida Cooperative Extension Service Institute of Food and Agricultural Sciences, University of Florida, 12 pp.	-	Approximated from physiographic provinces of Florida outlined by Brooks (1981). Broader Florida Aquifer System from Williams and Kuniansky (2016). Eastern Flatwoods as defined by Brooks (1981) was split at Lake Harney into two areas north and south of Lake Harney ('northshores' and 'southshores').
Eastern Flatwoods Southshores, Floridan Aquifer System	Williams, L.J., Kuniansky, E.L. (2016). Revised hydrogeologic framework of the Floridan aquifer system in Florida and parts of Georgia, Alabama, and South Carolina. U.S. Geological Survey Professional Paper 1807, 140 pp. Accessed March 31, 2021 from https://pubs.usgs.gov/pp/1807/pdf/pp1807.pdf	Brooks, H. K. (1981). Physiographic divisions of Florida. Report for the Florida Cooperative Extension Service Institute of Food and Agricultural Sciences, University of Florida, 12 pp.	-	Approximated from physiographic provinces of Florida outlined by Brooks (1981). Broader Florida Aquifer System from Williams and Kuniansky (2016). Eastern Flatwoods as defined by Brooks (1981) was split at Lake Harney into two areas north and south of Lake Harney ('northshores' and 'southshores').
Intermediate Aquifer, Floridan Aquifer System	Miller, J.A. (1990). Ground Water Atlas of the United States: Segment 6, Alabama, Florida, Georgia, South Carolina. U.S. Geological Survey Hydrologic Atlas 730-G, 30 pp. Accessed April 5, 2021 from https://www.nrc.gov/docs/ML1706/ML17060B027.pdf	Knochenmus, L.A. (2006). Regional Evaluation of the Hydrogeologic Framework, Hydraulic Properties, and Chemical Characteristics of the Intermediate Aquifer System Underlying Southern West-Central Florida. U.S. Geological Survey Scientific Investigations Report 2006-5013, 52 pp. Accessed April 5, 2021 from https://pubs.usgs.gov/sir/2006/5013/pdf/2006-5013.pdf	-	Approximated Fig. 1 of Knochenmus (2006); aquifer outline also displayed in Figs. 9 and 42 of Miller (1990)
Lower Coastal Plain, Floridan Aquifer System	Williams, L.J., Kuniansky, E.L. (2016). Revised hydrogeologic framework of the Floridan aquifer system in Florida and parts of Georgia, Alabama, and South Carolina. U.S. Geological Survey Professional Paper 1807, 140 pp. Accessed March 31, 2021 from https://pubs.usgs.gov/pp/1807/pdf/pp1807.pdf	Aucott, W.R. (1996). Hydrology of the Southeastern Coastal Plain Aquifer System in South Carolina and Parts of Georgia and North Carolina. U.S	-	Broader Florida Aquifer System from Williams and Kuniansky (2016). Approximate divide between the Upper and Lower Coastal Plain from Fig. 1 of Aucott (1996).

Aquifer	Reference 1	Reference 2	Reference 3	Steps taken to delineate
		Geological Survey Professional Paper 1410-E, 95 pp. Accessed March 31, 2021 from https://pubs.usgs.gov/pp/1410e/report.pdf		
Ocala Uplift, Floridan Aquifer System	Davis, H. (1996). Hydrogeologic Investigation and Simulation of Ground-Water Flow in the Upper Floridan Aquifer of North-Central Florida and Southwestern Georgia and Delineation of Contributing Areas for Selected City of Tallahassee, Florida, Water-Supply Wells. U.S. Geological Survey Water-Resources investigations Report 95-4296, 61 pp. Accessed March 31, 2021 from https://fl.water.usgs.gov/PDF_files/wri95_4296_davis.pdf	Clark, W. Z., Zisa, A. C. (1976) Physiographic map of Georgia: Georgia Department of Natural Resources, 1 sheet. Accessed March 31, 2021 from https://epd.georgia.gov/document/publication/sm-4-physiographic-map-georgia-12000000-1988/download	Brooks, H. K. (1981). Physiographic divisions of Florida. Report for the Florida Cooperative Extension Service Institute of Food and Agricultural Sciences, University of Florida, 12 pp.	Approximated from Fig. 2 of Davis (1996) and the physiographic provinces for Georgia and Florida by Clark and Zisa (1976) and Brooks (1981)
Okefenokee Basin, Floridan Aquifer System	Williams, L.J., Kuniansky, E.L. (2016). Revised hydrogeologic framework of the Floridan aquifer system in Florida and parts of Georgia, Alabama, and South Carolina. U.S. Geological Survey Professional Paper 1807, 140 pp. Accessed March 31, 2021 from https://pubs.usgs.gov/pp/1807/pdf/pp1807.pdf	Clark, W. Z., Zisa, A. C. (1976) Physiographic map of Georgia: Georgia Department of Natural Resources, 1 sheet. Accessed March 31, 2021 from https://epd.georgia.gov/document/publication/sm-4-physiographic-map-georgia-12000000-1988/download	-	Approximated from physiographic provinces of Georgia outlined by Clark and Zisa (1976). Broader Florida Aquifer System from Williams and Kuniansky (2016).
Sea Island, Floridan Aquifer System	Williams, L.J., Kuniansky, E.L. (2016). Revised hydrogeologic framework of the Floridan aquifer system in Florida and parts of Georgia, Alabama, and South Carolina. U.S. Geological Survey Professional Paper 1807, 140 pp. Accessed March 31, 2021 from https://pubs.usgs.gov/pp/1807/pdf/pp1807.pdf	Brooks, H. K. (1981). Physiographic divisions of Florida. Report for the Florida Cooperative Extension Service Institute of Food and Agricultural Sciences, University of Florida, 12 pp.	Clark, W. Z., Zisa, A. C. (1976) Physiographic map of Georgia: Georgia Department of Natural Resources, 1 sheet. Accessed March 31, 2021 from https://epd.georgia.gov/document/publication/sm-4-physiographic-map-georgia-12000000-1988/download	Approximated from physiographic provinces for Georgia and Florida by Clark and Zisa (1976) and Brooks (1981). Broader Florida Aquifer System from Williams and Kuniansky (2016).
Southern Pine Hills, Floridan Aquifer System	Barraclough, J. T., Marsh, O. T. (1962). Aquifers and quality of ground water along the Gulf Coast of western Florida. U.S. Geological Survey Report of Investigations No. 62, 41 pp. Accessed March 31, 2021 from https://ufdcimages.uflib.ufl.edu/UF/00/00/12/16/00001/UF00001216.pdf	-	-	Approximated from Fig. 1 of Barraclough and Marsh (1962)
Southwestern Flatwoods, Floridan Aquifer System	Williams, L.J., Kuniansky, E.L. (2016). Revised hydrogeologic framework of the Floridan aquifer system in Florida and parts of Georgia, Alabama, and South Carolina. U.S. Geological Survey Professional Paper 1807, 140 pp. Accessed March 31, 2021 from https://pubs.usgs.gov/pp/1807/pdf/pp1807.pdf	Brooks, H. K. (1981). Physiographic divisions of Florida. Report for the Florida Cooperative Extension Service Institute of Food and Agricultural Sciences, University of Florida, 12 pp.	-	Approximated from physiographic provinces of Florida outlined by Brooks (1981). Broader Florida Aquifer System from Williams and Kuniansky (2016).
Tifton Upland, Floridan Aquifer System	Davis, H. (1996). Hydrogeologic Investigation and Simulation of Ground-Water Flow in the Upper Floridan Aquifer of North-Central Florida and Southwestern Georgia and Delineation of Contributing Areas for Selected City of Tallahassee, Florida, Water-Supply Wells. U.S. Geological Survey Water-Resources investigations Report 95-4296, 61 pp. Accessed March 31, 2021 from https://fl.water.usgs.gov/PDF_files/wri95_4296_davis.pdf	Clark, W. Z., Zisa, A. C. (1976) Physiographic map of Georgia: Georgia Department of Natural Resources, 1 sheet. Accessed March 31, 2021 from https://epd.georgia.gov/document/publication/sm-4-	Brooks, H. K. (1981). Physiographic divisions of Florida. Report for the Florida Cooperative Extension Service Institute of Food and Agricultural Sciences, University of Florida, 12 pp.	Approximated from Fig. 2 of Davis (1996) and the physiographic provinces for Georgia and Florida by Clark and Zisa (1976) and Brooks (1981)

Aquifer	Reference 1	Reference 2	Reference 3	Steps taken to delineate
		physiographic-map-georgia-12000000-1988/download		
Upper Coastal Plain, Floridan Aquifer System	Williams, L.J., Kuniansky, E.L. (2016). Revised hydrogeologic framework of the Floridan aquifer system in Florida and parts of Georgia, Alabama, and South Carolina. U.S. Geological Survey Professional Paper 1807, 140 pp. Accessed March 31, 2021 from https://pubs.usgs.gov/pp/1807/pdf/pp1807.pdf	Aucott, W.R. (1996). Hydrology of the Southeastern Coastal Plain Aquifer System in South Carolina and Parts of Georgia and North Carolina. U.S Geological Survey Professional Paper 1410-E, 95 pp. Accessed March 31, 2021 from https://pubs.usgs.gov/pp/1410e/report.pdf	-	Broader Florida Aquifer System from Williams and Kuniansky (2016). Approximate divide between the Upper and Lower Coastal Plain from Fig. 1 of Aucott (1996).
Vidalia Upland, Floridan Aquifer System	Williams, L.J., Kuniansky, E.L. (2016). Revised hydrogeologic framework of the Floridan aquifer system in Florida and parts of Georgia, Alabama, and South Carolina. U.S. Geological Survey Professional Paper 1807, 140 pp. Accessed March 31, 2021 from https://pubs.usgs.gov/pp/1807/pdf/pp1807.pdf	Clark, W. Z., Zisa, A. C. (1976) Physiographic map of Georgia: Georgia Department of Natural Resources, 1 sheet. Accessed March 31, 2021 from https://epd.georgia.gov/document/publication/sm-4-physiographic-map-georgia-12000000-1988/download	-	Approximated from physiographic provinces of Georgia outlined by Clark and Zisa (1976). Broader Florida Aquifer System from Williams and Kuniansky (2016). Northeastern boundary set along the Savannah River.
Alabama Coastal Lowlands, Gulf Coast Regional Aquifer System	Mallory, M. J. (1993). Hydrogeology of the Southeastern Coastal Plain aquifer system in parts of eastern Mississippi and western Alabama. U.S. Geological Survey Professional Paper 1410-G, 66 pp. Accessed March 31, 2021 from https://pubs.usgs.gov/pp/1410g/report.pdf	Trapp Jr., H. (1973). Hydrology of sand-and-gravel aquifer in central and southern Escambia County, Florida. U.S. Geological Survey Open-File Report 74-218, 69 pp. Accessed March 31, 2021 from https://pubs.usgs.gov/of/1974/0218/report.pdf	Pratt, T. R., Richards, C. J., Milla, K. A., Wagner, J. R., Johnson, J. L., Curry, R. J. (1996). Hydrogeology of the Northwest Florida Water Management District. Northwest Florida Water Management District, Water Resources Special Report, 96-4.	Eastern margin approximated along the Conecuh and Escambia Rivers. This area was not included in Floridan Aquifer System, as some local hydrogeological reports in its eastern margins (e.g. Cantonment in northwest Florida) suggest the Floridan aquifer itself may be quite deep as "(t)he Pensacola Clay has been reached by only a few wells in the area" (quote from Trapp (1973)), and the Pensacola Clay overlies what has been deemed the "Floridan Aquifer System" (see Fig. 1 of Pratt et al. (1996)). The subarea contains an area that has been delineated as being part of both the Floridan and the Gulf Coastal Plain (see overlapping areas mapped in Fig. 1 of Mallory (1993)). Fig. 8 of Barraclough and Marsh (1962) also shows that the Upper Floridan Aquifer is more than 300 m deep in Baldwin County (Alabama). as the primary units of the Floridan do not immediately appear to be widely accessed, the subarea was included in the Gulf Coast Regional Aquifer rather than the Floridan Aquifer System.
Catahoula Area, Gulf Coast Regional Aquifer System	Weiss, J. S. (1990). Geohydrologic units of the coastal lowlands aquifer system, south-central United States. U.S. Geological Survey Regional aquifer-systems analysis. 42 pp. Accessed March 30, 2021 from https://pubs.usgs.gov/pp/1416c/report.pdf	Halford, K. J., Barber, N. L. (1995). Analysis of ground-water flow in the Catahoula aquifer system in the vicinity of Laurel and Hattiesburg, Mississippi. U.S. Geological Survey Water-Resources	Kuniansky, E. L., Bellino, J. C., Dixon, J. (2012). Transmissivity of the upper Floridan aquifer in Florida and parts of Georgia, South Carolina, and Alabama. U.S. Geological Survey Scientific	Northern margin of Gulf Coast Regional Aquifer System approximated from Fig. 2 of Weiss (1990). Hydrostratigraphy for central portion of subarea detailed by Halford and Barber (1995). Eastern margin approximated along portions of the Chickasawhay River.

Aquifer	Reference 1	Reference 2	Reference 3	Steps taken to delineate
		Investigations Report 94-4219, 78 pp. Accessed March 31, 2021 from https://pubs.usgs.gov/wri/1994/4219/report.pdf	Investigations Map 3204, 1 sheet, scale 1:100,000, Accessed March 31, 2021 from https://pubs.usgs.gov/sim/3204/pdf/USGS_SIM-3204_Kuniansky_Web.pdf	
Gonzales-New Orleans Aquifer, Gulf Coast Regional Aquifer System	Weiss, J. S. (1990). Geohydrologic units of the coastal lowlands aquifer system, south-central United States. U.S. Geological Survey Regional aquifer-systems analysis. 42 pp. Accessed March 30, 2021 from https://pubs.usgs.gov/pp/1416c/report.pdf	Louisiana Department of Transportation and Development (2014a). Water Resources of St. John the Baptist Parish, Louisiana. U.S. Geological Survey Fact Sheet 2014–3102, 6 pp. Accessed March 30, 2021 from https://pubs.usgs.gov/fs/2014/3102/pdf/fs2014-3102.pdf	Louisiana Department of Transportation and Development (2014b). Water Resources of Orleans Parish, Louisiana. U.S. Geological Survey Fact Sheet 2014–3017, 6 pp. Accessed March 30, 2021 from https://pubs.usgs.gov/fs/2014/3017/pdf/fs2014-3017.pdf	Outer margin of Gulf Coast Regional Aquifer System approximated from Fig. 2 of Weiss (1990). Detailed study site information for a portion of the subarea available from Louisiana Department of Transportation and Development (2014a, b)
Houston-Galveston Area, Gulf Coast Regional Aquifer System	Weiss, J. S. (1990). Geohydrologic units of the coastal lowlands aquifer system, south-central United States. U.S. Geological Survey Regional aquifer-systems analysis. 42 pp. Accessed March 30, 2021 from https://pubs.usgs.gov/pp/1416c/report.pdf	Braun, C.L., Ramage, J.K., Shah, S.D. (2019). Status of groundwater-level altitudes and long-term groundwater-level changes in the Chicot, Evangeline, and Jasper aquifers, Houston-Galveston region, Texas, 2019: U.S. Geological Survey Scientific Investigations Report 2019–5089, 18 pp. https://pubs.usgs.gov/sir/2019/5089/sir20195089.pdf	-	Outer margin of Gulf Coast Regional Aquifer System approximated from Fig. 2 of Weiss (1990).
Lafayette Area, Gulf Coast Regional Aquifer System	Weiss, J. S. (1990). Geohydrologic units of the coastal lowlands aquifer system, south-central United States. U.S. Geological Survey Regional aquifer-systems analysis. 42 pp. Accessed March 30, 2021 from https://pubs.usgs.gov/pp/1416c/report.pdf	Louisiana Department of Transportation and Development (2011). Water Resources of Lafayette Parish. U.S. Geological Survey Fact Sheet 2010–3048, 6 pp. Accessed March 30, 2021 from https://pubs.usgs.gov/fs/2010/3048/pdf/FS2010-3048.pdf	-	Outer margin of Gulf Coast Regional Aquifer System approximated from Fig. 2 of Weiss (1990). Detailed study site information for a portion of the subarea available from Louisiana Department of Transportation and Development (2011)
Mississippi Delta, Gulf Coast Regional Aquifer System	Weiss, J. S. (1990). Geohydrologic units of the coastal lowlands aquifer system, south-central United States. U.S. Geological Survey Regional aquifer-systems analysis. 42 pp. Accessed March 30, 2021 from https://pubs.usgs.gov/pp/1416c/report.pdf	Torak, L.J., Painter, J.A. (2019). Geostatistical estimation of the bottom altitude and thickness of the Mississippi River Valley alluvial aquifer: U.S. Geological Survey Scientific Investigations Map 3426, 2 sheets, Accessed March 31, 2021 from https://pubs.er.usgs.gov/publication/sim3426.	-	Outer margin of Gulf Coast Regional Aquifer System approximated from Fig. 2 of Weiss (1990). Delta subarea delineated southeast of Lafayette Area, south of deep wells (from completion reports) near Baton Rouge, and south of Gonzales-New Orleans aquifer area. Torak and Painter (2019) provide helpful alluvial thickness data for the delta region and also the Mississippi River Valley Alluvial Aquifer extending north of this delineated subregion (connecting to the Central Mississippi Embayment); alluvium in this delineated area can be up to 120 m thick (Torak and Painter, 2019).

Aquifer	Reference 1	Reference 2	Reference 3	Steps taken to delineate
Rio Grande Delta, Gulf Coast Regional Aquifer System	Weiss, J. S. (1990). Geohydrologic units of the coastal lowlands aquifer system, south-central United States. U.S. Geological Survey Regional aquifer-systems analysis. 42 pp. Accessed March 30, 2021 from https://pubs.usgs.gov/pp/1416c/report.pdf	-	-	Outer margin of Gulf Coast Regional Aquifer System approximated from Fig. 2 of Weiss (1990). Mexico portion of aquifer system delineated based on well completion data and topography.
Southern Hills, Gulf Coast Regional Aquifer System	Weiss, J. S. (1990). Geohydrologic units of the coastal lowlands aquifer system, south-central United States. U.S. Geological Survey Regional aquifer-systems analysis. 42 pp. Accessed March 30, 2021 from https://pubs.usgs.gov/pp/1416c/report.pdf	Buono, A. (1983). The Southern Hills Regional Aquifer System of Southeastern Louisiana And Southwestern Mississippi. U.S. Geological Survey Water-Resources Investigations Report 83-4189, 44 pp. Accessed March 31, 2021 from https://pubs.usgs.gov/wri/1983/4189/report.pdf	Boswell, E.H. (1979). The Citronelle aquifers in Mississippi. U.S. Geological Survey Water-Resources Investigations Report 78-131, 1 plate. Accessed March 31, 2021 from https://pubs.usgs.gov/wri/1978/0131/plate-1.pdf	Margin of Gulf Coast Regional Aquifer System approximated from Fig. 2 of Weiss (1990); northwestern boundary is Central Mississippi Embayment. Eastern border approximated from Figs. 1 and 2 of Buono (1983) and the Pearl River. Shallow aquifer ("Citronelle aquifers") information from Boswell (1979).
Victoria Area, Gulf Coast Regional Aquifer System	Weiss, J. S. (1990). Geohydrologic units of the coastal lowlands aquifer system, south-central United States. U.S. Geological Survey Regional aquifer-systems analysis. 42 pp. Accessed March 30, 2021 from https://pubs.usgs.gov/pp/1416c/report.pdf	Marvin, R.F., Shafer, G.H., Dale, O.C. (1962). Groundwater resources of Victoria and Calhoun Counties, Texas. Accessed March 30, 2021 from https://www.twdb.texas.gov/publications/reports/bulletins/doc/Bull.htm/B6202.asp	-	Outer margin of Gulf Coast Regional Aquifer System approximated from Fig. 2 of Weiss (1990). Delineation of central portion of subarea (and regional geology) aided by plate 1a of Marvin et al. (1962). Western margin of subarea approximated along the Nueses River.
Central High Plains, High Plains	Gutentag, E. D., Heimes, F. J., Krothe, N. C., Luckey, R. R., Weeks, J. B. (1984). Geohydrology of the High Plains aquifer in parts of Colorado, Kansas, Nebraska, New Mexico, Oklahoma, South Dakota, Texas, and Wyoming (No. 1400-B). Accessed February 10, 2021 from https://pubs.usgs.gov/pp/1400b/report.pdf	Luckey, R.L., Becker, M.F. (2003). Hydrogeology, water use, and simulation of flow in the High Plains aquifer in northwestern Oklahoma, southeastern Colorado, southwestern Kansas, northeastern New Mexico, and northwestern Texas. Water-Resources Investigations Report 99-4104, 73 pp. Accessed February 15, 2022 from https://pubs.usgs.gov/wri/wri994104/pdf/wri994104.pdf	McGuire, V.L., Johnson, M.R., Schieffer, J.S., Stanton, J.S., Sebree, S.K., Varstraeten (2003). Water in storage and approaches to groundwater management, High Plains Aquifer, 2000. US Geological Survey Circular 1243, 56 pp. Accessed February 15, 2022 from https://pubs.usgs.gov/circ/2003/circ1243/pdf/C1243.pdf	Approximated from Fig. 1 of Gutentag et al. (1984), Fig. 18 by McGuire et al. (2003), and Fig. 1 by Luckey and Becker (2003)
Equus Beds, High Plains	Gutentag, E. D., Heimes, F. J., Krothe, N. C., Luckey, R. R., Weeks, J. B. (1984). Geohydrology of the High Plains aquifer in parts of Colorado, Kansas, Nebraska, New Mexico, Oklahoma, South Dakota, Texas, and Wyoming (No. 1400-B). Accessed February 10, 2021 from https://pubs.usgs.gov/pp/1400b/report.pdf	Kansas Geological Survey (2021). Webpage entitled "High Plains Aquifer Regions in Kansas" (Kansas High Plains Aquifer Atlas). Accessed April 15, 2021 from https://geokansas.ku.edu/kansas-high-plains-aquifer-atlas)	-	Approximated from Fig. 1 of Gutentag et al. (1984). Great Bend Prairie area approximated from the "High Plains Aquifer Regions in Kansas" (Kansas High Plains Aquifer Atlas) accessed April 15, 2021 from the Kansas Geological Survey (2021; https://geokansas.ku.edu/kansas-high-plains-aquifer-atlas)
Great Bend Prairie, High Plains	Gutentag, E. D., Heimes, F. J., Krothe, N. C., Luckey, R. R., Weeks, J. B. (1984). Geohydrology of the High Plains aquifer in parts of Colorado, Kansas, Nebraska, New Mexico, Oklahoma, South Dakota, Texas, and Wyoming (No. 1400-B). Accessed February 10, 2021 from https://pubs.usgs.gov/pp/1400b/report.pdf	Kansas Geological Survey (2021). Webpage entitled "High Plains Aquifer Regions in Kansas" (Kansas High Plains Aquifer Atlas). Accessed April 15, 2021 from	-	Approximated from Fig. 1 of Gutentag et al. (1984). Great Bend Prairie area approximated from the "High Plains Aquifer Regions in Kansas" (Kansas High Plains Aquifer Atlas) accessed April 15, 2021 from the Kansas Geological Survey (2021;

Aquifer	Reference 1	Reference 2	Reference 3	Steps taken to delineate
		https://geokansas.ku.edu/kansas-high-plains-aquifer-atlas)		https://geokansas.ku.edu/kansas-high-plains-aquifer-atlas)
Northern High Plains, High Plains	Peterson, S. M., Traylor, J. P., Guira, M. (2020). Groundwater Availability of the Northern High Plains Aquifer in Colorado, Kansas, Nebraska, South Dakota, and Wyoming. U.S. Geological Survey Professional Paper 1864, 57 p., Accessed November 28, 2021 from https://pubs.usgs.gov/pp/1864/pp1864.pdf	Miller, J. A., Appel, C. L. (1997a). Ground Water Atlas of the United States: Kansas, Missouri, and Nebraska HA. United States Geological Survey Report 730-D. Accessed February 10, 2021 from https://pubs.usgs.gov/ha/ha730/ch_d/.	Gutentag, E. D., Heimes, F. J., Krothe, N. C., Luckey, R. R., Weeks, J. B. (1984). Geohydrology of the High Plains aquifer in parts of Colorado, Kansas, Nebraska, New Mexico, Oklahoma, South Dakota, Texas, and Wyoming (No. 1400-B). Accessed February 10, 2021 from https://pubs.usgs.gov/pp/1400b/report.pdf	Approximated from Fig. 7 of Peterson et al. (2020), Fig. 46 by Miller and Appel (1997a), and Fig. 1 by Gutentag et al. (1984)
Southern High Plains, High Plains	Gutentag, E. D., Heimes, F. J., Krothe, N. C., Luckey, R. R., Weeks, J. B. (1984). Geohydrology of the High Plains aquifer in parts of Colorado, Kansas, Nebraska, New Mexico, Oklahoma, South Dakota, Texas, and Wyoming (No. 1400-B). Accessed February 10, 2021 from https://pubs.usgs.gov/pp/1400b/report.pdf	Deeds, N.E., Harding, J.J., Jones, T.L., Singh, A., Hamlin, S., Reedy, R.C., Yan, T., Jigmond, M., Lupton, D., Scanlon, B.R., Seni, S., Dutton, A. (2015). Final Conceptual Model Report for the High Plains Aquifer System Groundwater Availability Model. Texas Water Development Board Report, 590 pp. Accessed February 15, 2022 from https://www.twdb.texas.gov/groundwater/models/gam/hpas/HPAS_GAM_Conceptual_Report.pdf	-	Approximated from Fig. 1 of Gutentag et al. (1984) and Fig. 2.0.1 by Deeds et al. (2015).
Crawford Upland, Interior Low Plateaus	Miller, J.A. (1995). Ground Water Atlas of the United States: Segment 10, Illinois, Indiana, Kentucky, Ohio, Tennessee. U.S. Geological Survey Hydrologic Atlas 730-K, 30 pp. Accessed April 11, 2021 from https://pubs.usgs.gov/ha/730k/report.pdf	Taylor, C.J., Nelson Jr., H.L. (2008). A compilation of provisional karst geospatial data for the Interior Low Plateaus physiographic region, central United States: U.S. Geological Survey Data Series 339, 26 pp. Accessed April 11, 2021 from https://pubs.usgs.gov/ds/339/pdf/ds339_web.pdf	Fenelon, J.M., Bobay, K.E., Greeman, T.K., Hoover, M.E., Cohen, D.A., Fowler, K.K., Woodfield, M.C., Durbin, J.M. (1994). Hydrogeologic atlas of aquifers in Indiana: U.S. Geological Survey Water-Resources Investigations Report 1992–4142, 197 pp. Accessed April 11, 2021 from https://pubs.er.usgs.gov/publication/wri924142	Broader Interior Low Plateaus outline approximated for Tennessee, Illinois, Kentucky, Indiana from Fig. 78 of Miller (1990) and Fig. 1 of Taylor and Nelson (2008). Crawford Upland approximated from Fig. 2 of Fenelon et al. (1994).
Eastern Highland Rim, Interior Low Plateaus	Miller, J.A. (1995). Ground Water Atlas of the United States: Segment 10, Illinois, Indiana, Kentucky, Ohio, Tennessee. U.S. Geological Survey Hydrologic Atlas 730-K, 30 pp. Accessed April 11, 2021 from https://pubs.usgs.gov/ha/730k/report.pdf	Brahana, J.V., Bradley, M.W. (1986b). Preliminary delineation and description of the regional aquifers of Tennessee--The Highland Rim Aquifer System. Water-Resources Investigations Report 82-4054, 35 pp. Accessed April 11, 2021 from	Taylor, C.J., Nelson Jr., H.L. (2008). A compilation of provisional karst geospatial data for the Interior Low Plateaus physiographic region, central United States: U.S. Geological Survey Data Series 339, 26 pp. Accessed	Broader Interior Low Plateaus outline approximated for Tennessee, Illinois, Kentucky, Indiana from Fig. 78 of Miller (1990) and Fig. 1 of Taylor and Nelson (2008). Highland Rim subarea approximated from Fig. 1 of Taylor and Nelson (2008) and Fig. 1 of Brahana and Bradley (1986b)

Aquifer	Reference 1	Reference 2	Reference 3	Steps taken to delineate
		https://pubs.usgs.gov/wri/wri824054/pdf/wrir_82-4054_a.pdf	April 11, 2021 from https://pubs.usgs.gov/ds/339/pdf/ds339_web.pdf	
Eastern Outer Bluegrass, Interior Low Plateaus	Miller, J.A. (1995). Ground Water Atlas of the United States: Segment 10, Illinois, Indiana, Kentucky, Ohio, Tennessee. U.S. Geological Survey Hydrologic Atlas 730-K, 30 pp. Accessed April 11, 2021 from https://pubs.usgs.gov/ha/730k/report.pdf	Taylor, C.J., Nelson Jr., H.L. (2008). A compilation of provisional karst geospatial data for the Interior Low Plateaus physiographic region, central United States: U.S. Geological Survey Data Series 339, 26 pp. Accessed April 11, 2021 from https://pubs.usgs.gov/ds/339/pdf/ds339_web.pdf	Davidson, B. (2018). Kentucky Interagency Groundwater Monitoring Network: Annual Report July 2017–June 2018. 28 pp. Accessed April 11, 2021 from http://www.uky.edu/KGS/water/gnet/itac17-18.pdf	Broader Interior Low Plateaus outline approximated for Tennessee, Illinois, Kentucky, Indiana from Fig. 78 of Miller (1990) and Fig. 1 of Taylor and Nelson (2008). Outer Bluegrass approximated from Fig. 1 of Davidson (2018).
Eastern Pennyroyal Plateau, Interior Low Plateaus	Miller, J.A. (1995). Ground Water Atlas of the United States: Segment 10, Illinois, Indiana, Kentucky, Ohio, Tennessee. U.S. Geological Survey Hydrologic Atlas 730-K, 30 pp. Accessed April 11, 2021 from https://pubs.usgs.gov/ha/730k/report.pdf	Taylor, C.J., Nelson Jr., H.L. (2008). A compilation of provisional karst geospatial data for the Interior Low Plateaus physiographic region, central United States: U.S. Geological Survey Data Series 339, 26 pp. Accessed April 11, 2021 from https://pubs.usgs.gov/ds/339/pdf/ds339_web.pdf	Davidson, B. (2018). Kentucky Interagency Groundwater Monitoring Network: Annual Report July 2017–June 2018. 28 pp. Accessed April 11, 2021 from http://www.uky.edu/KGS/water/gnet/itac17-18.pdf	Broader Interior Low Plateaus outline approximated for Tennessee, Illinois, Kentucky, Indiana from Fig. 78 of Miller (1990) and Fig. 1 of Taylor and Nelson (2008). Pennyroyal Plain approximated from Fig. 1 of Davidson (2018); northern limit approximated along the Salt River and Ohio River.
Inner Bluegrass, Interior Low Plateaus	Miller, J.A. (1995). Ground Water Atlas of the United States: Segment 10, Illinois, Indiana, Kentucky, Ohio, Tennessee. U.S. Geological Survey Hydrologic Atlas 730-K, 30 pp. Accessed April 11, 2021 from https://pubs.usgs.gov/ha/730k/report.pdf	Taylor, C.J., Nelson Jr., H.L. (2008). A compilation of provisional karst geospatial data for the Interior Low Plateaus physiographic region, central United States: U.S. Geological Survey Data Series 339, 26 pp. Accessed April 11, 2021 from https://pubs.usgs.gov/ds/339/pdf/ds339_web.pdf	Scanlon, B. R. (1989). Physical Controls on Hydrochemical Variability in the Inner Bluegrass Karst Region of Central Kentucky. Groundwater, 27, 639-646.	Broader Interior Low Plateaus outline approximated for Tennessee, Illinois, Kentucky, Indiana from Fig. 78 of Miller (1990) and Fig. 1 of Taylor and Nelson (2008). Inner Bluegrass area approximated from Fig. 1 of Scanlon (1989).
Mitchell Plateau, Interior Low Plateaus	Miller, J.A. (1995). Ground Water Atlas of the United States: Segment 10, Illinois, Indiana, Kentucky, Ohio, Tennessee. U.S. Geological Survey Hydrologic Atlas 730-K, 30 pp. Accessed April 11, 2021 from https://pubs.usgs.gov/ha/730k/report.pdf	Taylor, C.J., Nelson Jr., H.L. (2008). A compilation of provisional karst geospatial data for the Interior Low Plateaus physiographic region, central United States: U.S. Geological Survey Data Series 339, 26 pp. Accessed April 11, 2021 from https://pubs.usgs.gov/ds/339/pdf/ds339_web.pdf	Florea, L. J., Hasenmueller, N. R., Branam, T. D., Frushour, S. S., Powell, R. L. (2018). Karst geology and hydrogeology of the Mitchell Plateau of south-central Indiana. In: Field Guide 51, Published by The Geological Society of America, pp. 95-112.	Broader Interior Low Plateaus outline approximated for Tennessee, Illinois, Kentucky, Indiana from Fig. 78 of Miller (1990) and Fig. 1 of Taylor and Nelson (2008). Mitchell Plain subarea approximated from Fig. 1 of Florea et al. (2008).
Moulton Valley, Interior Low Plateaus	Geological Survey of Alabama (2018). Assessment of groundwater resources in Alabama, 2010-16. Geological Survey of Alabama Bulletin 186, 462 pp. Accessed April 8, 2021 from https://www.gsa.state.al.us/img/Groundwater/docs/assessment/00_B186_StatewideAssessment_Print_Document.pdf	Miller, J.A. (1990). Ground Water Atlas of the United States: Segment 6, Alabama, Florida, Georgia, South Carolina. U.S. Geological Survey Hydrologic Atlas 730-G, 30 pp. Accessed April 5, 2021 from https://www.nrc.gov/docs/ML1706/ML17060B027.pdf	-	Moulton Valley subarea approximated from Fig. 4 of Geological Survey of Alabama (2008) and Fig. 104 of Miller (1990). Broader Interior Low Plateaus outline approximated for Alabama from Fig. 4 of Geological Survey of Alabama (2008) and Fig. 104 of Miller (1990).

Aquifer	Reference 1	Reference 2	Reference 3	Steps taken to delineate
Nashville Basin, Interior Low Plateaus	Miller, J.A. (1995). Ground Water Atlas of the United States: Segment 10, Illinois, Indiana, Kentucky, Ohio, Tennessee. U.S. Geological Survey Hydrologic Atlas 730-K, 30 pp. Accessed April 11, 2021 from https://pubs.usgs.gov/ha/730k/report.pdf	Brahana, J.V., Bradley, M.W. (1986a). Preliminary delineation and description of the regional aquifers of Tennessee: The Central Basin Aquifer System. U.S. Geological Survey Water-Resources Investigations Report 82-4002, 40 pp. Accessed April 11, 2021 from https://pubs.usgs.gov/wri/wrir82-4002/pdf/wrir_82-4002_a.pdf	Taylor, C.J., Nelson Jr., H.L. (2008). A compilation of provisional karst geospatial data for the Interior Low Plateaus physiographic region, central United States: U.S. Geological Survey Data Series 339, 26 pp. Accessed April 11, 2021 from https://pubs.usgs.gov/ds/339/pdf/ds339_web.pdf	Broader Interior Low Plateaus outline approximated for Tennessee, Illinois, Kentucky, Indiana from Fig. 78 of Miller (1990) and Fig. 1 of Taylor and Nelson (2008). "Central Basin" and "Nashville Basin" (seemingly similar areas) approximated from Fig. 1 of Taylor and Nelson (2008) and Fig. 1 of Brahana and Bradley (1986a)
Norman Upland, Interior Low Plateaus	Miller, J.A. (1995). Ground Water Atlas of the United States: Segment 10, Illinois, Indiana, Kentucky, Ohio, Tennessee. U.S. Geological Survey Hydrologic Atlas 730-K, 30 pp. Accessed April 11, 2021 from https://pubs.usgs.gov/ha/730k/report.pdf	Taylor, C.J., Nelson Jr., H.L. (2008). A compilation of provisional karst geospatial data for the Interior Low Plateaus physiographic region, central United States: U.S. Geological Survey Data Series 339, 26 pp. Accessed April 11, 2021 from https://pubs.usgs.gov/ds/339/pdf/ds339_web.pdf	Fenelon, J.M., Bobay, K.E., Greeman, T.K., Hoover, M.E., Cohen, D.A., Fowler, K.K., Woodfield, M.C., Durbin, J.M. (1994). Hydrogeologic atlas of aquifers in Indiana: U.S. Geological Survey Water-Resources Investigations Report 1992–4142, 197 pp. Accessed April 11, 2021 from https://pubs.er.usgs.gov/publication/wri924142	Broader Interior Low Plateaus outline approximated for Tennessee, Illinois, Kentucky, Indiana from Fig. 78 of Miller (1990) and Fig. 1 of Taylor and Nelson (2008). Norman Upland approximated from Fig. 2 of Fenelon et al. (1994).
Shawnee Hills, Interior Low Plateaus	Miller, J.A. (1995). Ground Water Atlas of the United States: Segment 10, Illinois, Indiana, Kentucky, Ohio, Tennessee. U.S. Geological Survey Hydrologic Atlas 730-K, 30 pp. Accessed April 11, 2021 from https://pubs.usgs.gov/ha/730k/report.pdf	Taylor, C.J., Nelson Jr., H.L. (2008). A compilation of provisional karst geospatial data for the Interior Low Plateaus physiographic region, central United States: U.S. Geological Survey Data Series 339, 26 pp. Accessed April 11, 2021 from https://pubs.usgs.gov/ds/339/pdf/ds339_web.pdf	Leighton, M. M., Ekblaw, G. E., Horberg, L. (1948). Physiographic divisions of Illinois. The Journal of Geology, 56, 16-33.	Broader Interior Low Plateaus outline approximated for Tennessee, Illinois, Kentucky, Indiana from Fig. 78 of Miller (1990) and Fig. 1 of Taylor and Nelson (2008). Shawnee Hills approximated from Figs. 1 and 2 of Leighton et al. (1948)
Southern Highland Rim, Interior Low Plateaus	Miller, J.A. (1995). Ground Water Atlas of the United States: Segment 10, Illinois, Indiana, Kentucky, Ohio, Tennessee. U.S. Geological Survey Hydrologic Atlas 730-K, 30 pp. Accessed April 11, 2021 from https://pubs.usgs.gov/ha/730k/report.pdf	Brahana, J.V., Bradley, M.W. (1986b). Preliminary delineation and description of the regional aquifers of Tennessee--The Highland Rim Aquifer System. Water-Resources Investigations Report 82-4054, 35 pp. Accessed April 11, 2021 from https://pubs.usgs.gov/wri/wri824054/pdf/wrir_82-4054_a.pdf	Taylor, C.J., Nelson Jr., H.L. (2008). A compilation of provisional karst geospatial data for the Interior Low Plateaus physiographic region, central United States: U.S. Geological Survey Data Series 339, 26 pp. Accessed April 11, 2021 from https://pubs.usgs.gov/ds/339/pdf/ds339_web.pdf	Broader Interior Low Plateaus outline approximated for Tennessee, Illinois, Kentucky, Indiana from Fig. 78 of Miller (1990) and Fig. 1 of Taylor and Nelson (2008). Highland Rim subarea approximated from Fig. 1 of Taylor and Nelson (2008) and Fig. 1 of Brahana and Bradley (1986b)
Western Highland Rim, Interior Low Plateaus	Miller, J.A. (1995). Ground Water Atlas of the United States: Segment 10, Illinois, Indiana, Kentucky, Ohio, Tennessee. U.S. Geological Survey Hydrologic Atlas 730-K, 30 pp. Accessed April 11, 2021 from https://pubs.usgs.gov/ha/730k/report.pdf	Brahana, J.V., Bradley, M.W. (1986b). Preliminary delineation and description of the regional aquifers of Tennessee--The Highland Rim Aquifer System. Water-Resources Investigations Report 82-4054, 35 pp.	Taylor, C.J., Nelson Jr., H.L. (2008). A compilation of provisional karst geospatial data for the Interior Low Plateaus physiographic region, central United States: U.S. Geological Survey Data	Broader Interior Low Plateaus outline approximated for Tennessee, Illinois, Kentucky, Indiana from Fig. 78 of Miller (1990) and Fig. 1 of Taylor and Nelson (2008). Highland Rim subarea approximated from Fig. 1 of Taylor and Nelson (2008) and Fig. 1 of

Aquifer	Reference 1	Reference 2	Reference 3	Steps taken to delineate
		Accessed April 11, 2021 from https://pubs.usgs.gov/wri/wri824054/pdf/wrir_82-4054_a.pdf	Series 339, 26 pp. Accessed April 11, 2021 from https://pubs.usgs.gov/ds/339/pdf/ds339_web.pdf	Brahana and Bradley (1986b). Northern limit approximated along the Cumberland River.
Western Outer Bluegrass, Interior Low Plateaus	Miller, J.A. (1995). Ground Water Atlas of the United States: Segment 10, Illinois, Indiana, Kentucky, Ohio, Tennessee. U.S. Geological Survey Hydrologic Atlas 730-K, 30 pp. Accessed April 11, 2021 from https://pubs.usgs.gov/ha/730k/report.pdf	Taylor, C.J., Nelson Jr., H.L. (2008). A compilation of provisional karst geospatial data for the Interior Low Plateaus physiographic region, central United States: U.S. Geological Survey Data Series 339, 26 pp. Accessed April 11, 2021 from https://pubs.usgs.gov/ds/339/pdf/ds339_web.pdf	Davidson, B. (2018). Kentucky Interagency Groundwater Monitoring Network: Annual Report July 2017–June 2018. 28 pp. Accessed April 11, 2021 from http://www.uky.edu/KGS/water/gnet/itac17-18.pdf	Broader Interior Low Plateaus outline approximated for Tennessee, Illinois, Kentucky, Indiana from Fig. 78 of Miller (1990) and Fig. 1 of Taylor and Nelson (2008). Outer Bluegrass approximated from Fig. 1 of Davidson (2018).
Western Pennyroyal Plateau, Interior Low Plateaus	Miller, J.A. (1995). Ground Water Atlas of the United States: Segment 10, Illinois, Indiana, Kentucky, Ohio, Tennessee. U.S. Geological Survey Hydrologic Atlas 730-K, 30 pp. Accessed April 11, 2021 from https://pubs.usgs.gov/ha/730k/report.pdf	Taylor, C.J., Nelson Jr., H.L. (2008). A compilation of provisional karst geospatial data for the Interior Low Plateaus physiographic region, central United States: U.S. Geological Survey Data Series 339, 26 pp. Accessed April 11, 2021 from https://pubs.usgs.gov/ds/339/pdf/ds339_web.pdf	Davidson, B. (2018). Kentucky Interagency Groundwater Monitoring Network: Annual Report July 2017–June 2018. 28 pp. Accessed April 11, 2021 from http://www.uky.edu/KGS/water/gnet/itac17-18.pdf	Broader Interior Low Plateaus outline approximated for Tennessee, Illinois, Kentucky, Indiana from Fig. 78 of Miller (1990) and Fig. 1 of Taylor and Nelson (2008). Pennyroyal Plain approximated from Fig. 1 of Davidson (2018); divide between western and eastern portions approximated along Salt Lick Creek and Barren River
Lolo-Bitterroot Valley, Lolo-Bitterroot-Missoula Valleys	Briar, D. W., Dutton, D. M. (1999). Hydrogeology and Aquifer Sensitivity of the Bitterroot Valley, Ravalli County, Montana. Water-Resources Investigations Report, 99-4219. Accessed March 2, 2021 from https://pubs.usgs.gov/wri/1999/4219/report.pdf	Smith, L. N., LaFave, J., I., Patton, T. W. (2013). Groundwater resources of the Lolo-Bitterroot area: Mineral, Missoula, and Ravalli Counties, Montana. Montana Bureau of Mines and Geology. Montana Groundwater Assessment Atlas No. 4. Accessed March 2, 2021 from http://www.mbmg.mtech.edu/pdf-publications/gwaa4a.pdf	Smith, L. N. (2006). Hydrologic framework of the Lolo-Bitterroot Area ground-water characterization study : Montana Bureau of Mines and Geology Montana Ground-Water Assessment Atlas 4-B-02, 1 sheet, scale 1:250,000. Accessed March 2, 2021 from http://mbmg.mtech.edu/pdf-publications/GWAA04B-02.pdf	Approximated from Fig. 1 of Birar and Dutton (1999), Fig. 2 of Smith et al. (2003) and map by Smith (2006).
Missoula Valley, Lolo-Bitterroot-Missoula Valleys	Smith, L. N., LaFave, J., I., Patton, T. W. (2013). Groundwater resources of the Lolo-Bitterroot area: Mineral, Missoula, and Ravalli Counties, Montana. Montana Bureau of Mines and Geology. Montana Groundwater Assessment Atlas No. 4. Accessed March 2, 2021 from http://www.mbmg.mtech.edu/pdf-publications/gwaa4a.pdf	-	-	Approximated from Fig. 2 of Smith et al. (2003).
Albuquerque Basin, Middle Rio Grande	Bexfield, L. M., Anderholm, S. K. (2000). Predevelopment water-level map of the Santa Fe Group aquifer system in the middle Rio Grande basin between Cochiti Lake and San Acacia, New Mexico. U.S. Geological Survey Water-Resources Investigations Report 00–4249, 1 sheet. Accessed February 17, 2021 from https://doi.org/10.3133/wri004249	Bartolino, J.R., Cole, J.C. (2002). Ground-water resources of the Middle Rio Grande Basin. U.S. Geological Survey Water-Resources Circular 1222, 145 pp. Accessed November 28, 2021 from https://pubs.usgs.gov/circ/2002/circ1222/pdf/circ1222.pdf	Hawley, J. W., Haase, C.S., Lozinsky, R.P. (1995). An Underground View of the Albuquerque Basin. Report No. CONF-9411293-TRN: IM9704%%261. 37-55. Accessed November 28, 2021 from https://nmwrri.nmsu.edu/wp-	Approximated from map by Bexfield and Anderholm (2000), Fig. 1 by Hawley et al. (1995) and Fig. 2.1 by Bartolino and Cole (2002)

Aquifer	Reference 1	Reference 2	Reference 3	Steps taken to delineate
			content/uploads/2015/watcon/proc39/Hawley.pdf	
Espanola Basin, Middle Rio Grande	Land, L. (2016). Overview of Fresh and Brackish Water Quality in New Mexico. Open file Report 583. 4 pp. Accessed February 17, 2021 from https://geoinfo.nmt.edu/resources/water/amp/brochures/BWA/Estancia_Basin_FBWQNM.pdf	Manning, A.H. (2009). Ground-water temperature, noble gas, and carbon isotope data from the Española Basin, New Mexico: U.S. Geological Survey Scientific Investigations Report 2008–5200, 78 pp. Accessed November 28, 2021 from https://pubs.usgs.gov/sir/2008/5200/pdf/SIR08-5200.pdf	-	Approximated from map by Land (2016), Fig. 1 by Manning (2009)
San Luis Valley, Middle Rio Grande	Land, L. (2016). Overview of Fresh and Brackish Water Quality in New Mexico. Open file Report 583. 4 pp. Accessed February 17, 2021 from https://geoinfo.nmt.edu/resources/water/amp/brochures/BWA/Estancia_Basin_FBWQNM.pdf	-	-	Approximated from Map by Land (2016)
Central Mississippi Embayment, Mississippi Embayment	Arthur, J. K., Taylor, R. E. (1998). Ground-water flow analysis of the Mississippi embayment aquifer system, South-Central United States. US Geological Survey Professional Paper 1416-1, 59 pp. Accessed February 10, 2021 from https://pubs.usgs.gov/pp/1416i/report.pdf	Renken, R. A. (1998). Groundwater Atlas of the United States: Arkansas, Louisiana, Mississippi (HA-730-F). U.S. Geological Survey Hydrologic Investigations Atlas 730-F. 30 pp. Accessed November 28, 2021 fromhttps://pubs.usgs.gov/ha/730f/report.pdf	-	Approximated from Fig. 2 of Arthur and Taylor (1998) and Figs. 8-10 and Fig. 65 by Renken (1998)
Confined Claiborne Near Jackson, Mississippi Embayment	Stephenson, L. W. (1941). The ground-water resources of Mississippi. U.S. Geological Survey Water-Supply Paper 576, 540 pp. Accessed March 11, 2021 from https://pubs.usgs.gov/wsp/0576/report.pdf	-	-	Discussion on page 200 describing access to Claiborne aquifer (part of the Mississippi Embayment Aquifer System) by wells near Jackson
Eastern Mississippi Embayment, Mississippi Embayment	Arthur, J. K., Taylor, R. E. (1998). Ground-water flow analysis of the Mississippi embayment aquifer system, South-Central United States. US Geological Survey Professional Paper 1416-1, 59 pp. Accessed February 10, 2021 from https://pubs.usgs.gov/pp/1416i/report.pdf	Renken, R. A. (1998). Groundwater Atlas of the United States: Arkansas, Louisiana, Mississippi (HA-730-F). U.S. Geological Survey Hydrologic Investigations Atlas 730-F. 30 pp. Accessed November 28, 2021 fromhttps://pubs.usgs.gov/ha/730f/report.pdf	-	Approximated from Fig. 2 of Arthur and Taylor (1998) and Figs. 8-10 and Fig. 65 by Renken (1998)
Western Mississippi Embayment, Mississippi Embayment	Arthur, J. K., Taylor, R. E. (1998). Ground-water flow analysis of the Mississippi embayment aquifer system, South-Central United States. US Geological Survey Professional Paper 1416-1, 59 pp. Accessed February 10, 2021 from https://pubs.usgs.gov/pp/1416i/report.pdf	Renken, R. A. (1998). Groundwater Atlas of the United States: Arkansas, Louisiana, Mississippi (HA-730-F). U.S. Geological Survey Hydrologic Investigations Atlas 730-F. 30 pp. Accessed November 28, 2021 fromhttps://pubs.usgs.gov/ha/730f/report.pdf	-	Approximated from Fig. 2 of Arthur and Taylor (1998) and Figs. 8-10 and Fig. 65 by Renken (1998)

Aquifer	Reference 1	Reference 2	Reference 3	Steps taken to delineate
Delmarva Peninsula, North Atlantic Coastal Plain	Bachman, L.J., Shedlock, R.J., Phillips, P.J. (1987). Ground-water-quality assessment of the Delmarva Peninsula, Delaware, Maryland, and Virginia. U.S. Geological Survey Open-File Report 87-112, Accessed April 1, 2021 from https://pubs.usgs.gov/of/1987/0112/report.pdf	Sanford, W. E., Pope, J. P., Selnick, D. L., Stumvoll, R. F. (2012). Simulation of groundwater flow in the shallow aquifer system of the Delmarva Peninsula, Maryland and Delaware. US Geological Survey Open-File Report 2012–1140, 68 pp. Accessed November 29, 2021 from https://pubs.usgs.gov/of/2012/1140/pdf/OFR_2012-1140.pdf	-	Approximated from Fig. 1 of Bachman et al. (1987) and Fig. 1 by Sanford et al. (2012)
Maryland Western Shores, North Atlantic Coastal Plain	Vroblesky, D.A., Fleck, W.B. (1991). Hydrogeologic Framework of the Coastal Plain of Maryland, Delaware, and the District of Columbia. U.S. Geological Survey Professional Paper 1404-E, 52 pp. Accessed April 1, 2021 from https://pubs.usgs.gov/pp/1404e/report.pdf	-	-	Approximated from Fig. 1 of Vroblesky and Fleck (1991).
New Jersey Coastal Plain, North Atlantic Coastal Plain	Gill, H.E., Farlekas, G.M. (1976). Geohydrologic maps of the Potomac-Raritan-Magothy aquifer system in the New Jersey Coastal Plain. U.S. Geological Survey Hydrologic Atlas 557, 2 plates. Accessed April 1, 2021 from https://pubs.er.usgs.gov/publication/ha557	Gordon, A.D., Carleton, G.B., Rosman, R. (2021) Water-level conditions in the confined aquifers of the New Jersey Coastal Plain, 2013: U.S. Geological Survey Scientific Investigations Report 2019–5146, 104 p., 9 pl., Accessed April 1, 2021 from https://pubs.usgs.gov/sir/2019/5146/sir20195146.pdf	-	Approximated from Plate 1 of Gill et al. (1976) and Fig. 1 of Gordon et al. (2021).
North Carolina and Virginia Coastal Plain, North Atlantic Coastal Plain	Meng, A., Harsh, J.F. (1988). Hydrogeologic framework of the Virginia coastal plain. U.S. Geological Survey Professional Paper 1404-C, 85 pp. Accessed April 1, 2021 from https://pubs.usgs.gov/pp/pp1404-C/pdf/pp_1404-c.pdf	Winner Jr., M.D., Coble, R.W. (1989). Hydrogeologic framework of the North Carolina Coastal Plain aquifer system. U.S. Geological Survey Report 87-690, 167 pp. Accessed April 1, 2021 from https://pubs.usgs.gov/of/1987/0690/report.pdf	-	Approximated from Fig. 1 of Meng and Harsh (1988) and Fig. 1 of Winner Jr. and Coble (1989).
Powder River Basin, Northern Great Plains	Thamke, J. N., LeCain, G. D., Ryter, D. W., Sando, R., Long, A. J. (2014). Hydrogeologic framework of the uppermost principal aquifer systems in the Williston and Powder River structural basins, United States and Canada. US Geological Survey Scientific Investigations Report 2014-5047, 50 pp. Accessed February 15, 2021 from https://pubs.usgs.gov/sir/2014/5047/pdf/sir2014-5047.pdf	Long, A.J., Thamke, J.N., Davis, K.W., Bartos, T.T. (2018). Groundwater availability of the Williston Basin, United States and Canada: U.S. Geological Survey Professional Paper 1841, 54 pp. Accessed November 29, 2021 from https://pubs.usgs.gov/pp/1841/pp1841.pdf	-	Approximated from Fig. 1 of Long et al. (2018) and Fig. 1 by Thamke et al. (2014); "Miles City arch" boundary used to distinguish Williston and Powder River structural basins
Williston Basin, Northern Great Plains	Thamke, J. N., LeCain, G. D., Ryter, D. W., Sando, R., Long, A. J. (2014). Hydrogeologic framework of the uppermost principal aquifer systems in the Williston and Powder River structural basins, United States and Canada. US Geological Survey Scientific Investigations Report 2014-5047, 50 pp. Accessed February 15, 2021 from https://pubs.usgs.gov/sir/2014/5047/pdf/sir2014-5047.pdf	Long, A.J., Thamke, J.N., Davis, K.W., Bartos, T.T. (2018). Groundwater availability of the Williston Basin, United States and Canada: U.S. Geological Survey Professional Paper 1841,	-	Approximated from Fig. 1 of Long et al. (2018) and Fig. 1 by Thamke et al. (2014); "Miles City arch" boundary used to distinguish Williston and Powder River structural basins

Aquifer	Reference 1	Reference 2	Reference 3	Steps taken to delineate
		54 pp. Accessed November 29, 2021 from https://pubs.usgs.gov/pp/1841/pp1841.pdf		
Eastern Cambrian-Ordovician Aquifers, Northern Midwest Aquifer System	Young, H.L. (1992). Hydrogeology of the Cambrian-Ordovician Aquifer System in the Northern Midwest, United States. U.S. Geological Survey Professional Paper 1405-B, 108 pp. Accessed April 12, 2021 from https://pubs.usgs.gov/pp/1405b/report.pdf	Wilson, J.T. (2012). Water-quality assessment of the Cambrian-Ordovician aquifer system in the northern Midwest, United States. U.S. Geological Survey Scientific Investigations Report 2011–5229, 174 pp. Accessed April 12, 2021 from https://pubs.usgs.gov/sir/2011/5229/pdf/SIR20115229_web.pdf	-	Broader aquifer system title and its location approximated from Fig. 1 of Young (1992). Subcrop geology approximated from Fig. 9 of Wilson (2012). Southern margin extended south of the subcrop margin on the basis of the presence of deep wells.
Eastern Silurian-Devonian Aquifers, Northern Midwest Aquifer System	Young, H.L. (1992). Hydrogeology of the Cambrian-Ordovician Aquifer System in the Northern Midwest, United States. U.S. Geological Survey Professional Paper 1405-B, 108 pp. Accessed April 12, 2021 from https://pubs.usgs.gov/pp/1405b/report.pdf	Wilson, J.T. (2012). Water-quality assessment of the Cambrian-Ordovician aquifer system in the northern Midwest, United States. U.S. Geological Survey Scientific Investigations Report 2011–5229, 174 pp. Accessed April 12, 2021 from https://pubs.usgs.gov/sir/2011/5229/pdf/SIR20115229_web.pdf	Kay, R. T., Kraske, K. A. (1996). Ground-Water Levels in Aquifers Used for Residential Supply, Campton Township, Kane County, Illinois. U.S. Geological Survey Water-Resources Investigations Report 96-4009, Accessed April 12, 2021 from https://pubs.usgs.gov/wri/1996/4009/report.pdf	Broader aquifer system title and its location approximated from Fig. 1 of Young (1992). Silurian- and Devonian-aged rock subcrop approximated from Fig. 9 of Wilson (2012). These Silurian-Devonian aquifers subcrop along the western side of Lake Michigan; however, they are underlain by the Cambrian-Ordovician aquifers that subcrop to the west (see Fig. 11 of Young (1992)) and thus this "Silurian-Devonian" area is also included as part of the broader "Cambrian-Ordovician Aquifer System". Further, wells in this area (i.e., the "Silurian-Devonian Area") are known to penetrate the deep "Mount Simon Sandstone" aquifer that is Cambrian in age (for another example, a 711 foot well penetrated the Galena-Platteville Formation in 1860 see page B67 of Young (1992)).
Mississippian-Silurian-Devonian Carbonates, Northern Midwest Aquifer System	Olcott, P.G. (1992). Groundwater Atlas of the United States: Segment 9 Iowa, Michigan, Minnesota, Wisconsin. U.S. Geological Survey Hydrologic Atlas 730-J, 33 pp. Accessed April 12, 2021 from https://pubs.usgs.gov/ha/730j/report.pdf	Young, H.L. (1992). Hydrogeology of the Cambrian-Ordovician Aquifer System in the Northern Midwest, United States. U.S. Geological Survey Professional Paper 1405-B, 108 pp. Accessed April 12, 2021 from https://pubs.usgs.gov/pp/1405b/report.pdf	-	Broader aquifer system title and its location approximated from Fig. 1 of Young (1992). Subarea location approximated from Figs. 10 and 11 of Olcott (1992).
Northeast Missouri Carbonates, Northern Midwest Aquifer System	Miller, J.A. (1995). Ground Water Atlas of the United States: Segment 10, Illinois, Indiana, Kentucky, Ohio, Tennessee. U.S. Geological Survey Hydrologic Atlas 730-K, 30 pp. Accessed April 11, 2021 from https://pubs.usgs.gov/ha/730k/report.pdf	Miller, J.A., Appel, C.L. (1997b). Ground Water Atlas of the United States: Segment 3, Kansas, Missouri, Nebraska. U.S. Geological Survey Hydrologic Atlas 730-D, 26 pp. Accessed April 13, 2021 from	-	Approximated from Fig. 9 of Miller and Appel (1997b) and Fig. 12 of Miller (1995).

Aquifer	Reference 1	Reference 2	Reference 3	Steps taken to delineate
		https://pubs.usgs.gov/ha/730d/report.pdf		
Northern Cambrian-Ordovician Aquifers, Northern Midwest Aquifer System	Young, H.L. (1992). Hydrogeology of the Cambrian-Ordovician Aquifer System in the Northern Midwest, United States. U.S. Geological Survey Professional Paper 1405-B, 108 pp. Accessed April 12, 2021 from https://pubs.usgs.gov/pp/1405b/report.pdf	Wilson, J.T. (2012). Water-quality assessment of the Cambrian-Ordovician aquifer system in the northern Midwest, United States. U.S. Geological Survey Scientific Investigations Report 2011–5229, 174 pp. Accessed April 12, 2021 from https://pubs.usgs.gov/sir/2011/5229/pdf/SIR20115229_web.pdf	-	Broader aquifer system title and its location approximated from Fig. 1 of Young (1992). Subcrop geology approximated from Fig. 9 of Wilson (2012).
Upper Carbonate Aquifer, Northern Midwest Aquifer System	Olcott, P.G. (1992). Groundwater Atlas of the United States: Segment 9 Iowa, Michigan, Minnesota, Wisconsin. U.S. Geological Survey Hydrologic Atlas 730-J, 33 pp. Accessed April 12, 2021 from https://pubs.usgs.gov/ha/730j/report.pdf	Young, H.L. (1992). Hydrogeology of the Cambrian-Ordovician Aquifer System in the Northern Midwest, United States. U.S. Geological Survey Professional Paper 1405-B, 108 pp. Accessed April 12, 2021 from https://pubs.usgs.gov/pp/1405b/report.pdf	-	Broader aquifer system title and its location approximated from Fig. 1 of Young (1992). Upper Carbonate Aquifer location approximated from Fig. 17 of Olcott (1992).
Upper Peninsula Jacobsville Sandstone and Cambrian-Ordovician and Silurian-Devonian Aquifers, Northern Midwest Aquifer System	Olcott, P.G. (1992). Groundwater Atlas of the United States: Segment 9 Iowa, Michigan, Minnesota, Wisconsin. U.S. Geological Survey Hydrologic Atlas 730-J, 33 pp. Accessed April 12, 2021 from https://pubs.usgs.gov/ha/730j/report.pdf	-	-	Approximated from Figs. 77, 97 and 133 by Olcott (1992).
Western Cambrian-Ordovician Aquifers, Northern Midwest Aquifer System	Young, H.L. (1992). Hydrogeology of the Cambrian-Ordovician Aquifer System in the Northern Midwest, United States. U.S. Geological Survey Professional Paper 1405-B, 108 pp. Accessed April 12, 2021 from https://pubs.usgs.gov/pp/1405b/report.pdf	Wilson, J.T. (2012). Water-quality assessment of the Cambrian-Ordovician aquifer system in the northern Midwest, United States. U.S. Geological Survey Scientific Investigations Report 2011–5229, 174 pp. Accessed April 12, 2021 from https://pubs.usgs.gov/sir/2011/5229/pdf/SIR20115229_web.pdf	-	Broader aquifer system title and its location approximated from Fig. 1 of Young (1992). Subcrop geology approximated from Fig. 9 of Wilson (2012). Southern margin extended south of the subcrop margin on the basis of the presence of deep wells.
Osoyoos Aquifer, Okanagan Basin	Rathfelder, K., Gregory, L. (2019). Groundwater quality assessment and proposed objectives for the Osoyoos Aquifer. Water Science Series: WSS2019-06, Province of British Columbia, Victoria, 86 pp. Accessed March 15, 2021 from https://a100.gov.bc.ca/pub/acat/documents/r57603/1_1571784531661_1784376098.pdf	-	-	Approximated from Fig. 1 of Rathfelder and Gregory (2019) and well completion records for Washington and British Columbia

Aquifer	Reference 1	Reference 2	Reference 3	Steps taken to delineate
Central Piedmont Upland, Piedmont Plateau	Miller, J.A. (1990). Ground Water Atlas of the United States: Segment 6, Alabama, Florida, Georgia, South Carolina. U.S. Geological Survey Hydrologic Atlas 730-G, 30 pp. Accessed April 5, 2021 from https://www.nrc.gov/docs/ML1706/ML17060B027.pdf	-	-	Approximated from Fig. 3 of Miller (1990). Northeastern margin of area approximated along the Hardware and James Rivers.
Northcentral Piedmont Upland, Piedmont Plateau	Miller, J.A. (1990). Ground Water Atlas of the United States: Segment 6, Alabama, Florida, Georgia, South Carolina. U.S. Geological Survey Hydrologic Atlas 730-G, 30 pp. Accessed April 5, 2021 from https://www.nrc.gov/docs/ML1706/ML17060B027.pdf	-	-	Approximated from Fig. 3 of Miller (1990). Northeastern margin of area approximated along the Susquehanna River.
Northern Piedmont Upland, Piedmont Plateau	Miller, J.A. (1990). Ground Water Atlas of the United States: Segment 6, Alabama, Florida, Georgia, South Carolina. U.S. Geological Survey Hydrologic Atlas 730-G, 30 pp. Accessed April 5, 2021 from https://www.nrc.gov/docs/ML1706/ML17060B027.pdf	-	-	Approximated from Fig. 3 of Miller (1990).
Southcentral Piedmont Upland, Piedmont Plateau	Miller, J.A. (1990). Ground Water Atlas of the United States: Segment 6, Alabama, Florida, Georgia, South Carolina. U.S. Geological Survey Hydrologic Atlas 730-G, 30 pp. Accessed April 5, 2021 from https://www.nrc.gov/docs/ML1706/ML17060B027.pdf	-	-	Approximated from Fig. 3 of Miller (1990). Northeastern margin of area approximated along the South Yadkin and Pee Dee Rivers.
Southern Piedmont Upland, Piedmont Plateau	Geological Survey of Alabama (2018). Assessment of groundwater resources in Alabama, 2010-16. Geological Survey of Alabama Bulletin 186, 462 pp. Accessed April 8, 2021 from https://www.gsa.state.al.us/img/Groundwater/docs/assessment/00_B186_StatewideAssessment_Print_Document.pdf	Miller, J.A. (1990). Ground Water Atlas of the United States: Segment 6, Alabama, Florida, Georgia, South Carolina. U.S. Geological Survey Hydrologic Atlas 730-G, 30 pp. Accessed April 5, 2021 from https://www.nrc.gov/docs/ML1706/ML17060B027.pdf	-	Approximated from Fig. 4 of Geological Survey of Alabama (2018) and Fig. 3 of Miller (1990). Northeastern margin of area approximated along the Savannah River.
Abbotsford-Sumas Aquifer, Puget Sound Lowland	Scibek, J., Allen, D. M. (2005). Numerical groundwater flow model of the Abbotsford–Sumas aquifer, central Fraser Lowland of BC, Canada, and Washington State, US. Report prepared for Environment Canada, Vancouver, 203 (accessed February 9, 2021 via https://www.sfu.ca/personal/dallen/AB_Modeling_Report_Final.pdf)	Cox, S. E., Kahle, S. C. (1999). Hydrogeology, ground-water quality, and sources of nitrate in lowland glacial aquifers of Whatcom County, Washington, and British Columbia, Canada (Vol. 98, No. 4195). US Department of the Interior, US Geological Survey. https://pubs.er.usgs.gov/publication/wri984195	Vaccaro, J. J., Hansen, A. J., Jones, M. A. (1998). Hydrogeologic framework of the Puget Sound aquifer system, Washington and British Columbia. U.S. Geological Survey Professional Paper 1424-D, 87 pp. Accessed March 11, 2021 from https://pubs.usgs.gov/pp/1424d/report.pdf	Broader Puget Sound Lowland approximated from Vaccaro et al. (1998). Abbotsford-Sumas aquifer approximated from Map 4 of Scibek and Allen (2005) and Plate 2 of Cox and Kahle (1999) (the latter reference led to our extending the southern boundary of the aquifer ~10 km farther south than delineated by Scibek and Allen (2005)
Hood Canal and Admiralty Inlet Sub-basin, Puget Sound Lowland	Vaccaro, J. J., Hansen, A. J., Jones, M. A. (1998). Hydrogeologic framework of the Puget Sound aquifer system, Washington and British Columbia. U.S. Geological Survey Professional Paper 1424-D, 87 pp. Accessed March 11, 2021 from https://pubs.usgs.gov/pp/1424d/report.pdf	Washington Department of Ecology (2010). Puget Sound Groundwater Toxics Loading Analysis: Direct Discharge Pathway. Publication No. 10-03-122, 26 pp. Accessed March 12, 2021 from https://apps.ecology.wa.gov/publications/documents/1003122.pdf	-	Broader Puget Sound Lowland approximated from Vaccaro et al. (1998). Sub-basin outline approximated from Fig. 2 of Washington Department of Ecology (2010).
Lower Fraser Valley, Puget Sound Lowland	Vaccaro, J. J., Hansen, A. J., Jones, M. A. (1998). Hydrogeologic framework of the Puget Sound aquifer system, Washington and British Columbia. U.S. Geological Survey Professional Paper 1424-D, 87 pp. Accessed March 11, 2021 from https://pubs.usgs.gov/pp/1424d/report.pdf	Wilson, J. E., Brown, S., Schreier, H., Scovill, D., Zubel, M. (2008). Arsenic in groundwater wells in Quaternary deposits in the Lower Fraser Valley of British	-	Broader Puget Sound Lowland approximated from Vaccaro et al. (1998). Fraser River Delta outline approximated from Fig. 1 of Bridger and Allen (2006).

Aquifer	Reference 1	Reference 2	Reference 3	Steps taken to delineate
		Columbia. Canadian Water Resources Journal, 33(4), 397-412.		
Main Basin and Sinclair Inlet and Western Hood Canal, Puget Sound Lowland	Vaccaro, J. J., Hansen, A. J., Jones, M. A. (1998). Hydrogeologic framework of the Puget Sound aquifer system, Washington and British Columbia. U.S. Geological Survey Professional Paper 1424-D, 87 pp. Accessed March 11, 2021 from https://pubs.usgs.gov/pp/1424d/report.pdf	Washington Department of Ecology (2010). Puget Sound Groundwater Toxics Loading Analysis: Direct Discharge Pathway. Publication No. 10-03-122, 26 pp. Accessed March 12, 2021 from https://apps.ecology.wa.gov/publications/documents/1003122.pdf	-	Broader Puget Sound Lowland approximated from Vaccaro et al. (1998). Sub-basin outline approximated from Fig. 2 of Washington Department of Ecology (2010).
South Sound and Commencement Bay, Puget Sound Lowland	Vaccaro, J. J., Hansen, A. J., Jones, M. A. (1998). Hydrogeologic framework of the Puget Sound aquifer system, Washington and British Columbia. U.S. Geological Survey Professional Paper 1424-D, 87 pp. Accessed March 11, 2021 from https://pubs.usgs.gov/pp/1424d/report.pdf	Washington Department of Ecology (2010). Puget Sound Groundwater Toxics Loading Analysis: Direct Discharge Pathway. Publication No. 10-03-122, 26 pp. Accessed March 12, 2021 from https://apps.ecology.wa.gov/publications/documents/1003122.pdf	-	Broader Puget Sound Lowland approximated from Vaccaro et al. (1998). Sub-basin outline approximated from Fig. 2 of Washington Department of Ecology (2010).
Strait of Georgia Sub-basin, Puget Sound Lowland	Vaccaro, J. J., Hansen, A. J., Jones, M. A. (1998). Hydrogeologic framework of the Puget Sound aquifer system, Washington and British Columbia. U.S. Geological Survey Professional Paper 1424-D, 87 pp. Accessed March 11, 2021 from https://pubs.usgs.gov/pp/1424d/report.pdf	Washington Department of Ecology (2010). Puget Sound Groundwater Toxics Loading Analysis: Direct Discharge Pathway. Publication No. 10-03-122, 26 pp. Accessed March 12, 2021 from https://apps.ecology.wa.gov/publications/documents/1003122.pdf	-	Broader Puget Sound Lowland approximated from Vaccaro et al. (1998). Sub-basin outline approximated from Fig. 2 of Washington Department of Ecology (2010).
Strait of Juan de Fuca Sub-basin, Puget Sound Lowland	Vaccaro, J. J., Hansen, A. J., Jones, M. A. (1998). Hydrogeologic framework of the Puget Sound aquifer system, Washington and British Columbia. U.S. Geological Survey Professional Paper 1424-D, 87 pp. Accessed March 11, 2021 from https://pubs.usgs.gov/pp/1424d/report.pdf	Washington Department of Ecology (2010). Puget Sound Groundwater Toxics Loading Analysis: Direct Discharge Pathway. Publication No. 10-03-122, 26 pp. Accessed March 12, 2021 from https://apps.ecology.wa.gov/publications/documents/1003122.pdf	-	Broader Puget Sound Lowland approximated from Vaccaro et al. (1998). Sub-basin outline approximated from Fig. 2 of Washington Department of Ecology (2010).
Whidbey and Port Gardner Basins, Puget Sound Lowland	Vaccaro, J. J., Hansen, A. J., Jones, M. A. (1998). Hydrogeologic framework of the Puget Sound aquifer system, Washington and British Columbia. U.S. Geological Survey Professional Paper 1424-D, 87 pp. Accessed March 11, 2021 from https://pubs.usgs.gov/pp/1424d/report.pdf	Washington Department of Ecology (2010). Puget Sound Groundwater Toxics Loading Analysis: Direct Discharge Pathway. Publication No. 10-03-122, 26 pp. Accessed March 12, 2021 from https://apps.ecology.wa.gov/pu	-	Broader Puget Sound Lowland approximated from Vaccaro et al. (1998). Sub-basin outline approximated from Fig. 2 of Washington Department of Ecology (2010).

Aquifer	Reference 1	Reference 2	Reference 3	Steps taken to delineate
		blications/documents/1003122.pdf		
Mesilla Valley, Rincon-Mesilla Valleys	Nickerson, E. L., Myers, R. G. (1993). Geohydrology of the Mesilla ground-water basin, Dona Ana County, New Mexico, and El Paso County, Texas (No. 92-4156). US Geological Survey, Water Resources Division; US Geological Survey Water-Resources Investigations Report 92-4156. Accessed February 17, 2021 from https://pubs.usgs.gov/wri/1992/4156/report.pdf	Hawley, J. W., Lozinsky, R. P. (1992). Hydrogeologic framework of the Mesjlla Basin in New Mexico and western Texas. New Mexico Bureau of Mines and Mineral Resources Open File Report 323, 95 pp. Accessed November 29, 2021 from https://geoinfo.nmt.edu/publications/openfile/downloads/300-399/323/ofr_323.pdfhttps://geoinfo.nmt.edu/publications/openfile/downloads/300-399/323/ofr_323.pdf	-	Approximated from map on page 10 of Nickerson and Myers (1993) and Fig. 1 by Hawley and Lozinsky (1993)
Rincon Valley, Rincon-Mesilla Valleys	Fuchs, E. H., King, J. P., Carroll, K. C. (2019). Quantifying disconnection of groundwater from managed-ephemeral surface water during drought and conjunctive agricultural use. Water Resources Research, 55(7), 5871-5890.	-	-	Approximated from Fig. 2 of Fuchs et al. (2019)
East Salt River Basin, Salt Basin	Flora, S., Davis, T. (2009). Hydrologic Map Series (HMS), Water Level Change Map Series (WLCMS), and Basin Sweep Assessment Report ADWR Basins and Sub-Basins. Arizona Department of Water Resources Hydrology Division Field Services Section https://new.azwater.gov/sites/default/files/HMSWLCMSBasinSweepAssessmentReport2009.pdf	Laney, R. L., Hahn, M.E. (1986). Hydrogeology of the eastern part of the Salt River Valley area, Maricopa and Pinal Counties, Arizona. U.S. Geological Survey Water-Resources Investigations Report Water-Resources Investigations Report, 8 maps on 4 sheets. Accessed March 31, 2021 from https://pubs.er.usgs.gov/publication/wri864147	-	Approximated from Fig. 1 of Flora and Davis (2009). Hydrologic Map Series (HMS), Water Level Change Map Series (WLCMS), and Basin Sweep Assessment Report ADWR Basins and Sub-Basins. Arizona Department of Water Resources Hydrology Division Field Services Section https://new.azwater.gov/sites/default/files/HMSWLCMSBasinSweepAssessmentReport2009.pdf. See also: Laney and Hahn (1986).
West Salt River Basin, Salt Basin	Flora, S., Davis, T. (2009). Hydrologic Map Series (HMS), Water Level Change Map Series (WLCMS), and Basin Sweep Assessment Report ADWR Basins and Sub-Basins. Arizona Department of Water Resources Hydrology Division Field Services Section https://new.azwater.gov/sites/default/files/HMSWLCMSBasinSweepAssessmentReport2009.pdf	Anning, D. (2014). Conceptual Understanding and Groundwater Quality of Selected Basin-Fill Aquifers in the Southwestern United States: Section 7, Conceptual Understanding and Groundwater Quality of the Basin-Fill Aquifer in the West Salt River Valley, Arizona. U.S. Geological Survey Professional Paper 1781, 24 pp. Accessed March 31, 2021 from https://pubs.usgs.gov/pp/1781/pdf/pp1781_section7.pdf	-	Approximated from Fig. 1 of Flora and Davis (2009). Hydrologic Map Series (HMS), Water Level Change Map Series (WLCMS), and Basin Sweep Assessment Report ADWR Basins and Sub-Basins. Arizona Department of Water Resources Hydrology Division Field Services Section https://new.azwater.gov/sites/default/files/HMSWLCMSBasinSweepAssessmentReport2009.pdf. See also: Anning (2014).
Lower Santa Ynez Valley, Santa Ynez Valley	Upson, J. E., Thomasson, H. G. (1951). Geology and water resources of the Santa Ynez river basin, Santa Barbara County, California (Vol. 2). US Geological Survey Water Supply Report 1102, Accessed March 7, 2021 from https://pubs.usgs.gov/wsp/1107/report.pdf	Hamlin, S. N. (1985). Ground-water quality in the Santa Rita, Buellton, and Los Olivos hydrologic subareas of the	Wilson, H. D. (1959). Ground-water appraisal of Santa Ynez River basin, Santa Barbara County, California,	Approximated from Fig. 1 by Upson and Thomasson (1951), pp. 22 map in Hamlin (1985), and Fig. 2 of Wilson (1959). Lower portion of Valley based on the delineated

Aquifer	Reference 1	Reference 2	Reference 3	Steps taken to delineate
		Santa Ynez River basin, Santa Barbara County, California. Water Resources Investigations Report 84-4131, 84 pp. Accessed March 7, 2021 from https://pubs.usgs.gov/wri/1984/4131/report.pdf	1945-52. Water Supply Paper 1467. 128 pp. Accessed March 7, 2021 from https://pubs.usgs.gov/wsp/1467/report.pdf	subareas: "Lompoc" and "Santa Rita" and "Buellton" subarea
Upper Santa Ynez Valley, Santa Ynez Valley	Upson, J. E., Thomasson, H. G. (1951). Geology and water resources of the Santa Ynez river basin, Santa Barbara County, California (Vol. 2). US Geological Survey Water Supply Report 1102, Accessed March 7, 2021 from https://pubs.usgs.gov/wsp/1107/report.pdf	Hamlin, S. N. (1985). Ground-water quality in the Santa Rita, Buellton, and Los Olivos hydrologic subareas of the Santa Ynez River basin, Santa Barbara County, California. Water Resources Investigations Report 84-4131, 84 pp. Accessed March 7, 2021 from https://pubs.usgs.gov/wri/1984/4131/report.pdf	Wilson, H. D. (1959). Ground-water appraisal of Santa Ynez River basin, Santa Barbara County, California, 1945-52. Water Supply Paper 1467. 128 pp. Accessed March 7, 2021 from https://pubs.usgs.gov/wsp/1467/report.pdf	Approximated from Fig. 1 by Upson and Thomasson (1951), pp. 22 map in Hamlin (1985), and Fig. 2 of Wilson (1959). Upper portion of Valley based on the delineated subareas: "Santa Ynez" and "Headwater Subarea"
American and Auburn Basins, Sierra Nevada Foothills	Shelton, J.L., Fram, M.S., Munday, C.M., Belitz, K. (2010). Groundwater-quality data for the Sierra Nevada study unit, 2008. Results from the California GAMA program. U.S. Geological Survey Data Series 534, 106 pp. Accessed April 15, 2021 from https://pubs.usgs.gov/ds/534/ds_534.pdf	-	-	Approximated on the basis of the density of completed wells (from the California Department of Water Resources); general hydrogeology of region summarized by Shelton et al. (2010).
Butte Creek Basin, Sierra Nevada Foothills	Shelton, J.L., Fram, M.S., Munday, C.M., Belitz, K. (2010). Groundwater-quality data for the Sierra Nevada study unit, 2008. Results from the California GAMA program. U.S. Geological Survey Data Series 534, 106 pp. Accessed April 15, 2021 from https://pubs.usgs.gov/ds/534/ds_534.pdf	-	-	Approximated on the basis of the density of completed wells (from the California Department of Water Resources); general hydrogeology of region summarized by Shelton et al. (2010).
Calaveras Basin, Sierra Nevada Foothills	Shelton, J.L., Fram, M.S., Munday, C.M., Belitz, K. (2010). Groundwater-quality data for the Sierra Nevada study unit, 2008. Results from the California GAMA program. U.S. Geological Survey Data Series 534, 106 pp. Accessed April 15, 2021 from https://pubs.usgs.gov/ds/534/ds_534.pdf	-	-	Approximated on the basis of the density of completed wells (from the California Department of Water Resources); general hydrogeology of region summarized by Shelton et al. (2010).
Chowchilla Basin, Sierra Nevada Foothills	Shelton, J.L., Fram, M.S., Munday, C.M., Belitz, K. (2010). Groundwater-quality data for the Sierra Nevada study unit, 2008. Results from the California GAMA program. U.S. Geological Survey Data Series 534, 106 pp. Accessed April 15, 2021 from https://pubs.usgs.gov/ds/534/ds_534.pdf	-	-	Approximated on the basis of the density of completed wells (from the California Department of Water Resources); general hydrogeology of region summarized by Shelton et al. (2010).
Cosumnes Basin, Sierra Nevada Foothills	Shelton, J.L., Fram, M.S., Munday, C.M., Belitz, K. (2010). Groundwater-quality data for the Sierra Nevada study unit, 2008. Results from the California GAMA program. U.S. Geological Survey Data Series 534, 106 pp. Accessed April 15, 2021 from https://pubs.usgs.gov/ds/534/ds_534.pdf	-	-	Approximated on the basis of the density of completed wells (from the California Department of Water Resources); general hydrogeology of region summarized by Shelton et al. (2010).
Deer and White Basins, Sierra Nevada Foothills	Shelton, J.L., Fram, M.S., Munday, C.M., Belitz, K. (2010). Groundwater-quality data for the Sierra Nevada study unit, 2008. Results from the California GAMA program. U.S. Geological Survey Data Series 534, 106 pp. Accessed April 15, 2021 from https://pubs.usgs.gov/ds/534/ds_534.pdf	-	-	Approximated on the basis of the density of completed wells (from the California Department of Water Resources); general hydrogeology of region summarized by Shelton et al. (2010).
Dry and King Basins, Sierra Nevada Foothills	Shelton, J.L., Fram, M.S., Munday, C.M., Belitz, K. (2010). Groundwater-quality data for the Sierra Nevada study unit, 2008. Results from the California GAMA program. U.S. Geological Survey Data Series 534, 106 pp. Accessed April 15, 2021 from https://pubs.usgs.gov/ds/534/ds_534.pdf	-	-	Approximated on the basis of the density of completed wells (from the California Department of Water Resources); general

Aquifer	Reference 1	Reference 2	Reference 3	Steps taken to delineate
				hydrogeology of region summarized by Shelton et al. (2010).
Feather River Basin, Sierra Nevada Foothills	Shelton, J.L., Fram, M.S., Munday, C.M., Belitz, K. (2010). Groundwater-quality data for the Sierra Nevada study unit, 2008. Results from the California GAMA program. U.S. Geological Survey Data Series 534, 106 pp. Accessed April 15, 2021 from https://pubs.usgs.gov/ds/534/ds_534.pdf	-	-	Approximated on the basis of the density of completed wells (from the California Department of Water Resources); general hydrogeology of region summarized by Shelton et al. (2010).
Fresno Basin, Sierra Nevada Foothills	Shelton, J.L., Fram, M.S., Munday, C.M., Belitz, K. (2010). Groundwater-quality data for the Sierra Nevada study unit, 2008. Results from the California GAMA program. U.S. Geological Survey Data Series 534, 106 pp. Accessed April 15, 2021 from https://pubs.usgs.gov/ds/534/ds_534.pdf	-	-	Approximated on the basis of the density of completed wells (from the California Department of Water Resources); general hydrogeology of region summarized by Shelton et al. (2010).
Kaweah Basin, Sierra Nevada Foothills	Shelton, J.L., Fram, M.S., Munday, C.M., Belitz, K. (2010). Groundwater-quality data for the Sierra Nevada study unit, 2008. Results from the California GAMA program. U.S. Geological Survey Data Series 534, 106 pp. Accessed April 15, 2021 from https://pubs.usgs.gov/ds/534/ds_534.pdf	-	-	Approximated on the basis of the density of completed wells (from the California Department of Water Resources); general hydrogeology of region summarized by Shelton et al. (2010).
Merced Basin, Sierra Nevada Foothills	Shelton, J.L., Fram, M.S., Munday, C.M., Belitz, K. (2010). Groundwater-quality data for the Sierra Nevada study unit, 2008. Results from the California GAMA program. U.S. Geological Survey Data Series 534, 106 pp. Accessed April 15, 2021 from https://pubs.usgs.gov/ds/534/ds_534.pdf	-	-	Approximated on the basis of the density of completed wells (from the California Department of Water Resources); general hydrogeology of region summarized by Shelton et al. (2010).
Mokelumne Basin, Sierra Nevada Foothills	Shelton, J.L., Fram, M.S., Munday, C.M., Belitz, K. (2010). Groundwater-quality data for the Sierra Nevada study unit, 2008. Results from the California GAMA program. U.S. Geological Survey Data Series 534, 106 pp. Accessed April 15, 2021 from https://pubs.usgs.gov/ds/534/ds_534.pdf	-	-	Approximated on the basis of the density of completed wells (from the California Department of Water Resources); general hydrogeology of region summarized by Shelton et al. (2010).
Poso Basin, Sierra Nevada Foothills	Shelton, J.L., Fram, M.S., Munday, C.M., Belitz, K. (2010). Groundwater-quality data for the Sierra Nevada study unit, 2008. Results from the California GAMA program. U.S. Geological Survey Data Series 534, 106 pp. Accessed April 15, 2021 from https://pubs.usgs.gov/ds/534/ds_534.pdf	-	-	Approximated on the basis of the density of completed wells (from the California Department of Water Resources); general hydrogeology of region summarized by Shelton et al. (2010).
Stanislaus and Rock Creek Basins, Sierra Nevada Foothills	Shelton, J.L., Fram, M.S., Munday, C.M., Belitz, K. (2010). Groundwater-quality data for the Sierra Nevada study unit, 2008. Results from the California GAMA program. U.S. Geological Survey Data Series 534, 106 pp. Accessed April 15, 2021 from https://pubs.usgs.gov/ds/534/ds_534.pdf	-	-	Approximated on the basis of the density of completed wells (from the California Department of Water Resources); general hydrogeology of region summarized by Shelton et al. (2010).
Tule Basin, Sierra Nevada Foothills	Shelton, J.L., Fram, M.S., Munday, C.M., Belitz, K. (2010). Groundwater-quality data for the Sierra Nevada study unit, 2008. Results from the California GAMA program. U.S. Geological Survey Data Series 534, 106 pp. Accessed April 15, 2021 from https://pubs.usgs.gov/ds/534/ds_534.pdf	-	-	Approximated on the basis of the density of completed wells (from the California Department of Water Resources); general hydrogeology of region summarized by Shelton et al. (2010).
Tuolumne Basin, Sierra Nevada Foothills	Shelton, J.L., Fram, M.S., Munday, C.M., Belitz, K. (2010). Groundwater-quality data for the Sierra Nevada study unit, 2008. Results from the California GAMA program. U.S. Geological Survey Data Series 534, 106 pp. Accessed April 15, 2021 from https://pubs.usgs.gov/ds/534/ds_534.pdf	-	-	Approximated on the basis of the density of completed wells (from the California Department of Water Resources); general hydrogeology of region summarized by Shelton et al. (2010).
Upper Bear Basin, Sierra Nevada Foothills	Shelton, J.L., Fram, M.S., Munday, C.M., Belitz, K. (2010). Groundwater-quality data for the Sierra Nevada study unit, 2008. Results from the California GAMA program. U.S. Geological Survey Data Series 534, 106 pp. Accessed April 15, 2021 from https://pubs.usgs.gov/ds/534/ds_534.pdf	-	-	Approximated on the basis of the density of completed wells (from the California Department of Water Resources); general hydrogeology of region summarized by Shelton et al. (2010).

Aquifer	Reference 1	Reference 2	Reference 3	Steps taken to delineate
Upper San Joaquin, Sierra Nevada Foothills	Shelton, J.L., Fram, M.S., Munday, C.M., Belitz, K. (2010). Groundwater-quality data for the Sierra Nevada study unit, 2008. Results from the California GAMA program. U.S. Geological Survey Data Series 534, 106 pp. Accessed April 15, 2021 from https://pubs.usgs.gov/ds/534/ds_534.pdf	-	-	Approximated on the basis of the density of completed wells (from the California Department of Water Resources); general hydrogeology of region summarized by Shelton et al. (2010).
Yuba Basin, Sierra Nevada Foothills	Shelton, J.L., Fram, M.S., Munday, C.M., Belitz, K. (2010). Groundwater-quality data for the Sierra Nevada study unit, 2008. Results from the California GAMA program. U.S. Geological Survey Data Series 534, 106 pp. Accessed April 15, 2021 from https://pubs.usgs.gov/ds/534/ds_534.pdf	-	-	Approximated on the basis of the density of completed wells (from the California Department of Water Resources); general hydrogeology of region summarized by Shelton et al. (2010).
Southern St. Lawrence Lowlands, St. Lawrence Lowlands	Parent, M., Rivard, C., Lefebvre, R., Carrier, M.-A., Séjourné, S. (2014). Hydrogeological systems of the Montérégie Est region, southern Québec: Fieldtrip Guidebook, GeoMontreal 2013 Conference, Geological Survey of Canada Open File 7605, 43 pp.	Olcott, P.G. (1995). Ground Water Atlas of the United States: Segment 12, Connecticut, Maine, Massachusetts, New Hampshire, New York, Rhode Island, Vermont. U.S. Geological Survey Hydrologic Atlas 730-M, 30 pp. Accessed April 14, 2021 from https://pubs.usgs.gov/ha/730m/report.pdf	Patenaude, M., Baudron, P., Labelle, L., Masse-Dufresne, J. (2020). Evaluating bank-filtration occurrence in the Province of Quebec (Canada) with a GIS approach. Water 12, 662.	St. Lawrence Lowlands hydrogeological context approximated from Fig. 2 of Parent et al. (2014; including the divide between northern and southern portions of the broader aquifer system) and Fig. 4 by Patenaude et al. (2020); see also Fig. 102 by Olcutt (1995).
Eastern C Aquifer, The C Aquifer	Brown, C.R., Macy, J.P. (2012). Groundwater, Surface-Water, and Water-Chemistry Data from the C-Aquifer Monitoring Program, Northeastern Arizona, 2005–2011. U.S. Geological Survey Open-File Report 2012–1196, 46 pp. Accessed April 15, 2021 from https://pubs.usgs.gov/of/2012/1196/of2012-1196.pdf	-	-	Approximated from Fig. 1 of Brown and Macy (2012). East and west portions divided near Joseph City.
Western C Aquifer, The C Aquifer	Brown, C.R., Macy, J.P. (2012). Groundwater, Surface-Water, and Water-Chemistry Data from the C-Aquifer Monitoring Program, Northeastern Arizona, 2005–2011. U.S. Geological Survey Open-File Report 2012–1196, 46 pp. Accessed April 15, 2021 from https://pubs.usgs.gov/of/2012/1196/of2012-1196.pdf	-	-	Approximated from Fig. 1 of Brown and Macy (2012). East and west portions divided near Joseph City.
Avra Valley, Tucson Basin and Avra Valley	Flora, S., Davis, T. (2009). Hydrologic Map Series (HMS), Water Level Change Map Series (WLCMS), and Basin Sweep Assessment Report ADWR Basins and Sub-Basins. Arizona Department of Water Resources Hydrology Division Field Services Section https://new.azwater.gov/sites/default/files/HMSWLCMSBasinSweepAssessmentReport2009.pdf	Carruth, R.L., Kahler, L.M., Conway, B.D. (2018). Groundwater-storage change and land-surface elevation change in Tucson Basin and Avra Valley, south-central Arizona—2003–2016: U.S. Geological Survey Scientific Investigations Report 2018–5154, 34 pp. Accessed April 1, 2021 from https://pubs.usgs.gov/sir/2018/5154/sir20185154.pdf	Hanson, R.T. (1989). Aquifer-system compaction, Tucson Basin and Avra Valley, Arizona. Water-Resources Investigations Report 88-4172, 75 pp. Accessed April 15, 2021 from https://pubs.usgs.gov/wri/1988/4172/report.pdf	Approximated from Fig. 1 of Flora and Davis (2009). See also Fig. 1 of Carruth et al. (2018). Broader Tucson Basin and Avra Valley outline from Fig. 1 of Hanson (1989).
Upper Santa Cruz Basin, Tucson Basin and Avra Valley	Flora, S., Davis, T. (2009). Hydrologic Map Series (HMS), Water Level Change Map Series (WLCMS), and Basin Sweep Assessment Report ADWR Basins and Sub-Basins. Arizona Department of Water Resources Hydrology Division Field Services Section https://new.azwater.gov/sites/default/files/HMSWLCMSBasinSweepAssessmentReport2009.pdf	Coes, A., Gellenbeck, D.J., Towne, D.C., Freark, M.C. (2002). Ground water quality in the Upper Santa Cruz Basin. U.S. Geological Survey Water-Resources Investigations Report 00-4117, 66 pp. Accessed March 31, 2021 from	Hanson, R.T. (1989). Aquifer-system compaction, Tucson Basin and Avra Valley, Arizona. Water-Resources Investigations Report 88-4172, 75 pp. Accessed April 15, 2021 from	Approximated from Fig. 1 of Flora and Davis (2009). See also Coes et al. (2002). Broader Tucson Basin and Avra Valley outline from Fig. 1 of Hanson (1989).

Aquifer	Reference 1	Reference 2	Reference 3	Steps taken to delineate
		https://pubs.usgs.gov/wri/2000/4117/report.pdf	https://pubs.usgs.gov/wri/1988/4172/report.pdf	
Tularosa Basin, Tularosa-Huelco	McLean, J. S. (1970). Saline ground-water resources of the Tularosa basin, New Mexico (No. 561). US Geological Survey. Accessed February 17, 2021 from https://pubs.usgs.gov/unnumbered/70139928/report.pdf	Huff, G. F. (2005). Simulation of ground-water flow in the basin-fill aquifer of the Tularosa Basin, south-central New Mexico, predevelopment through 2040 (Vol. 4, No. 4). US Geological Survey Scientific Investigations Report 2004-5197, 108 pp. Accessed February 17, 2021 from https://pubs.usgs.gov/sir/2004/5197/pdf/sir20045197.pdf	-	Approximated from Fig. 1 of McLean (1970) and Fig. 1 by Huff (2005)
Valle de Juarez and Hueco Bolson, Tularosa-Huelco	Sanchez, R., Eckstein, G. (2020). Groundwater management in the borderlands of Mexico and Texas: The beauty of the unknown, the negligence of the present, and the way forward. Water Resources Research, 56(3), e2019WR026068.	Day, J. C. (1978). International Aquifer Management: The Hueco Bolson on the Rio Grande River. Natural Resources Journal, 163-180.	-	Approximated from Fig. 1 of Day (1978) and Fig. 2 of Sanchez and Eckstein (2020).
Ashley Valley, Uinta Basin	Hood, J. W. (1976). Characteristics of aquifers in the northern Uinta Basin area, Utah and Colorado. U.S. Geological Survey and Utah Department of Natural Resources, Division of Water Rights Technical Publication 53, 71 pp. Accessed March 7, 2021 from https://waterrights.utah.gov/docSys/v920/w920/w920009f.pdf	Zhang, Y., Gable, C. W., Zyvoloski, G. A., Walter, L. M. (2009). Hydrogeochemistry and gas compositions of the Uinta Basin: A regional-scale overview. AAPG Bulletin, 93(8), 1087-1118.	Hood, J. W. (1977). Hydrologic evaluation of Ashley Valley, northern Uinta Basin area, Utah. State of Utah Department of Natural Resources, Division of Water Rights Technical Publication 54, 71 pp. Accessed March 7, 2021 from https://pubs.usgs.gov/unnumbered/70043723/report.pdf	Northern Uinta Basin outline from Plates 1-3 of Hood (1976) and Fig. 1 by Zhang et al. (2009). Ashley Valley outline approximated from Fig. 1 of Hood (1977) and extended to northern margin of Uinta Basin
Birds Nest Aquifer, Uinta Basin	Vanden Berg, M. D., Lehle, D. R., Carney, S. M., Morgan, C. D. (2013). Geological characterization of the Bird's Nest Aquifer, Uinta Basin, Utah: assessment of the aquifer's potential as a saline water disposal unit. Utah Geological Survey Special Study 147, 57 pp. Accessed March 9, 2021 from https://ugspub.nr.utah.gov/publications/special_studies/ss-147/ss-147.pdf	-	-	Birds Nest Aquifer (lower) outline approximated from Fig. 19 of Vanden Berg et al. (2013).
Eastern Uinta Basin and Diamond Plateau, Uinta Basin	Hood, J. W. (1976). Characteristics of aquifers in the northern Uinta Basin area, Utah and Colorado. U.S. Geological Survey and Utah Department of Natural Resources, Division of Water Rights Technical Publication 53, 71 pp. Accessed March 7, 2021 from https://waterrights.utah.gov/docSys/v920/w920/w920009f.pdf	-	-	Northern Uinta Basin and Diamond Plateau region approximated from Plates 1-3 of Hood (1976).
Roosevelt Valley and Uinta River Basin and Pelican Lake region, Uinta Basin	Hood, J. W. (1976). Characteristics of aquifers in the northern Uinta Basin area, Utah and Colorado. U.S. Geological Survey and Utah Department of Natural Resources, Division of Water Rights Technical Publication 53, 71 pp. Accessed March 7, 2021 from https://waterrights.utah.gov/docSys/v920/w920/w920009f.pdf	Lambert, P.M., Marston, T., Kimball, B.A., Stolp, B.J. (2011). Assessment of groundwater/surface-water interaction and simulation of potential streamflow depletion induced by groundwater withdrawal, Uinta River near Roosevelt, Utah: U.S. Geological Survey Scientific Investigations Report 2011–5044, 47 pp.	-	Northern Uinta Basin and Roosevelt Valley, Uinta River Basin and Pelican Lake region outlines approximated from Plates 1-3 of Hood (1976). Further information on Roosevelt Valley available via Lambert et al. (2011)

Aquifer	Reference 1	Reference 2	Reference 3	Steps taken to delineate
		Accessed March 9, 2021 from https://pubs.usgs.gov/sir/2011/5044/pdf/sir20115044.pdf		
Western Uinta Basin, Uinta Basin	Hood, J. W. (1976). Characteristics of aquifers in the northern Uinta Basin area, Utah and Colorado. U.S. Geological Survey and Utah Department of Natural Resources, Division of Water Rights Technical Publication 53, 71 pp. Accessed March 7, 2021 from https://waterrights.utah.gov/docSys/v920/w920/w920009f.pdf	-	-	Northern Uinta Basin approximated from Plates 1-3 of Hood (1976).
Butte Valley, Upper Klamath Basin	Wood, P. R. (1960). Geology and ground-water features of the Butte Valley region, Siskiyou County, California. Geological Survey Water-Supply Paper 1491, 155 pp. Accessed March 20, 2021 from https://pdfs.semanticscholar.org/a448/a58a3c1ac120d400d26f75b74512f0a868a4.pdf	-	-	Approximated from Fig. 1 of Wood (1960)
Klamath River, Upper Klamath Basin	Gannett, M.W., Breen, K.H. (2015). Groundwater levels, trends, and relations to pumping in the Bureau of Reclamation Klamath Project, Oregon and California: U.S. Geological Survey Open-File Report 2015-1145, 19 pp. Accessed March 20, 2021 from https://pubs.usgs.gov/of/2015/1145/ofr20151145.pdf	-	-	Approximated from Fig. 1 of Gannett and Breen (2015).
Klamath Valley, Upper Klamath Basin	Gannett, M.W., Breen, K.H. (2015). Groundwater levels, trends, and relations to pumping in the Bureau of Reclamation Klamath Project, Oregon and California: U.S. Geological Survey Open-File Report 2015-1145, 19 pp. Accessed March 20, 2021 from https://pubs.usgs.gov/of/2015/1145/ofr20151145.pdf	-	-	Approximated from Fig. 1 of Gannett and Breen (2015).
Lower Klamath Lake, Upper Klamath Basin	Gannett, M.W., Breen, K.H. (2015). Groundwater levels, trends, and relations to pumping in the Bureau of Reclamation Klamath Project, Oregon and California: U.S. Geological Survey Open-File Report 2015-1145, 19 pp. Accessed March 20, 2021 from https://pubs.usgs.gov/of/2015/1145/ofr20151145.pdf	-	-	Approximated from Fig. 1 of Gannett and Breen (2015).
Northern Tule Lake, Upper Klamath Basin	Gannett, M.W., Breen, K.H. (2015). Groundwater levels, trends, and relations to pumping in the Bureau of Reclamation Klamath Project, Oregon and California: U.S. Geological Survey Open-File Report 2015-1145, 19 pp. Accessed March 20, 2021 from https://pubs.usgs.gov/of/2015/1145/ofr20151145.pdf	-	-	Approximated from Fig. 1 of Gannett and Breen (2015).
Southern Tule Lake, Upper Klamath Basin	Gannett, M.W., Breen, K.H. (2015). Groundwater levels, trends, and relations to pumping in the Bureau of Reclamation Klamath Project, Oregon and California: U.S. Geological Survey Open-File Report 2015-1145, 19 pp. Accessed March 20, 2021 from https://pubs.usgs.gov/of/2015/1145/ofr20151145.pdf	-	-	Approximated from Fig. 1 of Gannett and Breen (2015).
Sprague Basin, Upper Klamath Basin	Gannett, M. W., Lite, K. E., La Marche, J. L., Fisher, B. J., Polette, D. J. (2007). Ground-water hydrology of the upper Klamath Basin, Oregon and California. U. S. Geological Survey Scientific Investigations Report 2007–5050. 98 pp. Accessed March 20, 2021 from https://pubs.usgs.gov/sir/2007/5050/pdf/sir20075050.pdf	-	-	Approximated from Fig. 18 of
Swan Lake and Upper Klamath Lake Perimeter, Upper Klamath Basin	Gannett, M. W., Lite, K. E., La Marche, J. L., Fisher, B. J., Polette, D. J. (2007). Ground-water hydrology of the upper Klamath Basin, Oregon and California. U. S. Geological Survey Scientific Investigations Report 2007–5050. 98 pp. Accessed March 20, 2021 from https://pubs.usgs.gov/sir/2007/5050/pdf/sir20075050.pdf	-	-	Approximated from Fig. 18 of
Upper Lost River, Upper Klamath Basin	Gannett, M.W., Breen, K.H. (2015). Groundwater levels, trends, and relations to pumping in the Bureau of Reclamation Klamath Project, Oregon and California: U.S. Geological Survey Open-File Report 2015-1145, 19 pp.	-	-	Approximated from Fig. 1 of Gannett and Breen (2015).

Aquifer	Reference 1	Reference 2	Reference 3	Steps taken to delineate
	Accessed March 20, 2021 from https://pubs.usgs.gov/of/2015/1145/ofr20151145.pdf			
Northcentral Valley and Ridge, Valley and Ridge Aquifer System	Johnson, G.C., Zimmerman, T.M., Lindsey, B.D., Gross, E.L. (2011). Factors affecting groundwater quality in the Valley and Ridge aquifers, eastern United States, 1993–2002: U.S. Geological Survey Scientific Investigations Report 2011–5115, 84 pp. Accessed April 13, 2021 from https://pubs.usgs.gov/sir/2011/5115/support/sir2011-5115.pdf	-	-	Broader Valley and Ridge Aquifer System outline approximated from Fig. 1 of Johnson et al. (2011). Sorthern margin approximated at Deep Creek Lake and North Fork Shenandoah River.
Northern Valley and Ridge, Valley and Ridge Aquifer System	Johnson, G.C., Zimmerman, T.M., Lindsey, B.D., Gross, E.L. (2011). Factors affecting groundwater quality in the Valley and Ridge aquifers, eastern United States, 1993–2002: U.S. Geological Survey Scientific Investigations Report 2011–5115, 84 pp. Accessed April 13, 2021 from https://pubs.usgs.gov/sir/2011/5115/support/sir2011-5115.pdf	-	-	Southern boundary approximated at Deep Creek Lake and North Fork Shenandoah River. Broader Valley and Ridge Aquifer System outline approximated from Fig. 1 of Johnson et al. (2011).
Southcentral Valley and Ridge, Valley and Ridge Aquifer System	Johnson, G.C., Zimmerman, T.M., Lindsey, B.D., Gross, E.L. (2011). Factors affecting groundwater quality in the Valley and Ridge aquifers, eastern United States, 1993–2002: U.S. Geological Survey Scientific Investigations Report 2011–5115, 84 pp. Accessed April 13, 2021 from https://pubs.usgs.gov/sir/2011/5115/support/sir2011-5115.pdf	Brahana, J.V., Macy, J.A., Mulderink, D., Zemo, D. (1986). Preliminary delineation and description of the regional aquifers of Tennessee-Cumberland plateau aquifer system. U.S. Geological Survey, Water-Resources Investigations Report 82-338, 29 pp. Accessed April 13, 2021 from https://pubs.usgs.gov/wri/wrir82-338/pdf/wrir_82-338_a.pdf	-	Northern margin approximated along the Hiwassee River. Broader Valley and Ridge Aquifer System outline approximated from Fig. 1 of Johnson et al. (2011) and the Tennessee portion approximated from Fig. 1 of Brahana et al. (1986)
Southern Valley and Ridge, Valley and Ridge Aquifer System	Johnson, G.C., Zimmerman, T.M., Lindsey, B.D., Gross, E.L. (2011). Factors affecting groundwater quality in the Valley and Ridge aquifers, eastern United States, 1993–2002: U.S. Geological Survey Scientific Investigations Report 2011–5115, 84 pp. Accessed April 13, 2021 from https://pubs.usgs.gov/sir/2011/5115/support/sir2011-5115.pdf	Geological Survey of Alabama (2018). Assessment of groundwater resources in Alabama, 2010-16. Geological Survey of Alabama Bulletin 186, 462 pp. Accessed April 8, 2021 from https://www.gsa.state.al.us/img/Groundwater/docs/assessment/00_B186_StatewideAssessment_Print_Document.pdf	-	Northern margin approximated along the Watauga River. Broader Valley and Ridge Aquifer System outline approximated from Fig. 1 of Johnson et al. (2011), with southernmost extent approximated from Fig. 4 of Geological Survey of Alabama (2018)
Boise Valley and Homedale Murphy Area, Western Snake River Plain	Graham, W. G., Campbell, L. J. (1981). Groundwater resources of Idaho. Idaho Department of Water Resources Report, 61 pp. Accessed March 23, 2021 from https://idwr.idaho.gov/files/publications/198108-MISC-GW-Resources-ID.pdf	Lindholm, G. F. (1996). Summary of the Snake River Plain regional aquifer-system analysis in Idaho and eastern Oregon. U.S Geological Survey Professional Paper 1408-A. 59 pp. Accessed March 1, 2021 via https://pubs.usgs.gov/pp/1408a/report.pdf	-	Approximated from Plate 1 of Graham and Campbell (1981). Zones "30" and "32" merged into one; locations of completed wells were used to guide the western boundary of the area. Broader portions of the Snake River Plain (i.e., Central, Western and Eastern) were approximated from Fig. 1 of Lindholm (1996).
Bruneau-Grandview Area, Western Snake River Plain	Graham, W. G., Campbell, L. J. (1981). Groundwater resources of Idaho. Idaho Department of Water Resources Report, 61 pp. Accessed March 23, 2021 from https://idwr.idaho.gov/files/publications/198108-MISC-GW-Resources-ID.pdf	Lindholm, G. F. (1996). Summary of the Snake River Plain regional aquifer-system analysis in Idaho and eastern Oregon. U.S Geological Survey Professional Paper 1408-A. 59 pp. Accessed March 1, 2021 via	-	Approximated from Plate 1 of Graham and Campbell (1981). Broader portions of the Snake River Plain (i.e., Central, Western and Eastern) were approximated from Fig. 1 of Lindholm (1996).

Aquifer	Reference 1	Reference 2	Reference 3	Steps taken to delineate
		https://pubs.usgs.gov/pp/1408a/report.pdf		
Mountain Home Plateau, Western Snake River Plain	Graham, W. G., Campbell, L. J. (1981). Groundwater resources of Idaho. Idaho Department of Water Resources Report, 61 pp. Accessed March 23, 2021 from https://idwr.idaho.gov/files/publications/198108-MISC-GW-Resources-ID.pdf	Young, H.W. (1978). Reconnaissance of ground-water resources in the Mountain Home plateau area, southwest Idaho. U.S. Geological Survey Water--Resources Investigations Report 77-108, 48 pp. Accessed November 29, 2021 from https://pubs.usgs.gov/wri/1977/0108/report.pdf	Lindholm, G. F. (1996). Summary of the Snake River Plain regional aquifer-system analysis in Idaho and eastern Oregon. U.S Geological Survey Professional Paper 1408-A. 59 pp. Accessed March 1, 2021 via https://pubs.usgs.gov/pp/1408a/report.pdf	Approximated from Plate 1 of Graham and Campbell (1981) and Fig. 1 by Young (1981). Broader portions of the Snake River Plain (i.e., Central, Western and Eastern) were approximated from Fig. 1 of Lindholm (1996).
Central Willamette Valley, Willamette Valley	Woodward, D. G., Gannett, M. W., Vaccaro, J. J. (1998). Hydrogeologic framework of the Willamette Lowland aquifer system, Oregon and Washington. US Geological Survey Professional Paper 1424-B, 92 pp. Accessed February 16, 2021 from https://pubs.usgs.gov/pp/1424b/report.pdf	Herrera, N. B., Burns, E. R., Conlon, T. D. (2014). Simulation of groundwater flow and the interaction of groundwater and surface water in the Willamette Basin and Central Willamette Subbasin, Oregon. US Geological Survey Scientific Investigations Report 2014–5136, 174 pp. Accessed February 16, 2021 from https://pubs.usgs.gov/sir/2014/5136/pdf/sir20145136.pdf	-	Approximated from Fig. 2 of Woodward et al. (1998) and Herrera et al. (2014). Basin extent was broadened to include foothills of surrounding mountains, as a considerable number of wells exist in these areas. The Waldo and Salem Hills were used to divide this Central Willamette Valley from the Southern Willamette Valley to the south. The Chehalem Mountains were used as an approximate divide between this central portion of the aquifer system and the Tualatin Basin to the north.
Southern Willamette Valley, Willamette Valley	Woodward, D. G., Gannett, M. W., Vaccaro, J. J. (1998). Hydrogeologic framework of the Willamette Lowland aquifer system, Oregon and Washington. US Geological Survey Professional Paper 1424-B, 92 pp. Accessed February 16, 2021 from https://pubs.usgs.gov/pp/1424b/report.pdf	Herrera, N. B., Burns, E. R., Conlon, T. D. (2014). Simulation of groundwater flow and the interaction of groundwater and surface water in the Willamette Basin and Central Willamette Subbasin, Oregon. US Geological Survey Scientific Investigations Report 2014–5136, 174 pp. Accessed February 16, 2021 from https://pubs.usgs.gov/sir/2014/5136/pdf/sir20145136.pdf	-	Approximated from Fig. 2 of Woodward et al. (1998) and Herrera et al. (2014). Basin extent was broadened to include foothills of surrounding mountains, as a considerable number of wells exist in these areas. The Waldo and Salem Hills were used to divide this southern portion of the aquifer system from the Central Willamette Valley to the north.
Tualatin and Portland Basins, Willamette Valley	Woodward, D. G., Gannett, M. W., Vaccaro, J. J. (1998). Hydrogeologic framework of the Willamette Lowland aquifer system, Oregon and Washington. US Geological Survey Professional Paper 1424-B, 92 pp. Accessed February 16, 2021 from https://pubs.usgs.gov/pp/1424b/report.pdf	Herrera, N. B., Burns, E. R., Conlon, T. D. (2014). Simulation of groundwater flow and the interaction of groundwater and surface water in the Willamette Basin and Central Willamette Subbasin, Oregon. US Geological Survey Scientific Investigations Report 2014–5136, 174 pp. Accessed February 16, 2021 from https://pubs.usgs.gov/sir/2014/5136/pdf/sir20145136.pdf	Jones, M. A. (1999). Geologic framework for the Puget Sound aquifer system, Washington and British Columbia. US Geological Survey Professional Report 1424-C, 44 pp. Accessed February 25, 2021 from https://pubs.usgs.gov/pp/1424c/report.pdf	Approximated from Fig. 2 of Woodward et al. (1998) and Herrera et al. (2014). Basin extent was broadened to include foothills of surrounding mountains, as a considerable number of wells exist in these areas. The Chehalem Mountains were used as an approximate divide between this Tualatin Basin and the Central Willamette Valley to the south. The northern extent of the Basin crosses the Columbia River and is based on the approximate northern limit delineated in Fig. 1 of Jones (1999) (see page 12 or "C4" of report).

Aquifer	Reference 1	Reference 2	Reference 3	Steps taken to delineate
Wind River Basin East, Wind River Basin	Whitcomb, H.A., Lowry, M.E. (1968) Ground-water resources and geology of the Wind River Basin area, central Wyoming. U.S. Geological Survey Hydrologic Atlas 270, 14 pp. Accessed March 29, 2021 from https://pubs.usgs.gov/ha/270/report.pdf	-	-	Approximated from Plate 1 of Whitcomb and Lowry (1968). East-west basin divide approximated south of Boysen Reservoir
Wind River Basin West, Wind River Basin	Whitcomb, H.A., Lowry, M.E. (1968) Ground-water resources and geology of the Wind River Basin area, central Wyoming. U.S. Geological Survey Hydrologic Atlas 270, 14 pp. Accessed March 29, 2021 from https://pubs.usgs.gov/ha/270/report.pdf	-	-	Approximated from Plate 1 of Whitcomb and Lowry (1968). East-west basin divide approximated south of Boysen Reservoir
Abobe Creek Aquifer	Mustard, M.H., Cain, D. (1981). Hydrology and chemical quality of ground water in Kiowa County, Colorado. U.S. Geological Survey Open-File Report 81-1023, 2 plates, Accessed March 29, 2021 from https://pubs.er.usgs.gov/publication/ofr811023	-	-	Approximated from Plate 1 by Mustard and Cain (1981).
Agency Park	Van Liew, W.P., Gesink, M.L. (1985). Preliminary assessment of the ground-water resources of the alluvial aquifer, White River valley, Rio Blanco County, Colorado. U.S. Geological Survey Water-Resources Investigations Report 84-4307, 82 pp. Accessed March 29, 2021 from https://pubs.usgs.gov/wri/1984/4307/report.pdf	-	-	Approximated from plate 1 by Van Liew and Gesink (1985).
Amargosa Desert	Burbey, T. J. (1997). Hydrogeology and potential for ground-water development, carbonate-rock aquifers in southern Nevada and southeastern California. US Geological Survey Water-Resources Investigations Report 95-4168, 70 pp. Accessed March 10, 2021 from https://pubs.usgs.gov/wri/1995/4168/report.pdf	-	-	Approximated from Fig. 14 of Burbey (1997)
Animas Valley	Schwennesen, A.T., Hare, R.F. (1918). Ground water in the Animas, Playas, Hachita, and San Luis Basins, New Mexico, with analyses of water and soil. U.S. Geological Survey Water Supply Paper 422, 157 pp. Accessed March 29, 2021 from https://pubs.usgs.gov/wsp/0422/report.pdf	-	-	Approximated from Plate II of Schwennesen and Hare (1918).
Antelope Valley	Stamos, C. L., Christensen, A. H., Langenheim, V. (2017). Preliminary hydrogeologic assessment near the boundary of the Antelope Valley and El Mirage Valley groundwater basins, California. U.S. Geological Survey Scientific Investigations Report 2017–5065, 56 pp. Accessed March 20, 2021 from https://pubs.usgs.gov/sir/2017/5065/sir20175065.pdf	-	-	Approximated from Fig. 7 of Stamos et al. (2017)
Antlers Aquifer	Hart Jr., Davis, R.E. (1981). Geohydrology of the Antlers aquifer (Cretaceous), southeastern Oklahoma. U.S. Geological Survey Circular 81, 38 pp. Accessed April 5, 2021 from http://www.ogs.ou.edu/pubsscanned/Circulars/circular81mm.pdf	-	-	Approximated from Fig. 1 of Hart and Davis (1981)
Arbuckle-Simpson Aquifer	Christenson, S., Osborn, N.I., Neel, C.R., Faith, J.R., Blome, C.D., Puckette, James, Pantea, M.P. (2011). Hydrogeology and simulation of groundwater flow in the Arbuckle-Simpson aquifer, south-central Oklahoma: U.S. Geological Survey Scientific Investigations Report 2011–5029, 104 pp. Accessed April 5, 2021 from https://pubs.usgs.gov/sir/2011/5029/SIR2011-5029.pdf	-	-	Approximated from Fig. 1 of Christenson et al. (2011)
Bear Lake Valley	Graham, W. G., Campbell, L. J. (1981). Groundwater resources of Idaho. Idaho Department of Water Resources Report, 61 pp. Accessed March 23, 2021 from https://idwr.idaho.gov/files/publications/198108-MISC-GW-Resources-ID.pdf	-	-	Approximated from Plate 1 of Graham and Campbell (1981).
Bear River Bay	Price, D. (1985). Ground Water in Utah's Densely Populated Wasatch Front Area the Challenge and the Choices. U.S. Geological Survey Water-Supply Paper 2232, 78 pp. Accessed March 27, 2021 from https://pubs.usgs.gov/wsp/2232/report.pdf	-	-	Approximated from Fig. 1 of Price (1985)
Beaver Valley	Sandberg, G. W. (1966). Ground-water resources of selected basins in southwestern Utah. US Geological Survey Open File Report. 66 pp. Accessed March 7, 2021 from https://cicwcd.org/wp-content/uploads/2018/09/1966-	Lee, W.T. (1908). Water resources of Beaver Valley, Utah. U.S. Geological Survey	-	Approximated from the map on page 49 of Sandberg (1966) and plate 1 by Lee (1908)

Aquifer	Reference 1	Reference 2	Reference 3	Steps taken to delineate
	tech-pub-13-ground-water-resources-of-selected-basins-in-southwestern-utah-by-g-w-sandburg-usgs.pdf	Water Supply Paper 217, 60 pp. Accessed April 6, 2021 from https://pubs.usgs.gov/wsp/0217/report.pdf		
Big Bear Valley	Flint, L. E., Brandt, J., Christensen, A. H.,Flint, A.L., Hevesi, J.A., Jachens, R., Kulongoski, J.T., Martin, P., Sneed, M. (2012). Geohydrology of Big Bear Valley, California: phase 1--Geologic framework, recharge, and preliminary assessment of the source and age of groundwater. US Geological Survey Scientific Investigations Report 2012-5100, 122 pp. Accessed March 14, 2021 from https://pubs.usgs.gov/sir/2012/5100/pdf/sir20125100.pdf	-	-	Approximated from Fig. 1 of Flint et al. (2012)
Big Chino Valley	Langenheim, V. E., Duval, J. S., Wirt, L., DeWitt, E. (2000). Preliminary report on geophysics of the Verde River headwaters region, Arizona: US Geological Survey Open-File Report 00-403, 28 pp. Accessed March 12, 2021 from https://pubs.usgs.gov/of/2000/0403/pdf/of00-403p.pdf	Kennedy, J.R., Kahler, L.M., Read, A.L. (2019). Aquifer storage change and storage properties, 2010–2017, in the Big Chino Subbasin, Yavapai County, Arizona: U.S. Geological Survey Scientific Investigations Report 2019–5060, 39 pp. Accessed November 29, 2021 from https://pubs.usgs.gov/sir/2019/5060/sir20195060.pdf	-	Approximated from map on pp. 12 of Langenheim et al. (2000) and Fig. 2 by Kennedy et al. (2019)
Big Sandy - Rush Creek Aquifer	Mustard, M.H., Cain, D. (1981). Hydrology and chemical quality of ground water in Kiowa County, Colorado. U.S. Geological Survey Open-File Report 81-1023, 2 plates, Accessed March 29, 2021 from https://pubs.er.usgs.gov/publication/ofr811023	-	-	Approximated from Plate 1 by Mustard and Cain (1981).
Big Sandy Valley	Morrison, R.B. (1940). Ground-water resources of the Big Sandy Valley, Mohave County, Arizona. U.S. Geological Survey Report. 8 pp. Accessed April 15, 2021 from https://azmemory.azlibrary.gov/digital/collection/statepubs/id/6455/	-	-	Approximated from "Map of Big Sandy Valley" on page 8 of Morrison (1940).
Big Smoky Valley	Meinzer, O. E. (1916b). Ground water in Big Smoky Valley, Nevada. Water Supply Paper 375-D. 34 pp. Accessed March 23, 2021 from https://pubs.usgs.gov/wsp/0375d/report.pdf	-	-	Approximated from Plate 6 (page 7 of 34 in document) of Meinzer (1916b).
Bighorn Basin	Tauchen, P, Bartos, T.T., Clarey, K.E., Quillinan, S.A., Hallberg, L.L., Clark, M.L., Thompson, M., Gribb, N., Worman, B., Gracias, T. (2012). Wind/Bighorn River Basin Water Plan Update Groundwater Study Level 1 (2008–2011). Groundwater Determination. Wyoming Water Development Commission Technical Memorandum, 397 pp. Accessed March 29, 2021 from https://waterplan.state.wy.us/plan/bighorn/2010/gw-finalrept/gw-finalrept.pdf	Fisher, C.A. (1906). Geology and water resources of the Bighorn Basin, Wyoming. US Geological Survey Professional Paper 53, 97 pp. Accessed November 29, 2021 from https://pubs.usgs.gov/pp/0053/report.pdf	-	Approximate from maps in Chapter 3 of Tauchen et al. (2012) and Plate XII by Fisher (1906)
Biscayne Aquifer	Wacker, M.A., Cunningham, K.J., Williams, J.H. (2014). Geologic and hydrogeologic frameworks of the Biscayne aquifer in central Miami-Dade County, Florida: U.S. Geological Survey Scientific Investigations Report 2014–5138, 66 pp. Accessed March 31, 2021 from https://pubs.usgs.gov/sir/2014/5138/pdf/sir2014-5138.pdf	-	-	Approximated from Fig. 1 of Wacker et al. (2014).
Black Hills Uplift	Driscoll, D. G., Carter, J. M., Williamson, J. E., Putnam, L. D. (2002). Hydrology of the Black Hills area, South Dakota. US Geological Survey Water-Resources Investigations Report 2002-4094, 158 pp. Accessed February 16, 2021 from https://pubs.usgs.gov/wri/wri024094/pdf/wri024094.pdf	Back, W., Hanshaw, B. B., Plummer, L. N., Rahn, P. H., Rightmire, C. T., Rubin, M. (1983). Process and rate of dedolomitization: mass transfer and 14C dating in a regional	-	Approximated from Fig. 2 of Driscoll et al. (2002) and Fig. 1 by Back et al. (1983)

Aquifer	Reference 1	Reference 2	Reference 3	Steps taken to delineate
		carbonate aquifer. Geological Society of America Bulletin, 94, 1415-1429.		
Black Warrior River Aquifer System (Eutaw and McShan Formations and Tuscaloosa Group)	Kidd, R. E., Lambeth, D. S. (1995). Hydrogeology and ground-water quality in the Black Belt area of west-central Alabama, and estimated water use for aquaculture, 1990 (Vol. 94, No. 4074). US Department of the Interior, US Geological Survey. Accessed February 10, 2021 via https://citeseerx.ist.psu.edu/viewdoc/download?doi=10.1.1.1015.2227&rep=rep1&type=pdf	Strom, E. W., Mallory, M. J. (1995). Hydrogeology and simulation of ground-water flow in the Eutaw-McShan Aquifer and in the Tuscaloosa aquifer system in northeastern Mississippi. US Geological Survey Water-Resources Investigations Report 94-4223, 89 pp. Accessed November 29, 2021 from https://pubs.usgs.gov/wri/1994/4223/report.pdf	Miller, J.A. (1990). Ground Water Atlas of the United States: Segment 6, Alabama, Florida, Georgia, South Carolina. U.S. Geological Survey Hydrologic Atlas 730-G, 30 pp. Accessed April 5, 2021 from https://www.nrc.gov/docs/ML1706/ML17060B027.pdf	Mississippi portion approximated from Fig. 8 of Strom and Mallory (1995). Alabama portion approximated from Fig. 3 of Kidd and Lambeth (1990) and Fig. 73 by Miller (1990) for easternmost extent. Best estimates were made to connect these two separate delineations in the northwest portion of the aquifer's extent within Alabama.
Blue River Basin	Barkmann, P. E., Broes, L.D., Palkovic, M.J., Hopkins, J.C., Bird, K.S., Sebol, L.A., Fitzgerald. F.S. (2020). ON-010 Colorado Groundwater Atlas. Geohydrology. Colorado Geological Survey, Golden, CO. ON-010 Colorado Groundwater Atlas. Accessed March 29, 2021 from https://coloradogeologicalsurvey.org/water/colorado-groundwater-atlas/	-	-	Approximated from Fig. 12-01 of Barkmann et al. (2020).
Boulder Valley	Butler, J.A. Bobst, A.L. (2017). Hydrogeologic investigation of the Boulder Valley, Jefferson County, Montana. Montana Bureau of Mines and Geology Open-File Report 688, 140 pp. Accessed March 29, 2021 from http://mbmg.mtech.edu/pdf-open-files/mbmg688_web.pdf	-	-	Approximated from Fig. 2 of Butler and Bobst (2017).
Boulder Valley in northern Nevada	Bredehoeft, J.D., Farvolden, R.N. (1963). International Association of Scientific Hydrology, Commission of Subterranean Waters, Publication no. 64, p. 197–212. Accessed March 23, 2021 from http://hydrologie.org/redbooks/a064/064017.pdf	-	-	Approximated from Fig. 1 of Bredehoft and Farvolden (1963)
Buena Vista Valley in northern Nevada	Welch, A. H., Sorey, M. L., Olmsted, F. H. (1981). Hydrothermal system in Southern Grass Valley, Pershing County, Nevada. U.S. Geological Survey Open-File Report 81-915, 200 pp. Accessed March 22, 2021 from https://www.osti.gov/servlets/purl/5119283-5mJ8YB/	-	-	Approximated from Fig. 2 of Welch et al. (1981)
Buffalo Valley in northern Nevada	Welch, A. H., Sorey, M. L., Olmsted, F. H. (1981). Hydrothermal system in Southern Grass Valley, Pershing County, Nevada. U.S. Geological Survey Open-File Report 81-915, 200 pp. Accessed March 22, 2021 from https://www.osti.gov/servlets/purl/5119283-5mJ8YB/	-	-	Approximated from Fig. 2 of Welch et al. (1981)
Butler Valley	Tillman, F.D, Cordova, J.T., Leake, S.A., Thomas, B.E., Callegary, J.B. (2011). Water availability and use pilot: methods development for a regional assessment of groundwater availability, southwest alluvial basins, Arizona. U.S. Geological Survey Scientific Investigations Report 2011-5071, 132 pp. Accessed April 15, 2021 from https://pubs.usgs.gov/sir/2011/5071/sir2011-5071_text.pdf	-	-	Approximated from Fig. 2 of Tillman et al. (2011); boundaries also informed by the density of wells recorded in Arizona's well completion dataset.
Cache Valley	Graham, W. G., Campbell, L. J. (1981). Groundwater resources of Idaho. Idaho Department of Water Resources Report, 61 pp. Accessed March 23, 2021 from https://idwr.idaho.gov/files/publications/198108-MISC-GW-Resources-ID.pdf	Bjorklund, L. J., McGreevy, L. J. (1971). Ground-water resources of Cache Valley, Utah and Idaho. Utah Department of Natural Resources, Division of Water Rights Technical Publication No. 36, 85 pp. Accessed March 27, 2021 from	-	Approximated from Plate 1 of Graham and Campbell (1981) and Fig. 1 of Bjorklund and McGreevy (1971).

Aquifer	Reference 1	Reference 2	Reference 3	Steps taken to delineate
		https://waterrights.utah.gov/docSys/v920/w920/w920008y.pdf		
Camas Prairie	Graham, W. G., Campbell, L. J. (1981). Groundwater resources of Idaho. Idaho Department of Water Resources Report, 61 pp. Accessed March 23, 2021 from https://idwr.idaho.gov/files/publications/198108-MISC-GW-Resources-ID.pdf	-	-	Approximated from Plate 1 of Graham and Campbell (1981).
Canadian River Alluvial Aquifer	Ellis, J.H., Mashburn, S.L., Graves, G.M., Peterson, S.M., Smith, S.J., Fuhrig, L.T., Wagner, D.L., Sanford, J.E. (2017). Hydrogeology and simulation of groundwater flow and analysis of projected water use for the Canadian River alluvial aquifer, western and central Oklahoma. U.S. Geological Survey Scientific Investigations Report 2016–5180, 64 pp. Accessed April 5, 2021 from https://pubs.usgs.gov/sir/2016/5180/sir20165180.pdf	-	-	Approximated from Fig. 1 of Ellis et al. (2017).
Cape Cod Aquifer	Frimpter, M.H., Gay, F.B. (1979). Chemical quality of ground water on Cape Cod, Massachusetts. U.S. Geological Survey Water-Resources Investigations Report 79-65, 20 pp. Accessed April 14, 2021 from https://pubs.usgs.gov/wri/1979/0065/report.pdf	Masterson, J.P., Walter, D.A. (2009). Hydrogeology and groundwater resources of the coastal aquifers of southeastern Massachusetts. U.S. Geological Survey Circular 1338, 16 pp. Accessed April 14, 2021 from https://pubs.usgs.gov/circ/circ1338/pdf/circular%202009-1338_508.pdf	-	Approximated from map on title page of Frimpter and Gay (1979) and Fig. 1 of Masterson and Walter (2009).
Carpenteria Basin	La Rocque, G.A., Upson, J. E., Worts Jr., G. F. (1950). Wells and water levels in principal ground-water basins in Santa Barbara County, California – Part 1. Carpenteria, Goleta and Santa Ynez Valleys, 1930-41. U.S. Geological Survey Water-SupplyPaper 1068. 465 pp. Accessed March 20, 2021 from https://pubs.usgs.gov/wsp/1068/report.pdf	-	-	Approximated from Plate 1 of La Rocque et al. (1968)
Castle Hayne Aquifer	Lyke, W.L., Coble, R.W. (1987). Regional study of the Castle Hayne Aquifer of eastern North Carolina. U.S. Geological Survey Open-File Report 87-571, 2 pp. Accessed April 1, 2021 from https://pubs.usgs.gov/of/1987/0571/report.pdf	Eimers, J.L. Daniel III, C.C., Coble, R.W. (1994). Hydrogeology and simulation of ground-water flow at U.S. Marine Corps Air Station, Cherry Point, North Carolina, 1987-90. Water-Resources Investigations Report 94-4186, 81 pp. Accessed April 1, 2021 from https://pubs.usgs.gov/wri/1994/4186/report.pdf	Winner Jr., M.D., Coble, R.W. (1989). Hydrogeologic framework of the North Carolina Coastal Plain aquifer system. U.S. Geological Survey Report 87-690, 167 pp. Accessed April 1, 2021 from https://pubs.usgs.gov/of/1987/0690/report.pdf	Approximated from Fig. 1 of Lyke and Coble (1987) and Fig. 14 of Winner Jr. and Coble (1989). Eastern limit of Castle Hayne is extended farther than shown in Fig. 14 of Winner Jr and Coble (1989) as local reports near Cherry Point detail the importance of the Castle Hayne Aquifer -- see Eimers et al. (1994).
Cedar Mesa	Kolm, K.E., van der Heijde, P.K.M. (2014). Groundwater Systems In Delta County, Colorado: Surface Creek Valley Area. Report prepared for Delta County Board of County Commissioners, Colorado, 64 pp. Accessed March 28, 2021 from https://www.chc4you.org/wp-content/uploads/2017/01/Surface-Creek-Hydrology-Report-2014.pdf	-	-	Approximated from Fig. 23a of Kohm and van der Heijde (2014)
Cedar Valley	Brooks, L. E., Mason, J. L. (2005). Hydrology and simulation of ground-water flow in Cedar Valley, Iron County, Utah (No. 2005-5170). US Geological Survey Scientific Investigations Report 2005-5170, 127 pp. Accessed March 7, 2021 from https://pubs.usgs.gov/sir/2005/5170/PDF/SIR2005_5170.pdf	-	-	Approximated from Fig. 1 of Brooks et al. (2005)
Cedar Valley near Utah Lake	Jordan, J. L. (2013). Aquifer Parameter Estimation from Aquifer Tests and Specific-capacity Data in Cedar Valley and the Cedar Pass Area, Utah County, Utah. Utah Geological Survey Special Study 146.	-	-	Approximated from Fig. 1 of Jordan (2013)
Central Hachita Valley	Trauger, F. D., Herrick, E. H. (1962). Ground Water in Central Hachita Valley Northeast of the Big Hatchet Mountains, Hidalgo County, New Mexico. New	-	-	Approximated from map on page 3 of Trauger and Herrick (1961).

Aquifer	Reference 1	Reference 2	Reference 3	Steps taken to delineate
	Mexico State Engineer Office Technical Report 26, 26 pp. Accessed March 27, 2021 from https://www.ose.state.nm.us/Library/TechnicalReports/TechReport-026.pdf			
Central Minnesota Surficial and Buried Sand and Gravel Aquifers	Minnesota Department of Natural Resources (2021). Minnesota Groundwater Provinces 2021. Minnesota Department of Natural Resources map, 2 pp. Accessed April 14, 2021 from https://files.dnr.state.mn.us/waters/groundwater_section/provinces/2021-provinces.pdf	-	-	Approximated from map by the Minnesota Department of Natural Resources (2021)
Central Raton Basin	Geldon, A. L. (1989). Ground-water hydrology of the central Raton Basin, Colorado and New Mexico. U.S. Geological Survey Water-Supply Paper 2288, 90 pp. Accessed March 29, 2021 from https://pubs.usgs.gov/wsp/2288/report.pdf	Watts, K.R. (2006). Hydrostratigraphic framework of the Raton, Vermejo, and Trinidad aquifers in the Raton Basin, Las Animas County, Colorado: U.S. Geological Survey Scientific Investigations Report 2006–5129, 31 pp. Accessed March 30, 2021 from https://pubs.usgs.gov/sir/2006/5129/pdf/SIR06-5129_508.pdf	-	Approximated from Fig. 1 of Geldon (1989), with some guidance from Fig. 1 of Watts (2006)
Central Sevier Valley	Young, R. A., Carpenter, C. H. (1965). Ground-water conditions and storage in the Central Sevier Valley, Utah. US Geological Survey Water Supply Paper 1787. 106 pp. Accessed March 7, 2021 from https://pubs.usgs.gov/wsp/1787/report.pdf	-	-	Approximated from Plate 1 of Young and Carpenter (1965)
Cienega Basin	Flora, S., Davis, T. (2009). Hydrologic Map Series (HMS), Water Level Change Map Series (WLCMS), and Basin Sweep Assessment Report ADWR Basins and Sub-Basins. Arizona Department of Water Resources Hydrology Division Field Services Section https://new.azwater.gov/sites/default/files/HMSWLCMSBasinSweepAssessmentReport2009.pdf	-	-	Ciénega Basin approximated from Fig. 1 of Flora and Davis (2009). Hydrologic Map Series (HMS), Water Level Change Map Series (WLCMS), and Basin Sweep Assessment Report ADWR Basins and Sub-Basins. Arizona Department of Water Resources Hydrology Division Field Services Section https://new.azwater.gov/sites/default/files/HMSWLCMSBasinSweepAssessmentReport2009.pdf
Cimarron Basin	Adams, G.P., Bergman, D.L. (1996). Geohydrology of alluvium and terrace deposits of the Cimarron River from freedom to Guthrie, Oklahoma. U.S. Geological Survey Water-Resources Investigations Report 95-4066, 63 pp. Accessed April 5, 2021 from https://pubs.usgs.gov/wri/1995/4066/report.pdf	-	-	Approximated from Fig. 1 of Adams and Bergman (1996).
Clover Valley	Berger, D. L. (2006). Hydrogeology and Water Resources of Ruby Valley, Northeastern Nevada. Scientific Investigations Report 2005-5247, 48 pp. Accessed March 23, 2021 from https://pubs.usgs.gov/sir/2005/5247/sir2005-5247.pdf	-	-	Approximated from Fig. 1 of Berger (2006).
Coachella Valley	Sneed, M., Brandt, J.T., Solt, M. (2014). Land subsidence, groundwater levels, and geology in the Coachella Valley, California, 1993–2010: U.S. Geological Survey, Scientific Investigations Report 2014–5075, 62 pp. Accessed February 15, 2021 from https://pubs.usgs.gov/sir/2014/5075/pdf/sir2014-5075.pdf	-	-	Approximated from Figs. 1 and 2 of Sneed et al. (2014)
Coal Oil Basin	Van Liew, W.P., Gesink, M.L. (1985). Preliminary assessment of the ground-water resources of the alluvial aquifer, White River valley, Rio Blanco County, Colorado. U.S. Geological Survey Water-Resources Investigations Report 84-4307, 82 pp. Accessed March 29, 2021 from https://pubs.usgs.gov/wri/1984/4307/report.pdf	-	-	Approximated from plate 1 by Van Liew and Gesink (1985).
Coastal Maine Crystalline Aquifers	Harte, P.T., Robinson, G.R., Jr., Ayotte, J.D., Flanagan, S.F. (2008). Framework for evaluating water quality of the New England crystalline rock aquifers. U.S.	-	-	Approximated from Fig. 4 and "Coastal Maine" geologic province by Harte et al. (2008).

Aquifer	Reference 1	Reference 2	Reference 3	Steps taken to delineate
	Geological Survey Open-File Report 2008–1282, 47 pp. Accessed April 15, 2021 from https://pubs.usgs.gov/of/2008/1282/pdf/ofr2008-1282.pdf			
Connecticut Valley	Olcott, P.G. (1995). Ground Water Atlas of the United States: Segment 12, Connecticut, Maine, Massachusetts, New Hampshire, New York, Rhode Island, Vermont. U.S. Geological Survey Hydrologic Atlas 730-M, 30 pp. Accessed April 14, 2021 from https://pubs.usgs.gov/ha/730m/report.pdf	Connecticut Department of Energy and Environmental Protection (2021). Webpage entitled "Overview of the Ground Water Flow System in Connecticut". Accessed April 14, 2021 from https://portal.ct.gov/DEEP/Aquifer-Protection-and-Groundwater/Ground-Water/Ground-Water-Flow-System-in-Connecticut	-	Approximated from map displayed at top of webpage by Connecticut Department of Energy and Environmental Protection (2021) and also from Fig. 102 of Olcott (1995).
Coyote Springs Valley	Burbey, T. J. (1997). Hydrogeology and potential for ground-water development, carbonate-rock aquifers in southern Nevada and southeastern California. US Geological Survey Water-Resources Investigations Report 95-4168, 70 pp. Accessed March 10, 2021 from https://pubs.usgs.gov/wri/1995/4168/report.pdf	-	-	Approximated from Fig. 7 of Burbey (1997)
Crescent Valley in northern Nevada	Bredehoeft, J.D., Farvolden, R.N. (1963). International Association of Scientific Hydrology, Commission of Subterranean Waters, Publication no. 64, p. 197–212. Accessed March 23, 2021 from http://hydrologie.org/redbooks/a064/064017.pdf	-	-	Approximated from Fig. 1 of Bredehoft and Farvolden (1963)
Curlew Valley	Graham, W. G., Campbell, L. J. (1981). Groundwater resources of Idaho. Idaho Department of Water Resources Report, 61 pp. Accessed March 23, 2021 from https://idwr.idaho.gov/files/publications/198108-MISC-GW-Resources-ID.pdf	-	-	Approximated from Plate 1 of Graham and Campbell (1981).
Cuyama Valley	Everett, R.R., Gibbs, D.R., Hanson, R.T., Sweetkind, D.S., Brandt, J.T., Falk, S.E., Harich, C.R. (2013). Geology, water-quality, hydrology, and geomechanics of the Cuyama Valley groundwater basin, California, 2008–12: U.S. Geological Survey Scientific Investigations Report 2013–5108, 62 pp. Accessed March 7, 2021 from https://pubs.usgs.gov/sir/2013/5108/pdf/sir2013-5108.pdf	Sweetkind, D. S., Faunt, C. C., Hanson, R. T. (2013). Construction of 3-D geologic framework and textural models for Cuyama Valley groundwater basin, California. US Geological Survey Scientific Investigations Report 2013-5127. 58 pp. Accessed March 7, 2021 from https://pubs.usgs.gov/sir/2013/5127/pdf/sir2013-5127.pdf	-	Approximated from Fig. 1 of Everett et al. (2013) and Fig. 2 of Sweetkind et al. (2013)
Dakota Aquifer System	Leonard, R. B., Signor, D. C., Jorgensen, D. G., Helgesen, J. O. (1983). Geohydrology and hydrochemistry of the Dakota Aquifer, central United States. Journal of the American Water Resources Association, 19(6), 903-912.	Bredehoeft, J. D., Neuzil, C. E., Milly, P. C. D. (1983). Regional flow in the Dakota aquifer: A study of the role of confining layers. US Geological Survey Water-Supply Paper 2237, 50 pp. Accessed on February 9, 2021 from https://pubs.er.usgs.gov/publication/wsp2237	Macfarlane, P. A., Doveton, J. H., Whittemore, D. O. (1998). User's Guide to the Dakota Aquifer in Kansas. Kansas Geological Survey. Accessed February 9, 2021 from http://www.kgs.ku.edu/Publications/Bulletins/TS2/index.html	Approximated the complete boundary of the Dakota Formation from Fig. 6 of Leonard et al. (1983). Other portions of the Dakota Aquifer were digitized from page 30 of Prior et al. (2003; Iowa portion) and Macfarlane et al. (1998; for eastern margin of Kansas' Dakota Aquifer (Fig. 2 of Macfarlane et al., 1998) and general boundaries extending into Canada (Fig. 1 of Macfarlane et al., 1998)). This specific portion of the Dakota was delineated by exploring well completion depths, mainly drawing from well completion records for South Dakota (specifically, records of deep wells).

Aquifer	Reference 1	Reference 2	Reference 3	Steps taken to delineate
Deep Creek Valley	Gardner, P. M., Masbruch, M. D. (2015). Hydrogeologic and geochemical characterization of groundwater resources in Deep Creek Valley and adjacent areas, Juab and Tooele Counties, Utah, and Elko and White Pine Counties, Nevada. US Geological Survey Scientific Investigations Report 2015–5097, 66 pp. Accessed March 10, 2021 from https://pubs.usgs.gov/sir/2015/5097/sir20155097.pdf	-	-	Approximated from Fig. 6 of Gardner and Masbruch (2015)
Denver Basin	Ruybal, C. J., Hogue, T. S., McCray, J. E. (2019). Assessment of groundwater depletion and implications for management in the Denver Basin Aquifer System. JAWRA Journal of the American Water Resources Association, 55, 1130-1148.	Malenda, H.F., Penn, C.A., (2020). Groundwater levels in the Denver Basin bedrock aquifers of Douglas County, Colorado, 2011–19. U.S. Geological Survey Scientific Investigations Report 2020–5076, 44 pp., Accessed November 29, 2021 from https://pubs.usgs.gov/sir/2020/5076/sir20205076.pdf	Paschke, S.S. (2011). Groundwater availability of the Denver Basin aquifer system, Colorado. U.S. Geological Survey Professional Paper 1770, 274 pp. Accessed January 21, 2022 from https://pubs.usgs.gov/pp/1770/contents/pp1770.pdf	Approximated from Fig. A4 of Paschke et al. (2011), Fig. 1 by Malenda and Penn (2020), and Fig. 1 by Ruybal et al. (2019)
Desert Valley in northern Nevada	Lopes, T. J. (2010). Hydrologic Evaluation of the Jungo Area, Southern Desert Valley, Nevada. U. S. Geological Survey Open-File Report 2010-1009, 18 pp. Accessed March 23, 2021 from https://pubs.usgs.gov/of/2010/1009/pdf/ofr20101009.pdf	-	-	Approximated from Fig. 2 of Lopes (2010)
Detrital Valley Basin	Tillman, F.D, Garner, B.D., Truini, M. (2013). Preliminary groundwater flow model of the basin-fill aquifers in Detrital, Hualapai, and Sacramento Valleys, Mohave County, northwestern Arizona. U.S. Geological Survey Scientific Investigations Report 2013–5122, 52 pp. http://pubs.usgs.gov/sir/2013/5122/	-	-	Approximated from Fig. 2 by Tillman et al. (2013).
Diamond Valley in northern Nevada	Bredehoeft, J.D., Farvolden, R.N. (1963). International Association of Scientific Hydrology, Commission of Subterranean Waters, Publication no. 64, p. 197–212. Accessed March 23, 2021 from http://hydrologie.org/redbooks/a064/064017.pdf	-	-	Approximated from Fig. 1 of Bredehoft and Farvolden (1963)
Dry Lake Valley and Dreamer Valley	Hurlow, H. A. (2014). Hydrogeologic studies and groundwater monitoring in Snake Valley and adjacent hydrographic areas, west-central Utah and east-central Nevada (Vol. 135). Utah Geological Survey Bulletin 135, 272 pp.	-	-	Approximated from map on page 6 of Hurlow (2014)
Duck Valley	Graham, W. G., Campbell, L. J. (1981). Groundwater resources of Idaho. Idaho Department of Water Resources Report, 61 pp. Accessed March 23, 2021 from https://idwr.idaho.gov/files/publications/198108-MISC-GW-Resources-ID.pdf	-	-	Approximated from Plate 1 of Graham and Campbell (1981).
East Shore Area	Price, D. (1985). Ground Water in Utah's Densely Populated Wasatch Front Area the Challenge and the Choices. U.S. Geological Survey Water-Supply Paper 2232, 78 pp. Accessed March 27, 2021 from https://pubs.usgs.gov/wsp/2232/report.pdf	-	-	Approximated from Fig. 1 of Price (1985)
Eastern Champlain Valley Lowlands	Nystrom, E.A. (2006). Ground-water quality in the Lake Champlain Basin, New York, 2004. U.S. Geological Survey Open-File Report 2006-1088, 22 pp. Accessed April 15, 2021 from https://pubs.usgs.gov/of/2006/1088/pdf/Nystrom.OFR2006-1088.pdf	Scott, T.-M., Nystrom, E.A., Reddy, J.E. (2016). Groundwater quality in the Lake Champlain and Susquehanna River basins, New York, 2014. U.S. Geological Survey Open-File Report 2016–1153, 33 pp. Accessed April 15, 2021 from https://pubs.usgs.gov/of/2016/1153/ofr20161153.pdf	-	Approximated from Fig. 2 of Nystrom (2006) and Fig. 1 by Scott et al. (2016).

Aquifer	Reference 1	Reference 2	Reference 3	Steps taken to delineate
Eastern Dakota Aquifer	Leonard, R. B., Signor, D. C., Jorgensen, D. G., Helgesen, J. O. (1983). Geohydrology and hydrochemistry of the Dakota Aquifer, central United States. Journal of the American Water Resources Association, 19(6), 903-912.	Prior, J.C., Boekhoff, J.L., Howes, M.R., Libra, R.D., VanDorpe, P.E. (2003). Iowa's Groundwater Basics. Iowa Department of Natural Resources Report, 92 pp. Accessed November 29, 2021 from https://s-iihr34.iihr.uiowa.edu/publications/uploads/2014-08-24_08-08-21_es-06.pdf	-	Approximated from Fig. 9 of Leonard et al. (1983) and page 30 of Prior et al. (2003)
Eastern Kankakee Plains	Fenneman, N.M., Johnson, D.W. (1946). Physiographic divisions of the conterminous United States. U.S. Geological Survey map, 1:7,000,000 Scale.	Leighton, M. M., Ekblaw, G. E., Horberg, L. (1948). Physiographic divisions of Illinois. The Journal of Geology, 56, 16-33.	Gray, H.H. (2001). Map of Indiana showing physiographic divisions. Indiana Geological Survey Miscellaneous Map 69, 15 pp. Accessed April 12, 2021 from https://igws.indiana.edu/ReferenceDocs/Maps/PhysiographicRegions.pdf	Broader Till Plain physiographic region approximated from Fenneman and Johnson (1946). Kankakee Plain in Illinois approximated from Figs. 1 and 2 of Leighton et al. (1948); Indiana portion of subarea approximated from Gray (2001). Western margin of subarea approximated at northwest margin of the Iroquois Till Plains (located to the southwest of this subarea)
Elk City Aquifer	Becker, C.J., Runkle, D., Rea, A. (1997b). Digital data sets that describe aquifer characteristics of the Elk City Aquifer in western Oklahoma. U.S. Geological Survey Open-File Report 96-449. Accessed April 5, 2021 from https://pubs.usgs.gov/of/1996/ofr96-449/	-	-	Approximated from map by Becker et al. (1997b) displayed at: https://pubs.usgs.gov/of/1996/ofr96-449/ (accessed April 5, 2021)
Enid Isolated Terrace Aquifer	Becker, C. J., Runkle, D., Rea, A. (1997a). Digital Data Sets that Describe Aquifer Characteristics of the Enid Isolated Terrace Aquifer in Northwestern Oklahoma. U.S. Geological Survey Open-File Report, 96-450. Accessed April 5, 2021 from https://pubs.usgs.gov/of/1996/ofr96-450/	-	-	Approximated from map by Becker et al. (1997a) available at https://pubs.usgs.gov/of/1996/ofr96-450/
Estancia Basin	Land, L. (2016). Overview of Fresh and Brackish Water Quality in New Mexico. Open file Report 583. 4 pp. Accessed February 17, 2021 from https://geoinfo.nmt.edu/resources/water/amp/brochures/BWA/Estancia_Basin_FBWQNM.pdf	-	-	Approximated from Map by Land (2016)
Eureka and Eel River and Mad River Plains	Johnson, M. J. (1975). Ground-water conditions in the Eureka Area, Humboldt County, California. U.S. Geological Survey Water-Resources Investigations 78-127. 51 pp. Accessed March 20, 2021 from https://pubs.usgs.gov/wri/1978/0127/report.pdf	-	-	Approximated from Fig. 1 of Johnson (1975)
Fish Lake Valley	Reheis, M.C., Block, D. (2007). Surficial geologic map and geochronologic database, Fish Lake Valley, Esmeralda County, Nevada, and Mono County, California. U.S. Geological Survey Data Series 277, 14 pp. Accessed March 23, 2021 from https://pubs.usgs.gov/ds/277/report.pdf	-	-	Approximated from Fig. 1 of Reheis and Block (2007)
Fort Rock Basin	Miller, D.W. (1984). Appraisal of ground water conditions in the Fort Rock Basin, Lake County, Oregon. Oregon Water Resources Department Open-File Report, 78 pp. Accessed February 24, 2021 from https://digital.osl.state.or.us/islandora/object/osl%3A13403/datastream/OBJ/view	Oregon Water Resources Department (2021). Well Report Query. Web Page Accessed February 24, 2021 from https://apps.wrd.state.or.us/apps/gw/well_log/Default.aspx	-	Approximated from Fig.1 of Miller (1984) and influenced by the spatial distribution of groundwater wells recorded by the Oregon Water Resources Department (2021).
Fountain Creek Alluvial Aquifer	Radell, MJ., Lewis, M.E., Watts, K.R. (1994). Hydrogeologic characteristics of the alluvial aquifer and adjacent deposits of the Fountain Creek valley, El Paso County, Colorado. U.S. Geological Survey Water-Resources Investigations Report 94-4129, 3 maps and 11 cross sections, Accessed March 29, 2021 from https://pubs.er.usgs.gov/publication/wri944129	-	-	Approximated from Plate 1 of Radell et al. (1994).

Aquifer	Reference 1	Reference 2	Reference 3	Steps taken to delineate
Gallatin Valley	Kendy, E. (2001). Ground-water resources of the Gallatin Local Water Quality District, southwestern Montana. US Geological Survey Fact Sheet 007-01, 4 pp. Accessed March 29, 2021 from https://pubs.usgs.gov/fs/2001/0007/report.pdf	-	-	Approximated from Fig. 1 of Kendy (2001).
Garber-Wellington Aquifer	Mashburn, S.L., Ryter, D.W., Neel, C.R., Smith, S.J., Correll, J.S. (2014). Hydrogeology and simulation of ground-water flow in the Central Oklahoma (Garber-Wellington) Aquifer, Oklahoma, 1987 to 2009, and simulation of avail-able water in storage, 2010–2059. U.S. Geological Survey Scientific Investigations Report 2013–5219, 92 pp. Accessed April 5, 2021 from https://pubs.usgs.gov/sir/2013/5219/pdf/sir20135219_v2.0.pdf	-	-	Approximated from Fig. 4 of Mashburn et al. (2014)
Garden Valley	Graham, W. G., Campbell, L. J. (1981). Groundwater resources of Idaho. Idaho Department of Water Resources Report, 61 pp. Accessed March 23, 2021 from https://idwr.idaho.gov/files/publications/198108-MISC-GW-Resources-ID.pdf	-	-	Approximated from Plate 1 of Graham and Campbell (1981).
Gem Valley	Graham, W. G., Campbell, L. J. (1981). Groundwater resources of Idaho. Idaho Department of Water Resources Report, 61 pp. Accessed March 23, 2021 from https://idwr.idaho.gov/files/publications/198108-MISC-GW-Resources-ID.pdf	-	-	Approximated from Plate 1 of Graham and Campbell (1981).
Gila Bend Basin	Tillman, F.D, Cordova, J.T., Leake, S.A., Thomas, B.E., Callegary, J.B. (2011). Water availability and use pilot: methods development for a regional assessment of groundwater availability, southwest alluvial basins, Arizona. U.S. Geological Survey Scientific Investigations Report 2011-5071, 132 pp. Accessed April 15, 2021 from https://pubs.usgs.gov/sir/2011/5071/sir2011-5071_text.pdf	-	-	Approximated from Fig. 2 of Tillman et al. (2011); boundaries also informed by the density of wells recorded in Arizona's well completion dataset.
Gila Valley	Knechtel, M. M., Lohr, E. W. (1938). Geology and ground-water resources of the Valley of Gila River and San Simon Creek, Graham County, Arizona; with a section on the Chemical character of the ground water. U.S. Geological Survey Water-Supply Paper 796-F, 67 pp. Accessed March 29, 2021 from https://pubs.usgs.gov/wsp/0796f/report.pdf	-	-	Approximated from map on page 10 of Knechtel and Lohr (1938).
Goleta Basin	Bachman, S. (2010). Goleta Groundwater Basin Groundwater Management Plan. 91 pp. Accessed March 20,2021 from https://www.goletawater.com/assets/uploads/documents/groundwater-management/Groundwater_Management_Plan_Final_05-11-10.pdf	-	-	Approximated from Fig. 1-1 of Bachman (2010)
Goshen Valley	Brooks, L.E. (2013). Evaluation of the groundwater flow model for southern Utah and Goshen Valleys, Utah, updated to conditions through 2011, with new projections and groundwater management simulations: U.S. Geological Survey Open-File Report 2013–1171, 35 pp. Accessed March 7, 2021 from https://pubs.usgs.gov/of/2013/1171/pdf/ofr2013-1171.pdf	-	-	Approximated from Fig. 1 of Brooks (2013)
Grand Forks Aquifer	Wei, M., Allen, D. M., Carmichael, V., Ronneseth, K. (2010). State of understanding of the hydrogeology of the Grand Forks aquifer. Water Stewardship Division, BC Ministry of Environment Report, 99 pp. Accessed March 15, 2021 from https://www.grandforks.ca/wp-content/uploads/reports/2010-Hydrogeology-Study-of-Grand-Forks-area.pdf	-	-	Approximated from Fig. 2 of Wei et al. (2010)
Grand Valley	Lohman, S.W. (1965). Geology and Artesian Water Supply Grand Junction Area Colorado. U.S. Geological Survey Professional Paper 451, 157 pp. Accessed March 28, 2021 from https://pubs.usgs.gov/pp/0451/report.pdf	-	-	Approximated from Plate 1 of Lohman (1965)
Grass Valley in northern Nevada	Welch, A. H., Sorey, M. L., Olmsted, F. H. (1981). Hydrothermal system in Southern Grass Valley, Pershing County, Nevada. U.S. Geological Survey Open-File Report 81-915, 200 pp. Accessed March 22, 2021 from https://www.osti.gov/servlets/purl/5119283-5mJ8YB/	-	-	Approximated from Fig. 2 of Welch et al. (1981)
Great Plains Dakota Aquifer	Whittlemore, D.O., Macfarlane, P.A., Wilson, B.B. (2014). Water Resources of the Dakota Aquifer in Kansas. Kansas Geological Survey Bulletin 260, 68 pp.	-	-	Approximated from Fig. 3 of Whittlemore et al. (2014).

Aquifer	Reference 1	Reference 2	Reference 3	Steps taken to delineate
	Accessed April 15, 2021 from http://www.kgs.ku.edu/Publications/Bulletins/260/Bulletin_260_Dakota.pdf			
Hamlin Valley	Hurlow, H. A. (2014). Hydrogeologic studies and groundwater monitoring in Snake Valley and adjacent hydrographic areas, west-central Utah and east-central Nevada (Vol. 135). Utah Geological Survey Bulletin 135, 272 pp.	-	-	Approximated from map on page 6 of Hurlow (2014)
Hanna and Carbon Basins	Daddow, P.D. (1980). Ground-water data for the Hanna and Carbon basins, south-central Wyoming, through 1980. U.S. Geological Survey Open-File Report 85-628, 94 pp. Accessed March 29, 2021 from https://pubs.usgs.gov/of/1985/0628/report.pdf	-	-	Approximated from Fig. 3 of Daddow (1980).
Harney Basin	Albano, C., M., Minor, B., Freed, Z., Huntington, J. L. (2020). Status and Trends of Groundwater Dependent Vegetation in Relation to Climate and Shallow Groundwater in the Harney Basin, Oregon. Report for The Nature Conservancy in Oregon (Contract ORFO-090418-aa01), 63 pp.	Ginerich, S., Johnson, H., Gannett, M., Burns, E. (2016). A Proposed Plan of Study for Investigation of the Groundwater System of the Harney Basin, Oregon. Plan of Study for a Cooperative Water-Resources Investigation. U.S. Geological Suvey Report. 23 pp. Accessed February 24, 2021 from https://www.oregon.gov/OWRD/programs/GWWL/GW/HarneyBasinStudy/Documents/USGS_OWRD_harney_groundwater_planofstudy_Dec2016.pdf	-	Approximated from Fig. 1 of Albano et al. (2020), and Fig. 1 by Ginerich et al. (2016), and influenced by the spatial distribution of groundwater wells recorded by the Oregon Water Resources Department (2021).
Harquahala Basin	Tillman, F.D, Cordova, J.T., Leake, S.A., Thomas, B.E., Callegary, J.B. (2011). Water availability and use pilot: methods development for a regional assessment of groundwater availability, southwest alluvial basins, Arizona. U.S. Geological Survey Scientific Investigations Report 2011-5071, 132 pp. Accessed April 15, 2021 from https://pubs.usgs.gov/sir/2011/5071/sir2011-5071_text.pdf	-	-	Approximated from Fig. 2 of Tillman et al. (2011); boundaries also informed by the density of wells recorded in Arizona's well completion dataset.
Heber Valley	Roark, D.M., Holmes, W.F., Shlosar, H.K. (1991). Hydrology of Heber and Round Valleys, Wasatch County, Utah, with emphasis on simulation of ground-water flow in Heber Valley. U.S. Geological Survey Technical Publication 101, 101 pp. Accessed March 27, 2021 from https://waterrights.utah.gov/docSys/v920/y920/y9200009.pdf	-	-	Approximated from Fig. 1 of Roark et al. (1991)
Helena Valley FIll	Briar, D.W., Madison, J.P. (1992). Hydrogeology of the Helena Valley-fill aquifer system, west-central Montana. U.S. Geological Survey Water-Resources Investigations Report 92-4023, 97 pp. Accessed March 29, 2021 from https://pubs.usgs.gov/wri/1992/4023/report.pdf	-	-	Approximated from Fig. 1 of Briar and Madison (1992)
Honey Lake Valley	Handman, E. H., Londquist, C. J., Maurer, D.K. (1990). Ground-water resources of Honey Lake valley, Lassen County, California, and Washoe County, Nevada. Water-Resources Investigations Report 90-4050, 119 pp. Accessed March 21, 2021 from https://pubs.usgs.gov/wri/1990/4050/report.pdf	-	-	Approximated from Fig. 1 of Handman et al. (1990)
Hualapai Valley Basin	Tillman, F.D, Garner, B.D., Truini, M. (2013). Preliminary groundwater flow model of the basin-fill aquifers in Detrital, Hualapai, and Sacramento Valleys, Mohave County, northwestern Arizona. U.S. Geological Survey Scientific Investigations Report 2013–5122, 52 pp. http://pubs.usgs.gov/sir/2013/5122/	-	-	Approximated from Fig. 2 by Tillman et al. (2013).
Hungry Valley	Kinder, J. (2012). Development of a Groundwater Flow Model for Hungry Valley, Washoe County, Nevada. University of Nevada, Reno MSc Thesis, 223 pp. Accessed March 22, 2021 from	-	-	Approximated from Fig. 6 of Kinder (2012)

Aquifer	Reference 1	Reference 2	Reference 3	Steps taken to delineate
	https://scholarworks.unr.edu/bitstream/handle/11714/3578/Kinder_unr_0139M_10957.pdf?sequence=1&isAllowed=y			
Indian Springs Valley	Burbey, T. J. (1997). Hydrogeology and potential for ground-water development, carbonate-rock aquifers in southern Nevada and southeastern California. US Geological Survey Water-Resources Investigations Report 95-4168, 70 pp. Accessed March 10, 2021 from https://pubs.usgs.gov/wri/1995/4168/report.pdf	-	-	Approximated from Fig. 13 of Burbey (1997)
Jackson Hole Aquifer	Wright, P.R. (2013). Hydrogeology and water quality in the Snake River alluvial aquifer at Jackson Hole Airport, Jackson, Wyoming, water years 2011 and 2012: U.S. Geological Survey Scientific Investigations Report 2013–5184, 56 pp. Accessed March 29, 2021 from https://pubs.usgs.gov/sir/2013/5184/pdf/sir2013-5184.pdf	-	-	Approximated from Fig. 3 of Wright (2013).
Juab Valley	Thiros, S. A., Stolp, B. J., Hadley, H. K., Steiger, J. I. (1996). Hydrology and simulation of ground-water flow in Juab Valley, Juab County, Utah. Utah Department of Natural Resources, Division of Water Rights Technical Report. 100 pp. Accessed March 7, 2021 from https://waterrights.utah.gov/docSys/v920/y920/y920000j.pdf	-	-	Approximated from Fig. 1 of Thiros et al. (1996)
Judith Basin	Levings, J. F. (1983). Hydrogeology and simulation of water flow in the Kootenai aquifer of the Judith basin, central Montana. U.S. Geological Survey Water-Resources Investigations Report 83-4146, 44 pp. Accessed March 29, 2021 from https://pubs.usgs.gov/wri/1983/4146/report.pdf	-	-	Approximated from Fig. 1 of Levings (1983).
Kettle River Valley	Walters, K. L. (1960). Availability of ground water at the border stations at Laurier and Ferry, Washington. US Geological Circular 422, 12 pp. Accessed March 15, 2021 from https://pubs.usgs.gov/circ/1960/0422/report.pdf	-	-	Approximated from Fig. 4 of Walters (1960); British Columbia portion estimated based on well completion data
Kings River Valley in northern Nevada	Bredehoeft, J.D., Farvolden, R.N. (1963). International Association of Scientific Hydrology, Commission of Subterranean Waters, Publication no. 64, p. 197–212. Accessed March 23, 2021 from http://hydrologie.org/redbooks/a064/064017.pdf	-	-	Approximated from Fig. 1 of Bredehoft and Farvolden (1963)
Kootenai Valley	Graham, W. G., Campbell, L. J. (1981). Groundwater resources of Idaho. Idaho Department of Water Resources Report, 61 pp. Accessed March 23, 2021 from https://idwr.idaho.gov/files/publications/198108-MISC-GW-Resources-ID.pdf	-	-	Approximated from Plate 1 of Graham and Campbell (1981). Margins extended into Montana and British Columbia, guided by topography and well completion report data.
Lahontan Valley	Smith, D.W., Buto, S.G., Welborn, T.L. (2016). Groundwater-level change and evaluation of simulated water levels for irrigated areas in Lahontan Valley, Churchill County, west-central Nevada, 1992–2012: U.S. Geological Survey Scientific Investigations Report 2016-5045, 23 pp. Accessed March 10, 2021 from https://pubs.usgs.gov/sir/2016/5045/sir20165045.pdf	-	-	Approximated from Fig. 4 of Smith et al. (2016)
Lake Mohave Basin	Tillman, F.D, Garner, B.D., Truini, M. (2013). Preliminary groundwater flow model of the basin-fill aquifers in Detrital, Hualapai, and Sacramento Valleys, Mohave County, northwestern Arizona. U.S. Geological Survey Scientific Investigations Report 2013–5122, 52 pp. http://pubs.usgs.gov/sir/2013/5122/	-	-	Approximated from Fig. 2 by Tillman et al. (2013).
Lake Valley	Hurlow, H. A. (2014). Hydrogeologic studies and groundwater monitoring in Snake Valley and adjacent hydrographic areas, west-central Utah and east-central Nevada (Vol. 135). Utah Geological Survey Bulletin 135, 272 pp.	-	-	Approximated from map on page 6 of Hurlow (2014)
Laramie Basin	Bradley, E. (1955). Summary of the ground-water resources of the Laramie River drainage basin, Wyoming, and the North Platte River drainage basin from Douglas, Wyoming, to the Wyoming-Nebraska state line. U.S. Geological Survey Open-File Report 55-17, 24 pp. Accessed March 29, 2021 from https://pubs.usgs.gov/of/1955/0017/report.pdf	-	-	Approximated from Fig. 1 of Bradley (1955)
Las Vegas Basin	Burbey, T. J. (1997). Hydrogeology and potential for ground-water development, carbonate-rock aquifers in southern Nevada and southeastern California. US Geological Survey Water-Resources Investigations Report 95-	-	-	Approximated from Fig. 10 of Burbey (1997)

Aquifer	Reference 1	Reference 2	Reference 3	Steps taken to delineate
	4168, 70 pp. Accessed March 10, 2021 from https://pubs.usgs.gov/wri/1995/4168/report.pdf			
Lemhi Valley	Graham, W. G., Campbell, L. J. (1981). Groundwater resources of Idaho. Idaho Department of Water Resources Report, 61 pp. Accessed March 23, 2021 from https://idwr.idaho.gov/files/publications/198108-MISC-GW-Resources-ID.pdf	-	-	Approximated from Plate 1 of Graham and Campbell (1981).
Little Chino Valley	Kennedy, J.R., Kahler, L.M., Read, A.L. (2019). Aquifer storage change and storage properties, 2010–2017, in the Big Chino Subbasin, Yavapai County, Arizona: U.S. Geological Survey Scientific Investigations Report 2019–5060, 39 pp. Accessed November 29, 2021 from https://pubs.usgs.gov/sir/2019/5060/sir20195060.pdf	-	-	Approximated from Fig. 1 of Kennedy et al. (2019)
Lonesome and Prescott Valleys	Matlock, W. G., Davis, P. R., Roth, R. L. (1973). Groundwater in Little Chino Valley, Arizona: Tucson, University of Arizona, College of Agriculture, Agricultural Experiment Station. Technical Bulletin, 201, 19. Accessed March 12, 2021 from https://repository.arizona.edu/bitstream/handle/10150/602177/TB178.pdf?sequence=1	-	-	Approximated from Plate 1 of Matlock et al. (1973)
Long Island	Smolensky, D.A., Buxton, H.T., Shernoff, P.K. (1990). Hydrologic framework of Long Island, New York. U.S. Geological Survey Hydrologic Atlas 709, 3 plates, Accessed April 1, 2021 from https://pubs.usgs.gov/ha/709/plate-1.pdf	Olcott, P.G. (1995). Ground Water Atlas of the United States: Segment 12, Connecticut, Maine, Massachusetts, New Hampshire, New York, Rhode Island, Vermont. U.S. Geological Survey Hydrologic Atlas 730-M, 30 pp. Accessed April 14, 2021 from https://pubs.usgs.gov/ha/730m/report.pdf	Masterson, J. P., Pope, J. P., Fienen, M. N., Monti Jr, J., Nardi, M. R., Finkelstein, J. S. (2016). Assessment of groundwater availability in the Northern Atlantic Coastal Plain aquifer system from Long Island, New York, to North Carolina (No. 1829). US Geological Survey Professional Paper 1829, 90 pp. Accessed November 29, 2021 from https://pubs.usgs.gov/pp/1829/pp1829.pdf	Approximated from Plate 1 of Smolensky et al. (1990), Fig. 6 by Masterson et al. (2016), and Fig. 62 of Olcott (1995).
Long Valley - Round Valley	Graham, W. G., Campbell, L. J. (1981). Groundwater resources of Idaho. Idaho Department of Water Resources Report, 61 pp. Accessed March 23, 2021 from https://idwr.idaho.gov/files/publications/198108-MISC-GW-Resources-ID.pdf	-	-	Approximated from Plate 1 of Graham and Campbell (1981).
Lordsburg Valley	Schwennesen, A.T., Hare, R.F. (1918). Ground water in the Animas, Playas, Hachita, and San Luis Basins, New Mexico, with analyses of water and soil. U.S. Geological Survey Water Supply Paper 422, 157 pp. Accessed March 29, 2021 from https://pubs.usgs.gov/wsp/0422/report.pdf	-	-	Approximated from Plate II of Schwennesen and Hare (1918).
Los Angeles Basin	Land, M., Reichard, E.G., Crawford, S.M., Everett, R.R., Newhouse, M.W., Williams, C.F. (2004). Ground-water Quality of Coastal Aquifer Systems in the West Coast Basin, Los Angeles County, California, 1999–2002. U.S. Geological Survey Scientific Investigations Report 2004-5067, 88 pp. Accessed February 15, 2021 from https://pubs.usgs.gov/sir/2004/5067/sir2004-5067.pdf	Fram, M. S., Belitz, K. (2008). Groundwater quality in the Coastal Los Angeles Basin, California; United States Geological Survey Fact Sheet 2012-3096. Accessed February 14, 2021 from https://pubs.er.usgs.gov/publication/70039952.	Reichard, E. G., Land, M., Crawford, S.M., Johnson, T.D., Everett, R.R., Kulshan, T.V., Ponti, D.J., Halford, K.L., Johnson, T.A., Paybins, K.S., Nishikawa, T. (2003). Geohydrology, Geochemistry, and Ground-Water Simulation Optimization of the Central and West Coast Basins, Los Angeles County, California.	Approximated from Fig. 1 by Land et al. (2004), Fig. 6 by Reichard et al. (2003) and map on page 1 of Fram and Belitz (2008)

Aquifer	Reference 1	Reference 2	Reference 3	Steps taken to delineate
			USGS Numbered Series, 2003–4065. Accessed November 28, 2021 from https://pubs.usgs.gov/wri/wrir034065/wrir034065.pdf	
Lower Arkansas River Eastern Reach	Holmberg, M.J. (2017). Hydrogeologic characteristics and geospatial analysis of water-table changes in the alluvium of the lower Arkansas River Valley, southeastern Colorado, 2002, 2008, and 2015: U.S. Geological Survey Scientific Investigations Map 3378, 20 pp. Accessed March 29, 2021 from https://pubs.usgs.gov/sim/3378/sim3378.pdf	-	-	Approximated from Fig. 1 of Holmgren (2017); eastern and western reaches separated by John Martin Reservoir.
Lower Arkansas River Western Reach	Holmberg, M.J. (2017). Hydrogeologic characteristics and geospatial analysis of water-table changes in the alluvium of the lower Arkansas River Valley, southeastern Colorado, 2002, 2008, and 2015: U.S. Geological Survey Scientific Investigations Map 3378, 20 pp. Accessed March 29, 2021 from https://pubs.usgs.gov/sim/3378/sim3378.pdf	-	-	Approximated from Fig. 1 of Holmgren (2017); eastern and western reaches separated by John Martin Reservoir.
Lower Gila Basin	Tillman, F.D, Cordova, J.T., Leake, S.A., Thomas, B.E., Callegary, J.B. (2011). Water availability and use pilot: methods development for a regional assessment of groundwater availability, southwest alluvial basins, Arizona. U.S. Geological Survey Scientific Investigations Report 2011-5071, 132 pp. Accessed April 15, 2021 from https://pubs.usgs.gov/sir/2011/5071/sir2011-5071_text.pdf	-	-	Approximated from Fig. 2 of Tillman et al. (2011); boundaries also informed by the density of wells recorded in Arizona's well completion dataset.
Lower Reese River Valley and Antelope Valley	Bredehoeft, J.D., Farvolden, R.N. (1963). International Association of Scientific Hydrology, Commission of Subterranean Waters, Publication no. 64, p. 197–212. Accessed March 23, 2021 from http://hydrologie.org/redbooks/a064/064017.pdf	-	-	Approximated from Fig. 1 of Bredehoft and Farvolden (1963) and guided by locations of wells and topography (especially to distinguish upper and lower portions of basin).
Lower Ruby River Valley	Northern Rockies Engineering Inc. (2021). Plate B1. Shallow Aquifer Classification of theLower Ruby Valley, Montana. Accessed March 29, 2021 from https://www.northernrockiesengineering.com/wp-content/uploads/2020/03/PlatesB1_B2_Ruby_classification.pdf	-	-	Approximated from plates by Northern Rockies Engineering Inc. (2021). Note: publication date was not provided; instead, the year the report was accessed via the referenced website is presented in the reference.
Lower Walker Basin	Allander, K. K., Niswonger, R. G., Jeton, A. E. (2014). Simulation of the Lower Walker River Basin hydrologic system, west central Nevada, using PRMS and MODFLOW models. U.S. Geological Survey Scientific Investigations Report 2014–5190, 108 pp. Accessed March 21, 2021 from https://pubs.usgs.gov/sir/2014/5190/pdf/sir2014-5190.pdf	-	-	Approximated from Fig. 1 of Allander et al. (2014) with guidance from completed well locations
Lyman Mountain View Area	Robinove, C.J., Cummings, T.R. (1963). Ground-water resources and geology of the Lyman-Mountain View area, Uinta County, Wyoming. U.S. Geological Survey Water Supply Paper 1669, 48 pp. Accessed March 29, 2021 from https://pubs.usgs.gov/wsp/1669e/report.pdf	-	-	Approximated from plate 1 of Robinove and Cummings (1963)
Madison Aquifer	LaFave, J. (2011). Quality and Age of Water in the Madison Aquifer, Cascade County, Montana. Montana American Water Resources Association Conference, Session 2. 27 pp. Accessed March 29, 2021 from https://www.montanaawra.org/wp/ppts/2011/session2/5_LaFave_John_i.pdf	Downey, J. S. (1982). Geohydrology of the Madison and associated aquifers in parts of Montana, North Dakota, South Dakota, and Wyoming. U.S. Geological Survey Professional Paper 1273-G, 54 pp. Accessed March 29, 2021 from https://pubs.usgs.gov/pp/1273g/report.pdf	-	While the Madison Aquifer is extensive (spanning multiple states; see Downey (1982)), the relatively small region in central Montana is delineated here as it is "...(m)ost utilized in Cascade Co." (quote LaFave (2011)).

Aquifer	Reference 1	Reference 2	Reference 3	Steps taken to delineate
Malad Valley	Graham, W. G., Campbell, L. J. (1981). Groundwater resources of Idaho. Idaho Department of Water Resources Report, 61 pp. Accessed March 23, 2021 from https://idwr.idaho.gov/files/publications/198108-MISC-GW-Resources-ID.pdf	-	-	Approximated from Plate 1 of Graham and Campbell (1981).
Mammoth Basin	Flora, S., Davis, T. (2009). Hydrologic Map Series (HMS), Water Level Change Map Series (WLCMS), and Basin Sweep Assessment Report ADWR Basins and Sub-Basins. Arizona Department of Water Resources Hydrology Division Field Services Section https://new.azwater.gov/sites/default/files/HMSWLCMSBasinSweepAssessmentReport2009.pdf	-	-	Approximated from Fig. 1 of Flora and Davis (2009)
Maricopa-Stanfield Basin	Flora, S., Davis, T. (2009). Hydrologic Map Series (HMS), Water Level Change Map Series (WLCMS), and Basin Sweep Assessment Report ADWR Basins and Sub-Basins. Arizona Department of Water Resources Hydrology Division Field Services Section https://new.azwater.gov/sites/default/files/HMSWLCMSBasinSweepAssessmentReport2009.pdf	-	-	Approximated from Fig. 1 of Flora and Davis (2009). Hydrologic Map Series (HMS), Water Level Change Map Series (WLCMS), and Basin Sweep Assessment Report ADWR Basins and Sub-Basins. Arizona Department of Water Resources Hydrology Division Field Services Section https://new.azwater.gov/sites/default/files/HMSWLCMSBasinSweepAssessmentReport2009.pdf
Mason Valley	Carroll, R.W., Pohll, G., McGraw, D., Garner, C., Knust, A., Boyle, D., Minor, T., Bassett, S. Pohlmann, K. (2010). Mason Valley Groundwater Model: Linking Surface Water and Groundwater in the Walker River Basin, Nevada 1. JAWRA Journal of the American Water Resources Association, 46(3), 554-573.	-	-	Approximated from Fig. 2 of Carroll et al. (2010); southern margins extended to include recorded drilled wells.
McMullen Valley	Kam, W. (1964). Geology and Ground-Water Resources of McMullen Valley Maricopa, Yavapai, and Yuma Counties, Arizona. U.S. Geological Survey Water-Supply Paper 1665, 70 pp. Accessed April 15, 2021 from https://pubs.usgs.gov/wsp/1665/report.pdf	-	-	Approximated from Fig. 2 of Kam (1964)
Mesquite Valley	Burbey, T. J. (1997). Hydrogeology and potential for ground-water development, carbonate-rock aquifers in southern Nevada and southeastern California. US Geological Survey Water-Resources Investigations Report 95-4168, 70 pp. Accessed March 10, 2021 from https://pubs.usgs.gov/wri/1995/4168/report.pdf	-	-	Approximated from Fig. 15 of Burbey (1997)
Michigan Basin	Olcott, P.G. (1992). Groundwater Atlas of the United States: Segment 9 Iowa, Michigan, Minnesota, Wisconsin. U.S. Geological Survey Hydrologic Atlas 730-J, 33 pp. Accessed April 12, 2021 from https://pubs.usgs.gov/ha/730j/report.pdf	Westjohn, D.B., Weaver, T.L. (1998). Hydrogeologic framework of the Michigan Basin regional aquifer system. US Geological Survey Professional Paper 1418, 55 pp. Accessed November 29, 2021 from https://pubs.usgs.gov/pp/1418/report.pdf	-	Approximated from Figs. 50-64 by Olcott (1992) and Fig. 1 by Westjohn and Weaver (1998)
Middle Yellowstone Area	Madison, J.P., LaFave, J.I., Patton, T.W., Smith, L.N., Olson, J.N. (2014). Groundwater resources of the Middle Yellowstone River area: Treasure and Yellowstone counties, Montana Part A* - Descriptive Overview and Water-Quality Data: Montana Bureau of Mines and Geology Montana Ground-Water Assessment Atlas 3-A, 82 pp. Accessed March 29, 2021 from http://mbmg.mtech.edu/pdf-publications/gwaa_3.pdf	-	-	Approximated from Fig. 10 of Madison et al. (2014).
Milk River	Pétré, M. A., Rivera, A., Lefebvre, R., Hendry, M. J., Folnagy, A. J. (2016). A unified hydrogeological conceptual model of the Milk River transboundary	Phillips, F. M., Bentley, H. W., Davis, S. N., Elmore, D., Swanick, G. B. (1986). Chlorine 36 dating	-	Approximated from Fig. 2 of Pétré et al. (2016) and Figs. 1 and 2 by Phillips et al. (1986)

Aquifer	Reference 1	Reference 2	Reference 3	Steps taken to delineate
	aquifer, traversing Alberta (Canada) and Montana (USA). Hydrogeology Journal, 24, 1847-1871.	of very old groundwater: 2. Milk River aquifer, Alberta, Canada. Water Resources Research, 22, 2003-2016.		
Mill Creek Aquifer	Graham, W. G., Campbell, L. J. (1981). Groundwater resources of Idaho. Idaho Department of Water Resources Report, 61 pp. Accessed March 23, 2021 from https://idwr.idaho.gov/files/publications/198108-MISC-GW-Resources-ID.pdf	-	-	Approximated from Plate 1 of Graham and Campbell (1981)
Mimbres Basin	Hanson, R.T., McLean, J.S., Miller, R.S. (1994). Hydrogeologic framework and preliminary simulation of ground-water flow in the Mimbres Basin, Southwestern New Mexico: U.S. Geological Survey, Water-Resources Investigations Report 94-4011, 118 pp. Accessed March 27, 2021 from https://pubs.usgs.gov/wri/1994/4011/report.pdf	Finch, S. T., Mccoy, A., Melis, E. (2008). Geologic Controls on Groundwater Flow in the Mimbres Basin, South Western New Mexico. In New Mexico Geological Society Guide Book, 59th Field Conference, pp. 189-198. Accessed March 27, 2021 from https://nmgs.nmt.edu/publications/guidebooks/downloads/59/59_p0189_p0198.pdf	White, W. N. (1931). Preliminary report on the ground-water supply of Mimbres Valley, New Mexico. US Geological Survey Water Supply Paper 637, 23 pp. Accessed November 7, 2021 from https://pubs.usgs.gov/wsp/0637B/report.pdf	Approximated from Fig. 1 of Hansen et al. (1994), Fig. 1 of Finch et al. (2008) and Plate 1 by White (1931)
Mojave Basin	Stamos, C. L., Christensen, A. H., Langenheim, V. (2017). Preliminary hydrogeologic assessment near the boundary of the Antelope Valley and El Mirage Valley groundwater basins, California. U.S. Geological Survey Scientific Investigations Report 2017–5065, 56 pp. Accessed March 20, 2021 from https://pubs.usgs.gov/sir/2017/5065/sir20175065.pdf	-	-	Approximated from Fig. 7 of Stamos et al. (2017)
Montecito Basin	Muir, K.S. (1968). Ground-water reconnaissance of the Santa Barbara-Montecito area, Santa Barbara County, California: U.S. Geological Survey Water Supply Paper 1859–A, 28 pp. Accessed March 20, 2021 from https://pubs.usgs.gov/wsp/1859a/report.pdf	-	-	Approximated from Plate 1 of Muir (1968)
Muscatatuck Plateau and Dearborn Upland	Gray, H.H. (2001). Map of Indiana showing physiographic divisions. Indiana Geological Survey Miscellaneous Map 69, 15 pp. Accessed April 12, 2021 from https://igws.indiana.edu/ReferenceDocs/Maps/PhysiographicRegions.pdf	Schrader, G.P. (2004). Unconsolidated Aquifer Systems of Ripley County, Indiana. Indiana Department of Natural Resources, Division of Water Report, 3 pp. Accessed April 12, 2021 from https://www.in.gov/dnr/water/files/ripley_unconsolidated_text.pdf	-	Approximated from map by Gray (2001). Muscatatuck Plateau and Dearborn Upland combined as local hydrogeologic report refers to these two regions as a single unit (e.g., quote Schrader (2004): "Muscatatuck Plateau / Dearborn Upland Till Aquifer Subsystem."
Napa Valley	Kunkle, F., Upson, J. E. (1960). Geology and ground water in Napa and Sonoma Valleys, Napa and Sonoma Counties, California. U.S. Geological Survey Water-Supply Paper 1495, 260 pp. Accessed March 27, 2021 from https://pubs.usgs.gov/wsp/1495/report.pdf	-	-	Approximated from Plate 4 of Kunkle and Upson (1960).
New Hampshire Seacoast	Mack, T.J. (2008). Assessment of Ground-Water Resources in the Seacoast Region of New Hampshire. Scientific Investigations Report 2008–5222, 66 pp. Accessed April 15, 2021 from https://pubs.usgs.gov/sir/2008/5222/pdf/sir2008-5222.pdf	-	-	Approximated from Fig. 7 of Mack (2008)
North Park Basin	Robson, S.G., Graham, G. (1996). Geohydrology of the North Park area, Jackson County, Colorado; with a section on water law. U.S. Geological Survey Water-Resources Investigations Report 96-4166, 1 plate. Accessed March 29, 2021 from https://pubs.usgs.gov/wri/1996/4166/plate-1.pdf	-	-	Approximated from plate 1 by Robson and Graham (1996).

Aquifer	Reference 1	Reference 2	Reference 3	Steps taken to delineate
Northern Green River Basin	Bartos, T.T., Hallberg, L.L., Eddy-Miller, C.A. (2015). Hydrogeology, groundwater levels, and generalized potentiometric-surface map of theGreen River Basin lower Tertiary aquifer system, 2010–14, in the northern Green River structural basin, Wyoming: U.S. Geological Survey Scientific Investigations Report 2015–5090, 33 pp. Accessed March 29, 2021 from https://pubs.usgs.gov/sir/2015/5090/sir20155090.pdf	-	-	Approximated from Fig. 1 of Bartos et al. (2015)
Northern San Juan Basin	Kernodle, J. M. (1996). Hydrogeology and steady-state simulation of ground-water flow in the San Juan Basin, New Mexico, Colorado, Arizona, and Utah. US Geological Survey Water-Resources Investigations Report 95-4187, 126 pp. Accessed March 12, 2021 from https://pubs.usgs.gov/wri/1995/4187/report.pdf	Welder, G. E. (1986). Plan of study for the regional aquifer system analysis of the San Juan structural basin, New Mexico, Colorado, Arizona, and Utah. U.S. Geological Survey Water Resources Investigations Report 85-4294, 27 pp. Accessed April 14, 2021 from https://pubs.usgs.gov/wri/1985/4294/report.pdf	-	Approximated from Fig. 3 of Kernodle (1996) and Fig. 4 of Welder (1986). Southern portion approximated on the basis of groundwater well density (derived from New Mexico's well completion dataset).
Nussbaum Aquifer	Mustard, M.H., Cain, D. (1981). Hydrology and chemical quality of ground water in Kiowa County, Colorado. U.S. Geological Survey Open-File Report 81-1023, 2 plates, Accessed March 29, 2021 from https://pubs.er.usgs.gov/publication/ofr811023	-	-	Approximated from Plate 1 by Mustard and Cain (1981)
Ogden Valley	Jordan, J. L., Smith, S.D., Inkenbrandt, P.C., Lowe, M., Hardwick, C.L., Wallace, J., Kirby, S.M., King, J.K., Payne, E.E. (2019). Characterization of the groundwater system in Ogden Valley, Weber County, Utah, with emphasis on groundwater–surface-water interaction and the groundwater budget. Utah Geological Survey Report, Special Study 165. 237 pp. Accessed March 27, 2021 from https://ugspub.nr.utah.gov/publications/special_studies/ss-165/ss-165.pdf	-	-	Approximated from Fig. 1 of Dustin (1978)
Ozark Plateaus Aquifer System	Clark, B. R., Duncan, L. L., Knierim, K. J. (2019). Groundwater availability in the Ozark Plateaus aquifer system. US Geological Survey Professional Paper 1854, 95 pp. Accessed February 13, 2021 from https://pubs.er.usgs.gov/publication/pp1854	Imes, J. L., Emmett, L. F. (1994). Geohydrology of the Ozark Plateaus aquifer system in parts of Missouri, Arkansas, Oklahoma, and Kansas (No. 1414-D). Accessed February 13, 2021 from https://pubs.usgs.gov/pp/1414d/report.pdf	Hays, P.D., Knierim, K.J., Breaker, Brian, Westerman, D.A., and Clark, B.R., 2016, Hydrogeology and hydrologic conditions of the Ozark Plateaus aquifer system: U.S. Geological Survey Scientific Investigations Report 2016–5137, 61 p., http://dx.doi.org/10.3133/sir20165137.	Approximated from Fig. 1 of Hays et al. (2016), Fig. 1 by Clark et al. (2019), and Fig. 2 by Imes and Emmett (1994)
Pahranagat Valley	Burbey, T. J. (1997). Hydrogeology and potential for ground-water development, carbonate-rock aquifers in southern Nevada and southeastern California. US Geological Survey Water-Resources Investigations Report 95-4168, 70 pp. Accessed March 10, 2021 from https://pubs.usgs.gov/wri/1995/4168/report.pdf	-	-	Approximated from Fig. 4 of Burbey (1997)
Pahrump Valley	Burbey, T. J. (1997). Hydrogeology and potential for ground-water development, carbonate-rock aquifers in southern Nevada and southeastern California. US Geological Survey Water-Resources Investigations Report 95-4168, 70 pp. Accessed March 10, 2021 from https://pubs.usgs.gov/wri/1995/4168/report.pdf	-	-	Approximated from Fig. 15 of Burbey (1997)
Pahsimeroi Valley	Graham, W. G., Campbell, L. J. (1981). Groundwater resources of Idaho. Idaho Department of Water Resources Report, 61 pp. Accessed March 23, 2021	-	-	Approximated from Plate 1 of Graham and Campbell (1981).

Aquifer	Reference 1	Reference 2	Reference 3	Steps taken to delineate
	from https://idwr.idaho.gov/files/publications/198108-MISC-GW-Resources-ID.pdf			
Pahvant Valley	Holmes, W. F., Thiros, S. A. (1990). Ground-water hydrology of Pahvant Valley and adjacent areas, Utah. United State Geological Survey Technical Publication 98, 64 pp. Accessed March 7, 2021 from https://waterrights.utah.gov/docSys/v920/y920/y9200006.pdf	-	-	Approximated from Fig. 8 of Brooks et al. (2005)
Paradise Valley in northern Nevada	Bredehoeft, J.D., Farvolden, R.N. (1963). International Association of Scientific Hydrology, Commission of Subterranean Waters, Publication no. 64, p. 197–212. Accessed March 23, 2021 from http://hydrologie.org/redbooks/a064/064017.pdf	-	-	Approximated from Fig. 1 of Bredehoft and Farvolden (1963)
Parowan Valley	Marston, T.M. (2017). Water resources of Parowan Valley, Iron County, Utah: U.S. Geological Survey Scientific Investigations Report 2017–5033, 45 pp. Accessed March 7 2021 from https://doi.org/10.3133/sir20175033.	-	-	Approximated from Fig. 1 of Marston et al. (2017)
Payette Valley	Graham, W. G., Campbell, L. J. (1981). Groundwater resources of Idaho. Idaho Department of Water Resources Report, 61 pp. Accessed March 23, 2021 from https://idwr.idaho.gov/files/publications/198108-MISC-GW-Resources-ID.pdf	-	-	Approximated from Plate 1 of Graham and Campbell (1981). Oregon portion based on well completion data.
Pearl and Chattahoochee Aquifer System	Miller, J.A. (1990). Ground Water Atlas of the United States: Segment 6, Alabama, Florida, Georgia, South Carolina. U.S. Geological Survey Hydrologic Atlas 730-G, 30 pp. Accessed April 5, 2021 from https://www.nrc.gov/docs/ML1706/ML17060B027.pdf	-	-	Approximated from Fig. 73 of Miller (1990)
Pecos Valley	Bruun, B., Jackson, K., Lake, P., Walker, J. (2016). Texas Aquifers Study. Texas Water Development Board Report. 336 pp. Accessed April 1, 2021 from https://www.twdb.texas.gov/groundwater/docs/studies/TexasAquifersStudy_2016.pdf#page=89	-	-	Approximated from Fig. 6-28 by Bruun et al. (2016). New Mexico portion of aquifer approximated on the basis of well completion records.
Peedee and Black Creek and Cape Fear Aquifers	Winner Jr., M.D., Coble, R.W. (1989). Hydrogeologic framework of the North Carolina Coastal Plain aquifer system. U.S. Geological Survey Report 87-690, 167 pp. Accessed April 1, 2021 from https://pubs.usgs.gov/of/1987/0690/report.pdf	Harden, S. L., Fine, J. M., Spruill, T. B. (2003). Hydrogeology and ground-water quality of Brunswick County, North Carolina. U.S. Geological Survey, Water-Resources Investigations Report 03–4051, 98 pp. Accessed April 1, 2021 from https://pubs.usgs.gov/wri/2003/4051/wri20034051.pdf	-	Approximated from Fig. 2 of Winner Jr. and Coble (1989) and Plate 9 of Harden et al. (2003).
Pend Orielle East	Graham, W. G., Campbell, L. J. (1981). Groundwater resources of Idaho. Idaho Department of Water Resources Report, 61 pp. Accessed March 23, 2021 from https://idwr.idaho.gov/files/publications/198108-MISC-GW-Resources-ID.pdf	-	-	Approximated from Plate 1 of Graham and Campbell (1981). Eastern portion of Pend Orielle River aquifer was delineated separately from the western portion.
Pend Orielle West	Graham, W. G., Campbell, L. J. (1981). Groundwater resources of Idaho. Idaho Department of Water Resources Report, 61 pp. Accessed March 23, 2021 from https://idwr.idaho.gov/files/publications/198108-MISC-GW-Resources-ID.pdf	-	-	Approximated from Plate 1 of Graham and Campbell (1981). Western portion of Pend Orielle River aquifer was delineated separately from the eastern portion.
Picacho Basin	Pool., D.R., Carruth, R., Meehan, W.D. (2001). Hydrogeology of Picacho Basin, south-central Arizona. Water-Resources Investigations Report 2000-4277, 72 pp. Accessed April 15, 2021 from https://pubs.usgs.gov/wri/2000/4277/report.pdf	-	-	Approximated from Fig. 2 of Pool et al. (2001).
Playas Valley	Schwennesen, A.T., Hare, R.F. (1918). Ground water in the Animas, Playas, Hachita, and San Luis Basins, New Mexico, with analyses of water and soil. U.S. Geological Survey Water Supply Paper 422, 157 pp. Accessed March 29, 2021 from https://pubs.usgs.gov/wsp/0422/report.pdf	-	-	Approximated from Plate II of Schwennesen and Hare (1918).

Aquifer	Reference 1	Reference 2	Reference 3	Steps taken to delineate
Plymouth-Carver-Kingston-Duxburya Aquifer System	Masterson, J.P., Walter, D.A. (2009). Hydrogeology and groundwater resources of the coastal aquifers of southeastern Massachusetts. U.S. Geological Survey Circular 1338, 16 pp. Accessed April 14, 2021 from https://pubs.usgs.gov/circ/circ1338/pdf/circular%202009-1338_508.pdf	-	-	Approximated from Fig. 1 of Masterson and Walter (2009).
Powell Park	Van Liew, W.P., Gesink, M.L. (1985). Preliminary assessment of the ground-water resources of the alluvial aquifer, White River valley, Rio Blanco County, Colorado. U.S. Geological Survey Water-Resources Investigations Report 84-4307, 82 pp. Accessed March 29, 2021 from https://pubs.usgs.gov/wri/1984/4307/report.pdf	-	-	Approximated from plate 1 by Van Liew and Gesink (1985).
Priest River Basin	Graham, W. G., Campbell, L. J. (1981). Groundwater resources of Idaho. Idaho Department of Water Resources Report, 61 pp. Accessed March 23, 2021 from https://idwr.idaho.gov/files/publications/198108-MISC-GW-Resources-ID.pdf	-	-	Approximated from Plate 1 of Graham and Campbell (1981). Southern boundary of basin delineated based on topography.
Quinn River Valley in northern Nevada	Huntington, J.L., Minor, B., Bromley, M., Morton, C. (2018). Reconnaissance Investigation of Phreatophyte Vegetation Vigor for Selected Hydrographic Areas in Nevada. Division of Hydrologic Sciences, Desert Research Institute, Reno, NV Report, 75 pp. Accessed March 23, 2021 from http://www.conservationgateway.org/ConservationByGeography/NorthAmerica/UnitedStates/nevada/water/Documents/Final%20DRI-TNC%20spatiotemporal%20phreatophyte%20report_may31.pdf	-	-	Approximated from Fig. 6 of Huntington et al. (2018); boundary extended northward and delineated based on well completion reports.
Railroad Valley	Rose, T. P., Davisson, M. L., Smith, D. K., Kenneally, J. M. (1998). Isotope hydrology investigation of regional groundwater flow in central Nevada. Hydrologic Resources Management Program and Underground Test Area Operable Unit FY 1997 Progress Report. Chapter 6. Accessed March 10, 2021 from https://core.ac.uk/download/pdf/204554577.pdf#page=62	-	-	Approximated from Fig. 3 of Rose et al. (1989)
Ranegras Plain	Tillman, F.D, Cordova, J.T., Leake, S.A., Thomas, B.E., Callegary, J.B. (2011). Water availability and use pilot: methods development for a regional assessment of groundwater availability, southwest alluvial basins, Arizona. U.S. Geological Survey Scientific Investigations Report 2011-5071, 132 pp. Accessed April 15, 2021 from https://pubs.usgs.gov/sir/2011/5071/sir2011-5071_text.pdf	-	-	Approximated from Fig. 2 of Tillman et al. (2011); boundaries also informed by the density of wells recorded in Arizona's well completion dataset.
Red River Aquifer	Smith, S.J., Ellis, J.H., Paizis, N.C., Becker, C.J., Wagner, D.L., Correll, J.S., Hernandez, R.J. (2021). Hydrogeology and model-simulated groundwater availability in the Salt Fork Red River aquifer, southwestern Oklahoma, 1980–2015. U.S. Geological Survey Scientific Investigations Report 2021–5003, 85 pp. Accessed April 5, 2021 from https://pubs.usgs.gov/sir/2021/5003/sir20215003.pdf	Smith, S.J., Ellis, J.H., Wagner, D.L., Peterson, S.M. (2017). Hydrogeology and simulated groundwater flow and availability in the North Fork Red River aquifer, southwest Oklahoma, 1980–2013: U.S. Geological Survey Scientific Investigations Report 2017–5098, 107 pp. Accessed April 5, 2021 from https://pubs.usgs.gov/sir/2017/5098/sir20175098.pdf	-	Approximated from Fig. 1 of Smith et al. (2017) and Fig. 1 of Smith et al. (2021).
Redlands Mesa	Kolm, K.E., van der Heijde, P.K.M. (2014). Groundwater Systems In Delta County, Colorado: Surface Creek Valley Area. Report prepared for Delta County Board of County Commissioners, Colorado, 64 pp. Accessed March 28, 2021 from https://www.chc4you.org/wp-content/uploads/2017/01/Surface-Creek-Hydrology-Report-2014.pdf	-	-	Approximated from Fig. 23b of Koh and van der Heijde (2014)
Rogers Mesa	Watts, K.R. (2008) Availability, sustainability, and suitability of ground water, Rogers Mesa, Delta County, Colorado—types of analyses and data for use in subdivision water-supply reports: U.S. Geological Survey Scientific	-	-	Approximated from Fig. 1 of Watts (2008)

Aquifer	Reference 1	Reference 2	Reference 3	Steps taken to delineate
	Investigations Report 2008–5020, 53 pp. Accessed March 28, 2021 from https://pubs.usgs.gov/sir/2008/5020/pdf/SIR2008-5020.pdf			
Rogue Basin	Robison, J. H. (1971). Availability and quality of ground water in the Medford area, Jackson County, Oregon. U.S. Geological Survey Hydrologic Atlas 392, 2 plates. Accessed March 16, 2021 from https://pubs.er.usgs.gov/publication/ha392	State of Oregon Department of Environmental Quality (2015). Statewide Groundwater Monitoring Program: Mid-Rogue Basin 2015. 30 pp. Accessed March 16, 2021 from https://www.oregon.gov/deq/FilterDocs/gw-DEQ16-LAB-0042-TR.pdf	-	Approximated from Fig. 2 of State of Oregon Department of Environmental Quality (2015) and plate 1 of Robison (1971)
Roswell Basin	Land, L. (2016). Overview of Fresh and Brackish Water Quality in New Mexico. Open file Report 583. 4 pp. Accessed February 17, 2021 from https://geoinfo.nmt.edu/resources/water/amp/brochures/BWA/Estancia_Basin_FBWQNM.pdf	-	-	Approximated from Map by Land (2016)
Round Valley	Graham, W. G., Campbell, L. J. (1981). Groundwater resources of Idaho. Idaho Department of Water Resources Report, 61 pp. Accessed March 23, 2021 from https://idwr.idaho.gov/files/publications/198108-MISC-GW-Resources-ID.pdf	-	-	Approximated from Plate 1 of Graham and Campbell (1981)
Ruby Valley	Berger, D. L. (2006). Hydrogeology and Water Resources of Ruby Valley, Northeastern Nevada. Scientific Investigations Report 2005-5247, 48 pp. Accessed March 23, 2021 from https://pubs.usgs.gov/sir/2005/5247/sir2005-5247.pdf	-	-	Approximated from Fig. 3 of Berger (2006)
Rush Springs Aquifer	Becker, M.F., Runkle, D.L. (1998). Hydrogeology, water quality, and geochemistry of the Rush Springs aquifer, western Oklahoma. U.S. Geological Survey Water-Resources Investigations Report 98-4081, 44 pp. Accessed April 5, 2021 from https://pubs.usgs.gov/wri/1998/4081/report.pdf	-	-	Approximated from Fig. 1 of Becker and Runkle (1998)
Rush Valley	Gardner, P. M., Kirby, S. (2011). Hydrogeologic and geochemical characterization of groundwater resources in Rush Valley, Tooele County, Utah. U. S. Geological Survey Scientific Investigations Report 2011–5068, 80 pp. Accessed March 7, 2021 from https://pubs.usgs.gov/sir/2011/5068/pdf/sir20115068.pdf	-	-	Approximated from Fig. 1 of Gardner and Kirby (2011)
Sacramento Valley Basin	Tillman, F.D, Garner, B.D., Truini, M. (2013). Preliminary groundwater flow model of the basin-fill aquifers in Detrital, Hualapai, and Sacramento Valleys, Mohave County, northwestern Arizona. U.S. Geological Survey Scientific Investigations Report 2013–5122, 52 pp. http://pubs.usgs.gov/sir/2013/5122/	-	-	Approximated from Fig. 2 by Tillman et al. (2013)
Salinas Valley	Salinas Valley Basin Integrated Sustainability Plan (2020). Accessed February 15, 2021 from https://svbgsa.org/wp-content/uploads/2019/03/Valley-Wide-Integrated-Sustainability-Plan-optimized.pdf	Hamlin, H. (1904). Water resources of the Salinas Valley, California. U.S. Geological Survey Water Supply Paper 89, 123 pp. Accessed April 6, 2021 from https://pubs.usgs.gov/wsp/0089/report.pdf	-	Approximated from Fig. 5-2 of Salinas Valley Basin Integrated Sustainability Plan (2020) and Plate 1 of Hamlin (1904).
Salt Lake Valley	Thiros, S.A. (2003). Hydrogeology of shallow basin-fill deposits in areas of Salt Lake Valley, Salt Lake County, Utah. U.S. Geological Survey Water-Resources Investigations Report 2003-4029, 32 pp. Accessed March 27, 2021 from https://pubs.usgs.gov/wri/wri034029/pdf/wri034029.pdf	-	-	Approximated from Fig. 1 of Thiros (2003)
San Antonio Creek Valley	Martin, P. (1984). Development and calibration of a two-dimensional digital model for the analysis of the ground-water flow system in the San Antonio Creek Valley, Santa Barbara County, California. Water-Resources	-	-	Approximated from maps on pages 8 and 9 of Martin (1984)

Aquifer	Reference 1	Reference 2	Reference 3	Steps taken to delineate
	Investigations Report 84-4340, 73 pp. Accessed March 7, 2021 from https://pubs.usgs.gov/wri/1984/4340/report.pdf			
San Jacinto Basin	Kent, R., Belitz, K. (2009). Ground-water quality data in the Upper Santa Ana Watershed Study Unit, November 2006 to March 2007: Results from the California GAMA Program: U.S. Geological Survey Data Series 404, 116 pp. Accessed March 21, 2021 from https://pubs.usgs.gov/ds/404/ds404.pdf	-	-	Approximated from Fig. 2 of Kent and Belitz (2009)
San Pedro Basin	Callegary, J.B., Minjárez Sosa, I., Tapia Villaseñor, E.M., dos Santos, P., Monreal Saavedra, R., Grijalva Noriega, F.J., Huth, A.K., Gray, F., Scott, C.A., Megdal, S.B., Oroz Ramos, L.A., Rangel Medina, M., Leenhouts, J.M. (2016). San Pedro River Aquifer Binational Report: International Boundary and Water Commission. 173 pp. Accessed February 12, 2021 via https://www.ibwc.gov/Files/Binational_Study_Transboundary_San_Pedro_Aquifer.pdf	Sanchez, R., Lopez, V., Eckstein, G. (2016). Identifying and characterizing transboundary aquifers along the Mexico–US border: An initial assessment. Journal of Hydrology, 535, 101-119.	Flora, S., Davis, T. (2009). Hydrologic Map Series (HMS), Water Level Change Map Series (WLCMS), and Basin Sweep Assessment Report ADWR Basins and Sub-Basins. Arizona Department of Water Resources Hydrology Division Field Services Section https://new.azwater.gov/sites/default/files/HMSWLCMSBasinSweepAssessmentReport2009.pdf	USA portion approximated from Fig. 1 of Flora and Davis (2009). Hydrologic Map Series (HMS), Water Level Change Map Series (WLCMS), and Basin Sweep Assessment Report ADWR Basins and Sub-Basins. Arizona Department of Water Resources Hydrology Division Field Services Section https://new.azwater.gov/sites/default/files/HMSWLCMSBasinSweepAssessmentReport2009.pdf ("Allen Flat" basin included with "Sierra Vista" basin). Mexico Portion estimate from Fig. 3 of Sanchez et al. (2016)
San Simon Valley	Schwennesen, A.T., Forbes, R.H. (1919). Ground water in San Simon Valley, Arizona and New Mexico. U.S. Geological Survey Water Supply Paper 425, 161 pp. Accessed March 29, 2021 from https://pubs.usgs.gov/wsp/0425a/report.pdf	-	-	Approximated from plate 1 of Schwennesen and Forbes (1919)
Sanpete Valley	Richardson, G. B. (1907). Underground water in Sanpete and Central Sevier Valleys, Utah. U.S. Geological Survey Water-Supply and Irrigation Paper No. 199, 77 pp. Accessed March 7, 2021 from https://pubs.usgs.gov/wsp/0199/report.pdf	-	-	Approximated from Fig. 1 of Richardson (1907)
Santa Barbara and Foothill Basin	Nishikawa, T., ed. (2018). Santa Barbara and Foothill groundwater basins Geohydrology and optimal water resources management—Developed using density dependent solute transport and optimization models, U.S. Geological Survey, Scientific Investigations Report 2018-5059. Accessed March 20, 2021 from https://pubs.usgs.gov/sir/2018/5059/sir20185059_.pdf	-	-	Approximated from Fig. 1 of Nishikawa (2018)
Santa Clara Valley	Hanson, R. T., (2015). Hydrologic framework of the Santa Clara Valley, California. Geosphere 11, doi:10.1130/GES01104.1 Accessed March 27, 2021 from https://ca.water.usgs.gov/pubs/2015/Hanson2015.pdf	-	-	Approximated from Fig. 1 of Hanson (2015)
Santa Clara-Calleguas Basin	Hanson, R.T., Martin, P., Koczot, K.M. (2002). Simulation of ground-water/surface-water flow in the Santa Clara-Calleguas ground-water basin, Ventura County, California. Water-Resources Investigations Report 2002-4136, 172 pp. Accessed March 20, 2021 from https://pubs.usgs.gov/wri/wri024136/wrir024136.pdf	-	-	Approximated from Fig. 7 of Hanson et al. (2002)
Santa Maria Basin and Nipomo Valley	Hughes, J. L. (1977). Evaluation of Ground-water Quality in the Santa Maria Valley, California. US Geological Survey, Water Resources Division Report 76-128,. 77 pp. Accessed March 7, 2021 from https://pubs.usgs.gov/wri/1976/0128/report.pdf	Worts, G. F., Thomasson, H. G. (1951). Geology and ground-water resources of the Santa Maria Valley Area, California. US Geological Survey Water Supply Paper 1000, 177 pp. Accessed March 7, 2021 from https://pubs.usgs.gov/wsp/1000/report.pdf	-	Approximated from Fig. 1 of Hughes (1977) and Fig. 1 by Worts and Thomasson (1951)

Aquifer	Reference 1	Reference 2	Reference 3	Steps taken to delineate
Santa Rosa Valley	Valin, Z.C., McLaughlin, R. J. (2005). Locations and data for water wells of the Santa Rosa Valley, Sonoma County, California. U.S. Geological Survey Open File Report 2005-1318. 16 pp. Accessed March 12, 2021 from https://pubs.usgs.gov/of/2005/1318/of2005-1318.pdf	Cardwell, G. T. (1958). Geology and ground water in the Santa Rosa and Petaluma Valley areas, Sonoma County, California. U.S. Geological Survey Water-Supply Paper 1427, 284 pp. Accessed November 29, 2021 from https://pubs.usgs.gov/wsp/1427/report.pdf	-	Approximated from map on page 2 of Valin and McLaughlin (2005) and Fig. 1 by Cardwell (1958)
Sawtooth Valley and Bear Valley	Graham, W. G., Campbell, L. J. (1981). Groundwater resources of Idaho. Idaho Department of Water Resources Report, 61 pp. Accessed March 23, 2021 from https://idwr.idaho.gov/files/publications/198108-MISC-GW-Resources-ID.pdf	-	-	Approximated from Plate 1 of Graham and Campbell (1981)
Scott Valley	Wood, P. R. (1960). Geology and ground-water features of the Butte Valley region, Siskiyou County, California. Geological Survey Water-Supply Paper 1491, 155 pp. Accessed March 20, 2021 from https://pdfs.semanticscholar.org/a448/a58a3c1ac120d400d26f75b74512f0a868a4.pdf	-	-	Approximated from Fig. 1 of Wood (1960)
Scott Valley and Mann Creek	Graham, W. G., Campbell, L. J. (1981). Groundwater resources of Idaho. Idaho Department of Water Resources Report, 61 pp. Accessed March 23, 2021 from https://idwr.idaho.gov/files/publications/198108-MISC-GW-Resources-ID.pdf	-	-	Approximated from Plate 1 of Graham and Campbell (1981)
Sevier Desert	Mower, R. W., Feltis, R. D. (1968). Ground-water hydrology of the Sevier Desert, Utah. US Geological Survey, Water Supply Paper 1854, 88 pp. Accessed March 7, 2021 from https://pubs.usgs.gov/wsp/1854/report.pdf	-	-	Approximated from Fig. 1 of Mower and Feltis (1968)
Seymour-Blaine Aquifer System	Bruun, B., Jackson, K., Lake, P., Walker, J. (2016). Texas Aquifers Study. Texas Water Development Board Report. 336 pp. Accessed April 1, 2021 from https://www.twdb.texas.gov/groundwater/docs/studies/TexasAquifersStudy_2016.pdf#page=89	Chastain-Howley, A., Dean, K.E., Spear, A.A. (2004). Groundwater Availability Model for the Seymour Aquifer, Texas Water Development Board Report, 533 pp. Accessed April 5, 2021 from https://www.twdb.texas.gov/groundwater/models/gam/symr/symr.asp	-	Approximated from Fig. 6-33 by Bruun et al. (2016) and map by Chastain-Howley et al. (2004) available at https://www.twdb.texas.gov/groundwater/models/gam/symr/symr.asp (Accessed April 5, 2021)
Shasta Valley	Wood, P. R. (1960). Geology and ground-water features of the Butte Valley region, Siskiyou County, California. Geological Survey Water-Supply Paper 1491, 155 pp. Accessed March 20, 2021 from https://pdfs.semanticscholar.org/a448/a58a3c1ac120d400d26f75b74512f0a868a4.pdf	-	-	Approximated from Fig. 1 of Wood (1960)
Sierra Valley	Bachand, P.A.M., Birt, K.S., Bachand, S.M. (2020). Groundwater relationships to pumping, precipitation and geology in high-elevation basin, Sierra Valley, CA. Technical Report to Report to Feather River Land. Sierra Valley, CA. 66 pp. Accessed March 21, 2021 from http://aquaticcommons.org/27004/1/Sierra%20Valley%20Recharge%20FINAL%202020-03-10%20SECURE.pdf	-	-	Approximated from Fig. 4 of Bachand et al. (2020)
Silurian-Devonian Aquifers in Northern Michigan	Olcott, P.G. (1992). Groundwater Atlas of the United States: Segment 9 Iowa, Michigan, Minnesota, Wisconsin. U.S. Geological Survey Hydrologic Atlas 730-J, 33 pp. Accessed April 12, 2021 from https://pubs.usgs.gov/ha/730j/report.pdf	-	-	Approximated from Fig. 79 by Olcott (1992)
Silver State Valley in northern Nevada	Lopes, T. J. (2010). Hydrologic Evaluation of the Jungo Area, Southern Desert Valley, Nevada. U. S. Geological Survey Open-File Report 2010-1009, 18 pp.	-	-	Approximated from Fig. 2 of Lopes (2010)

Aquifer	Reference 1	Reference 2	Reference 3	Steps taken to delineate
	Accessed March 23, 2021 from https://pubs.usgs.gov/of/2010/1009/pdf/ofr20101009.pdf			
Smith Valley	Loeltz, O. J., Eakin, T. E. (1953). Geology and water resources of Smith Valley, Lyon and Douglas Counties, Nevada. U.S. Geological Survey Water-Supply Paper 1228, 94 pp. Accessed March 21, 2021 from https://pubs.usgs.gov/wsp/1228/report.pdf	-	-	Approximated from Plate 2 of Loeltz and Eakin (1953)
Snake Valley	Gardner, P. M., Masbruch, M. D. (2015). Hydrogeologic and geochemical characterization of groundwater resources in Deep Creek Valley and adjacent areas, Juab and Tooele Counties, Utah, and Elko and White Pine Counties, Nevada. US Geological Survey Scientific Investigations Report 2015–5097, 66 pp. Accessed March 10, 2021 from https://pubs.usgs.gov/sir/2015/5097/sir20155097.pdf	-	-	Approximated from Fig. 6 of Gardner and Masbruch (2015)
South Park Basin	Barkmann, P. E., Broes, L.D., Palkovic, M.J., Hopkins, J.C., Bird, K.S., Sebol, L.A., Fitzgerald. F.S. (2020). ON-010 Colorado Groundwater Atlas. Geohydrology. Colorado Geological Survey, Golden, CO. ON-010 Colorado Groundwater Atlas. Accessed March 29, 2021 from https://coloradogeologicalsurvey.org/water/colorado-groundwater-atlas/	Donegan, K.C. (2018). Groundwater levels in the South Park Basin 2018. Colorado Division of Water Resources Report, 29 pp. Accessed November 29, 2021 from https://dnrweblink.state.co.us/dwr/ElectronicFile.aspx?docid=3351305&dbid=0	Barkmann, P.E., Moore, A., Johnson, J. (2013) South Park Groundwater Quality Scoping Study. Report for Coalition for the Upper South Platte, 74 pp. Accessed November 29, 2021 from https://cusp.ws/wp-content/uploads/2014/10/South-Park-Groundwater-Quality-Scoping-Study_Final.pdf	Approximated from Fig. 12-01 of Barkmann et al. (2020), Fig. 1a by Donegan (2018), and Fig. 1 by Barkmann et al. (2013)
South Platte Basin	Wellman, T.P. (2015). Evaluation of groundwater levels in the South Platte River alluvial aquifer, Colorado, 1953–2012, and design of initial well networks for monitoring groundwater levels. U.S. Geological Survey Scientific Investigations Report 2015–5015, 67 pp., Accessed March 29, 2021 from https://pubs.usgs.gov/sir/2015/5015/pdf/sir2015-5015.pdf	-	-	Approximated from Fig. 3 of Wellman (2015)
Southern San Juan Basin	Kernodle, J. M. (1996). Hydrogeology and steady-state simulation of ground-water flow in the San Juan Basin, New Mexico, Colorado, Arizona, and Utah. US Geological Survey Water-Resources Investigations Report 95-4187, 126 pp. Accessed March 12, 2021 from https://pubs.usgs.gov/wri/1995/4187/report.pdf	-	-	Approximated from Fig. 3 of Kernodle (1996). Southern portion approximated on the basis of groundwater well density (derived from New Mexico's well completion dataset)
Southern Utah Valley	Brooks, L.E. (2013). Evaluation of the groundwater flow model for southern Utah and Goshen Valleys, Utah, updated to conditions through 2011, with new projections and groundwater management simulations: U.S. Geological Survey Open-File Report 2013–1171, 35 pp. Accessed March 7, 2021 from https://pubs.usgs.gov/of/2013/1171/pdf/ofr2013-1171.pdf	Selck, B. J., Carling, G. T., Kirby, S. M., Hansen, N. C., Bickmore, B. R., Tingey, D. G., Rey, K., Wallace, J. Jordan, J. L. (2018). Investigating anthropogenic and geogenic sources of groundwater contamination in a semi-arid alluvial basin, Goshen Valley, UT, USA. Water, Air, Soil Pollution, 229(6), 1-17.	-	Approximated from Fig. 1 of Brooks (2013)
Spanish Springs Valley	Berger, D. L., Ross, W. C., Thodal, C. E., Robledo, A. R. (1997). Hydrogeology and simulated effects of urban development on water resources of Spanish Springs Valley, Washoe County, West-Central Nevada. U.S. Geological Survey Water-Resources Investigations Report 96-4297, 87 pp. Accessed March 22, 2021 from https://pubs.usgs.gov/wri/1996/4297/report.pdf	-	-	Approximated from Fig. 4 of Berger et al. (1997)
Spring Valley	Gardner, P. M., Masbruch, M. D. (2015). Hydrogeologic and geochemical characterization of groundwater resources in Deep Creek Valley and adjacent	-	-	Approximated from Fig. 6 of Gardner and Masbruch (2015)

Aquifer	Reference 1	Reference 2	Reference 3	Steps taken to delineate
	areas, Juab and Tooele Counties, Utah, and Elko and White Pine Counties, Nevada. US Geological Survey Scientific Investigations Report 2015–5097, 66 pp. Accessed March 10, 2021 from https://pubs.usgs.gov/sir/2015/5097/sir20155097.pdf			
Star Valley	Walker, E.H. (1965). Ground-water in the upper Star Valley, Wyoming. U.S. Geological Survey Water Supply Paper 1809-C, 33 pp. Accessed March 29, 2021 from https://pubs.usgs.gov/wsp/1809c/report.pdf	-	-	Approximated from Fig. 1 of Walker (1965)
Steptoe Valley	Frick, E. (1985). Quantitative analysis of groundwater flow in valley-fill deposits in Steptoe Valley, Nevada. Doctoral dissertation, University of Nevada, Reno. 199 pp. Accessed March 10, 2021 from https://scholarworks.unr.edu/bitstream/handle/11714/1293/Mackay219_Frick.pdf?sequence=1	-	-	Approximated from Fig. 1 of Frick (1985)
Surface and Tongue Basins	Kolm, K.E., van der Heijde, P.K.M. (2014). Groundwater Systems In Delta County, Colorado: Surface Creek Valley Area. Report prepared for Delta County Board of County Commissioners, Colorado, 64 pp. Accessed March 28, 2021 from https://www.chc4you.org/wp-content/uploads/2017/01/Surface-Creek-Hydrology-Report-2014.pdf	-	-	Approximated from Fig. 25b of Kohm and van der Heijde (2014)
Surprise Valley	Cantwell, C.A., Fawler, A.P.G. (2014). Fluid Geochemistry of the Surprise Valley Geothermal System. Proceedings of the Thirty-Ninth Workshop on Geothermal Reservoir EngineeringStanford University, Stanford, California, February 24-26, 2014SGP-TR-202. Accessed March 27, 2021 from https://pangea.stanford.edu/ERE/pdf/IGAstandard/SGW/2014/Cantwell.pdf	-	-	Approximated from Fig. 1 of Cantwell and Fowler (2014)
Teton Valley	Graham, W. G., Campbell, L. J. (1981). Groundwater resources of Idaho. Idaho Department of Water Resources Report, 61 pp. Accessed March 23, 2021 from https://idwr.idaho.gov/files/publications/198108-MISC-GW-Resources-ID.pdf	-	-	Approximated from Plate 1 of Graham and Campbell (1981)
Three Lakes Valley	Burbey, T. J. (1997). Hydrogeology and potential for ground-water development, carbonate-rock aquifers in southern Nevada and southeastern California. US Geological Survey Water-Resources Investigations Report 95-4168, 70 pp. Accessed March 10, 2021 from https://pubs.usgs.gov/wri/1995/4168/report.pdf	-	-	Approximated from Fig. 12 of Burbey (1997)
Tijuana-San Diego Basin	International Hydrological Programme, Division of Water Sciences (2009). Atlas of Transboundary Aquifers. UNESCO Report, 322 pp. Accessed March 21, 2021 from https://isarm.org/sites/default/files/resources/files/2%20Atlas%20of%20TBA.pdf	-	-	Approximated from map on page 99 of International Hydrological Programme, Division of Water Sciences (2009)
Tillman Terrace	Osborn, N.I. (2002). Update of the Hydrologic Survey of the Tillman Terrace Groundwater Basin, Southwestern Oklahoma. Oklahoma Water Resources Board Technical Report GW2002-1, 21 pp., Accessed April 5, 2021 from https://www.owrb.ok.gov/studies/reports/reports_pdf/tillman_update.pdf	-	-	Approximated from well completion data and Fig. 1 of Osborn (2002)
Tippett and Antelope Valleys	Gardner, P. M., Masbruch, M. D. (2015). Hydrogeologic and geochemical characterization of groundwater resources in Deep Creek Valley and adjacent areas, Juab and Tooele Counties, Utah, and Elko and White Pine Counties, Nevada. US Geological Survey Scientific Investigations Report 2015–5097, 66 pp. Accessed March 10, 2021 from https://pubs.usgs.gov/sir/2015/5097/sir20155097.pdf	-	-	Approximated from Fig. 6 of Gardner and Masbruch (2015)
Toole Valley	Thomas, H. E. (1946). Ground water in Tooele Valley, Tooele County, Utah (No. 4, pp. 91-238). Utah Department of Natural Resources, Division of Water Rights. 148 pp. Accessed March 7, 2021 from https://waterrights.utah.gov/docSys/v920/w920/w9200083.pdf	-	-	Approximated from Fig. 1 of Gardner and Kirby (2011)
Transboundary Santa Cruz Basin	Callegary, J.B., Minjárez Sosa, I., Tapia Villaseñor, E.M., dos Santos, P., Monreal Saavedra, R., Grijalva Noriega, F.J., Huth, A.K., Gray, F., Scott, C.A.,	Sanchez, R., Lopez, V., Eckstein, G. (2016). Identifying and	Flora, S., Davis, T. (2009). Hydrologic Map Series	USA portion approximated from Fig. 1 of Flora and Davis (2009). Hydrologic Map Series

Aquifer	Reference 1	Reference 2	Reference 3	Steps taken to delineate
	Megdal, S.B., Oroz Ramos, L.A., Rangel Medina, M., Leenhouts, J.M. (2016). San Pedro River Aquifer Binational Report: International Boundary and Water Commission. 173 pp. Accessed February 12, 2021 via https://www.ibwc.gov/Files/Binational_Study_Transboundary_San_Pedro_Aquifer.pdf	characterizing transboundary aquifers along the Mexico–US border: An initial assessment. Journal of Hydrology, 535, 101-119.	(HMS), Water Level Change Map Series (WLCMS), and Basin Sweep Assessment Report ADWR Basins and Sub-Basins. Arizona Department of Water Resources Hydrology Division Field Services Section https://new.azwater.gov/sites/default/files/HMSWLCMSBasinSweepAssessmentReport2009.pdf	(HMS), Water Level Change Map Series (WLCMS), and Basin Sweep Assessment Report ADWR Basins and Sub-Basins. Arizona Department of Water Resources Hydrology Division Field Services Section https://new.azwater.gov/sites/default/files/HMSWLCMSBasinSweepAssessmentReport2009.pdf. Mexico Portion estimate from Fig. 3 of Sanchez et al. (2016)
Uncompahgre Basin	Craig, T.W. (1971). Ground water of the Uncompahgre Valley Montrose County, Colorado. MSc Thesis, University of Missouri-Rolla, 119 pp. Accessed March 28, 2021 from https://scholarsmine.mst.edu/cgi/viewcontent.cgi?article=6120&context=masters_theses	-	-	Approximated from Fig. 5 of Craig et al. (1971)
Upper Arkansas River Basin	Watts, K.R. (2005). Hydrogeology and quality of ground water in the upper Arkansas River Basin from Buena Vista to Salida, Colorado, 2000-2003. U.S. Geological Survey Scientific Investigations Report 2005-5179, 61 pp. Accessed March 27, 2021 from https://pubs.usgs.gov/sir/2005/5179/pdf/SIR2005-5179.pdf	-	-	Approximated from Fig. 1 by Watts (2005)
Upper Beaverhead Basin	Uthman, W., Beck J. (1998). Hydrogeology of the Upper Beaverhead Basin near Dillon, Montana. Montana Bureau of Mines and Geology Open-File Report 384, 605 pp. Accessed March 29, 20221 from http://dnrc.mt.gov/divisions/water/management/docs/ground-water-studies/hydrogeology_upper_beaverhead_near_dillon.pdf	-	-	Approximated from Fig. 1 of Uthman and Beck (1998); northeast boundary extended beyond Fig. 1 outline based on well completion data
Upper Deschutes Basin	Gannett, M.W., Lite, Jr., K.E., Morgan, D.S., and Collins, C.A. (2001). Ground-water hydrology of the upper Deschutes Basin, Oregon: U.S. Geological Survey Water-Resources Investigations Report 00-4162, 74 p. Accessed February 24, 2021 from https://pubs.usgs.gov/wri/wri004162/	Oregon Water Resources Department (2021). Well Report Query. Web Page Accessed February 24, 2021 from https://apps.wrd.state.or.us/apps/gw/well_log/Default.aspx	-	Approximated from Fig.1 of Gannett et al. (2001) and influenced by the spatial distribution of groundwater wells recorded by the Oregon Water Resources Department (2021)
Upper Humboldt Basin	Plume, R. W. (2009). Hydrogeologic Framework and Occurrence and Movement of Ground Water in the Upper Humboldt River Basin, Northeastern Nevada. US Geological Survey Scientific Investigations Report 2009–5014, 30 pp. Accessed March 2021 from https://pubs.usgs.gov/sir/2009/5014/pdf/sir20095014.pdf	-	-	Approximated from Plate 1 of Plume (2009)
Upper Reese River Valley in northern Nevada	Bredehoeft, J.D., Farvolden, R.N. (1963). International Association of Scientific Hydrology, Commission of Subterranean Waters, Publication no. 64, p. 197–212. Accessed March 23, 2021 from http://hydrologie.org/redbooks/a064/064017.pdf	-	-	Approximated from Fig. 1 of Bredehoft and Farvolden (1963) and guided by locations of wells and topography (especially to distinguish upper and lower portions of basin)
Upper Salmon Basin	Graham, W. G., Campbell, L. J. (1981). Groundwater resources of Idaho. Idaho Department of Water Resources Report, 61 pp. Accessed March 23, 2021 from https://idwr.idaho.gov/files/publications/198108-MISC-GW-Resources-ID.pdf	-	-	Approximated from Plate 1 of Graham and Campbell (1981)
Upper Santa Ana Basin	Kent, R., Belitz, K. (2009). Ground-water quality data in the Upper Santa Ana Watershed Study Unit, November 2006 to March 2007: Results from the California GAMA Program: U.S. Geological Survey Data Series 404, 116 pp. Accessed March 21, 2021 from https://pubs.usgs.gov/ds/404/ds404.pdf	-	-	Approximated from Fig. 2 of Kent and Belitz (2009)

Aquifer	Reference 1	Reference 2	Reference 3	Steps taken to delineate
Utah Lake Valley	Dustin, J.D. (1978). Hydrogeology of Utah Lake with Emphasis on Goshen Bay. PhD Dissertation, Brigham Young University. 184 pp. Accessed March 27, 2021 from https://apps.dtic.mil/sti/pdfs/ADA065478.pdf	-	-	Approximated from Fig. 1 of Dustin (1978)
Uvas Valley	State of New Mexico Office of the State Engineer (1998). Nutt-Hockett Basin Hydrographic Survey Report. 267 pp. Accessed March 27, 2021 from https://www.ose.state.nm.us/HydroSurvey/nutt-hockett/report.pdf	-	-	Approximated from map on page vi of State of New Mexico, Office of the State Engineer (1998)
Vamoosa Ada Aquifer	D'Lugosz, J.J., McClaflin, R.G. (1986). Geohydrology of the Vamoosa-Ada aquifer east-central Oklahoma with a section on chemical quality of water. U.S. Geological Survey Circular 87, 48 pp. Accessed April 5, 2021 from http://www.ogs.ou.edu/pubsscanned/Circulars/circular87mm.pdf	-	-	Approximated from plate 1 of D'Lugosz and McClaflin (1986)
Warm Springs Valley	Glenn, R.J. (1968). Water resources of Warm Springs Valley, Washoe County, Nevada. University of Nevada, Reno, Geological Sciences and Engineering Thesis 98 pp. Accessed March 21, 2021 from https://scholarworks.unr.edu/handle/11714/1315	-	-	Approximated from Plate 1 of Glenn (1968)
Weiser River Valley	Graham, W. G., Campbell, L. J. (1981). Groundwater resources of Idaho. Idaho Department of Water Resources Report, 61 pp. Accessed March 23, 2021 from https://idwr.idaho.gov/files/publications/198108-MISC-GW-Resources-ID.pdf	-	-	Approximated from Plate 1 of Graham and Campbell (1981)
West Bench	Carstarphen, C., Patton, T., and LaFave, J.I. (2014). Water levels in the Upper West Bench alluvial aquifer, Red Lodge, Montana: Montana Bureau of Mines and Geology Information Pamphlet 8, 8 pp. Accessed March 29, 2021 from http://mbmg.mtech.edu/pdf-publications/ip_8.pdf	-	-	Approximated from Fig. 1 of Carstarphen et al. (2014)
Western Champlain Valley Lowlands	Nystrom, E.A. (2006). Ground-water quality in the Lake Champlain Basin, New York, 2004. U.S. Geological Survey Open-File Report 2006-1088, 22 pp. Accessed April 15, 2021 from https://pubs.usgs.gov/of/2006/1088/pdf/Nystrom.OFR2006-1088.pdf	Scott, T.-M., Nystrom, E.A., Reddy, J.E. (2016). Groundwater quality in the Lake Champlain and Susquehanna River basins, New York, 2014. U.S. Geological Survey Open-File Report 2016–1153, 33 pp. Accessed April 15, 2021 from https://pubs.usgs.gov/of/2016/1153/ofr20161153.pdf	-	Approximated from Fig. 2 of Nystrom (2006) and Fig. 1 by Scott et al. (2016)
Western Kankakee Plains	Fenneman, N.M., Johnson, D.W. (1946). Physiographic divisions of the conterminous United States. U.S. Geological Survey map, 1:7,000,000 Scale.	Leighton, M. M., Ekblaw, G. E., Horberg, L. (1948). Physiographic divisions of Illinois. The Journal of Geology, 56, 16-33.	-	Broader Till Plain physiographic region approximated from Fenneman and Johnson (1946). Kankakee Plain in Illinois approximated from Figs. 1 and 2 of Leighton et al. (1948). Eastern margin of subarea approximated along the Iroquois River
Wet Mountain Valley	Londquist, C. J., Livingston, R.K. (1978). Water-resources appraisal of the Wet Mountain Valley, in parts of Custer and Fremont Counties, Colorado. Water-Resources Investigations 78-1, 62 pp. Accessed March 27, 2021 from https://pubs.usgs.gov/wri/1978/0001/report.pdf	-	-	Approximated from Fig. 3 of Londquist and Livingston (1978)
White River Valley	Maxey, G. B., Eakin, T. E. (1949). Ground water in White River Valley, White Pine, Nye, and Lincoln Counties, Nevada. U.S. Department of the Interior Water Resources Bulletin 8, 64 pp. Accessed March 10, 2021 from https://www.nrc.gov/docs/ML0331/ML033140348.pdf	-	-	Approximated from maps on page 63 and 64 of Maxey and Eakin (1949)
Willcox-Douglas Basin	Arizona Department of Water Resources (2018). Groundwater Flow Model of the Willcox Basin, Arizona Department of Water Resources Report, 196 pp. Accessed on February 12, 2021 from https://new.azwater.gov/sites/default/files/Willcox_Report_2018.pdf	Flora, S., Davis, T. (2009). Hydrologic Map Series (HMS), Water Level Change Map Series (WLCMS), and Basin Sweep Assessment Report ADWR Basins and Sub-Basins. Arizona	-	Approximated from Arizona Department of Water Resources (2018). This report states (quote AZ DWR): "..the Willcox Basin is a closed basin that is in direct hydraulic connection with the Aravaipa and Douglas basins to the north and south, respectively."

Aquifer	Reference 1	Reference 2	Reference 3	Steps taken to delineate
		Department of Water Resources Hydrology Division Field Services Section https://new.azwater.gov/sites/default/files/HMSWLCMSBasinSweepAssessmentReport2009.pdf		And thus the Aravaipa and Douglas Basins of Flora and Davis (2009) were included in the delineated boundary for the Willcox Basin
Wisconsin Precambrian Aquifer	U.S. Geological Survey (1984). National water summary 1984: Hydrologic events, selected water-quality trends, and ground-water resources. U.S. Geological Survey Water Supply Paper 2275, 477 pp. Accessed April 13, 2021 from https://pubs.usgs.gov/wsp/2275/report.pdf	-	-	Approximated from Fig. 1 of the "National Water Summary-Wisconsin" (page 449) by the U.S. Geological Survey (1984)
Wood River Valley	Graham, W. G., Campbell, L. J. (1981). Groundwater resources of Idaho. Idaho Department of Water Resources Report, 61 pp. Accessed March 23, 2021 from https://idwr.idaho.gov/files/publications/198108-MISC-GW-Resources-ID.pdf	-	-	Approximated from Plate 1 of Graham and Campbell (1981)
Yuma Basin	Olmsted, F. H., Loeltz, O. J., Irelan, B. (1973). Geohydrology of the Yuma area, Arizona and California. US Geological Survey Professional Paper 486-H, 237 pp. Accessed February 15, 2021 from https://pubs.usgs.gov/pp/0486h/report.pdf	-	-	Approximated from Fig. 2 of Olmsted et al. (1973); southwestern margin approximated by Colorado River

Supplementary Note 3.2 – Rationale for developing a new geodatabase of US aquifer systems

Here we provide several examples of aspects of the USGS' Principal Aquifer database available for download here (accessed January 14, 2022 https://water.usgs.gov/GIS/metadata/usgswrd/XML/aquifers_us.xml) that motivated us not to use that database in this study and, instead, create a new geodatabase of aquifer boundaries.

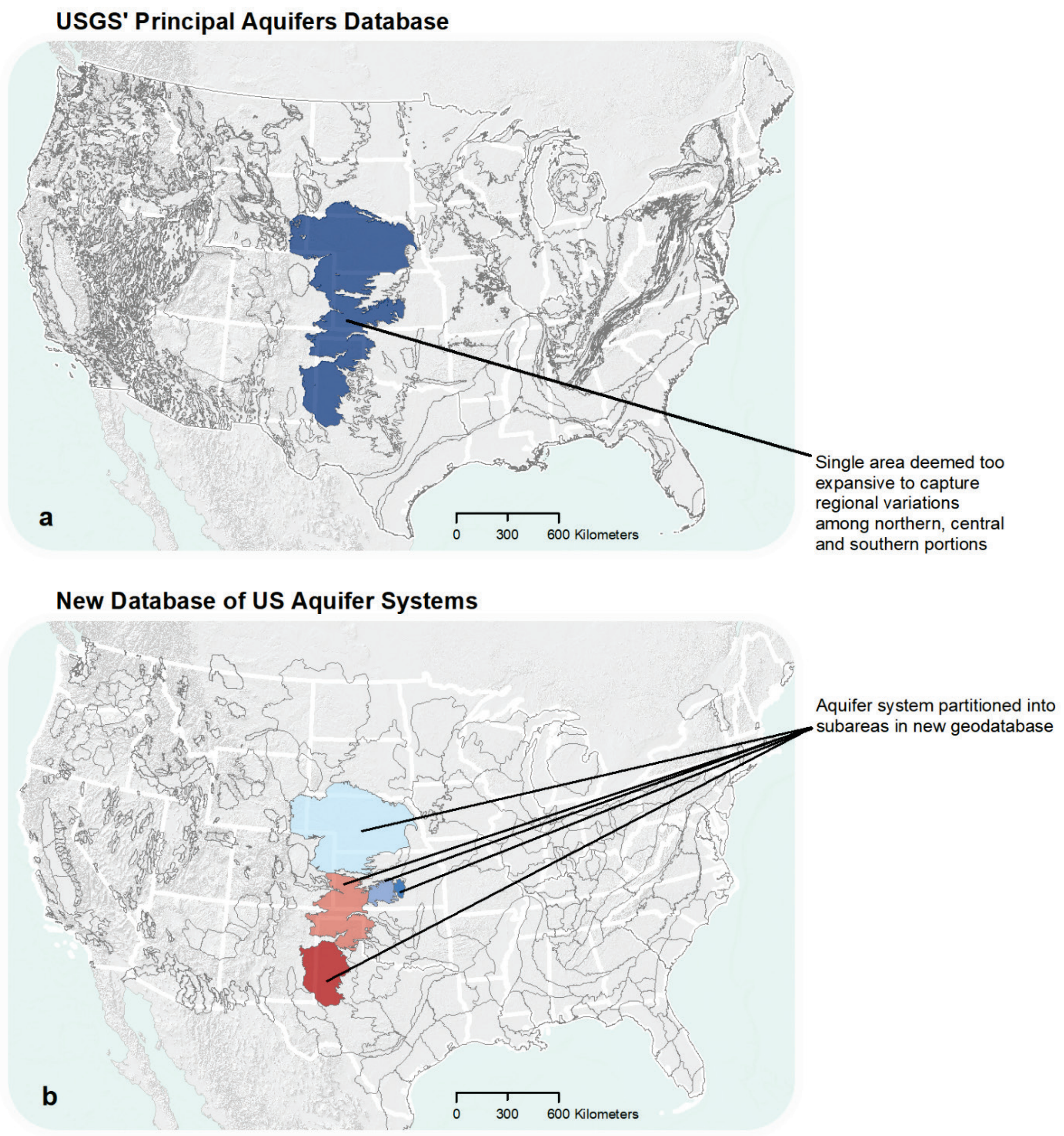

Supplementary Fig. 6. Comparison of Principal Aquifer geodatabase (top panel "a") and new aquifer boundaries delineated for this study (lower panel "b"). The Principal Aquifer (accessed Jan-14-2022 via https://water.usgs.gov/GIS/metadata/usgswrd/XML/aquifers_us.xml) area for the High Plains (blue) was deemed too expansive to capture important variations in well depths and hydrogeologic conditions among the different subareas of the High Plains (at its area of approximately 450 thousand km^2). We have partitioned the aquifer into five subareas on the basis of locally relevant research (panel "b"; see Supplementary Note 3.1).

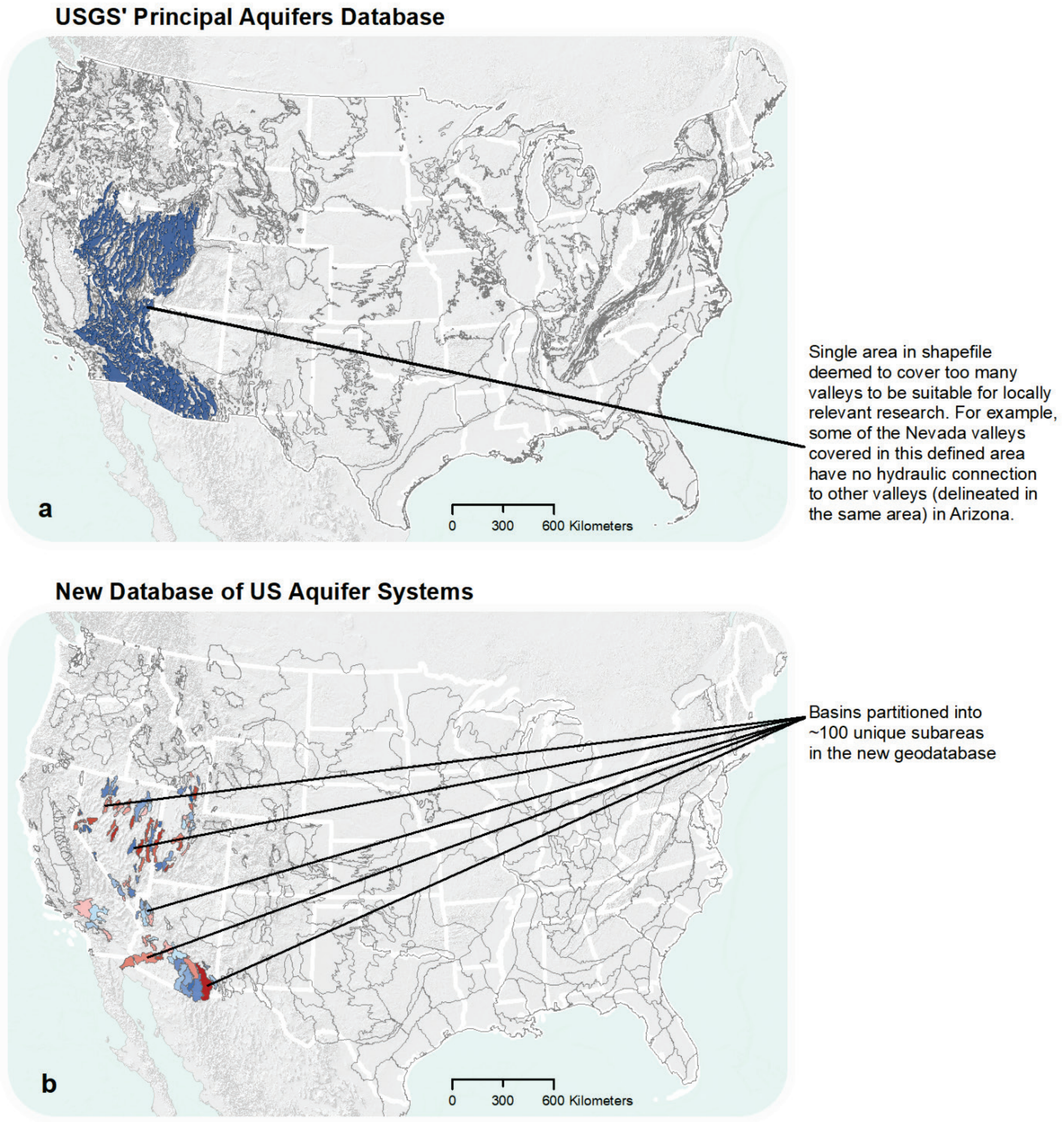

Supplementary Fig. 7. Comparison of Principal Aquifer geodatabase (top panel "a") and new aquifer boundaries delineated for this study (lower panel "b"). The Principal Aquifer (accessed Jan-14-2022 via https://water.usgs.gov/GIS/metadata/usgswrd/XML/aquifers_us.xml) area for one of the "Basin and Range basin-fill aquifers" is both too expansive for locally relevant research (~350 thousand km^2 in just this one polygon) and also combines some valleys that do not share a hydraulic connection into one area (e.g., valleys in northwestern Nevada and valleys in southeastern Arizona are included in this same polygon). We have delineated ~100 unique valleys on the basis of local hydrogeologic research (panel "b"; see Supplementary Note 3.1).

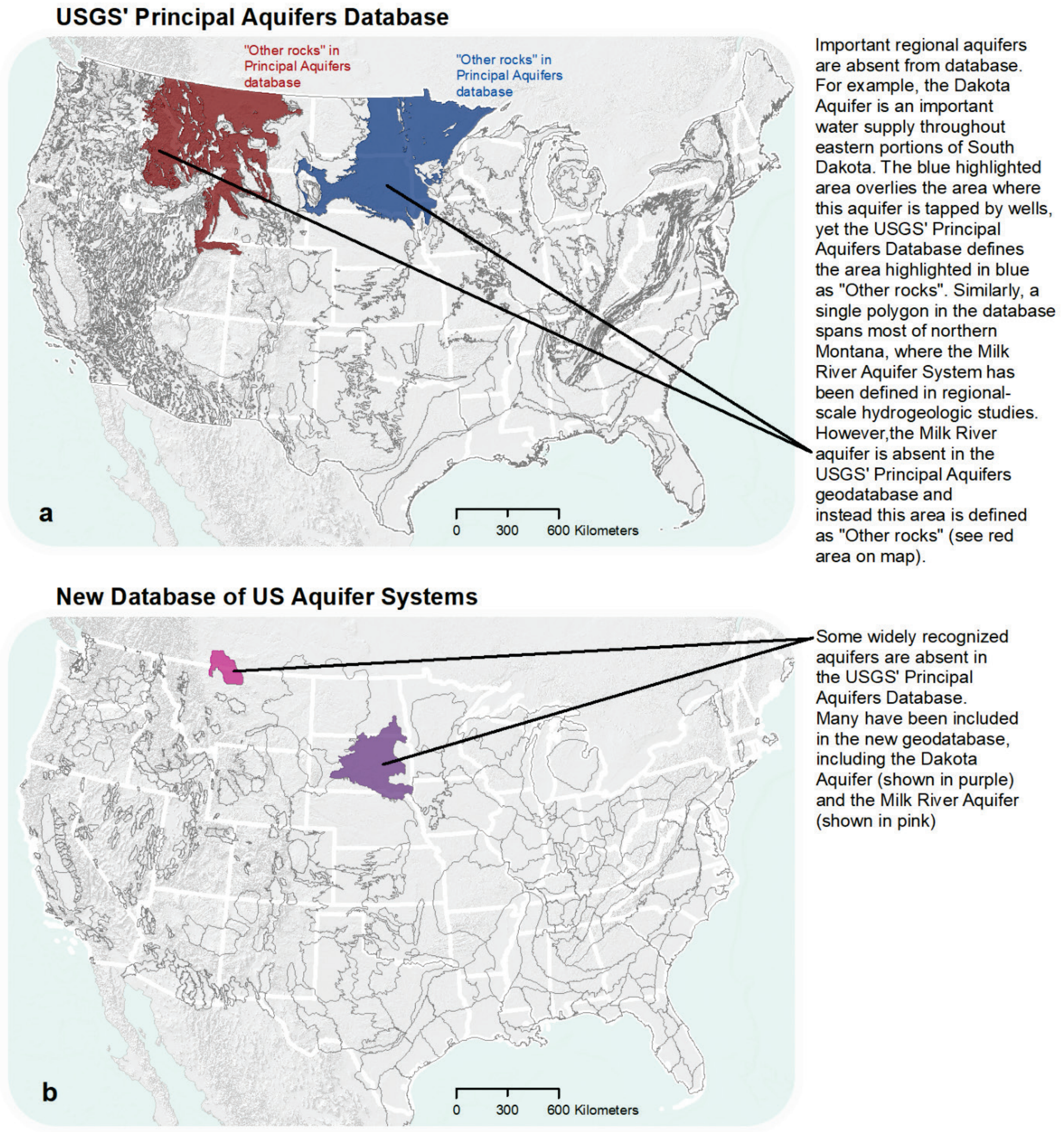

Supplementary Fig. 8. Comparison of Principal Aquifer geodatabase (top panel "a") and new aquifer boundaries delineated for this study (lower panel "b"). The Principal Aquifer database does not include the Dakota Aquifer of South Dakota, which was characterized in a USGS Water Supply Paper more than a century ago (Darton, N.H. (1909). Geology and underground waters of South Dakota. US Geological Survey Water-Supply Paper 227, 181 pp. Accessed July 13, 2021 from https://pubs.usgs.gov/wsp/0227/report.pdf). We have added the Dakota Aquifer (in eastern South Dakota, southeastern North Dakota, and northeastern Nebraska) to the new geodatabase (panel "b"; see Supplementary Note 3.1).

Supplementary Note 4 – Hydrostratigraphy in 12 aquifer systems with fossil water

The first figure (i.e., Fig. 1) in the main text presents cross sections for twelve aquifer systems across the United States; fossil groundwater has been reported in well waters derived from each of the twelve aquifer systems. Here we provide details and citations (including quotes from cited papers) highlighting how Fig. 1 was developed.

Supplementary Note 4.1 – Categorizing hydrostratigraphy based on local-scale studies

Here we provide quotes from a suite of regional-scale hydrogeologic studies that describe how each layer was categorized into one of the broad hydrogeologic categories (e.g., carbonate aquifer; endogenous bedrock).

Supplementary Table 5. Hydrostratigraphy details for the Cuyama Valley, California.

Formation name	Category	Quote
Alluvial aquifers	Unconsolidated aquifer	"Geologic maps of the Cuyama Valley (Vedder and Repenning, 1975; Kellogg and others, 2008) show two units of Holocene and Pleistocene-aged alluvial deposits, termed younger and older alluvium, underlying the three monitoring well sites." Everett et al., 2013. "The main water-bearing deposits in the study area are the saturated portions of the younger and older alluvium and the Morales Formation." (Everett et al. 2013)
Morales formation aquifer	Clastic sedimentary rock **aquifer** (consolidated or semi-consolidated rock)	"The Morales Formation (QTm, fig. 2) is a Pliocene Pleistocene fluvial deposit that is up to 5,000 feet thick and consists of massive- to thick-bedded, partly consolidated deposits of clay, silt, sand, and gravel (Hill and others, 1958; Ellis and others, 1993).". **Everett** et al., 2013. "The main water-bearing deposits in the study area are the saturated portions of the younger and older alluvium and the Morales Formation." (Everett et al. 2013)
Bedrock	Sedimentary rock **aquitard** (consolidated or semi-consolidated rock) OR Endogenous bedrock *(*for display purposes, we show a sedimentary rock aquitard in Fig. 1)*	"non-water-bearing rocks—the crystalline granitic rocks and all consolidated sedimentary rocks older than the Morales Formation". **Everett** et al., 2013. "All rocks that are older than the Morales Formation were considered by previous investigators to be non-water-bearing" (Everett et al., 2013)

Everett, R. R., Gibbs, D. R., Hanson, R. T., Sweetkind, D. S., Brandt, J. T., Falk, S. E., Harich, C. R. (2013). Geology, water-quality, hydrology, and geomechanics of the Cuyama Valley groundwater basin, California, 2008-12. US US Geological Survey Scientific Investigations Report 2013–5108, 76 pp. Accessed February 21, 2022 from https://pubs.usgs.gov/sir/2013/5108/pdf/sir2013-5108.pdf

Supplementary Table 6. Hydrostratigraphy details for the Denver Basin

Formation name	Category	Quote
Denver Formation	Clastic sedimentary rock **aquifer** (consolidated or semi-consolidated rock)	"Alluvial fan, swamp, overbank deposits; andesitic fluvial sandstone with volcanic ash deposits, coal, lignite, mudstone/claystone; Fe-rich sediments; sediments source of Se and U to groundwater." (Musgrove et al., 2014) "Confined to unconfined aquifer" (Musgrove et al., 2014). "Confined in central part. Contains a water table only near out-crops. Moderately permeable. May yield as much as 200 gallons per minute." (Robson & Banta, 1995)
Arapahoe Formation	Clastic sedimentary rock **aquifer** (consolidated or semi-consolidated rock)	"Fluvial environment, alluvial fan deposits near mountain front; conglomerates, sandstone, siltstone, shale; pebbles and cobbles with granite, chert, metamorphic rocks, and quartize; shale more prevalent in northern part of basin." (Musgrove et al., 2014). "Productive confined aquifer"- (Musgrove et al., 2014).
Laramie Formation	Sedimentary rock **aquitard** (consolidated or semi-consolidated rock)	"Upper part shale, silty shale, silt stone, and interbedded fine sandstone." (Robson & Banta, 1995). "Shale is impermeable". (Robson & Banta, 1995). See figure 83 from Ref (Robson & Banta, 1995)
Laramie-Fox Hill Formation	Clastic sedimentary rock **aquifer** (consolidated or semi-consolidated rock)	"Laramie Formation: swamps, deltas, overbank deposits; claystone, coal, fluvial channel sandstone; contains coal and lignite beds. Fox Hills Sandstone: marine beach and delta-front environment; sandstone, thin siltstone and claystone beds; contains marine fossils." (Musgrove et al., 2014) "Productive confined to unconfined aquifer" – (Musgrove et al., 2014)
Pierre shale	Sedimentary rock **aquitard** (consolidated or semi-consolidated rock)	"A thick unit of low-permeability Cretaceous age Pierre Shale underlies the Laramie-Fox Hills aquifer and forms the base of the aquifer system" – (Musgrove et al., 2014)

Musgrove, M., Beck, J. A., Paschke, S. S., Bauch, N. J., Mashburn, S. L. (2014). Quality of Groundwater in the Denver Basin Aquifer System, Colorado, 2003-5. US Geological Survey Scientific Investigations Report 2014-5051, 123 pp. Accessed February 21, 2022 from https://pubs.usgs.gov/sir/2014/5051/pdf/sir2014-5051.pdf

Robson, S. G., & Banta, E. R. (1995). Ground water atlas of the United States: Segment 2, Arizona, Colorado, New Mexico, Utah. US Geological Survey Hydrologic Investigations Atlas 730-C, Segment 2, 34 pp. Accessed February 21, 2022 from https://pubs.usgs.gov/ha/730c/report.pdf

Supplementary Table 7. Hydrostratigraphy details for the Western Carrizo-Wilcox Aquifer System

Formation name	Category	Quote
Younger sediments	Clastic sedimentary rock **aquifer** (consolidated or semi-consolidated rock) *(may be unconsolidated in places)*	"Sand, silt, clay, and some gravel." (Sandeen 1987) (Table 6). "May yield small quantities of water to shallow dug wells." (Sandeen 1987)
Sparta Sand	Clastic sedimentary rock **aquifer** (consolidated or semi-consolidated rock) *(may be unconsolidated in places)*	"Interbedded sand, clay, and silt." (Sandeen 1987) (Table 6). "Feeds springs; may yield some water to dug wells." (Sandeen 1987)
Weches Formation	Sedimentary rock **aquitard** (consolidated or semi-consolidated rock)	"Glauconite, glauconitic clay and sand. Secondary deposits of limestone in outcrop." (Sandeen 1987) (Table 6). Not known to yield water to wells in Rusk County. (Sandeen 1987)
Queen City Sand	Clastic sedimentary rock **aquifer** (consolidated or semi-consolidated rock)	"Sand, silt, clay, and some lignite." (Sandeen 1987) (Table 6). "Yields small to moderate quantities of freshwater. (Sandeen 1987) "Water is stored in the sand, loosely cemented sandstone, and interbedded clay layers of the Queen City Formation that reaches 2,000 feet in thickness in South Texas." (George et al. 2011)
Reklaw Formation	Sedimentary rock **aquitard** (consolidated or semi-consolidated rock)	"Glauconitic clay, some sand, weathers to a red clayey soil, limonite seams, iron concretions." (Sandeen 1987) (Table 6). "Yields small quantities of water to wells." (Sandeen 1987) "The Carrizo-Wilcox aquifer is separated from the overlying Queen City aquifer by the Reklaw or Bigford Fm., which is a confining unit (Fig. 2)." (Huang et al. 2012)
Carrizo Sand	Clastic sedimentary rock **aquifer** (consolidated or semi-consolidated rock)	"Grey to white. Often massive sand, clay lenses; may be predominantly clayey". (Sandeen 1987) (Table 6). "Yields large to moderate quantities of freshwater. In hydrologic continuity with the Wilcox." (Sandeen 1987)
Wilcox Group	Clastic sedimentary rock **aquifer** (consolidated or semi-consolidated rock)	"Thin, sometimes massive beds of sand; clay and lignite. Beds often dis- continuous." (Sandeen 1987) (Table 6). "Yields large to moderate quantities of freshwater. In hydrologic continuity with the Mil cox." (Sandeen 1987)
Midway marine clay	Sedimentary rock **aquitard** (consolidated or semi-consolidated rock)	"Calcareous clay and minor amounts of limestone, silt, and glauconitic clay." (Sandeen 1987) (Table 6). "Not known to yield water to wells in Rusk County; upper sand may contain some slightly saline water." (Sandeen 1987)

Sandeen, W. M. (1984). Ground-water resources of Rusk County, Texas. US Geological Survey Open-File Report 83-757, 116 pp. Accessed February 21, 2022 from https://pubs.usgs.gov/of/1983/0757/report.pdf

George, P.G., Mace, R.E., Petrossian, R. (2011). Aquifers of Texas. Texas Water Development Board Report 380, 182 pp. Accessed February 21, 2022 from https://www.twdb.texas.gov/publications/reports/numbered_reports/doc/R380_AquifersofTexas.pdf

Huang, Y., Scanlon, B. R., Nicot, J. P., Reedy, R. C., Dutton, A. R., Kelley, V. A., Deeds, N. E. (2012). Sources of groundwater pumpage in a layered aquifer system in the Upper Gulf Coastal Plain, USA. Hydrogeology Journal, 20, 783-796.

Supplementary Table 8. Hydrostratigraphy details for the Dakota Aquifer System

Formation name	Category	Quote
Pleistocene deposit	Unconsolidated aquifer	"Glacial aquifers in the model area consist primarily of outwash deposits composed of sorted gravel, sand, and silt." (Marini et al., 2012)
Cretaceous shale confining layer	Sedimentary rock **aquitard** (consolidated or semi-consolidated rock)	"The confining layer overlying the Dakota, which we call the "Cretaceous shale confining layer," includes the entire sequence above the Dakota-Newcastle Sand- stone. Sedimentary rocks from this dominantly shale sequence crop out over much of South Dakota." (Bredehoeft et al., 1983) | "Low permeability confining layers separate the major aquifers" (Konikow, & Neuzil, 2007).
Newcastle Sandstone	Clastic sedimentary rock **aquifer** (consolidated or semi-consolidated rock)	"As noted above, Gries (1958) indicates that the Newcastle Sandstone is quite thin and may be dis- continuous in western South Dakota. However, subsurface correlations suggest that the sandstones tend to be continuous" (Bredehoeft et al., 1983) | "The Inyan Kara, Newcastle, and Dakota Sandstones were treated as a single continuous unit forming the Dakota Aquifer" (Konikow, & Neuzil, 2007).
Skull Creek Shale	Sedimentary rock **aquitard** (consolidated or semi-consolidated rock)	"we considered the overlying Cretaceous shale and the underlying Skull Creek Shale and Madison confining units." (Konikow, & Neuzil, 2007).
Inyan Kara Group	Clastic sedimentary rock **aquifer** (consolidated or semi-consolidated rock)	"The Inyan Kara, Newcastle, and Dakota Sandstones were treated as a single continuous unit forming the Dakota Aquifer" (Konikow, & Neuzil, 2007).
Dakota Sandstone	Clastic sedimentary rock **aquifer** (consolidated or semi-consolidated rock)	"The Inyan Kara, Newcastle, and Dakota Sandstones were treated as a single continuous unit forming the Dakota Aquifer" (Konikow, & Neuzil, 2007).
Madison confining layer	Sedimentary rock **aquitard** (consolidated or semi-consolidated rock)	"Low permeability confining layers separate the major aquifers" (Konikow, & Neuzil, 2007).
Madison Group	Carbonate aquifer	"Madison Limestone, an important carbonate aquifer" (Konikow, & Neuzil, 2007).
Undifferentiated Paleozoic rocks	Sedimentary rock **aquitard** (consolidated or semi-consolidated rock)	"We have chosen to treat the Paleozoic rocks beneath the Madison as impermeable in our models, thus no flow in these units is represented in figure 16" (Bredehoeft et al., 1983)
Precambrian Crystalline rocks	Endogenous bedrock	"The interbedded layering of the mudstone and conglomerates in the Sioux Quartzite are so thoroughly lithified that the pore spaces are almost entirely filled with cement, causing the Sioux Quartzite to be nearly impermeable". (Davis et al., 2019)

Bredehoeft, J. D., C. E. Neuzil, and P. C. D. Milly (1983), Regional flow in the Dakota aquifer: A study of the role of confining layers, U.S. Geological Survey Water Supply Paper 2237, 45 pp. Accessed February 21, 2022 from https://pubs.usgs.gov/wsp/2237/report.pdf

Konikow, L. F., & Neuzil, C. E. (2007). A method to estimate groundwater depletion from confining layers. Water Resources Research, 43.

Marini, K. A., Hoogestraat, G. K., Aurand, K. R., & Putnam, L. D. (2012). *Conceptual and numerical models of the glacial aquifer system north of Aberdeen, South Dakota*. US Geological Survey.

Davis, K. W., Eldridge, W. G., Valder, J. F., & Valseth, K. J. (2019). Groundwater-flow model and analysis of groundwater and surface-water interactions for the Big Sioux aquifer, Sioux Falls, South Dakota. Scientific Investigations Report 2019-5117, 102 pp. Accessed February 21, 2022 from https://pubs.usgs.gov/sir/2019/5117/sir20195117.pdf

Supplementary Table 9. Hydrostratigraphy details for the Floridan Aquifer System

Formation name	Category	Quote
Surficial aquifer	Unconsolidated aquifer	"The surficial aquifer system consists mostly of sand and locally contains gravel and sandy lime stone of Pliocene to Holocene age. Where these sediments are thick and highly permeable," (Williams, and Kuniansky, 2016) | "The surficial aquifer system forms a thin irregular blanket of terrace and alluvial sands that can act as an important source sink layer for temporary storage of groundwater that may ultimately recharge the underlying Floridan aquifer system. (Williams, and Kuniansky, 2016)
Upper confining unit	Sedimentary rock **aquitard** (consolidated or semi-consolidated rock)	"The upper confining unit includes all low-permeability late and middle Miocene beds, where present, and locally includes low-permeability post-Miocene beds (Miller, 1986). The generalized thickness and extent of the upper confining unit is shown in figure 21 and a more detailed version of this map is presented on plate 3. Interbedded, locally phosphatic, sand, silt, and clay are the predominant clastic components of the upper confining unit. Locally, lower permeability early Miocene carbonate rocks are included in the upper confining unit. A residuum of limestone may locally form a semi confining layer in the outcrop areas of the Upper Floridan aquifer," (Williams, and Kuniansky, 2016)
Lower Brunswick aquifer	Carbonate aquifer	"Clarke and others (1990) identified the lower Brunswick aquifer entirely within Miocene sediments" (Williams, and Kuniansky, 2016) | "Locally the lower Brunswick is a carbonate aquifer." (Williams, and Kuniansky, 2016)
Upper Floridan aquifer	Carbonate aquifer	"The Upper Floridan aquifer includes the uppermost or shallowest permeable zones in the Floridan aquifer system." (Williams, and Kuniansky, 2016) | "The Floridan aquifer system includes the vertically continuous carbonate-rock system described by Miller (1986)". (Williams, and Kuniansky, 2016) | " the carbonate rocks of the Upper Floridan aquifer may directly overlie clastic rocks of the Lisbon and Claiborne aquifers;" (Williams, and Kuniansky, 2016)
Middle confining unit	Sedimentary rock **aquitard** (consolidated or semi-consolidated rock)	"The middle confining units of Miller (1986) were a series of numbered discontinuous lower permeability units in the approximate middle part of the aquifer system." (Williams, and Kuniansky, 2016). | "In correlation logs used by Miller (1986), this highly resistive low porosity unit is mapped as middle confining unit MCUI, consisting of hard dolomitic limestone and dolostone and is identified by Johnson (1984)" (Williams, and Kuniansky, 2016).
Lower Floridan aquifer	Carbonate aquifer	"The Floridan aquifer system is divided into the Upper and Lower Floridan aquifers." (Williams, and Kuniansky, 2016). | "The base of the Lower Floridan aquifer in the northern coastal region generally is marked by low permeability limestone and marl in the lower part of the Avon Park Formation". (Williams, and Kuniansky, 2016). | "Because the Floridan aquifer system largely is composed of a vertical sequence of carbonate rocks" (Williams, and Kuniansky, 2016).
Base of Floridan aquifer	Sedimentary rock **aquitard** (consolidated or semi-consolidated rock)	"The base of the Floridan aquifer system is marked by the lower confining unit, consisting of predominantly low permeability late Paleocene to middle Eocene rocks." (Williams, and Kuniansky, 2016). | In the panhandle of Florida, the base of the system is marked by lower to middle Eocene rocks. In that area, the rocks grade from sandy limestone in the eastern panhandle to argillaceous limestone, sandy limestone, and clay in the western panhandle where they become part of the lower confining unit. (Williams, and Kuniansky, 2016).

Williams, L.J., and Kuniansky, E.L., 2016, Revised hydrogeologic framework of the Floridan aquifer system in Florida and parts of Georgia, Alabama, and South Carolina. U.S. Geological Survey Professional Paper 1807, 140 p., 23 pls., http://dx.doi.org/10.3133/pp1807.

Miller, J. A. (1986). Hydrogeologic framework of the Floridan aquifer system in Florida and parts of Georgia, Alabama, and South Carolina. Department of the Interior, US Geological Survey Professional Paper 1403-B, 101 pp. Accessed February 21, 2022 from https://pubs.usgs.gov/pp/1403b/report.pdf

Supplementary Table 10. Hydrostratigraphy details for the North Atlantic Coastal Plain

Formation name	Category	Quote
Surficial	Unconsolidated aquifer	"The Surficial aquifer is the uppermost aquifer in the NACP aquifer system." (Masterson et al., 2013) | "Across most of the New Jersey Coastal Plain, permeable surficial sediments of Pleistocene to Holocene age that are hydraulically continuous with older, underlying sediments." (Masterson et al., 2013)
Calvert aquifer system	Clastic sedimentary rock **aquifer** (consolidated or semi-consolidated rock)	"The Lower Chesapeake regional aquifer includes the Lower Kirkwood-Cohansey aquifer system in New Jersey, the Milford, Frederica, Federalsburg, and Cheswold local aquifers in Delaware, the Choptank and Calvert local aquifers in Maryland, the Saint Marys aquifer in Virginia, and the Pungo River aquifer in North Carolina." (Masterson et al., 2013)
Calvert confining unit	Sedimentary rock **aquitard** (consolidated or semi-consolidated rock)	"It consists primarily of marine clay and sandy clay of Miocene age, and its thickness increases northward." (Masterson et al., 2013)
Piney Point aquifer	Clastic sedimentary rock **aquifer** (consolidated or semi-consolidated rock)	"The Piney Point aquifer consists of marine sediments of mostly Eocene to Oligocene age, though it also may include sediments of Miocene age in some locations" (Masterson et al., 2013) | "a productive section of the Piney Point aquifer north of the James River and south of the Potomac River in Virginia composes calcite-cemented sands and moldic limestone." (Masterson et al., 2013)
Nanjemoy-Marlboro confining unit	Sedimentary rock **aquitard** (consolidated or semi-consolidated rock)	"The regional confining unit includes the Vincentown Manasquan confining unit in New Jersey; the Nanjemoy-Marlboro Clay confining unit in Delaware, Maryland, and Virginia; and the Beaufort confining unit in North Carolina." (Masterson et al., 2013) | "The confining unit overlaying the Aquia aquifer is made of marine silt, clay, and sandy clay ranging from a thickness of 50 ft in North Carolina to more than 900 ft in New Jersey (Trapp, 1992)." (Masterson et al., 2013)
Aquia aquifer	Clastic sedimentary rock **aquifer** (consolidated or semi-consolidated rock)	"The Aquia aquifer composes permeable marine sediments of Paleocene age and consists primarily of medium- to coarse grained glauconitic and fossiliferous quartz sands." (Masterson et al., 2013)
Severn confining unit	Sedimentary rock **aquitard** (consolidated or semi-consolidated rock)	"The northern section of the Monmouth-Mount Laurel regional aquifer is separated from the Aquia regional aquifer above by an overlying confining unit that includes the Navesink-Hornerstown confining unit in New Jersey and the Severn confining unit in Delaware and Maryland. The confining unit consists of marine silt, clay, and silty and clayey glauconitic sand of primarily Cretaceous age." (Masterson et al., 2013)
Magothy aquifer	Clastic sedimentary rock **aquifer** (consolidated or semi-consolidated rock)	"The Magothy aquifer composes primarily sandy parts of the Magothy Formation, which were deposited in a transitional fluvial-marine environment during the Late Cretaceous" (Masterson et al., 2013)
Magothy-Patapsco confining unit	Sedimentary rock **aquitard** (consolidated or semi-consolidated rock)	"This unit is referred to as the Raritan Clay confining unit in New York and New Jersey, the Magothy-Patapsco confining unit in Delaware and Maryland." (Masterson et al., 2013)
Upper Patapsco aquifer	Clastic sedimentary rock **aquifer** (consolidated or semi-consolidated rock)	"The top of the Potomac aquifer in Virginia now correlates with the top of the Upper Patapsco aquifer in Maryland" (Masterson et al., 2013)
Confining unit	Sedimentary rock **aquitard** (consolidated or semi-consolidated rock)	"The Potomac-Patapsco regional aquifer consists of two local aquifers, the Upper Patapsco and Lower Patapsco, separated by an intervening confining unit." (Masterson et al., 2013)
Lower Patapsco aquifer	Clastic sedimentary rock **aquifer** (consolidated or semi-consolidated rock)	"It is composed of fluvial-deltaic sediments of primarily Early Cretaceous age in Maryland and Delaware, and Late Cretaceous age in New Jersey and New York." (Masterson et al., 2013)
Confining unit	Sedimentary rock **aquitard** (consolidated or semi-consolidated rock)	"The Potomac-Patuxent aquifer (where present) is overlain by a confining unit separating this aquifer from the overlying Potomac-Patapsco aquifer. This regional confining unit, called the Potomac confining unit in the RASA study and known as the Arundel Clay confining unit in Maryland, composes hard clays and silts within the fluvial-deltaic Potomac and Raritan formations in New Jersey, Delaware, Maryland, and northern Virginia." (Masterson et al., 2013)
Patuxent aquifer	Clastic sedimentary rock **aquifer** (consolidated or semi-consolidated rock)	"The Potomac-Patuxent **aquifer** is the lowermost (depth) regional aquifer of the NACP." (Masterson et al., 2013) | "The Potomac-Patuxent aquifer is primarily of Early Cretaceous age and of fluvial-deltaic origin. It consists primarily of lenses of medium- to coarse-grained quartz sand with some gravel interbedded with lenses of clay and silt." (Masterson et al., 2013)
Bedrock	Endogenous bedrock	"The NACP is underlain by a wedge of unconsolidated to partially consolidated sediments that range in age from Early Cretaceous to Holocene (fig. 6). These sediments unconformably overlie a basement of Precambrian to Paleozoic-age consolidated bedrock." (Masterson et al., 2013) "The basement beneath the Virginia Coastal Plain is composed primarily of igneous and metamorphic rocks as old as 2,500 m.y. (Paleozoic to Proterozoic ages; Powars and Bruce, 1999)." (MacFarland and Bruce, 2006)

Masterson, J. P., Pope, J. P., Monti Jr, J., Nardi, M. R., Finkelstein, J. S., & McCoy, K. J. (2013). Hydrogeology and hydrologic conditions of the Northern Atlantic Coastal Plain aquifer system from Long Island, New York, to North Carolina (No. 2013-5133). US Geological Survey.

McFarland, E.R., Bruce, T.S. (2006). The Virginia Coastal Plain Hydrogeologic Framework: U.S. Geological Survey Professional Paper 1731, 118 p., 25 pls Accessed February 21, 2022 from https://pubs.usgs.gov/pp/2006/1731/PP1731.pdf

Supplementary Table 11. Hydrostratigraphy details for the Milk River Aquifer System

Formation name	Category	Quote
Bearpaw Formation & surficial sediments	Sedimentary rock **aquitard** (consolidated or semi-consolidated rock) *(may be unconsolidated in places)*	"As the glacial drift consists mainly of low permeability till, the surficial deposits in the study area generally do not constitute productive aquifers." (Pétré et al. (2016)
Belly River Group/ Judith River formation	Clastic sedimentary rock **aquifer** (consolidated or semi-consolidated rock)	"The Belly River Group/Judith River Formation constitutes an aquifer with a hydraulic conductivity ranging from 9 × 10−8 to 8.8 × 10−7 m/s". Pétré et al. (2015) | "The dark shale, sandstone, and coal seams of the Foremost Formation are overlain by massive yellow and grey sandstone of the Oldman Formation and thick sandstones and siltstones of the Dinosaur Park Formation." (Pétré et al., 2015)
Claggett Shale/Pakowki Formation	Sedimentary rock **aquitard** (consolidated or semi-consolidated rock)	"The Milk River Formation is overlain by a thick unit of marine shales, the Pakowki Formation (Claggett Shale equivalent in Montana), which is a 130- m-thick aquitard." (Pétré et al., 2015)
Upper Alderson Sands of the Alderson Member	Clastic sedimentary rock **aquifer** (consolidated or semi-consolidated rock)	"The overlapping area of the Alderson Member corresponds to the Upper Alderson Sands. This part is still water bearing." (Pétré et al., 2015) | "The Upper Alderson Sands, which form small lobate sand bodies." (Pétré et al., 2015)
Deadhorse Coulee Member	Sedimentary rock **aquitard** (consolidated or semi-consolidated rock)	"The Milk River Formation (Eagle Sandstone in Montana) is a regressive clastic wedge deposited during the Late Cretaceous." (Pétré et al., 2015) | "The Milk River Formation has been traditionally subdivided into three members: the basal Telegraph Creek Member, the middle Virgelle Member, and the upper Deadhorse Coulee Member." (Pétré et al., 2015) | "It is a nonmarine unit deposited in the coastal plain environments landward of the Virgelle shore-faces". (Pétré et al., 2015) | "This well-defined unit consists predominantly of interbedded shale, siltstone, and fine-grained sandstone with coal seams." (Pétré et al., 2015).<br>"The Deadhorse Coulee Member represents the upper part of the Milk River Formation as named by Tovell (1956). It is a nonmarine unit deposited in the coastal plain environments landward of the Virgelle shore-faces (O'Connell 2014)" (Pétré et al., 2016). | "The upper member of the Milk River Formation is the low-permeability Deadhorse Coulee Member. This well-defined unit consists predominantly of interbedded shale, siltstone, and fine-grained sandstone with coal seams (Payenberg 2002b)." (Pétré et al., 2016).
Virgelle Member Milk (Milk River Aquifer)	Clastic sedimentary rock **aquifer** (consolidated or semi-consolidated rock)	"The Virgelle Member gradationally overlies the Telegraph Creek Member (Meijer-Drees and Myhr 1981). It consists of grey to buff, thick bedded, fine- to medium-grained sandstone with thinly bedded siltstone (Tuck 1993)." (Pétré et al., 2015) | "The middle Virgelle Member is the most important aquifer within the formation." (Pétré et al., 2015) | "The Telegraph Creek Member is a transitional unit between the shale of the Colorado Group and the massive sandstone of the Virgelle Member of the Milk River Formation. It is interpreted as deposits of an offshore to shore-face transition (Payenberg 2002a)." (Pétré et al., 2015)
Telegraph Creek Member	Clastic sedimentary rock **aquifer** (consolidated or semi-consolidated rock)	"The Telegraph Creek Member is a transitional unit between the shale of the Colorado Group and the massive sandstone of the Virgelle Member of the Milk River Formation." (Pétré et al., 2015) | "The Telegraph Creek Member consists of thinly interbedded sandy shale, siltstone, and fine-grained shaly sandstone." (Pétré et al., 2015)
Colorado Group	Sedimentary rock **aquitard** (consolidated or semi-consolidated rock)	"It was deposited during marine conditions in a moderate-depth sea environment. The Colorado Group consists mainly of dark grey to black bentonitic marine shale." (Pétré et al., 2015) | "discharge or outflow may occur through vertical leakage along the buried valleys and through the underlying aquitards of the Colorado Group as shown by the vertical hydraulic gradient (Fig. 8)." (Pétré et al., 2015)
Bow Island Sandstone	Clastic sedimentary rock **aquifer** (consolidated or semi-consolidated rock)	"Colorado Group underlies the geological units in the study area. It is a 500-m thick regional aquitard and contains several thin sandstone beds, the most significant being the Bow Island Sandstone." (Pétré et al., 2015). I "By comparing the hydraulic heads in the surficial aquifer, the MRA and the Bow Island sandstone on a cross-section, Phillips et al. (1986) concluded that the water leaves the aquifer via vertical leakage." (Pétré et al., 2015).

Pétré, M. A., Rivera, A., & Lefebvre, R. (2015). Three-dimensional unified geological model of the milk river transboundary aquifer (Alberta, Canada–Montana, USA). Canadian Journal of Earth Sciences, 52(2), 96-111.

Pétré, M. A., Rivera, A., Lefebvre, R., Hendry, M. J., & Folnagy, A. J. (2016). A unified hydrogeological conceptual model of the Milk River transboundary aquifer, traversing Alberta (Canada) and Montana (USA). Hydrogeology Journal, 24(7), 1847-1871.

Supplementary Table 12. Hydrostratigraphy details for the Black Warrior River Aquifer System

Formation name	Category	Quote
Midway Group	Sedimentary rock **aquitard** (consolidated or semi-consolidated rock)	"South and west of the Ripley Cuesta is another belt of lowlands, the Flatwoods, underlain by the Paleocene Porters Creek Clay of the Midway Group." Mallory (1993) | "The Midway Group is composed predominantly of marine clay and shale but includes subordinate sand and limestone beds." Mallory (1993)
Ripley aquifer (part of Selma Group)	Clastic sedimentary rock **aquifer** (consolidated or semi-consolidated rock)	"An important aquifer in Tippah, eastern Benton, and northern Union Counties. The McNoiry sand member ranges up to 250 feet thick and is the unit which most of the wells utilize in the Ripley. Quality is good except for hardness." (Shows, 1970)
Demopolis Chalk and Mooreville Chalk (part of Selma Group; *"Coffee sand" formation may be an aquifer in areas)*	Sedimentary rock **aquitard** (consolidated or semi-consolidated rock)	"These formations in western Alabama and eastern Mississippi, in ascending order, are the Mooreville Chalk, the Demopolis Chalk, the Ripley Formation, and the Prairie Bluff Chalk." Cushing et al., (1964) "Not an aquifer." (referring to Demopolis Chalk and Mooreville chalk) (Shows, 1970)
Tombigbee Sand Member	Clastic sedimentary rock **aquifer** (consolidated or semi-consolidated rock)	"A persistent sand at the top of the formation, known as the Tombigbee Sand Member (Hilgard, 1860, p. 61), is massive, highly glauconitic, calcareous, and fossiliferous in the upper part." Cushing et al., (1964)
Eutaw Formation	Clastic sedimentary rock **aquifer** (consolidated or semi-consolidated rock)	"The main body of the formation is composed of gray clay interbedded with fine glauconitic sand. Thin beds of fine to medium glauconitic sand are common and are fairly persistent near the base of the formation, which is normally marked by a thin bed of fine gravel. The sands are commonly cross bedded or show distinct stratification." Cushing et al., (1964) | "Thin beds of fine to medium glauconitic sand within the Eutaw and McShan Formations make up the bulk of the upper Black Warrior River regional aquifer, locally known as the Eutaw-McShan aquifer." Mallory (1993)
McShan Formation	Clastic sedimentary rock **aquifer** (consolidated or semi-consolidated rock)	"It consists of laminated micaceous glauconitic gray clay, fine sand, and lenticular beds of fine to medium glauconitic sand." Cushing et al., (1964) | "Thin beds of fine to medium glauconitic sand within the Eutaw and McShan Formations make up the bulk of the upper Black Warrior River regional aquifer, locally know as the Eutaw-McShan aquifer." Mallory (1993)
Gordo Formation	Clastic sedimentary rock **aquifer** (consolidated or semi-consolidated rock)	"It is composed of thick beds of sand containing gravel in the lower part and multicolored clay and shale interbedded with sand in the upper part." Cushing et al., (1964)<br>"The Gordo Formation is an important aquifer in Alabama and Mississippi." Cushing et al., (1964)
Coker Formation	Clastic sedimentary rock **aquifer** (consolidated or semi-consolidated rock)	"In Alabama, the Coker has been subdivided into the Eoline Member and an upper unnamed member." Cushing et al., (1964)<br><br>"The Eoline Member (Monroe and others, 1946, p. 194-197) consists of thin-bedded clay, sandy clay, shale, and sand, mostly of marine origin; subordinate beds of sand occur throughout the unit." Cushing et al., (1964) | "Permeable sand and gravel beds in the Coker and Gordo Formations of the Tuscaloosa Group make up the lower Black Warrior River aquifer." Mallory (1993)
Massive sand	Clastic sedimentary rock **aquifer** (consolidated or semi-consolidated rock)	"The massive sand is "a series of medium- to coarse grained sands * * * " (according to McGlothlin, 1944, p. 40). Interbedded shale and clay occur in the thick beds of coarse sand, chert, and quartz gravel which compose the main body of the unit." Cushing et al., (1964) | "Although the massive sand is not generally used as a source of ground water, it is potentially one of the most important aquifers in the embayment." Mallory (1993)
Lower Cretaceous rocks	Clastic sedimentary rock **aquifer** (consolidated or semi-consolidated rock)	"Lower Cretaceous rocks do not crop out on the eastern side of the embayment. However, in Mississippi and Alabama they occur in the subsurface as thick sands, clays, and shales." Cushing et al., (1964) | "In places, some of the uppermost beds of the Lower Cretaceous series are lithologically very similar to the basal beds of the over- lying Coker Formation and are included in this aquifer."
Paleozoic rocks	Sedimentary rock **aquitard** (consolidated or semi-consolidated rock)	"The lower Black Warrior River aquifer is the lowest aquifer in the Southeastern Coastal Plain aquifer system. In the northern part of the study area, the basal confining unit of the Southeastern Coastal Plain aquifer system consists of Paleozoic rocks. These consolidated shales, sandstones, limestones, and dolomites have much smaller permeability than the overlying Cretaceous sediments." Mallory (1993)

Cushing, E. M., Boswell, E. H., & Hosman, R. L. (1964). General geology of the Mississippi Embayment. US Geological Survey Professional Paper 448-B, 32 pp. Accessed February 21, 2022 from https://pubs.usgs.gov/pp/0448b/report.pdf

Mallory, M. J. (1993). Hydrogeology of the southeastern coastal plain aquifer system in parts of eastern Mississippi and western Alabama. Regional aquifer-system analysis. Southeastern coastal plain. US Geological Survey Professional Paper 1410-G, 66 pp. Accessed February 21, 2022 from https://pubs.usgs.gov/pp/1410g/report.pdf

Shows, T.N. (1970). Water resources of Mississippi. Mississippi Geological, Economic and Topographical Survey Bulletin 113, 182 pp. Accessed February 21, 2022 from https://www.mdeq.ms.gov/wp-content/uploads/2017/06/Bulletin-113.pdf

Supplementary Table 13. Hydrostratigraphy details for the Mojave Basin

Formation name	Category	Quote
Undifferentiated alluvium	Unconsolidated aquifer	“The undifferentiated alluvium (QTa), which forms the regional aquifer, is more than 1000 m thick at some locations (Subsurface Surveys, 1990), and consists of alluvial and basin-fill deposits.” Kulongoski et al., (2003)
Igneous and metamorphic basement complex	Endogenous bedrock	“The pre-Tertiary basement complex typically has low porosity and permeability, yielding only small quantities of water to wells; however, where the basement complex is intensely fractured, as along major faults, the bedrock is more permeable.” Stamos et al., (2001)

Kulongoski, J. T., Hilton, D. R., & Izbicki, J. A. (2003). Helium isotope studies in the Mojave Desert, California: implications for groundwater chronology and regional seismicity. Chemical Geology, 202(1-2), 95-113.

Stamos, C. L., Martin, P., Nishikawa, T., & Cox, B. F. (2001). Simulation of ground-water flow in the Mojave River Basin, California. US Geological Survey Water-Resources Investigations Report, 01-4002.

Supplementary Table 14. Hydrostratigraphy details for the California Central Valley (northern)

Formation name	Category	Quote
Alluvial Fan Deposit	Unconsolidated aquifer	"The alluvial fan is highly suitable for banking purposes, as it generally consists of permeable river deposits with high well yields that allow quick recovery." (EWA, 2003)
Tehama Formation	Unconsolidated aquifer	"The Tehama Formation in the western portion of the basin is derived from Coast Range sediment. In most of the Sacramento Groundwater Basin, the Tuscan, Mehrten, and Tehama formations are overlain with relatively thin alluvial deposits." (EWA, 2003)<br><br>"The groundwater basin west of the Sacramento River is composed of the Tehama Formation, which has exhibited subsidence in Yolo County" (EWA, 2003)<br><br>"Of particular importance to this study is the Plio-Pleistocene Tehama Formation, a productive aquifer described in some detail below…" Davisson and Criss (1993)<br><br>"In the subsurface the Tehama deposits generally are water-saturated and unconsolidated, except near the base of the section where moderately consolidated gravels occur" Davisson and Criss (1993)<br><br>"The Tehama Formation is a 600-900 m thick fluvial deposit that extends from the Coast Ranges to the axis of the Sacramento Valley, and from Red Bluff in the north to the Montezuma Hills in the eastern Delta region (Olmsted and Davis, 1961). This formation, which has been locally deformed by late Cenozoic uplift and tilting along the western basin margin, consists of detritus derived from the rapidly rising Coast Ranges (Thomasson et al., 1960; Loewen et al., 1992). The deposits are characterized by yellowish or brownish to blue-green clays interbedded with sands and gravels." Davisson and Criss (1993)
Eocene and Post-Eocene Continental Deposit	Clastic sedimentary rock **aquifer** (consolidated or semi-consolidated rock)	"The base of the post-Eocene continental deposits is equivalent to the base of the Tehama Formation of Pliocene age which in some places at least may be of late Oligocene and early Miocene age. The post-Eocene deposits contain most of the fresh ground water in the valley." Page, (1974).<br><br>"The continental sediments consist mostly of sand and gravel interbedded and mixed with clay and silt deposited by streams and lakes." Thiros et al. (2010)
Granitic and Metamorphic rocks of the Sierra Nevada	Endogenous bedrock	"Granitic, volcanic, and metamorphic rocks that crop out and underlie the eastern part of the valley form an almost impermeable boundary for the basin-fill groundwater system." (EWA, 2003)

Page, R. W. (1974). Base and thickness of the post-Eocene continental deposits in the Sacramento Valley, California (No. 45-73). US Geological Survey. (https://pubs.usgs.gov/wri/1973/0045/report.pdf)

Environmental Water Account ("EWA") (2003). Draft Environmental Impact Statement Environmental Impact Report Volume I Chapters 1-9. (https://www.usbr.gov/mp/ewa/docs/v1-draft-enviro-impact-statement-environmental-impact-report.pdf)

Thiros, S. A., Bexfield, L. M., Anning, D. W., & Huntington, J. M. (2010). Conceptual understanding and groundwater quality of selected basin-fill aquifers in the southwestern United States (No. 1781). US Geological Survey. (https://pubs.usgs.gov/pp/1781/)

Marchand, D. E., & Allwardt, A. (1977). Late Cenozoic stratigraphic units, northeastern San Joaquin Valley, California (No. 77-748). US Geological Survey. (https://pubs.usgs.gov/bul/1470/report.pdf)

Davisson, M. L., & Criss, R. E. (1993). Stable isotope imaging of a dynamic groundwater system in the southwestern Sacramento Valley, California, USA. Journal of Hydrology, 144(1-4), 213-246.

Davisson, M. L., & Criss, R. E. (1993). Stable isotope imaging of a dynamic groundwater system in the southwestern Sacramento Valley, California, USA. Journal of Hydrology, 144(1-4), 213-246.

Supplementary Table 15. Hydrostratigraphy details for the Mississippi Embayment Aquifer System

Formation name	Category	Quote
Mississippi River Valley alluvial aquifer	Unconsolidated aquifer	The alluvial deposits that make up the aquifer are mostly of Holocene age." (Hosman, & Weiss, (1991) | "The materials constituting the aquifer range in size from coarse gravel to clay. They commonly grade downward from fine sand, silt, and clay at the top to coarse sand or gravel at the base." (Hosman, & Weiss, (1991)
Upper Claiborne aquifer	Clastic sedimentary rock **aquifer** (consolidated or semi-consolidated rock)	"The aquifer consists of interbedded fine sand, silt, and clay with common occurrences of lignite." (Hosman, & Weiss, (1991)
Cook Mountain Formation	Sedimentary rock **aquitard** (consolidated or semi-consolidated rock)	"The clay, which is mostly the Cook Mountain Formation (included in the Laredo Formation in part of southern Texas), underlies about 92,000 mi^2 of the area." (Hosman, & Weiss, (1991) | "the Cook Mountain generally constitutes a confining bed" (Hosman, & Weiss, (1991)
Middle Claiborne aquifer (Sparta Sand)	Clastic sedimentary rock **aquifer** (consolidated or semi-consolidated rock)	"This aquifer underlies about 136,000 mi^2 and consists primarily of the Sparta Sand, which is present in most of the study area as a continentally derived sand with clay interbeds of varying thickness and extent." (Hosman, & Weiss, (1991)
Cane River Formation	Sedimentary rock **aquitard** (consolidated or semi-consolidated rock)	"In Texas, three formations are equivalent to the Cane River Formation. They are, in ascending order, the Reklaw Formation, the Queen City Sand, and the Weches Formation (except in extreme southern Texas, where the Bigford Formation and El Pico Clay represent these units). The Reklaw Formation, which is mostly clay, is virtually the entire lower Claiborne confining unit east of the Sabine uplift." (Hosman, & Weiss, (1991)
Lower Claiborne-Upper Wilcox aquifer	Clastic sedimentary rock **aquifer** (consolidated or semi-consolidated rock)	"Most of the aquifer is the Carrizo Sand and its equivalent, the Meridian Sand Member of the Tallahatta Formation (table 1). The Carrizo or Meridian is an extensive sand, commonly massive and unbroken by clay beds, that represents the basal unit of the Claiborne Group." (Hosman, & Weiss, (1991)
Middle Wilcox aquifer	Clastic sedimentary rock **aquifer** (consolidated or semi-consolidated rock)	"Because the middle Wilcox aquifer is composed chiefly of thin interbedded sand, silt, and clay, it has water-bearing characteristics different from those of typical massive and aquifers."
Lower Wilcox aquifer	Clastic sedimentary rock **aquifer** (consolidated or semi-consolidated rock)	"This aquifer only occurs in the Mississippi embayment aquifer system. In the northern part of the Mississippi embayment, a massive sand aquifer, the Fort Pillow Sand of Tennessee, Arkansas, and Missouri (Moore and Brown, 1969), occurs in the lower to middle part of the Wilcox deposits." (Hosman, & Weiss, (1991)
Midway Group	Sedimentary rock **aquitard** (consolidated or semi-consolidated rock)	"The Midway (Paleocene) confining unit (pi. 18) is a thick confining layer that is the base of the flow system for Tertiary aquifers in most of the study area." (Hosman, & Weiss, (1991) | "The Midway consists mostly of dense marine clays, with lesser amounts of calcareous materials in the lower part." (Hosman, & Weiss, (1991)
Nacatoch Sand	Clastic sedimentary rock **aquifer** (consolidated or semi-consolidated rock)	"An important aquifer in the northeastern part of the study area, it is composed of sand beds in the Nacatoch Sand in Arkansas; the McNairy Sand in Missouri, Illinois, Kentucky, and Tennessee; and the McNairy Sand Member of the Ripley Formation of northern Mississippi." (Hosman, & Weiss, (1991)
Ozark Plateaus aquifer system	Carbonate aquifer	"Flat-lying to southward-dipping limestone, dolomite, and sandstone comprise the principal aquifers of the Ozark Plateaus aquifer system" Renken, R. A. (1998)

Hosman, R. L., & Weiss, J. S. (1991). Geohydrologic units of the Mississippi embayment and Texas coastal uplands aquifer systems, south-central United States (No. 1416-B). US Government Printing Office. (https://pubs.er.usgs.gov/publication/pp1416B)

Renken, R. A. (1998). Ground Water Atlas of the United States: Segment 5, Arkansas, Louisiana, Mississippi (No. 730-F, pp. F1-F28). US Geological Survey. (https://pubs.er.usgs.gov/publication/ha730F)

Supplementary Table 16. Hydrostratigraphy details for the Central High Plains

Formation name	Category	Quote
Alluvial Valley & High Plains aquifer	Unconsolidated aquifer	"Unconsolidated alluvial and eolian deposits" Macfarlane, P. A. (1996) | "Alluvial Valley & High Plains aquifer" Macfarlane, P. A. (1996)
Upper Cretaceous aquitard	Sedimentary rock **aquitard** (consolidated or semi-consolidated rock)	"Pierre Shale, Niobrara Chalk, Carlile Shale, Greenhorn Limestone, Graneros Shale**" Macfarlane, P. A. (1996) | "Upper Cretaceous** aquitard**"** P. A. (1996)
Upper Dakota aquifer	Clastic sedimentary rock **aquifer** (consolidated or semi-consolidated rock)	"Buff to light-brown, fine to medium grained sandstone with interbedded shale." Luckey and Mark, 1999 | "Upper Dakota aquifer" Macfarlane, P. A. (1996)
Kiowa shale aquitard	Sedimentary rock **aquitard** (consolidated or semi-consolidated rock)	"Gray to black shale with some fine-grained sandstone in upper part." Luckey and Mark, 1999. | "Kiowa shale aquitard" Macfarlane, P. A. (1996)
Lower Dakota aquifer	Clastic sedimentary rock **aquifer** (consolidated or semi-consolidated rock)	"Longford member" Macfarlane, P. A. (1996) "Cheyenne Sandstone" Macfarlane, P. A. (1996) | "White to buff, fine to medium-grained sandstone with some interbedded shales. Unit contains some conglomerate in lower part." Luckey and Mark, 1999 I "Lower Dakota aquifer" Macfarlane, P. A. (1996)
Morrison-Dockum aquifer	Clastic sedimentary rock **aquifer** (consolidated or semi-consolidated rock)	"Morrison Formation Luckey and Mark, 1999. "Varicolored shale, sandstone, limestone, dolostone, and conglomerate." Luckey and Mark, 1999. "The Dockum Group is composed of sandstone with interbedded shales grading upward to a shaly sandstone or siltstone Luckey and Mark, 1999. "The Dakota Sandstone, the Lytle Sandstone, and the Dockum Group all provide sufficient water for stock and domestic use and may provide sufficient water for irrigation, particularly when combined with the High Plains aquifer or with each other." Luckey and Mark, 1999. "Morrison-Dockum aquifer" Macfarlane, P. A. (1996)
Permian Aquitard	Sedimentary rock **aquitard** (consolidated or semi-consolidated rock)	"Undifferentiated Permian" Macfarlane, P. A. (1996) "Undifferentiated red beds" Luckey et al., 1999. "Predominately red or orange, shale, mudstone, siltstone, sandstone, dolostone, and anhydrite with some gypsum, limestone and halite." Luckey et al., 1999. | "Permian Aquitard" Macfarlane, P. A. (1996)
Cedar Hills Sandstone	Clastic sedimentary rock **aquifer** (consolidated or semi-consolidated rock)	"Cedar Hill Sandstone" Macfarlane, P. A. (1996) "Cedar Hills Sandstone aquifer" Macfarlane, P. A. (1996)

Luckey, R. L., Becker, M.F. (1999). Hydrogeology, water use, and simulation of flow in the High Plains aquifer in northwestern Oklahoma, southeastern Colorado, southwestern Kansas, northeastern New Mexico, and northwestern Texas. US Geological Survey Water-Resources Investigations Report 99-4104, 73 pp. Accessed February 21, 2022 from https://pubs.usgs.gov/wri/wri994104/pdf/wri994104.pdf

Macfarlane, P. A. (1996). An analysis of the upper part of the regional flow system along the southern ground-water flow "corridor" in the Dakota aquifer using a steady-state, vertical profile flow model. Open-File Report. http://www.kgs.ku.edu/Hydro/Publications/1996/OFR96_1d/OFR96-1d.pdf

Supplementary Note 4.2 – Alternate versions of main text Fig. 1

On the following page we provide a version of Fig. 1 (main text) but with greater detail (e.g., including labels for each layer). We could not easily include these details in the main text version (i.e., Fig. 1 in the main text) because of font size limitations.

Supplementary Fig. 9 (following page). Documented occurrence of fossil groundwater in the US and boundaries of our newly created United States Aquifer Database. (a) Boundaries of our locally relevant study areas delineated after reviewing hundreds of primary literature sources describing aquifer boundaries (see Supplementary Table 4 for references). Yellow polygons (n=45) represent aquifer systems where fossil water has been identified (Supplementary Table 1); pink polygons represent other aquifer systems that have also been included in our analyses. (b) The Milk River Aquifer System is dominated by clastic sedimentary aquitards and aquifers, with fossil groundwater reported in some wells with depths exceeding ~150 m [58]. (c) The Denver Basin is a multi-layered clastic sedimentary aquifer system, with fossil groundwater reported in some wells with depths exceeding ~150 m [59]. (d) The Dakota Aquifer System is comprised of carbonate and clastic sedimentary rocks overlying endogenous bedrock, with fossil groundwater reported in some wells in southeastern South Dakota at depths exceeding ~60 m [60] and also in a parallel flow system in Nebraska in some wells with depths exceeding ~170 m [61]. (e) The central portion of the High Plains Aquifer System consists of unconsolidated deposits overlying sedimentary rocks (mostly clastic rocks; e.g., sandstones and mudstones of the Dakota Formation), with fossil groundwater reported in some wells with depths exceeding ~150 m [62]. (f) The North Atlantic Coastal Plain is a multi-layered sedimentary aquifer system underlain by endogenous bedrock, with fossil groundwater reported in some wells with depths exceeding ~80 m [63]. (g) The Floridan Aquifer System consists of a surficial aquifer that is underlain by sedimentary rocks including widespread carbonate aquifers interbedded with confining layers, with fossil water reported in some wells with depths exceeding ~180 m [64]. (h) The Black Warrior River Aquifer System is dominated by clastic consolidated or semi-consolidated aquifers and Paleozoic bedrock, with fossil groundwater reported in some wells with depths exceeding ~150 m [21]. (i) The central portion of the Mississippi Embayment Aquifer System consists of unconsolidated alluvium overlying consolidated clastic sedimentary rocks, with fossil groundwater reported in some wells with depths exceeding ~100 m [1]. (j) The western portion of the Carrizo-Wilcox Aquifer System is a multi-layered sedimentary aquifer system, with fossil water reported at depths exceeding ~400 m [65]. (k) The Mojave Basin consists of alluvium overlying endogenous rock, with fossil groundwater reported in some wells with depths exceeding ~200 m [66]. (l) The Cuyama Valley is comprised of alluvial materials overlying (semi)consolidated clastic bedrock, with fossil water reported in some wells with depths exceeding ~200 m [20]. (m) The northern portion of California's Central Valley Aquifer System is comprised is comprised of alluvial materials overlying (semi)consolidated clastic bedrock, with fossil water reported in some wells with depths of 115 to 300 m [67]. Each of the twelve cross sections (panels b-m) are based on descriptions and figures presented by refs.[20,58,68-77]. See Supplementary Tables 5-16 for detailed descriptions of hydrostratigraphy.

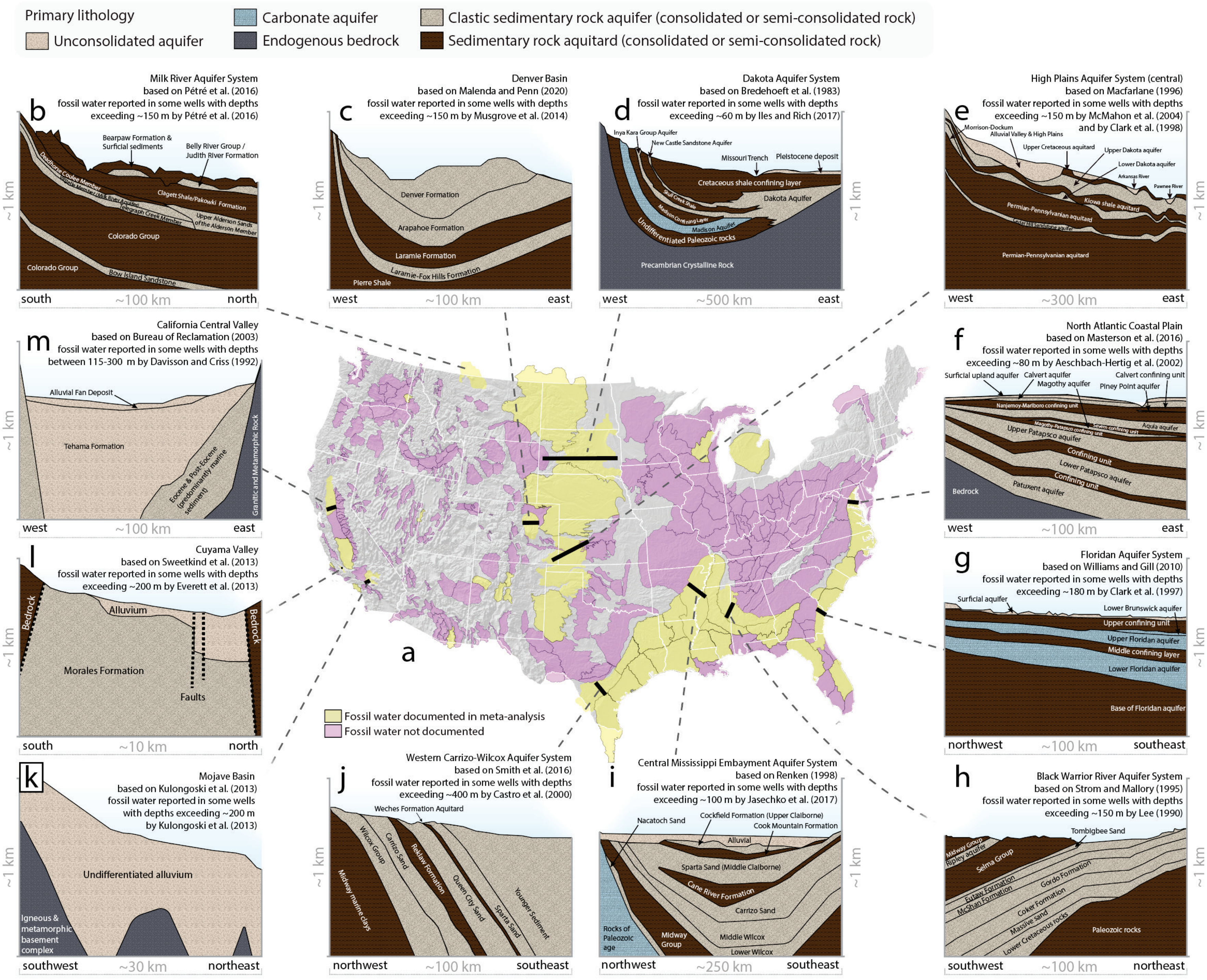

Primary lithology
Unconsolidated aquifer
Carbonate aquifer
Endogenous bedrock
Clastic sedimentary rock aquifer (consolidated or semi-consolidated rock)
Sedimentary rock aquitard (consolidated or semi-consolidated rock)
b
Milk River Aquifer System
based on Pétré et al. (2016)
fossil water reported in some wells with depths exceeding ~150 m by Pétré et al. (2016)
Bearpaw Formation & Surficial sediments
Belly River Group / Judith River Formation
Deadhorse Coulee Member
Clagett Shale/Pakowki Formation
Telegraph Creek Member
Upper Alderson Sands of the Alderson Member
Colorado Group
Colorado Group
Bow Island Sandstone
~1 km
south
~100 km
north
c
Denver Basin
based on Malenda and Penn (2020)
fossil water reported in some wells with depths exceeding ~150 m by Musgrove et al. (2014)
Denver Formation
Arapahoe Formation
Laramie Formation
Laramie-Fox Hills Formation
Pierre Shale
~1 km
west
~100 km
east
d
Dakota Aquifer System
based on Bredehoeft et al. (1983)
fossil water reported in some wells with depths exceeding ~60 m by Iles and Rich (2017)
Inya Kara Group Aquifer
New Castle Sandstone Aquifer
Missouri Trench
Pleistocene deposit
Cretaceous shale confining layer
Dakota Aquifer
Skull Creek Shale
Madison Confining Layer
Madison Aquifer
Undifferentiated Paleozoic rocks
Precambrian Crystalline Rock
~1 km
west
~500 km
east
e
High Plains Aquifer System (central)
based on Macfarlane (1996)
fossil water reported in some wells with depths exceeding ~150 m by McMahon et al. (2004) and by Clark et al. (1998)
Morrison-Dockum
Alluvial Valley & High Plains
Upper Cretaceous aquitard
Upper Dakota aquifer
Lower Dakota aquifer
Arkansas River
Pawnee River
Kiowa shale aquitard
Permian-Pennsylvanian aquitard
Cedar Hill Sandstone aquifer
Permian-Pennsylvanian aquitard
~1 km
west
~300 km
east
m
California Central Valley
based on Bureau of Reclamation (2003)
fossil water reported in some wells with depths between 115-300 m by Davisson and Criss (1992)
Alluvial Fan Deposit
Tehama Formation
Eocene & Post-Eocene (predominantly marine sediment)
Granitic and Metamorphic Rock
~1 km
west
~100 km
east
f
North Atlantic Coastal Plain
based on Masterson et al. (2016)
fossil water reported in some wells with depths exceeding ~80 m by Aeschbach-Hertig et al. (2002)
Surficial upland aquifer
Calvert aquifer
Magothy aquifer
Calvert confining unit
Piney Point aquifer
Nanjemoy-Marlboro confining unit
Magothy-Patapsco confining unit
Severn confining unit
Aquia aquifer
Upper Patapsco aquifer
Confining unit
Lower Patapsco aquifer
Confining unit
Patuxent aquifer
Bedrock
~1 km
west
~100 km
east
l
Cuyama Valley
based on Sweetkind et al. (2013)
fossil water reported in some wells with depths exceeding ~200 m by Everett et al. (2013)
Bedrock
Alluvium
Bedrock
Morales Formation
Faults
~1 km
south
~10 km
north
a
Fossil water documented in meta-analysis
Fossil water not documented
g
Floridan Aquifer System
based on Williams and Gill (2010)
fossil water reported in some wells with depths exceeding ~180 m by Clark et al. (1997)
Surficial aquifer
Lower Brunswick aquifer
Upper confining unit
Upper Floridan aquifer
Middle confining layer
Lower Floridan aquifer
Base of Floridan aquifer
~1 km
northwest
~100 km
southeast
k
Mojave Basin
based on Kulongoski et al. (2013)
fossil water reported in some wells with depths exceeding ~200 m by Kulongoski et al. (2013)
Undifferentiated alluvium
Igneous & metamorphic basement complex
~1 km
southwest
~30 km
northeast
j
Western Carrizo-Wilcox Aquifer System
based on Smith et al. (2016)
fossil water reported in some wells with depths exceeding ~400 m by Castro et al. (2000)
Weches Formation Aquitard
Midway marine clays
Wilcox Group
Carrizo Sand
Reklaw Formation
Queen City Sand
Sparta Sand
Younger Sediment
~1 km
northwest
~100 km
southeast
i
Central Mississippi Embayment Aquifer System
based on Renken (1998)
fossil water reported in some wells with depths exceeding ~100 m by Jasechko et al. (2017)
Nacatoch Sand
Cockfield Formation (Upper Claiborne)
Cook Mountain Formation
Alluvial
Sparta Sand (Middle Claiborne)
Cane River Formation
Carrizo Sand
Middle Wilcox
Lower Wilcox
Rocks of Paleozoic age
Midway Group
~1 km
northwest
~250 km
southeast
h
Black Warrior River Aquifer System
based on Strom and Mallory (1995)
fossil water reported in some wells with depths exceeding ~150 m by Lee (1990)
Tombigbee Sand
Midway Group Ripley aquifer
Selma Group
Eutaw Formation
McShan Formation
Gordo Formation
Coker Formation
Massive sand
Lower Cretaceous rocks
Paleozoic rocks
~1 km
southwest
~100 km
northeast

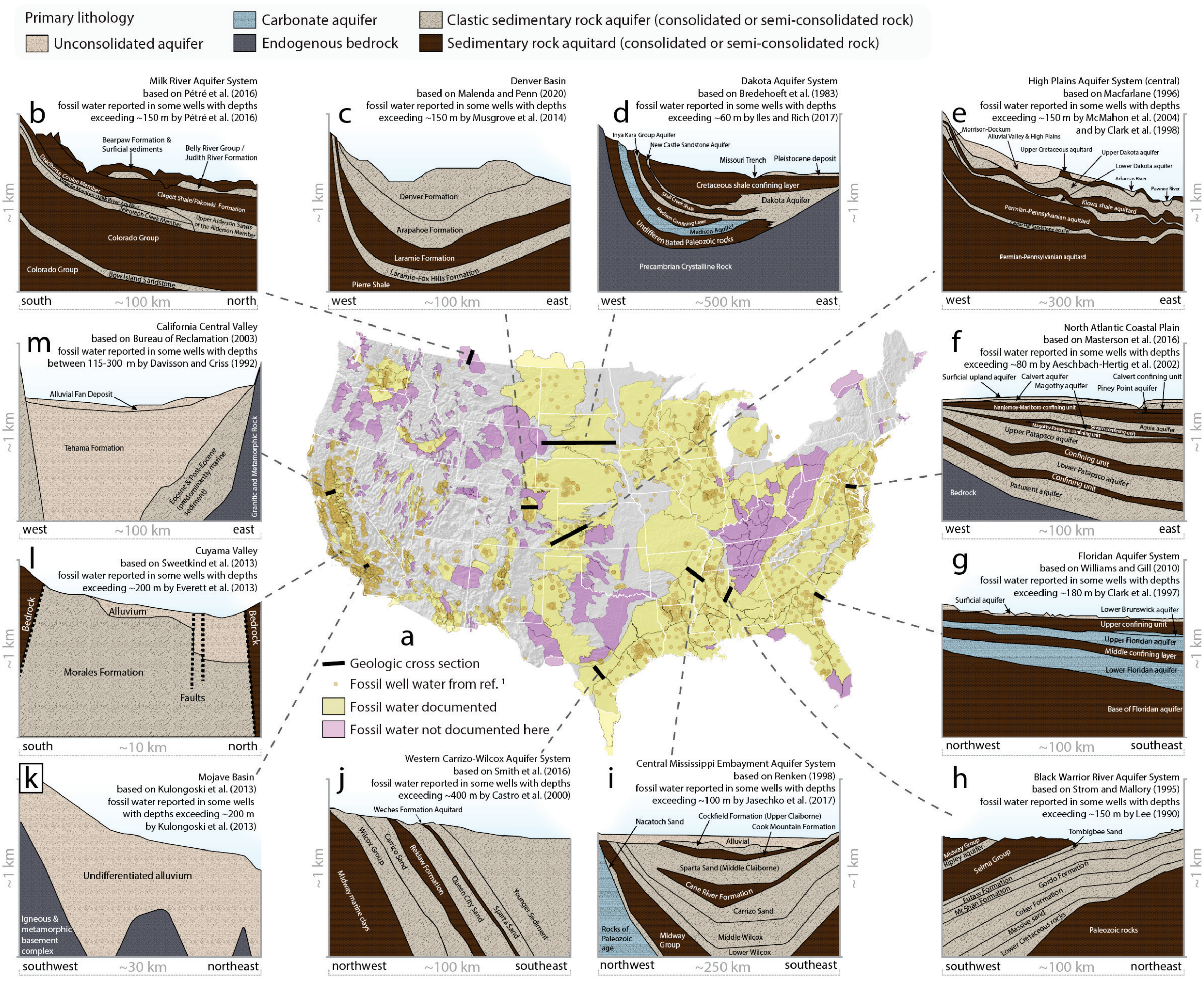
Primary lithology
Unconsolidated aquifer
Carbonate aquifer
Endogenous bedrock
Clastic sedimentary rock aquifer (consolidated or semi-consolidated rock)
Sedimentary rock aquitard (consolidated or semi-consolidated rock)
b
Milk River Aquifer System
based on Pétré et al. (2016)
fossil water reported in some wells with depths exceeding ~150 m by Pétré et al. (2016)
Bearpaw Formation & Surficial sediments
Belly River Group / Judith River Formation
Deadhorse Coulee Member
Clagett Shale/Pakowki Formation
Telegraph Creek Member
Upper Alderson Sands of the Alderson Member
Colorado Group
Colorado Group
Bow Island Sandstone
south
~100 km
north
~1 km
c
Denver Basin
based on Malenda and Penn (2020)
fossil water reported in some wells with depths exceeding ~150 m by Musgrove et al. (2014)
Denver Formation
Arapahoe Formation
Laramie Formation
Laramie-Fox Hills Formation
Pierre Shale
west
~100 km
east
~1 km
d
Dakota Aquifer System
based on Bredehoeft et al. (1983)
fossil water reported in some wells with depths exceeding ~60 m by Iles and Rich (2017)
Inya Kara Group Aquifer
New Castle Sandstone Aquifer
Missouri Trench
Pleistocene deposit
Cretaceous shale confining layer
Dakota Aquifer
Skull Creek Shale
Madison Confining Layer
Madison Aquifer
Undifferentiated Paleozoic rocks
Precambrian Crystalline Rock
west
~500 km
east
~1 km
e
High Plains Aquifer System (central)
based on Macfarlane (1996)
fossil water reported in some wells with depths exceeding ~150 m by McMahon et al. (2004) and by Clark et al. (1998)
Morrison-Dockum
Alluvial Valley & High Plains
Upper Cretaceous aquitard
Upper Dakota aquifer
Lower Dakota aquifer
Arkansas River
Pawnee River
Kiowa shale aquitard
Permian-Pennsylvanian aquitard
Cedar Hill Sandstone aquifer
Permian-Pennsylvanian aquitard
west
~300 km
east
~1 km
m
California Central Valley
based on Bureau of Reclamation (2003)
fossil water reported in some wells with depths between 115-300 m by Davisson and Criss (1992)
Alluvial Fan Deposit
Tehama Formation
Eocene & Post-Eocene (predominantly marine sediment)
Granitic and Metamorphic Rock
west
~100 km
east
~1 km
f
North Atlantic Coastal Plain
based on Masterson et al. (2016)
fossil water reported in some wells with depths exceeding ~80 m by Aeschbach-Hertig et al. (2002)
Surficial upland aquifer
Calvert aquifer
Magothy aquifer
Calvert confining unit
Piney Point aquifer
Nanjemoy-Marlboro confining unit
Magothy-Patapsco confining unit
Severn confining unit
Aquia aquifer
Upper Patapsco aquifer
Confining unit
Lower Patapsco aquifer
Confining unit
Patuxent aquifer
Bedrock
west
~100 km
east
~1 km
l
Cuyama Valley
based on Sweetkind et al. (2013)
fossil water reported in some wells with depths exceeding ~200 m by Everett et al. (2013)
Bedrock
Alluvium
Bedrock
Morales Formation
Faults
south
~10 km
north
~1 km
a
Geologic cross section
Fossil well water from ref. 1
Fossil water documented
Fossil water not documented here
g
Floridan Aquifer System
based on Williams and Gill (2010)
fossil water reported in some wells with depths exceeding ~180 m by Clark et al. (1997)
Surficial aquifer
Lower Brunswick aquifer
Upper confining unit
Upper Floridan aquifer
Middle confining layer
Lower Floridan aquifer
Base of Floridan aquifer
northwest
~100 km
southeast
~1 km
k
Mojave Basin
based on Kulongoski et al. (2013)
fossil water reported in some wells with depths exceeding ~200 m by Kulongoski et al. (2013)
Undifferentiated alluvium
Igneous & metamorphic basement complex
southwest
~30 km
northeast
~1 km
j
Western Carrizo-Wilcox Aquifer System
based on Smith et al. (2016)
fossil water reported in some wells with depths exceeding ~400 m by Castro et al. (2000)
Weches Formation Aquitard
Midway marine clays
Wilcox Group
Carrizo Sand
Reklaw Formation
Queen City Sand
Sparta Sand
Younger Sediment
northwest
~100 km
southeast
~1 km
i
Central Mississippi Embayment Aquifer System
based on Renken (1998)
fossil water reported in some wells with depths exceeding ~100 m by Jasechko et al. (2017)
Nacatoch Sand
Cockfield Formation (Upper Claiborne)
Cook Mountain Formation
Alluvial
Sparta Sand (Middle Claiborne)
Cane River Formation
Carrizo Sand
Rocks of Paleozoic age
Midway Group
Middle Wilcox
Lower Wilcox
northwest
~250 km
southeast
~1 km
h
Black Warrior River Aquifer System
based on Strom and Mallory (1995)
fossil water reported in some wells with depths exceeding ~150 m by Lee (1990)
Tombigbee Sand
Midway Group
Ripley aquifer
Selma Group
Eutaw Formation
McShan Formation
Gordo Formation
Coker Formation
Massive sand
Lower Cretaceous rocks
Paleozoic rocks
southwest
~100 km
northeast
~1 km

Supplementary Fig. 10 (previous page). Reported fossil groundwater in the US from ref. [1] and boundaries of our newly created United States Aquifer Database. (a) Boundaries of our locally relevant study areas delineated after reviewing hundreds of primary literature sources describing aquifer boundaries (see Supplementary Table 4 for references). Yellow polygons (n=111) represent aquifer systems where fossil water has been identified in ref. [1]; pink polygons represent other aquifer systems that have also been included in our analyses. Gold circles represent wells where fossil water has been reported in ref. [1]. (b) The Milk River Aquifer System is dominated by clastic sedimentary aquitards and aquifers, with fossil groundwater reported in some wells with depths exceeding ~150 m [58] . (c) The Denver Basin is a multi-layered clastic sedimentary aquifer system, with fossil groundwater reported in some wells with depths exceeding ~150 m [59]. (d) The Dakota Aquifer System is comprised of carbonate and clastic sedimentary rocks overlying endogenous bedrock, with fossil groundwater reported in some wells in southeastern South Dakota at depths exceeding ~60 m [60] and also in a parallel flow system in Nebraska in some wells with depths exceeding ~170 m [61]. (e) The central portion of the High Plains Aquifer System consists of unconsolidated deposits overlying sedimentary rocks (mostly clastic rocks; e.g., sandstones and mudstones of the Dakota Formation), with fossil groundwater reported in some wells with depths exceeding ~150 m [62]. (f) The North Atlantic Coastal Plain is a multi-layered sedimentary aquifer system underlain by endogenous bedrock, with fossil groundwater reported in some wells with depths exceeding ~80 m [63]. (g) The Floridan Aquifer System consists of a surficial aquifer that is underlain by sedimentary rocks including widespread carbonate aquifers interbedded with confining layers, with fossil water reported in some wells with depths exceeding ~180 m [64]. (h) The Black Warrior River Aquifer System is dominated by clastic consolidated or semi-consolidated aquifers and Paleozoic bedrock, with fossil groundwater reported in some wells with depths exceeding ~150 m [21]. (i) The central portion of the Mississippi Embayment Aquifer System consists of unconsolidated alluvium overlying consolidated clastic sedimentary rocks, with fossil groundwater reported in some wells with depths exceeding ~100 m [1]. (j) The western portion of the Carrizo-Wilcox Aquifer System is a multi-layered sedimentary aquifer system, with fossil water reported at depths exceeding ~400 m [65]. (k) The Mojave Basin consists of alluvium overlying endogenous rock, with fossil groundwater reported in some wells with depths exceeding ~200 m [66]. (l) The Cuyama Valley is comprised of alluvial materials overlying (semi)consolidated clastic bedrock, with fossil water reported in some wells with depths exceeding ~200 m [20]. (m) The northern portion of California's Central Valley Aquifer System is comprised is comprised of alluvial materials overlying (semi)consolidated clastic bedrock, with fossil water reported in some wells with depths of 115 to 300 m [67]. Each of the twelve cross sections (panels b-m) are based on descriptions and figures presented by refs.[20,58,68-77]. See Supplementary Tables 5-16 for detailed descriptions of hydrostratigraphy.

Supplementary Note 5 – Statistical relationships between groundwater level change over time and the prevalence of deep wells

We explore relationships between the prevalence of deep wells (>100 m to >300 m) and groundwater level changes over time. Specifically, we test two different types of relationships.

a) We evaluate correlations between the proportion of recorded wells within an aquifer system's boundaries that exceed 200±100m *versus* the median groundwater level change over time (median of all monitoring wells' Theil-Sen slopes, determined for any aquifers with sufficient data for analyses as described in Supplementary Note 2.3 and Supplementary Fig. 5). Positive correlation coefficients (Spearman ρ) suggest that aquifer systems where a larger proportion of wells tend to be deep also tend to have higher magnitude groundwater level declines (and are less likely to host monitoring wells where the typical time series (i.e., median change over time) tends to show groundwater level shallowing over time)

b) Second, we evaluate correlations between the proportion of recorded wells within an aquifer system's boundaries that exceed 200±100m *versus* the proportion of all monitoring wells within the aquifer system (with sufficient data for analysis, see Methods) that have Theil-Sen slope values indicative of groundwater level declines over time (i.e., the proportion of monitoring wells within the aquifer system bounds with positive Theil-Sen Slopes describing variations of 'depth to groundwater' versus 'measurement date'). Positive correlation coefficients (Spearman ρ) suggest that aquifer systems where a larger proportion of wells tend to be deep also tend to have a greater proportion of monitoring wells that indicate declining groundwater levels over time.

Because we have three thresholds for 'deep wells' (i.e., the (i) proportion of wells deeper than 100 m, (ii) proportion of wells deeper than 200 m, (iii) proportion of wells deeper than 300 m) and two different plots for which we determine rank correlation coefficients (i.e., "a") and "b)" above in this Supplementary Note 5), we calculate six Spearman rank correlation coefficients for each studied time interval. Further, because we study five unique time intervals (i.e., (i) 1950-1975, (ii) 1975-2000, (iii) 2000-2015, (iv) 1950-2015, and (v) 1975-2015) we report a total of six correlation coefficients for each time interval and therefore complete a total of 24 correlation coefficient calculations (i.e., six correlation calculations per time interval, multiplied by five time-intervals).

The rank correlation coefficients are presented in the tables below (i.e., within this Supplementary Note 5). Further, we present a figure for one of the time intervals to provide an example of the substantial scatter in these statistical relationships and provide a clearer view of the statistical relationships we summarize in the tables within this Supplementary Note 5.

We stress that although positive correlation coefficients are more common than negative correlation coefficients (Supplementary Table 17), the use of fossil groundwater does not have to mean that groundwater use is non-renewable. Indeed, the prevalence of deep wells may also be indicative of responses of groundwater users; specifically, some groundwater users may construct deeper wells where groundwater levels are declining where hydrogeologic conditions enable such a construction activities.

Supplementary Table 17. Statistical relationships between the proportion of wells within an aquifer system with depths exceeding 100 m, 200 m or 300 m versus two metrics of groundwater level change over time for the time interval 2000-2015: (i) the fraction of all monitoring wells within an aquifer system indicating that groundwater levels have declined over time, and (ii) the median Theil-Sen slope determined on the basis of all monitoring wells within the aquifer system with sufficient data for analyses. Spearman rank correlation coefficients (ρ) are displayed. Each row of the table represents a different time interval (e.g., 1950-1975). Each gray-shaded set of columns represent a different well depth threshold (i.e., "100 m" corresponds to the proportion of wells within an aquifer system with depths exceeding 100 m). For an example of scatterplots of data associated with the time interval 2000-2015 (i.e., row 4 in this table) interval see Supplementary Fig. 11).

**Timespan**	**Fraction of wells deeper than 100 m**		**Fraction of wells deeper than 200 m**		**Fraction of wells deeper than 300 m**	
	Median* water level variation over time (median Theil-Sen slope among monitoring wells)	Proportion* of wells exhibiting water level declines (fraction of monitoring wells with Theil-Sen slopes of greater than zero	Median* water level variation over time (median Theil-Sen slope among monitoring wells)	Proportion* of wells exhibiting water level declines (fraction of monitoring wells with Theil-Sen slopes of greater than zero	Median* water level variation over time (median Theil-Sen slope among monitoring wells)	Proportion* of wells exhibiting water level declines (fraction of monitoring wells with Theil-Sen slopes of greater than zero
1950-1975	$\rho = 0.294$	$\rho = 0.226$	$\rho = 0.279$	$\rho = 0.189$	$\rho = 0.262$	$\rho = 0.154$
1975-2000	$\rho = 0.063$	$\rho = 0.045$	$\rho = -0.008$	$\rho = -0.015$	$\rho = -0.069$	$\rho = -0.077$
2000-2015	$\rho = 0.208$	$\rho = 0.210$	$\rho = 0.185$	$\rho = 0.204$	$\rho = 0.125$	$\rho = 0.130$
1950-2015	$\rho = 0.299$	$\rho = 0.212$	$\rho = 0.251$	$\rho = 0.158$	$\rho = 0.192$	$\rho = 0.082$
1975-2015	$\rho = 0.222$	$\rho = 0.135$	$\rho = 0.176$	$\rho = 0.096$	$\rho = 0.119$	$\rho = 0.026$

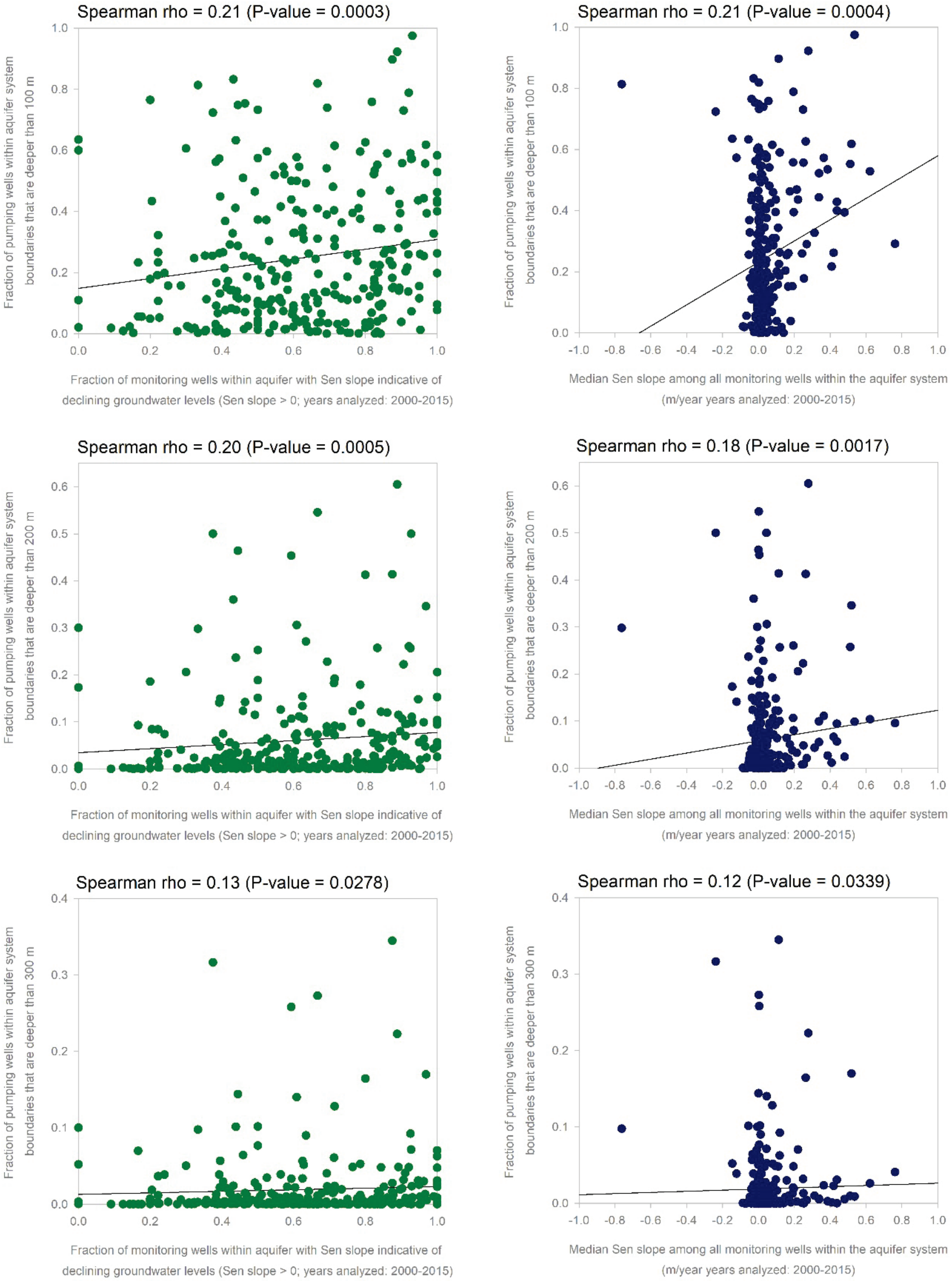

Supplementary Fig. 11. Statistical relationships between the proportion of wells within an aquifer system with depths exceeding 200±100 m versus two metrics of groundwater level change over time: (i – left side column of plots with **green points**) the fraction of all monitoring wells within an aquifer system indicating that groundwater levels have declined over time (i.e., the proportion of monitoring

wells within an aquifer system with a Theil-Sen slope exceeding zero, where the Theil-Sen slope reflects the statistical relationship between depth to groundwater versus measurement date for a given monitoring well); and, (ii – right side plots with **blue points**) the median Theil-Sen slope determined on the basis of all monitoring wells within the aquifer system with sufficient data for analyses (where the Theil-Sen slope reflects the statistical relationship between depth to groundwater versus measurement date for a given monitoring well). We only examined aquifer systems with at least 5 monitoring wells meeting our criteria for analyses for a given time interval. The Spearman rank correlation coefficient is displayed on top of each plot; these values are replicated in Supplementary Table 17.

Supplementary Note 6 – Groundwater quality in shallow versus deep wells

Supplementary Note 6.1 – Compilation of US groundwater quality data

Here we describe how groundwater quality data for the contiguous US were downloaded. We data from https://www.waterqualitydata.us/#siteType=Well&mimeType=csv&sorted=no&providers=NWIS&providers=STEWARDS&providers=STORET; specifically, we entered the following terms in the search field entitled "Characteristics": "Arsenic (NWIS, STEWARDS, STORET)", "Nitrate (NWIS, STORET)", "Total Dissolved Solids (NWIS, STEWARDS, STORET)" (complete query URLs (entered January 11, 2022) are:

- https://www.waterqualitydata.us/#siteType=Well&characteristicName=Nitrate&mimeType=csv&sorted=no&dataProfile=resultPhysChem&providers=NWIS&providers=STEWARDS&providers=STORET *and*
- https://www.waterqualitydata.us/#siteType=Well&characteristicName=Arsenic&mimeType=csv&sorted=no&dataProfile=resultPhysChem&providers=NWIS&providers=STEWARDS&providers=STORET *and*
- https://www.waterqualitydata.us/#siteType=Well&characteristicName=Total%20dissolved%20solids&mimeType=csv&sorted=no&dataProfile=resultPhysChem&providers=NWIS&providers=STEWARDS&providers=STORET

Here we detail steps to quality check each dataset: (i) arsenic, (ii) nitrate, (iii) total dissolved solids.

(i) To quality control the downloaded arsenic dataset, we excluded measurements returning a below detection result if the detection limit equaled or exceed 10 µg/L. We also any measurement that does not include the term "Dissolved" in the field entitled "ResultSampleFractionText (this step excluded measurements of "Total" arsenic, as we concluded that including "Total" arsenic measurements in our dataset would implicitly and inappropriately treat dissolved and total arsenic measurements as equivalent in our analyses). We excluded any measurements with a "ActivityMediaSubdivisionName" value other than "Groundwater". A small number of measurements (n=7) reported "Present Above Quantification Limit"; these samples were excluded from further analyses, as we did not analyze data for an upper limit value for the analytical quantification limit. We excluded measurements with the flag "Detected Not Quantified" in the field "ResultDetectionConditionText". Well depth data were compiled from the field entitled "WellDepthMeasure/MeasureValue" first and, if this field was empty, we compiled well depth data from the field entitled "WellHoleDepthMeasure/MeasureValue". For stations with multiple measurements meeting the aforementioned quality control criteria, we analyzed only the most recent measurement.

(ii) To quality control the downloaded nitrate dataset, we excluded all measurements that does not include the term "Dissolved" in the field entitled "ResultSampleFractionText". We excluded measurements that do not clearly specify sample media as "Groundwater" in the field "ActivityMediaSubdivisionName". We excluded records reporting "Detected Not Quantified" or "Systematic Contamination" under the field "ResultDetectionConditionText". We excluded measurements if the units did not specify 'as NO3' or 'as N' (i.e., some records specify only "mg/l" or 'ug/l' or 'ppm', and were thus deleted as this information was insufficient to determine if the units are 'as NO3' or 'as N'). We converted nitrate data into consistent units of "nitrate as N" (as some measurements and stated detection limits are recorded in units of "mg/l as NO3"). We excluded measurements recording a below detection limit measurement if the stated detection limit equaled or exceeded 10 mg/L NO_3^- as N. Well depth data were compiled from the field entitled "WellDepthMeasure/MeasureValue" first and, if this field did not contain a number, we compiled well depth data from the field entitled "WellHoleDepthMeasure/MeasureValue" instead. For stations with

multiple measurements meeting the aforementioned quality control criteria, we analyzed only the most recent measurement.

(iii) To quality control total dissolved solids (TDS) data, we excluded all records that does not specify “Dissolved” in the field “ResultSampleFractionText”. We excluded measurements that do not state “Groundwater” under the field entitled “ActivityMediaSubdivisionName”. We deleted any records with a below detection measurement if the detection limit was stated to be equal to or greater than 1000 mg/L. We excluded records reporting “Detected Not Quantified” or “Systematic Contamination” or “Present above Quantification Limit” under the field “ResultDetectionConditionText”. We converted units of “tons/ac ft” to mg/L by multiplying by the 735.47. We excluded a records reporting measurements in units of “tons/day”. Well depth data were compiled from the field entitled “WellDepthMeasure/MeasureValue” first and, if this field was empty, we compiled well depth data from the field entitled “WellHoleDepthMeasure/MeasureValue”. For stations with multiple measurements meeting the aforementioned quality control criteria, we analyzed only the most recent measurement.

Supplementary Note 6.2 – Groundwater quality in deep and shallow wells in aquifer systems

The following figures provide, for each of our three study analytes (dissolved arsenic, dissolved nitrate, total dissolved solids), information about the spatial distribution of well waters that exceed the following analyte-specific threshold concentrations: ***Dissolved arsenic (As):*** threshold concentration of 10 µg/L; ***Dissolved nitrate (NO_3):*** threshold concentration of 10 mg/L (measured as N; i.e., 10 mg/L NO_3-N); and, ***Total dissolved solids (TDS):*** threshold concentration of 3,000 mg/L.

The following figures’ panels provide the following information:

a) Panel (a) presents a map of well water quality measurements made in wells with total depths shallower than 50 m. Colored points are those that exceed the analyte-specific concentration threshold (for example, a well water sample with more than 10 mg/L NO_3-N).
b) Panel (b) presents a map of aquifers shaded by the fraction of shallow wells with groundwater quality data (<50 m; see panel (a)) that have concentrations exceeding the analyte-specific threshold (e.g., the proportion of wells within the aquifer system that have > 10 mg/L NO_3-N). We only shade areas for aquifer systems with at least n=20 water quality measurements.
c) Panel (c) presents a map of well water quality measurements made in wells with total depths deeper than 100 m and shallower than 300 m. Colored points are those that exceed the analyte-specific concentration threshold (e.g., a well water sample with more than 10 mg/L NO_3-N).
d) Panel (d) presents a map of aquifers shaded by the fraction of deep wells with groundwater quality data (100-300 m; see panel (c)) that have concentrations exceeding the analyte-specific threshold (e.g., the proportion of wells within the aquifer system that have > 10 mg/L NO_3-N). We only shade areas for aquifer systems with at least n=20 water quality measurements.
e) Panel (e) plots the fraction of wells that have concentrations exceeding the analyte-specific threshold. Downward-pointing dark blue triangles represent wells with depths between 100-300 m (i.e., values used to shade aquifers in panel d), whereas upward-pointing light blue triangles represent wells with depths shallower than 50 m (i.e., values used to shade aquifers in panel b). We only plot aquifer systems with at least n=20 water quality measurements for both shallow (<50 m) and deep (100-300 m) well depth intervals (i.e., aquifers that appear both panel (b) and panel (d)).

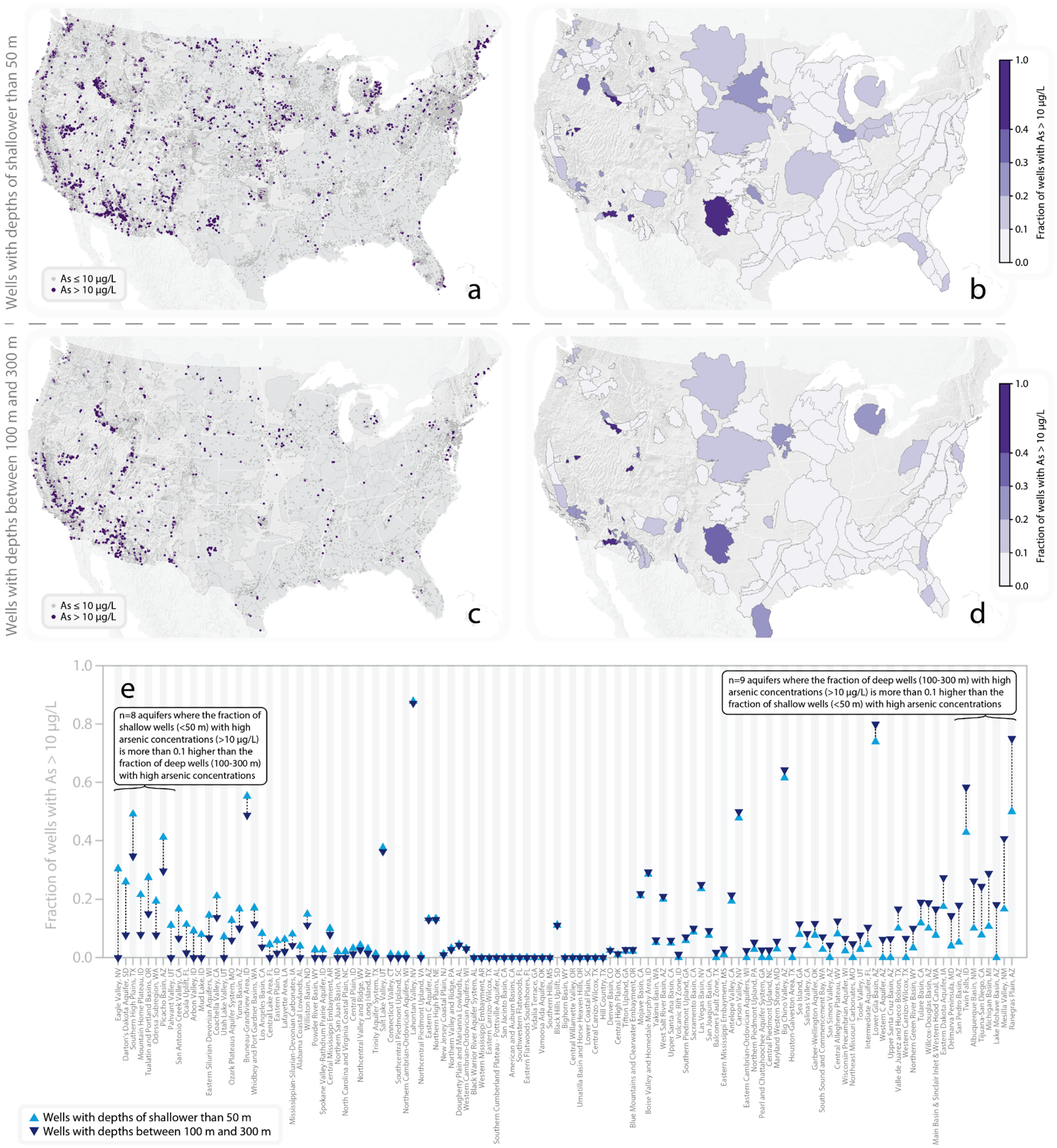

Supplementary Fig. 12. Spatial distributions of dissolved arsenic in US well waters. (a) Well water arsenic measurements in wells with total depths shallower than 50 m; purple colored points are those that exceed 10 µg/L. (b) Aquifers shaded by the fraction of shallow wells (<50 m; see panel (a)) that have arsenic concentrations exceeding 10 µg/L. (c) Well water arsenic measurements in wells with total depths deeper than 100 m and shallower than 300 m; purple colored points are those that exceed 10 µg/L. (d) Aquifers shaded by the fraction of deep wells (100-300 m; see panel (c)) that have arsenic concentrations exceeding 10 µg/L. (e) The fraction of wells that have arsenic concentrations exceeding 10 µg/L for individual aquifer systems (see labels at base of figure). Downward-pointing dark blue triangles represent wells with depths between 100-300 m (i.e., values used to shade aquifers in panel (d)), whereas upward-pointing light blue triangles represent wells with depths shallower than 50 m (i.e., values used to shade aquifers in panel (b)). Dashed vertical black lines mark differences between shallower wells (<50 m; upward-pointing light blue triangles) and deeper wells (100-300 m; downward-pointing dark blue triangles) in their respective frequencies with which these two groups of wells (shallow, deep) have high-arsenic concentrations (>10 µg/L).

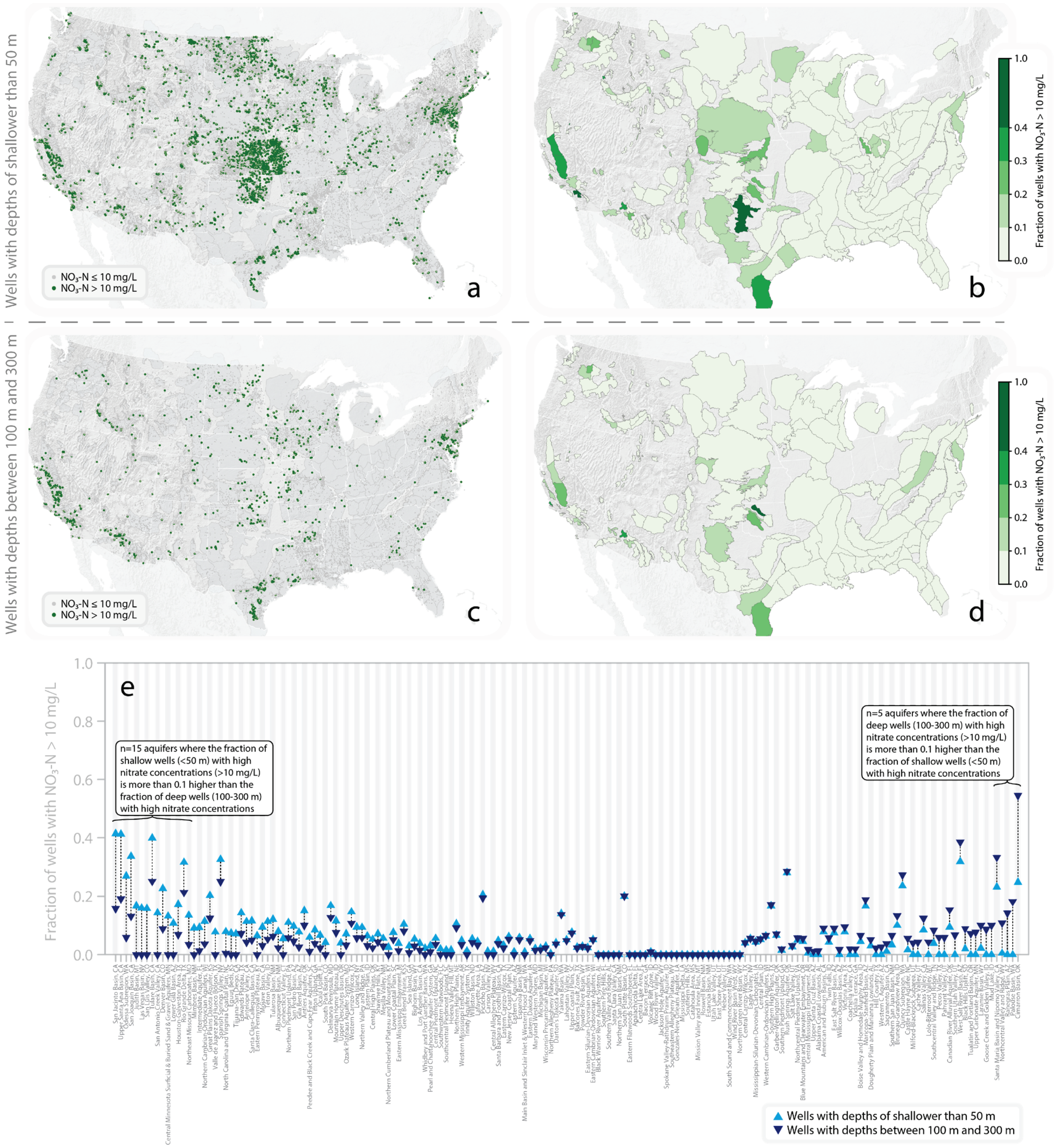

Supplementary Fig. 13. Spatial distributions of dissolved nitrate (NO_3) in US well waters. (a) Well water NO_3 measurements in wells with total depths shallower than 50 m; green colored points are those that exceed 10 mg/L (NO_3 as N). (b) Aquifers shaded by the fraction of shallow wells (<50 m; see panel (a)) that have nitrate concentrations exceeding 10 mg/L. (c) Well water nitrate measurements in wells with total depths deeper than 100 m and shallower than 300 m; green colored points are those that exceed 10 mg/L. (d) Aquifers shaded by the fraction of deep wells (100-300 m; see panel (c)) that have nitrate concentrations exceeding 10 mg/L. (e) The fraction of wells that have nitrate concentrations exceeding 10 mg/L for individual aquifer systems (see labels at base of figure). Downward-pointing dark blue triangles represent wells with depths between 100-300 m (i.e., values used to shade aquifers in panel (d)), whereas upward-pointing light blue triangles represent wells with depths shallower than 50 m (i.e., values used to shade aquifers in panel (b)). Dashed vertical black lines mark differences among shallower wells (<50 m; upward-pointing light blue triangles) and deeper wells (100-300 m; downward-pointing dark blue triangles) in their respective frequencies with which these two groups of wells (shallow, deep) have high-NO_3 (>10 mg/L as N).

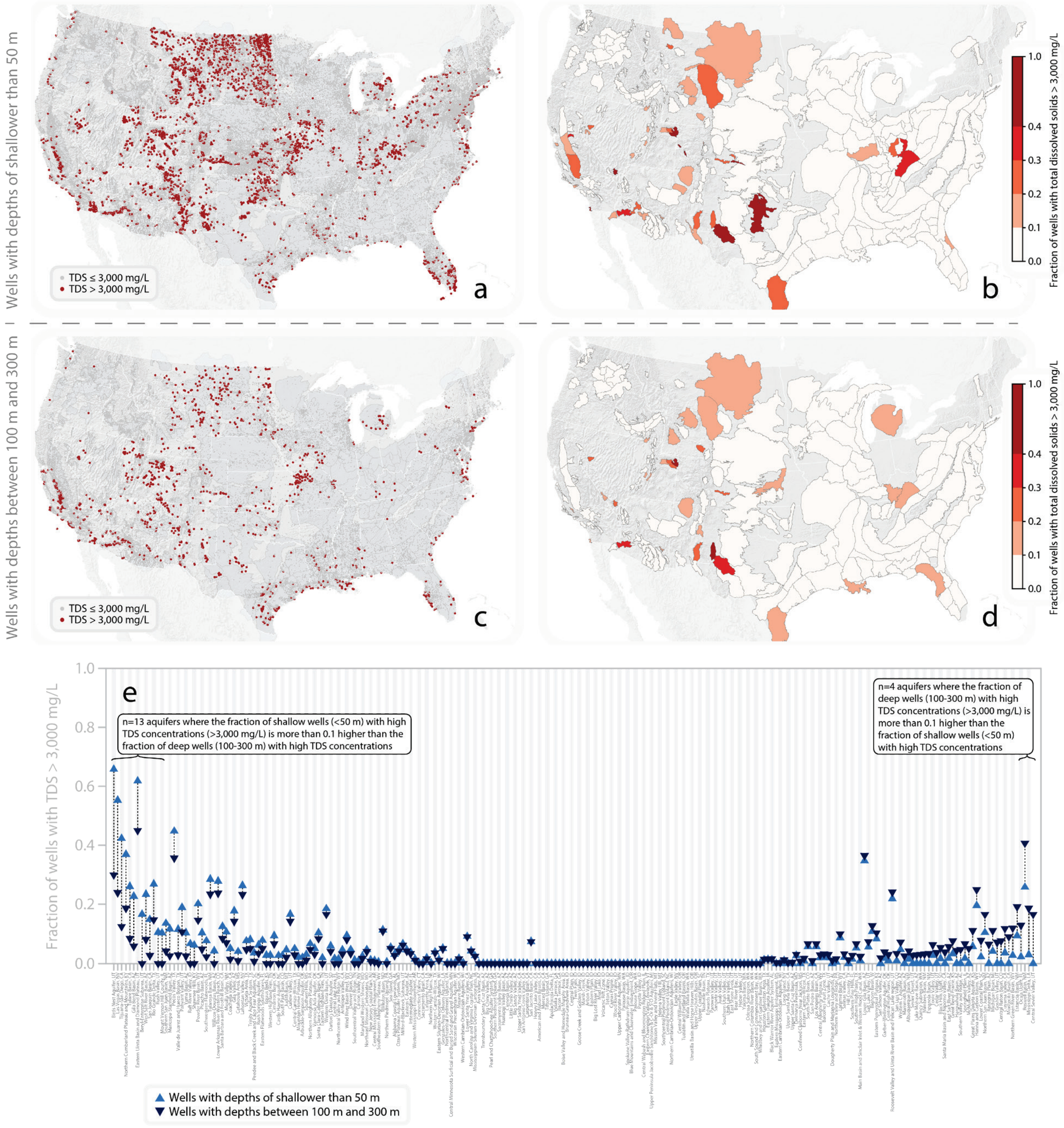

Supplementary Fig. 14. Spatial distributions of total dissolved solids (TDS) in US well waters. (a) Well water TDS measurements in wells with total depths shallower than 50 m; red colored points are those that exceed 3,000 mg/L. (b) Aquifers shaded by the fraction of shallow wells (<50 m; see panel (a)) that have TDS concentrations exceeding 3,000 mg/L. (c) Well water TDS measurements in wells with total depths deeper than 100 m and shallower than 300 m; red colored points are those that exceed 3,000 mg/L. (d) Aquifers shaded by the fraction of deep wells (100-300 m; see panel (c)) that have TDS concentrations exceeding 3,000 mg/L. (e) The fraction of wells that have TDS concentrations exceeding 3,000 mg/L for individual aquifer systems (see labels at base of figure). Downward-pointing dark blue triangles represent wells with depths between 100-300 m (i.e., values used to shade aquifers in panel (d)), whereas upward-pointing light blue triangles represent wells with depths shallower than 50 m (i.e., values used to shade aquifers in panel (b)). Dashed vertical black lines mark differences between shallower wells (<50 m; upward-pointing triangles) and deeper wells (100-300 m; downward-pointing triangles) in their respective frequencies with which these two groups of wells (shallow, deep) have high-TDS (>3,000 mg/L).

Supplementary Note 7 – Examples of marketing linked to the age of water

Here we document eight examples of webpages that refer to the age of water associated with their product (e.g., beer) or establishment (e.g., spa) on a website (Supplementary Table 18).

Supplementary Table 18. Examples of companies marketing water on the basis of its old age

#	Category	Quote from webpage	Website	Date Accessed
1	Beer and spirits	"From deep within the Earth's crust, the finest drinking water on the planet springs forth to Memphis. This unique aquifer supplies WISEACRE with the most necessary of ingredients for the production of crisp, light-colored lager. ***The rains that fell to earth 3000 years ago*** are filtered very slowly through hundreds of feet of fine grain sand, culminating in a huge underground lake filled with 57 trillion gallons of virtually mineral-free water. From this prestigious water reserve, we supply Memphis with Sands, our one-of-a-kind Lager. Beauteous in its simplicity, it is very low in bitterness and full of delicious flavors of bread and crackery malt."	https://wiseacrebrew.com/memphis-sands	Jan. 6, 2022
2	Beer and spirits	"Founded in 2005 by Martin Townshend, this genuine real ale brewery is based in the leafy green heart of the nations hop growing region. As well as Nelson hops, Townshend's also has access to ***an aquifier (sic) of ancient water*** to brew with, drawn up from the Motueka aquifer."	https://townshendbrewery.co.nz/pages/story?_pos=1&_sid=af12fc6b2&_ss=r	Jan. 6, 2022
3	Beer and spirits	"Our water is a cherished natural resource, drawn straight from the Southern Hills Aquifer. ***These springs are over two thousand years old*** and provide water that is pristine and naturally perfect for brewing, which is why we decided to call the brand Spring Loaded"	https://beerconnoisseur.com/articles/abita-brewing-co-launches-spring-loaded-spiked-sparkling-water	Jan. 6, 2022
4	Bottled water	"***Water "up to 30,000 years old" has become a McCashin's signature ingredient*** for the Nelson brewery's beer, ciders and soft drinks." and "The water source has been scientifically carbon-dated as being between 14,000 and 30,000 years old."	https://www.rnz.co.nz/news/business/299274/bored-old-water-makes-for-exclusive-brews	Jan. 6, 2022
5	Bottled water	"***FIJI Water comes from an ancient aquifer*** deep within the earth on the island of Viti Levu, where it is protected and preserved from external impurities and remains untouched by man—until you unscrew the cap."	https://www.wonderful.com/brands/fiji/	Jan. 6, 2022
6	Spa	"***Hot spring water is considered old water, fossil water,*** ancient and irreplaceable. Heated by geothermal processes and emerging at 105° F., the water carries dissolved mineral salts reputed to have healing powers. The water contains calcium carbonate, calcium sulfate, sodium sulfate, sodium chloride, and lithium."	https://www.nps.gov/places/langford-hot-springs.htm	Jan. 7, 2022
7	Bottled water	"The water of the source emerges at the surface from a Well that is over 235 metres (770 feet) deep. ***Scientific studies have shown that this water can take up to 750 years to percolate through the 355 million year old limestones and dolomite fractures and joints before being drawn to the surface.*** The groundwater dissolves the minerals out of the limestones and dolomites to give Ballygowan its particular hydrochemical attributes."	https://www.ballygowan.ie/our-water-story/	Jan. 13, 2022
8	Bottled water	"Since the Roman times, healing properties have been attributed to water from St. Ann's Well, a geothermal spring in Buxton, Derbyshire in England. When the Romans arrived in Buxton, they built a bath around the spring, which they named Aquae Arnemetiae (The Waters of the Goddess of the Grove). ***The well, which taps 5,000-year-old rainwater that traveled across mineral-rich rock, was first mentioned as a holy well by William Worcester around 1460.***"	https://aleteia.org/2020/10/01/bottled-mineral-water-comes-from-original-st-anns-holy-well-in-england/	Jan. 13, 2022

Supplementary Note 8 – Map of US Aquifer Database

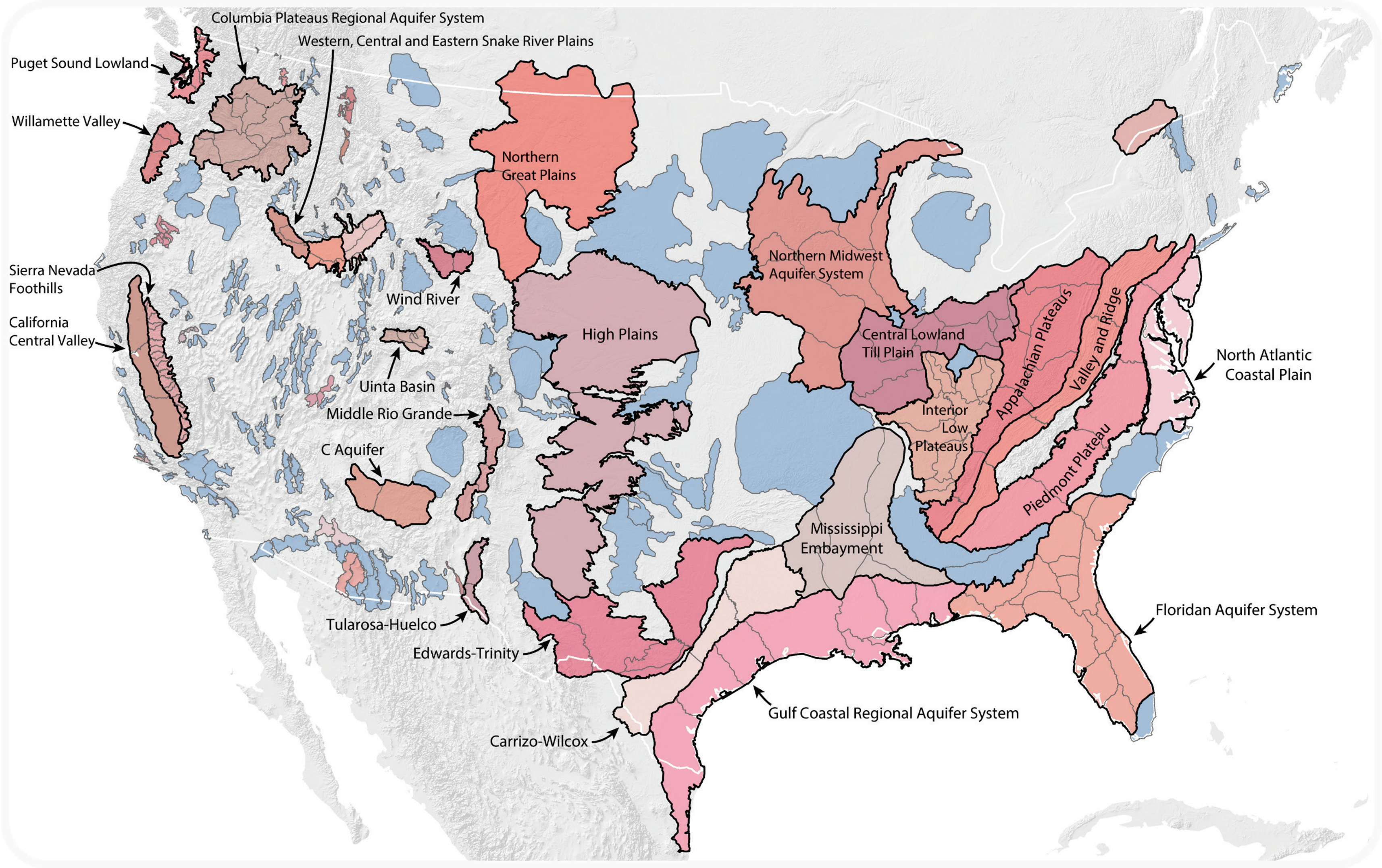

Supplementary Fig. 15. Selection of broader aquifer systems in the US. Each polygon with grey outlines represents one of the 440 polygons within the US Aquifer Database (see Data Availability Statement). A selection of expansive broader aquifer systems are outlined in black, and are labelled accordingly.
